# Label-free quantitative identification of abnormally ubiquitinated proteins as useful biomarkers for human lung squamous cell carcinomas

**DOI:** 10.1007/s13167-019-00197-8

**Published:** 2020-01-04

**Authors:** Miaolong Lu, Wei Chen, Wei Zhuang, Xianquan Zhan

**Affiliations:** 1 Key Laboratory of Cancer Proteomics of Chinese Ministry of Health, Xiangya Hospital, Central South University, 87 Xiangya Road, Changsha, 410008 Hunan People’s Republic of China; 2 Hunan Engineering Laboratory for Structural Biology and Drug Design, Xiangya Hospital, Central South University, 87 Xiangya Road, Changsha, 410008 Hunan People’s Republic of China; 3 State Local Joint Engineering Laboratory for Anticancer Drugs, Xiangya Hospital, Central South University, 87 Xiangya Road, Changsha, 410008 Hunan People’s Republic of China; 4 Shanghai Applied Protein Technology, Shanghai, 200233 People’s Republic of China; 5 Department of Thoracic Surgery, Xiangya Hospital, Central South University, 87 Xiangya Road, Changsha, 410008 Hunan People’s Republic of China; 6 Department of Oncology, Xiangya Hospital, Central South University, 88 Xiangya Road, Changsha, 410008 Hunan People’s Republic of China; 7 National Clinical Research Center for Geriatric Disorders, Xiangya Hospital, Central South University, 88 Xiangya Road, Changsha, 410008 Hunan People’s Republic of China

**Keywords:** Lung squamous cell carcinoma, Quantitative ubiquitinomics, Multiomics, Ubiquitin–proteasome system (UPS), Tumor inflammation, Cell adhesion, Metabolic reprogramming, Signal transduction, Ubiqitination-related biomarker, Predictive preventive personalized medicine (PPPM)

## Abstract

**Background:**

Ubiquitination is an important molecular event in lung squamous cell carcinoma (LSCC), which currently is mainly studied in nonsmall cell lung carcinoma cell models but lacking of ubiquitination studies on LSCC tissues. Here, we presented the ubiquitinated protein profiles of LSCC tissues to explore ubiquitination-involved molecular network alterations and identify abnormally ubiquitinated proteins as useful biomarkers for predictive, preventive, and personalized medicine (PPPM) in LSCC.

**Methods:**

Anti-ubiquitin antibody-based enrichment coupled with LC-MS/MS was used to identify differentially ubiquitinated proteins (DUPs) between LSCC and control tissues, followed by integrative omics analyses to identify abnormally ubiquitinated protein biomarkers for LSCC.

**Results:**

Totally, 400 DUPs with 654 ubiquitination sites were identified,, and motifs A-X (1/2/3)-K* were prone to be ubiquitinated in LSCC tissues. Those DUPs were involved in multiple molecular network systems, including the ubiquitin–proteasome system (UPS), cell metabolism, cell adhesion, and signal transduction. Totally, 44 hub molecules were revealed by protein–protein interaction network analysis, followed by survival analysis in TCGA database (494 LSCC patients and 20,530 genes) to obtain 18 prognosis-related mRNAs, of which the highly expressed mRNAs VIM and IGF1R were correlated with poorer prognosis, while the highly expressed mRNA ABCC1 was correlated with better prognosis. VIM-encoded protein vimentin and ABCC1-encoded protein MRP1 were increased in LSCC, which were all associated with poor prognosis. Proteasome-inhibited experiments demonstrated that vimentin and MRP1 were degraded through UPS. Quantitative ubiquitinomics found ubiquitination level was decreased in vimentin and increased in MRP1 in LSCC. These findings showed that the increased vimentin in LSCC might be derived from its decreased ubiquitination level and that the increased MRP1 in LSCC might be derived from its protein synthesis > degradation. GSEA and co-expression gene analyses revealed that VIM and MRP1 were involved in multiple crucial biological processes and pathways. Further, TRIM2 and NEDD4L were predicted as E3 ligases to regulate ubiquitination of vimentin and MRP1, respectively.

**Conclusion:**

These findings revealed ubiquitinomic variations and molecular network alterations in LSCC, which is in combination with multiomics analysis to identify ubiquitination-related biomarkers for in-depth insight into the molecular mechanism and therapeutic targets and for prediction, diagnosis, and prognostic assessment of LSCC.

**Electronic supplementary material:**

The online version of this article (10.1007/s13167-019-00197-8) contains supplementary material, which is available to authorized users.

## Introduction

Lung squamous cell carcinoma (LSCC) accounts for approximately 25–30% of all cases of nonsmall cell lung cancer (NSCLC) with more than 70% patients diagnosed in advanced stage [1, 2] and causes approximately 400,000 deaths per year worldwide [3]. Currently, surgery, radiation, and chemotherapy are still its main treatment. Early diagnosis and targeted drug therapy for LSCC remains a huge clinical challenge in LSCC [3]. The reason is that currently FDA-approved targeted drug therapies, such as epidermal growth factor receptor (EGFR) mutation or EML4-ALK fusion-based targeted therapies, are mainly suitable for lung adenocarcinoma (LUAD) but not for LSCC patients. Although FGFR1 amplification and DDR2 mutation are nominated as “druggable” targets for LSCC patients, their clinical efficacy are still under clinical trials [4, 5]. Early diagnosis and early therapy are an effective approach to improve the survival status of LSCC patients. However, currently, there is a lack of universally accepted biomarkers for early diagnosis and therapeutic targets for LSCC.

It is well-known that LSCC is a complex chronic disease with a series of molecular alterations at the levels of genome, transcriptome, and proteome, and is involved in extensive chronic inflammation in its pathogenesis [6–8]. Predictive, preventive, and personalized medicine (PPPM) is an effective strategy to treat LSCC patients [6–9]. Integrative omics-based biomarkers have important scientific merits for insights into the molecular mechanism, discovery of therapeutic targets, and service for early diagnosis and prognostic assessment of LSCC patients to reduce the mortality and improve patient prognosis [10, 11].

Proteome is the final performer of genome and transcriptome and stays in a dynamic balance between protein synthesis and degradation, which was mainly regulated by ubiquitination. Ubiquitination is a common post-translational modification (PTM) [12, 13]. Ubiquitination is involved in multistep reactions catalyzed by a series of enzymes, including ubiquitin-activating enzyme (E1), ubiquitin-conjugating enzyme (E2), and ubiquitin ligase (E3) [14]. Ubiquitin is a 76-amino-acid protein (8.5 kDa), whose carboxyl (C) terminus can be covalently bond to ε-amino group at protein lysine residue [15]. The ubiquitinated proteins are commonly degraded through the ubiquitin–proteasome system (UPS), which mainly degrades short half-life regulatory proteins and structural abnormal, misconstructed, or damaged proteins [16]. Besides protein-degradation functions, ubiquitination also participates in many nonprotein-degrading functions, including internalization and downregulation of receptors, DNA repair, inflammatory signaling, intracellular trafficking, autophagy, enzymatic activity regulation, and assembly of multiprotein complexes [17]. Thus, abnormal ubiquitination is associated with many diseases, including tumor, neurodegenerative disease, and inflammation [18]. For example, a study demonstrated that linear ubiquitination prevented inflammation and regulated immune signaling [19]. Inflammation and ubiquitination all participated in the progress of Alzheimer’s disease [20, 21]. As for tumors, UPS dysfunction could either enhance the effect of oncoproteins or reduce the amount of suppressor proteins. Deregulation of E3 ligases contributes to cancer development, and overexpression of E3 ligases is often associated with poor prognosis [22]. Also, E3 ligases can determine the specificity of protein substrates and are themselves “druggable” enzymes, which can serve as potential cancer targets as well as cancer biomarkers. Until now, many tumor therapeutic drugs have been developing based on UPS, such as bortezomib (FDA approved for multiple myeloma and mantle cell lymphoma) [23] and carfilzomib (FDA approved for relapsed and refractory multiple myeloma) [24]. It clearly demonstrates the scientific importance of protein ubiquitination in carcinogenesis and tumor-targeted therapy. Ubiquitinomics study may provide novel insights into the molecular mechanisms and discovery of effective biomarkers for early diagnosis and targeted drug therapy for PPPM in LSCC.

Liquid chromatography-tandem mass spectrometry (LC-MS/MS) is a key technique to characterize and quantify ubiquitinated proteins and ubiquitination sites. Although ubiquitination is a low abundance event in the human body, commercially specific anti-K-ε-GG antibodies enable to preferentially enrich tryptic ubiquitinated peptides before MS/MS analysis. Anti-ubiquitin antibody (specific anti-K-ε-GG group)-based label-free coupled with LC-MS/MS is an effective method to detect, identify, and quantify ubiquitinated proteins and ubiquitination sites, and more than 10,000 ubiquitination sites have been identified and quantified [25]. Currently, ubiquitinomes of lung cancer cells have been studied [26, 27]. However, tissue ubiquitinomics has not been reported in LSCC.

Moreover, lung cancer transcriptomics data based on RNA-seq and clinical information can be easily obtained from the public TCGA database, which contains several hundred lung cancer patients [3, 28]. There have been a large number of studies based on TCGA transcriptomics data and clinical information, which have contributed to PPPM in LSCC, and these works have been systematically reviewed [29]. Integration of ubiquitinomics data and large-scale transcriptomics data with useful clinical information will offer more valuable biomarkers for PPPM in LSCC.

This study used anti-K-ε-GG antibodies-based enrichment in combination with LC-MS/MS to identify differentially ubiquitinated proteins (DUPs) in human LSCC tissues compared to tumor-adjacent control tissues, followed by bioinformatics analysis to reveal functional characteristics of DUPs and ubiquitination-related molecular network alterations. Further, multiomics that integrated DUPs and transcriptomics data were used to investigate in-depth the clinical values of ubiquitination in LSCC for an in-depth understanding of molecular mechanisms, discovery of therapeutic targets, and identification of effective ubiquitination-related biomarkers for PPPM in LSCC.

## Materials and methods

### Tissue specimen

Human LSCC and tumor-adjacent control lung tissues from each lung cancer patient were obtained from the Department of Thoracic surgery, Xiangya Hospital, Central South University. This study was reviewed and approved by the Xiangya Hospital Medical Ethics Committee of Central South University, China. Once the tumor tissue was surgically removed, it was immediately stored in liquid nitrogen. A portion of each tissue sample was removed for pathological diagnosis, and the remainder was stored in an ultra-low temperature freezer (− 80 °C) for this study. Clinical characteristics of each sample was collected (Table 1).

**Table 1:** Clinical information of LSCC and control tissue samples

Sample ID	Sex	Age (years)	Smoking status	Pathological diagnosis	Tissues
1	Female	49	20 years	Right middle and upper LSCC	Cancer; tumor-adjacent normal control tissue
2	Female	57	40 years	Right middle and lower high-medium differentiated LSCC	Cancer; tumor-adjacent normal control tissue
3	Female	59	40 years	Right moderately differentiated LSCC	Cancer; tumor-adjacent normal control tissue
4	Male	60	Nonsmoker	Right moderately differentiated LSCC	Cancer; tumor-adjacent normal control tissue
5	Female	46	30 years	Right lower high-medium differentiated LSCC	Cancer; tumor-adjacent normal control tissue

*LSCC* = lung squamous cell carcinoma

### Protein extraction

Five LSCC tissue samples (*n* = 5; 150 mg per patient) were mixed as LSCC tissue sample (750 mg), and five corresponding tumor-adjacent control lung tissues (*n* = 5; 150 mg per patient) were mixed as lung control tissue sample (750 mg). The mixed LSCC tissues or control tissues were washed in 0.9% NaCl solution (3 mL, 5×) to remove blood contamination, and homogenized in urea lysis buffer including 7 M urea, 2 M thiourea, 100 mM dithiothreitol (DTT), and 1 mM phenylmethyl-sulfonyl fluoride (PMSF). Lysates were sonicated (80 W, 10 s, interval 15 s; 10×) and centrifuged (15,000×*g*, 20 min, 4 °C). The supernatant was collected as protein sample. Protein concentration was measured by the Bradford method.

### Trypsin digestion of proteins

Each sample was treated (600 rpm, 37 °C, and 1.5 h) with DTT (the final concentration of DTT was 10 mM) and was kept at room temperature. The DTT-treated sample was treated (dark, 30 min) with iodoacetamide (the final concentration of iodoacetamide was 50 mM). Then, uranyl acetate (UA) was added with an UA final concentration 2 M that was made from UA dilution by 50 mM Tris-HCl buffer (pH 8.0). Each protein sample was digested (37 °C, 15–18 h) with trypsin (trypsin:protein = 1:50 at wt:wt). After trypsin digestion, trifluoroacetic acid (TFA) was added (final TFA = 0.1%), and pH was adjusted to pH ≤ 3 with 10% TFA. The tryptic peptides were desalted with C18 Cartridges (Empore™ SPE Cartridges C18, bed i.d. 7 mm, volume 3 ml, Sigma) and lyophilized.

### Enrichment of ubiquitinated peptides

Each sample was reconstituted with a volume (1.4 mL) of precooled immunoaffinity purification (IAP) buffer (50 mM MOPS/NaOH pH 7.2, 10 mM Na_2_HPO_4_, and 50 mM NaCl). The pretreated anti-K-ε-GG antibody beads [PTMScan ubiquitin remnant motif (K-ε-GG) kit, Cell Signal Technology) were added to each tryptic peptide sample, incubated (4 °C, 1.5 h), and centrifuged (2000×*g*, 30 s). After the supernatant was discarded, the beads with anti-K-ε-GG antibody-binding tryptic peptides were washed with a volume (1 mL) of precooled IAP buffer (3×), and then washed with precooled water (3×). A volume (40 μL) of 0.15% TFA was added, followed by incubation (10 min, room temperature) (2×) and centrifugation (2000×*g*, 30 s). The supernatant was the enriched ubiquitinated peptide sample, which was processed to be desalted with C18 STAGE Tips.

### LC-MS/MS

Easy nLC (Proxeon Biosystems, now Thermo Fisher Scientific) coupled with Q Exactive mass spectrometer (Thermo Scientific) was used for LC-MS/MS analysis. The enriched peptides were loaded into a reverse-phase trap column (Thermo Scientific Acclaim PepMap100, 100 μm × 2 cm, nanoViper C18) and then online entered into C18-reversed phase analytical column (Thermo Scientific Easy Column, length 10 cm, i.d. 75 μm, and 3 μm resin) to be separated with buffer A (0.1% formic acid) and buffer B (84% acetonitrile and 0.1% formic acid), in a separation gradient of buffer B at a flow rate of 300 nL/min for 120 min. The mass spectrometer parameters were set as positive-ion mode, selection of a data-dependent top 10 precursor ions for MS/MS analysis with high-energy collision dissociation (HCD) at 30 eV, MS survey scan range *m/z* 300–1800, automatic gain control (AGC) 3e6, maximum inject time 10 ms, and dynamic exclusion duration 40.0 s. The resolution was 70,000 at *m*/*z* 200 for MS scan and 17,500 at *m*/*z* 200 for MS/MS scan. The MS/MS raw data for each sample were combined and searched with MaxQuant 1.5.3.17 software to identify and quantify ubiquitinated proteins and ubiquination sites. The main parameters were set as trypsin for enzyme, four missed cleavages, 6 ppm for MS tolerance, 20 ppm for MS/MS tolerance, database uniprot_human_156639_20170105.fasta, carbamidomethyl for fixed modification, oxidation at Met residue, acetylation at protein N-term, and GlyGly at K residue for variable modification, reverse for decoy database pattern, true for included contaminants, FDR ≤ 0.01 for peptide, FDR ≤ 0.01 for ubiquitination site, FDR ≤ 0.01 for protein, and 2 min for time window (match between runs).

Each ubiquitinated peptide and ubiquitination site was determined with amino acid sequence. The differential ubiquitination level of each ubiquitinated peptide was determined with the ratio (tumor/control) > 2.0 or < 0.5, and *p* value < 0.05. Proteins containing this type of differentially ubiquitinated peptides were defined as differentially ubiquitinated proteins (DUPs).

### Bioinformatics analysis of DUPs in LSCC

Motif-X (http://motif-x.med.harvard.edu/) was used to predict the ubiquitination motifs with the extracted amino acid sequences that contained the ubiquitination site and seven upstream/downstream amino acid residues from this ubiquitination site (totally 15 amino acid residues) [30, 31]. The Motif-X parameters were set as width 15, occurrences 20, background IPI human proteome, and significance threshold 0.0001. For DUPs, the DAVID software (version 6.8, https://david.ncifcrf.gov/) was used to carry out gene ontology (GO) enrichment analysis, including cellular components (CC), molecular functions (MF), and biological processes (BP), and then those DUPs were clustered into different functional categories [32], with statistical significance *p* < 0.05. The statistically significant pathway networks were mined with the Kyoto Encyclopedia of Genes and Genomes (KEGG) pathway analysis. The KEGG online service tool KOBAS (http://kobas.cbi.pku.cn) was used to annotate the proteins’ KEGG database description [33, 34]. The STRING database (https://string-db.org/) was used to analyze protein−protein interaction (PPI) networks [35]. The STRING results, XGMML format, were imported into Cytoscape (http://www.cytoscape.org/) to visualize the functional networks and calculate the topological properties of the nodes [36].

### LSCC transcriptomics data and statistical analysis

The level 3 gene expression RNA-seq data [20,531 genes; generated from 502 LSCC tissues (*n* = 502 patients) and 51 tumor-adjacent lung control tissues (those 51 lung control tissues belonged to those 502 patients)] and clinical information derived from 494 LSCC patients (those 494 patients had complete prognostic data, and belonged to those 502) in the TCGA database were obtained with UCSC Xena browser (https://xenabrowser.net/) [28]. R package pROC and survival analyses were used to calculate the receiver operating characteristic (ROC) curves for overall survival (OS) and recurrence-free survival (RFS) after initial therapy [37]. The optimal cutoff value for one specific gene was determined based on the Youden index [38]. Log-rank test was performed to assess the difference between the survival curves. The genes co-expressed with VIM and ABCC1 (|Spearman *r*| ≥ 0.5) were examined with cBioPortal for Cancer Genomics (http://www.cbioportal.org/) [39]. Single gene GSEA based on the TCGA database was used to explore the pathway differences between samples with high and low expressions of VIM and ABCC1 [40]. The E3-substrate interaction network (http://ubibrowser.ncpsb.org/) was used to predict the E3s of VIM and ABCC1 [41].

### Cell lines and western blot

Human LSCC cells H520 were purchased from Central South University (Changsha, China), H226 from the Chinese Academy of Sciences (Shanghai, China), and Calu-1 from the American Type Culture Collection (ATCC) (Manassas, USA). Three cell lines were all cultured in RPMI-1640 medium plus 10% fetal bovine serum (FBS, Gibco) (5% CO_2_ atmosphere, 37 °C). For inhibitory analysis of proteasome treated with 10 mM of proteasome inhibitor MG132, each cell line was incubated (6 h) and then lysed with lysis buffer [150 mM NaCl, 2 mM NaH_2_PO_4_, 50 mM Tris-HCl pH 7.5, 25 mM NaF, 1% (v/v) Triton X-100, 2 mM EDTA, 10% (v/v) glycerol, and protease/phosphatase inhibitor cocktails (Sigma)].

Equal amounts of protein samples were separated with 10% sodium dodecyl sulfate (SDS)–polyacrylamide gel electrophoresis (PAGE) and transferred onto polyvinylidene difluoride (PVDF) membranes. Proteins on PVDF membrane were incubated (4 °C, overnight) with primary antibodies against VIM-encoded protein vimentin (1:2000; CUSABIO), ABCC1-encoded protein MRP1 (multidrug resistance-associated protein 1) (1:1000; CUSABIO), IGF1R (1:1000; CUSABIO), and β-actin (1:2000, Santa Cruz Biotechnology), and then incubated (2 h, room temperature) with horseradish peroxidase-conjugated goat anti-rabbit secondary antibody (1:5000; Santa Cruz Biotechnology). ImageJ software (version 1.45s) was used to measure the gray value of the western blot results. The western blot image was digitized to calculate mean ± SD with Student’s *t* test (*p* < 0.05).

## Results

### DUP profiling in LSCC

**Fig. 1:**
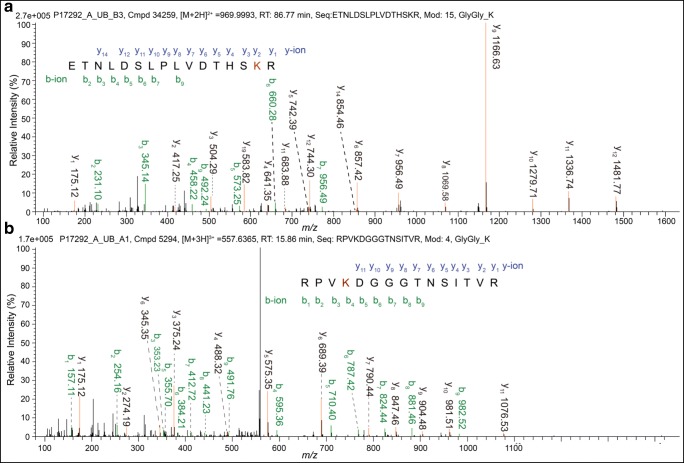
Representative MS/MS spectra of ubiquitinated peptides: ^425^ETNLDSLPLVDTHSK*R^440^ from vimentin (P08670) (**a**) and ^633^RPVK*DGGGTNSITVR^647^ from multidrug resistance-associated protein 1 (MRP1) (P33527) (**b**). K* = ubiquitinated lysine residue

A total of 400 DUPs with 654 ubiquitination sites were identified in LSCC vs. tumor-adjacent control tissues (Supplemental Table 1). A representative MS/MS spectrum of ubiquitinated peptide ^425^ETNLDSLPLVDTHSK*R^440^ (precursor ion [M+2H]^2+^*m*/*z* = 969.9993, retention time RT = 86.77 min, and K* = ubiquitinated lysine residue) from vimentin (P08670) was shown (Fig. 1a), with high signal to noise (S/N) ratio and excellent b-ion and y-ion series (b_2_, b_3_, b_4_, b_5_, b_6_, b_7_, b_9_, y_1_, y_2_, y_3_, y_4_, y_5_, y_6_, y_7_, y_8_, y_9_, y_10_, y_11_, y_12_, and y_13_). The ubiquitination site was localized at residue K_439_ in vimentin amino acid sequence, and its ubiquitination level was significantly decreased with a ratio of T/N (tumor/control) = 0.36 in LSCCs compared to controls (Supplemental Table 1). There is another representative MS/MS spectrum of ^633^RPVK*DGGGTNSITVR^647^ (precursor ion [M+3H]^3+^*m*/*z* = 557.6365, RT = 15.86 min, and K* = ubiquitinated lysine residue) from MRP1 (P33527) (Fig. 1b), with high S/N ratio and excellent b-ion and y-ion series (b_1_, b_2_, b_3_, b_4_, b_5_, b_6_, b_7_, b_8_, b_9_, y_1_, y_2_, y_3_, y_4_, y_5_, y_6_, y_7_, y_8_, y_9_, y_10_, and y_11_). The ubiquitination site was localized at residue K_636_ in MRP1 amino acid sequence, and its ubiquitination level was significantly increased in LSCCs compared to controls (Supplemental Table 1). Totally, 125 (31.25%) of 400 DUPs were found to have two or more identified ubiquitination sites. Among 654 ubiquitination sites, the ubiquitination levels of 104 sites were significantly increased and 131 were significantly decreased in LSCCs compared to controls, 346 ubiquitination sites were only quantitatively detected in LSCCs but not in controls, and 73 ubiquitination sites were only quantitatively detected in controls but not in LSCCs (Supplemental Table 1). Thus, totally, 450 (104 + 346) ubiquitination sites showed increased ubiquitination levels in LSCCs, and 207 (131 + 73) ubiquitination sites showed decreased ubiquitination levels in LSCCs.

### Ubiquitination motifs occurred in LSCC

**Fig. 2:**
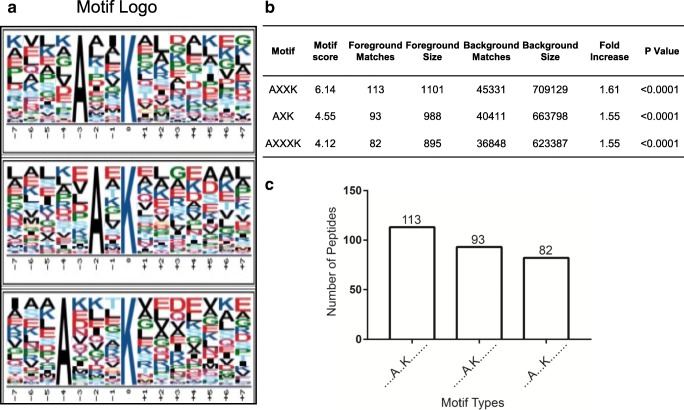
Ubiquitination motifs occurred in human LSCCs. **a** Potential ubiquitin recognition motif logos (AXXK, AXK, and AXXXK) in human LSCCs. **b** The statistically significant A-X (1/2/3)-K* motifs in LSCCs. **c** The number of ubiquitinated peptides among three motif types

Motif-X analysis revealed statistically significant ubiquitination motifs that were prone to be ubiquitinated in LSCC, based on those identified 654 ubiquitination sites in LSCC tissues. The results found that motifs A-X-K*, A-XX-K*, and A-XXX-K* (X = any amino acid residue, K* was the lysine residue that is prone to be ubiquitinated) were significantly prone to be ubiquitinated (Fig. 2). Of them, A-XX-K* was the most significant motif determined with 113 (113/654 = 17.3%) ubiquitination sites. The other two motifs A-X-K and A-XXX-K were determined with 93 (93/654 = 14.2%) and 82 (82/654 = 12.5%) ubiquitination sites, respectively. The ubiquitination motifs A-X(1/2/3)-K* showing alanine residue (A) in the upstream of the ubiquitination site (K*) had a certain influence on the occurrence of ubiquitination at K residue, but no amino acid residue in the downstream of the ubiquitination site (K*) was found to be meaningful for the occurrence of ubiquitination at K residue.

### Functional characteristics of DUPs in LSCC

GO enrichment analysis of 400 DUPs revealed 10 statistically significant functional clusters to comprehensively reflect the functional characteristics of DUPs in LSCC [42]. Comprehensive analysis of these functional clusters found those DUPs were involved in many cancer-related biological functions, such as cell–cell adhesion in cluster 1, regulation of the assemble of proteasome complex and UPS in clusters 2 and 3, transcriptional and translational regulations in cluster 6, cell signal transduction in cluster 2, and anti-tumor drug metabolism in cluster 8 (Table 2).

**Table 2:** The functional categories of 400 DUPs identified with GO analysis

Category	Term	*p* value
Annotation cluster 1		
GOTERM_MF_DIRECT	Cadherin binding involved in cell–cell adhesion	3.11E-18
GOTERM_CC_DIRECT	Cell–cell adherens junction	2.24E-17
GOTERM_BP_DIRECT	Cell–cell adhesion	1.23E-13
Annotation cluster 2		
GOTERM_CC_DIRECT	Proteasome accessory complex	4.60E-12
GOTERM_BP_DIRECT	Antigen processing and presentation of exogenous peptide antigen via MHC class I, TAP-dependent	1.59E-11
GOTERM_BP_DIRECT	Regulation of cellular amino acid metabolic process	3.49E-09
GOTERM_BP_DIRECT	NIK/NF-kappaB signaling	5.35E-09
GOTERM_BP_DIRECT	Negative regulation of ubiquitin-protein ligase activity involved in mitotic cell cycle	1.27E-08
GOTERM_BP_DIRECT	Stimulatory C-type lectin receptor signaling pathway	1.85E-08
GOTERM_BP_DIRECT	Positive regulation of ubiquitin-protein ligase activity involved in regulation of mitotic cell cycle transition	2.83E-08
GOTERM_BP_DIRECT	Anaphase-promoting complex-dependent catabolic process	4.43E-08
GOTERM_BP_DIRECT	Tumor necrosis factor-mediated signaling pathway	8.37E-08
GOTERM_BP_DIRECT	Positive regulation of canonical Wnt signaling pathway	1.04E-07
GOTERM_BP_DIRECT	T cell receptor signaling pathway	2.37E-07
GOTERM_BP_DIRECT	Fc-epsilon receptor signaling pathway	4.90E-07
GOTERM_BP_DIRECT	Protein polyubiquitination	7.65E-07
GOTERM_BP_DIRECT	Negative regulation of canonical Wnt signaling pathway	4.36E-06
Annotation cluster 3		
GOTERM_CC_DIRECT	Proteasome regulatory particle, base subcomplex	4.88E-10
GOTERM_CC_DIRECT	Nuclear proteasome complex	1.00E-09
GOTERM_CC_DIRECT	Cytosolic proteasome complex	7.28E-09
GOTERM_MF_DIRECT	Proteasome-activating ATPase activity	1.54E-08
GOTERM_BP_DIRECT	Positive regulation of RNA polymerase II transcriptional preinitiation complex assembly	3.41E-07
GOTERM_MF_DIRECT	TBP-class protein binding	2.10E-06
GOTERM_BP_DIRECT	Positive regulation of proteasomal protein catabolic process	1.05E-05
GOTERM_BP_DIRECT	Protein catabolic process	1.41E-04
Annotation cluster 4		
GOTERM_BP_DIRECT	Regulation of ventricular cardiac muscle cell action potential	1.11E-03
GOTERM_MF_DIRECT	Cell-adhesive protein binding involved in bundle of His cell–Purkinje myocyte communication	5.30E-03
Annotation cluster 5		
GOTERM_CC_DIRECT	Haptoglobin–hemoglobin complex	2.43E-05
GOTERM_CC_DIRECT	Endocytic vesicle lumen	1.71E-04
GOTERM_MF_DIRECT	Haptoglobin binding	1.10E-03
GOTERM_BP_DIRECT	Positive regulation of cell death	1.90E-02
GOTERM_CC_DIRECT	Hemoglobin complex	1.97E-02
GOTERM_MF_DIRECT	Oxygen transporter activity	2.90E-02
GOTERM_BP_DIRECT	Oxygen transport	3.43E-02
Annotation cluster 6		
GOTERM_BP_DIRECT	SRP-dependent cotranslational protein targeting to membrane	9.85E-05
GOTERM_BP_DIRECT	Nuclear-transcribed mRNA catabolic process, nonsense-mediated decay	5.83E-04
GOTERM_BP_DIRECT	Viral transcription	1.69E-03
GOTERM_BP_DIRECT	Translational initiation	5.82E-03
GOTERM_CC_DIRECT	Ribosome	3.40E-02
Annotation cluster 7		
GOTERM_MF_DIRECT	Voltage-gated anion channel activity	5.30E-03
GOTERM_MF_DIRECT	Porin activity	5.30E-03
GOTERM_CC_DIRECT	Pore complex	1.11E-02
GOTERM_BP_DIRECT	Anion transport	3.02E-02
GOTERM_BP_DIRECT	Regulation of anion transmembrane transport	4.34E-02
Annotation cluster 8		
GOTERM_BP_DIRECT	Daunorubicin metabolic process	1.00E-02
GOTERM_BP_DIRECT	Doxorubicin metabolic process	1.00E-02
Annotation cluster 9		
GOTERM_BP_DIRECT	Nucleotide-excision repair, DNA damage recognition	1.00E-02
GOTERM_BP_DIRECT	Global genome nucleotide-excision repair	2.47E-02
Annotation cluster 10		
GOTERM_MF_DIRECT	Neutral amino acid transmembrane transporter activity	1.82E-02
GOTERM_BP_DIRECT	Neutral amino acid transport	2.62E-02

### Ubiquitination-involved molecular network alternations in LSCC

**Fig. 3:**
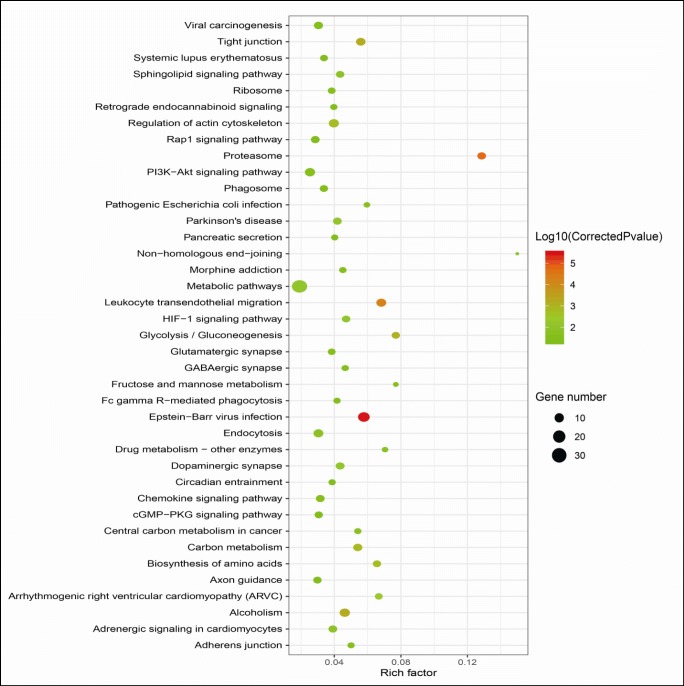
Ubiquitination-involved pathway–network alterations in human LSCCs. A total of 39 significantly significant enriched pathways (*p* < 0.05 and FDR < 0.05) were identified. The darker dot means the more significant enrichment. The size of the dot represents the number of DUPs enriched in the pathway

KEGG pathway network analysis of 400 DUPs revealed 39 significant KEGG pathways (*p* < 0.05 and FDR < 0.05) (Fig. 3, Supplemental Table 2). Comprehensive analysis of all pathways demonstrated that DUPs were mainly enriched in four important tumor-related molecular network systems, including the UPS, cell energy metabolism, cell–cell adhesion, and cell signal transduction. Insights into these ubiquitination-involved molecular network changes might reveal the roles of ubiquitination in the carcinogenesis process of LSCC.

#### Ubiquitin–proteasome system

The proteasome is a pivotal component of UPS for ubiquitin-mediated proteolysis. The 26S proteasome is a complex, including two 19S regulatory particles (PA700) and one 20S core particle. This study discovered three DUPs (Rpn3, Rpn5, and Rpn6) in PA700 (Lid) and seven DUPs (Rpn13, Rpt1–Rpt6) in PA700 (Base). Their ubiquitination levels were significantly increased at residues K_34_ (ratio T**/**N = 2.27) in Rpn13; K_293_ (T+/N−) in Rpt2; K_46_ (ratio T**/**N = 4.96) in Rpt1; K_372_ (T+/N−) in Rpt5; K_273_ (T+/N−) in Rpt4; K_346_ (T+/N−), K_330_ (T+/N−), and K_290_ (T+/N−) in Rpt6; K_194_ (T+/N−), K_328_ (T+/N−), and K_62_ (ratio T**/**N = 2.37) in Rpt4; K_273_ (T+/N−) in Rpn3; K_32_ (T+/N−) in Rpn6; and K_147_ (T+/N−) in Rpn5. The ubiquitination level was significantly decreased at residue K_53_ (ratio T**/**N = 0.33) in Rpt5 (Supplemental Fig. 1.1).

#### Cell energy metabolism

Abnormal ubiquitinations in cell energy metabolism-related pathways might contribute to tumor metabolic reprogramming that is a hallmark of cancer. KEGG pathway analysis revealed that DUPs were involved in cell energy metabolism, including glycolysis/gluconeogenesis, carbon metabolism, central carbon metabolism, and fructose and mannose metabolism in cancer. In addition, most DUPs (HK1, GAPDH, ALDOA, ENO1, and PGK1) in HIF-1 signaling pathway were involved in glucose metabolism; thus, HIF-1 signaling pathway was also classified into cell energy metabolism (Supplemental Fig. 1.6). For glycolysis pathway, all 12 ubiquitination sites within 8 DUPs (including PKM, P14618, a rate-limiting enzyme) had the significantly increased ubiquitination levels; of these, ubiquitination at residue K270 in PKM was only detected in LSCC tissues, which indicated that ubiquitination might affect glycolysis (Supplemental Fig. 1.2). For fructose and mannose metabolism pathway, this study identified four DUPs, whose ubiquitination levels were significantly increased at residues K_111_ (ratio T/N = 3.84) and K_200_ (ratio T/N = 3.8) in P04075; K_117_ (T+/N−), K_186_ (ratio T/N = 16.91), K_194_ (ratio T/N = 2.67), and K_215_ (ratio T/N = 3.22) in P04406; K_97_ (T+/N−) in B3KXY9; and K_168_ (T+/N−) in P60174 (Supplemental Fig. 1.3). For central carbon metabolism of cancer pathway, this study identified six DUPs, whose ubiquitination levels were significantly increased at K_97_ (T+/N−) in B3KXY9, K_270_ (T+/N−) in P14618, K_178_ (T+/N−) and K_362_ (ratio T/N = 7.19) in Q15758, K_270_ (ratio T/N = 110.93) and K_502_ (T+/N−) in Q59GX2, K_19_ (T+/N−) in Q01650, and K_431_ (T+/N−) in A0A024R8U1 (Supplemental Fig. 1.4). For carbon metabolism pathway, this study identified 10 DUPs, whose ubiquitination levels were significantly increased at K_380_ (T+/N−) and K_57_ (T+/N−) in O43175; K_59_ (ratio T/N = 2.66) and K_377_ (ratio T/N = 16.52) in P52209; K_130_ (T+/N−) in A0A140VK56; K_81_ (T+/N−) in A0A24R4F1; K_111_ (ratio T/N = 3.84) and K_200_ (ratio T/N = 3.8) in P04075; K_117_ (T+/N−), K_215_ (ratio T/N = 3.22), K_194_ (ratio T/N = 2.67), and K_186_ (ratio T/N = 16.91) in P04406; K_97_ (T+/N−) in B3KXY9; K_216_ (T+/N−) in P00558; K_270_ (T+/N−) in P14618; and K_168_ (T+/N−) in P60174 (Supplemental Fig. 1.5). For carbon metabolism pathway, this study identified eight DUPs, whose ubiquitination levels were significantly increased at K_299_ (T+/N−) in A0A0S2Z3S6; K_502_ (T+/N−) and K_270_ (ratio T/N = 110.93) in Q59GX2; K_97_ (T+/N−) in B3KXY9; K_1033_ (T+/N−) in P08069; K_117_ (T+/N−), K_215_ (ratio T/N = 3.22), K_194_ (ratio T/N = 2.67), and K_186_ (ratio T/N = 16.91) in P04406; K_81_ (T+/N−) in A0A024RF1; K_111_ (ratio T/N = 3.84) and K_200_ (ratio T/N = 3.8) in P04075; and K_216_ (T+/N−) in P00558 (Supplemental Fig. 1.6).

#### Cell–cell adhesion

Epithelial cell adhesion-associated pathways included tight junction and adherens junction, which participated in sustaining cell polarity and regulating cell proliferation and differentiation. For tight junction pathway, this study found that ubiquitination levels were significantly increased at residues K_304_ (T+/N−), K_338_ (ratio T/N = 9.37), K_336_ (ratio T/N = 21.89), K_326_ (ratio T/N = 5.07), K_96_ (ratio T/N = 4.74), and K_60_ (ratio T/N = 4.98) in tuba; K_794_ (T+/N−) in integrin; K_13_ (T+/N−) in PCNA; K_62_ (T+/N−) in PP2A (A0A140VJT0); K_312_ (T+/N−) in actin 4; K_380_ (T+/N−) in myosin (A0A024QZJ4); and K_679_ (T+/N−), K_1410_ (T+/N−), and K_972_ (ratio T/N = 5.40) in myosin (A0A024R1N1) and were significantly decreased at residues K_151_ (T−/N+), K_360_ (ratio T/N = 0.09), and K_162_ (ratio T/N = 0.14) in ERM; K_620_ (ratio T/N = 0.41) in myosin (P35580); K_135_ (ratio T/N = 0.32) in RhoA; K_21_ (ratio T/N = 0.48) in PP2A (A0A140VJS0); and K_257_ (ratio T/N = 0.05) and K_239_ (T−/N+) in claudin (Supplemental Fig. 1.7). For the adherens junction pathway, this study found that ubiquitination levels were significantly increased at residues K_312_ (T+/N−) in A0A024R694; K_1033_ (T+/N−) in P08069; K_935_ (T+/N−) in A0A024RC65; K_119_ (T+/N−) in A0A024R324; K_749_ (T+/N−), K_676_ (T+/N−), K_810_ (T+/N−), and K_355_ (ratio T/N = 4.99) in O60716; and K_147_ (T+/N−) in A0A024R1P2 and were significantly decreased at residues K_161_ (ratio T/N = 0.2) in A0A024RC65 and K_135_ (ratio T/N = 0.32) in A0A024R324 (Supplemental Fig. 1.8).

#### Cell signal transduction

Cell signal transduction pathways included PI3K-AKT, RAP1, and cGMP–PKG signaling pathways. For the PI3K-AKT pathway, 13 DUPs were identified, and the ubiquitination levels were significantly increased at residues K_1033_ (T+/N−) in IGFIR, K_62_ (T+/N−) in PP2A (A0A140VJT0), K_517_ (T+/N−) and K_587_ (T+/N−) in SYK, K_95_ (T+/N−) and K_624_ (T+/N−) in HSP90, K_617_ (T+/N−) in EPHA2, and K_794_ (T+/N−) in ITGB1 and were significantly decreased at residues K_23_ (ratio T**/**N = 0.15) in GNB2, K_23_ (ratio T/N = 0.12) in GNB1, K_34_ (T−/N+) in GNG12, K_21_ (ratio T/N = 0.48) in PP2A (A0A140VJS0), K_106_ (ratio T/N = 0.43) in 14-3-3, K_11_ (T−/N+) in GNB5, and K_957_ (T−/N+) in the extracellular matrix (ECM) (Supplemental Fig. 1.9). For RAP1 signaling pathway, nine DUPs were identified, and the ubiquitination levels were significantly increased at residues K_46_ (T+/N−) in P04899; K_147_ (T+/N−) in A0A024R1P2; K_92_ (T+/N−) in P08754; K_119_ (T+/N−) in A0A024R324; K_617_ (T+/N−) in A0A024QZA8; K_794_ (T+/N−) in P05556; K_749_ (T+/N−); K_676_ (T+/N−), K_406_ (T+/N−), K_810_ (T+/N−), and K_355_ (ratio T/N = 4.99) in O60716; K_91_ (T+/N−) in P07737; and K_1033_ (T+/N−) in P08069 and were significantly decreased at residues K_135_ (ratio T/N = 0.32) in A0A024R324 (Supplemental Fig. 1.10). For the cGMP–PKG signaling pathway, this study identified eight DUPs, and the ubiquitination levels were significantly increased at residues K_605_ (T+/N−), K_444_ (T+/N−), K_468_ (ratio T/N = 22.88), K_661_ (T+/N−), K_212_ (T+/N−) in P05023; K_128_ (T+/N−) in A0A0S2Z3L2; K_75_ (T+/N−) in A0A024R968; K_12_ (T+/N−) in P21796; K_12_ (T+/N−) in Q9Y277; K_46_ (T+/N−) in P04899; K_92_ (T+/N−) in P08754; and K_119_ (T+/N−) in A0A024R324 and were significantly decreased at residue K_135_ (ratio T/N = 0.32) in A0A024R324 (Supplemental Fig. 1.11).

### Identification of hub molecules with PPI analysis of DUPs

**Fig. 4:**
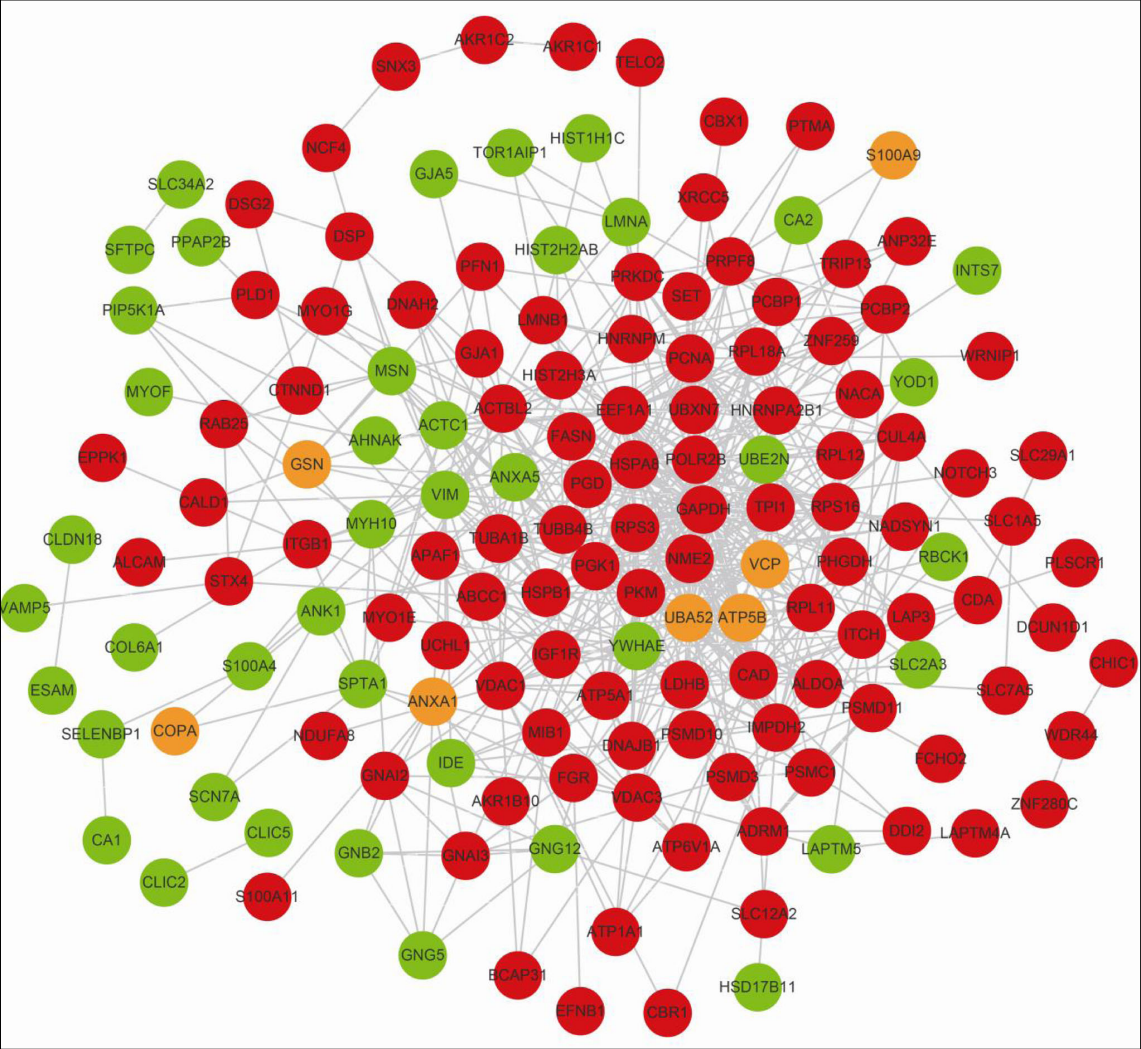
Protein–protein interaction (PPI) network in human LSCCs. Red node means the increased ubiquitination level in LSCCs. Green node means the decreased ubiquitination level in LSCCs. Yellow node means multiple ubiquitination sites in a protein with reverse (some increased and some decreased) ubiquitination levels in LSCCs

All 400 DUPs were input into the STRING software to construct PPI networks. The PPI results were imported into Cytoscape software in combination with ubiquitination intensity change of each DUP. After the isolated and partially connected nodes were removed, a complex network of DUPs was constructed (Fig. 4). The red node represented the increased intensities of all identified ubiquitination sites in one protein, the green node represented the decreased intensities of all identified ubiquitination sites in one protein, and the yellow node indicated at least two ubiquitination sites in a protein with inconsistent ubiquitination intensities. A total of 44 molecules were identified as hub molecules with topology property degrees ≥ 10, among which 11.4% (5/44) molecules, including vimentin (VIM) (degree = 21), ACTC1 (degree = 18), YWHAE (degree = 14), ANXA5 (degree = 12), and UBE2N (degree = 11), had the decreased ubiquitination levels; 9.1% (4/44) molecules, including UBA52 (degree = 43), ATP5B (degree = 19), VCP (degree = 14), and ANXA1 (degree = 10), had at least two ubiquitination sites with inconsistent ubiquitination levels; and the rest of the molecules (35/44 = 79.5%) had an average degree of 17.7 and increased ubiquitination levels (Supplemental Table 3).

### Ubiquitination affects LSCC patient’s outcome by regulating intracellular abundance of vimentin and MRP1

Survival analysis of 44 hub molecules was performed based on LSCC RNA-seq and clinical information of 494 LSCC patients from the TCGA database, which identified 18 prognosis-related mRNAs, including MRP1, IGF1R, VIM, ATP5A1, TUBA1B, VDAC1, HSPA8, PCNA, ATP5B, ACTC1, HSPB1, ITCH, ANXA5, UBE2N, UBXN7, VDAC3, PRPF8, and PHGDH (Table 3). The high expressions of seven mRNAs (ATP5A1 and IGF1R in OS; VDAC1, VIM, and PRPF8 in RFS; and ITCH and ANXA5 in both OS and RFS) were associated with poorer survival status, while the high expressions of other nine mRNAs (ACTC1 and UBXN7 in OS; TUBA1B, HSPA8, PCNA, ATP5B, HSPB1, VDAC3, and PHGDH in RFS; and UBE2N and ABCC1 in both OS and RFS) were associated with better survival status (Supplemental Fig. 2).

**Table 3:** Ubiquitination status of 18 prognosis-related molecules

UniProt accession	Gene symbol	Description	Number of modified sites	Modified peptides	Ratio (T/N)
P25705	ATP5A1	ATP synthase subunit alpha	1	VGLK*APGIIPR	NA
P68363	TBA1B	Tubulin alpha-1B chain	6	AYHEQLSVAEITNACFEPANQMVK*CDPR	NA
				DVNAAIATIK*TKR	9.37
				DVNAAIATIKTK*R	21.89
				GDVVPK*DVNAAIATIK	5.07
				QLFHPEQLITGK*EDAANNYAR	4.74
				TIGGGDDSFNTFFSETGAGK*HVPR	4.98
P21796	VDAC1	Voltage-dependent anion-selective channel protein 1	1	AVPPTYADLGK*SAR	NA
P11142	HSPA8	Heat shock cognate 71 kDa protein	7	AMTK*DNNLLGK	NA
				CNEIINWLDK*NQTAEKEEFEHQQK	2.04
				ELEK*VCNPIITK	6.59
				GTLDPVEK*ALR	NA
				LDK*SQIHDIVLVGGSTR	NA
				MVQEAEK*YKAEDEK	2.55
				NQTAEKEEFEHQQK*ELEK	NA
P12004	PCNA	Proliferating cell nuclear antigen	1	ILK*CAGNEDIITLR	NA
P08670	VIM	Vimentin	9	ETNLDSLPLVDTHSK*R	0.36
				FLEQQNK*ILLAELEQLKGQGK	0.29
				ILLAELEQLK*GQGK	0.49
				K*LLEGEESR	0.26
				K*VESLQEEIAFLK	0.17
				LREK*LQEEMLQR	0.41
				RQVDQLTNDK*AR	0.21
				RQVQSLTCEVDALK*GTNESLER	0.36
				TLLIK*TVETR	0.26
P06576	ATP5B	ATP synthase subunit beta	1	VLDSGAPIK*IPVGPETLGR	NA
P68032	ACTC1	Actin, alpha cardiac muscle 1	1	VAPEEHPTLLTEAPLNPK*ANR	0.46
P04792	HSPB1	Heat shock protein beta-1	1	AQLGGPEAAK*SDETAAK	NA
P08069	IGF1R	Insulin-like growth factor 1 receptor	1	VAIK*TVNEAASMR	NA
Q96J02	ITCH	E3 ubiquitin-protein ligase Itchy homolog	2	FIYGNQDLFATSQSK*EFDPLGPLPPGWEK	3.05
				VYYVDHVEK*R	NA
P08758	ANXA5	Annexin A5	4	GAGTNEK*VLTEIIASR	0.38
				HALK*GAGTNEK	0.44
				LIVALMK*PSR	NA
				LYDAYELK*HALK	0.17
P61088	UBE2N	Ubiquitin-conjugating enzyme E2 N	2	DK*WSPALQIR	0.14
				ICLDILK*DK	0.26
O94888	UBXN7	UBX domain-containing protein 7	1	DVWSNEAVK*NIIR	NA
Q9Y277	VDAC3	Voltage-dependent anion-selective channel protein 3	1	CNTPTYCDLGK*AAK	NA
Q6P2Q9	PRPF8	Pre-mRNA-processing-splicing factor 8	2	DLILADYGKK*	NA
				DLILADYGK*K	NA
O43175	PHGDH	D-3-phosphoglycerate dehydrogenase	2	NAGNCLSPAVIVGLLK*EASK	NA
				SATK*VTADVINAAEK	NA
P33527	ABCC1	Multidrug resistance-associated protein 1	3	RPVK*DGGGTNSITVR	NA
				TYQVAHMKSK*	NA
				TYQVAHMK*SK	NA

**Fig. 5:**
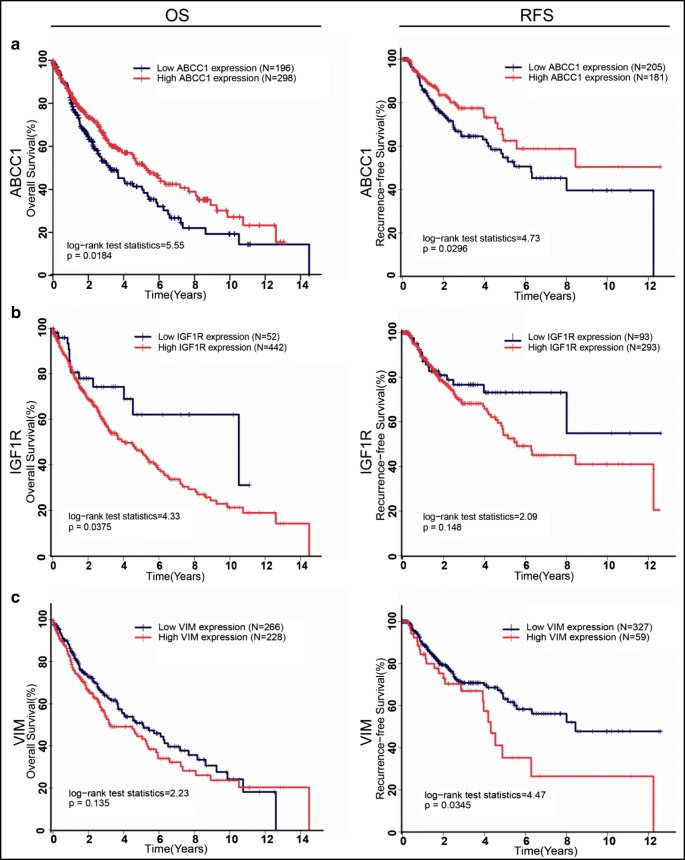
Survival analysis of ABCC1, IGF1R, and VIM in human LSCCs. **a** ABCC1 had significant prognostic value in both overall survival (OS) rate and recurrence free survival (RFS) rate. **b** IGF1R had significant prognostic value in OS rate but not in RFS rate. **c** VIM had significant prognostic value in RFS rate but not in OS rate

**Fig. 6:**
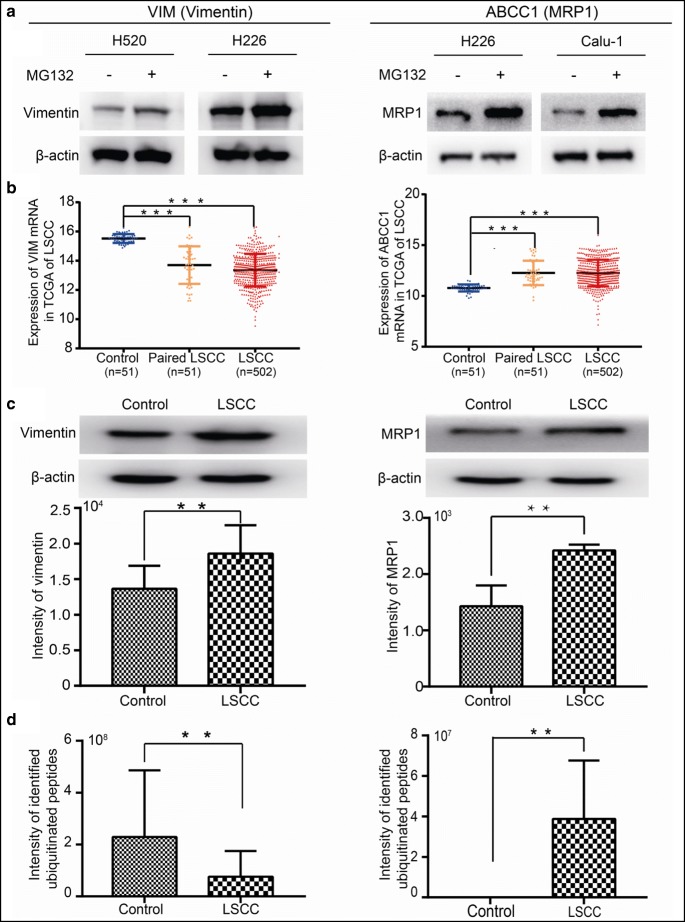
Comprehensive analysis of vimentin and MRP1 in human LSCCs. **a** Proteasome inhibitor MG-132 induced the increase of vimentin in LSCC cell lines H520 and H226, and MRP1 in H226 and calu-1. **b** In the TCGA database, the expression of VIM mRNA was downregulated in 502 LSCC tissues (LSCC) compared with 51 adjacent lung tissue samples (control). VIM expression was also decreased in 51 paired LSCC tissues (paired LSCC) and their adjacent lung tissue samples (control), while the expression of ABCC1 was upregulated in 502 LSCC tissues (LSCC) compared with 51 adjacent lung tissue samples (control). ABCC1 expression was also increased in 51 paired LSCC tissues (paired LSCC) and their adjacent lung tissue samples (control). **c** Western blotting showed both vimentin and MRP1 were increased in LSCC tissues (LSCC) compared with adjacent control tissues (control). β-Actin was detected as a loading control in the western blot. **d** The intensity of ubiquitinated peptides of vimentin and MRP1 in adjacent lung tissue samples (control) and LSCC tissues. Three independent experiments were conducted for each assay. * represent *p* value < 0.05, ** *p* value < 0.01 and *** *p* value < 0.001

Because post-transcriptional/translational modifications caused inconsistencies between mRNA and protein levels, further analysis of 18 prognosis-related mRNAs with literature review found that IGF1R, vimentin (VIM encoded), and MRP1 (ABCC1 encoded) were prognosis-related proteins in LSCC [43–45]. For MRP1, its high mRNA expression was associated better survival status (Fig. 5a); however, its high protein expression was associated with poorer survival status [43]. For IGF1R and VIM (vimentin), their high mRNA and protein expressions were all associated with poorer survival status compared to their low expressions (Fig. 5b, c). The abundances of vimentin and MRP1 were increased in the proteasome inhibitor MG132-treated LSCC cell lines (H520 and H226 for vimentin; H226 and Calu-1 for MRP1), indicating that vimentin and MRP1 were degraded through the UPS (Fig. 6a). Based on LSCC TCGA data, the mRNA level of VIM was significantly downregulated (Fig. 6b). The mRNA level of MRP1-encoded ABCC1 was significantly upregulated (Fig. 6b). Both vimentin and MRP1 were increased in the protein level in LSCC tissues (vimentin: fold change = 1.28; MRP1: fold change = 1.78) (Fig. 6c). The ubiquitination intensities at residues K_129_, K_139_, K_168_, K_188_, K_223_, K_334_, K_402_, K_439_, and K_445_ in vimentin were significantly decreased in LSCC tissues, with an average ratio = 0.38 (*p* < 0.01) (Fig. 6d). The ubiquitination intensities at residues K_496_, K_498_, and K_636_ in MRP1 were significantly increased in LSCC tissues with the average intensity of ubiquitination 3.8 × 10^7^, while those three ubiquitination sites were only detected in LSCC tissues (Fig. 6d). These results demonstrated that the increased protein expression of vimentin in LSCC was mainly derived from its decreased ubiquitination levels in LSCC, which caused the poorer survival status in LSCC, whereas the increased protein expression of MRP1 in LSCCs was mainly derived from its mRNA ABCC1 high expression in LSCC. Although the higher ubiquitination level increased MRP1 degradation, this degradation did not offset MRP1 synthesis due to its highly expressed mRNAs; thus, MRP1 was still increased in the protein level to cause the poorer survival status in LSCC. These results clearly demonstrated that ubiquitination regulated the intracellular protein abundance of vimentin and MRP1 by affecting the degradation of both proteins, which in turn affects the patient’s prognosis.

### Identification of downstream biological processes and pathways mediated by vimentin and MRP1 in LSCC

**Fig. 7:**
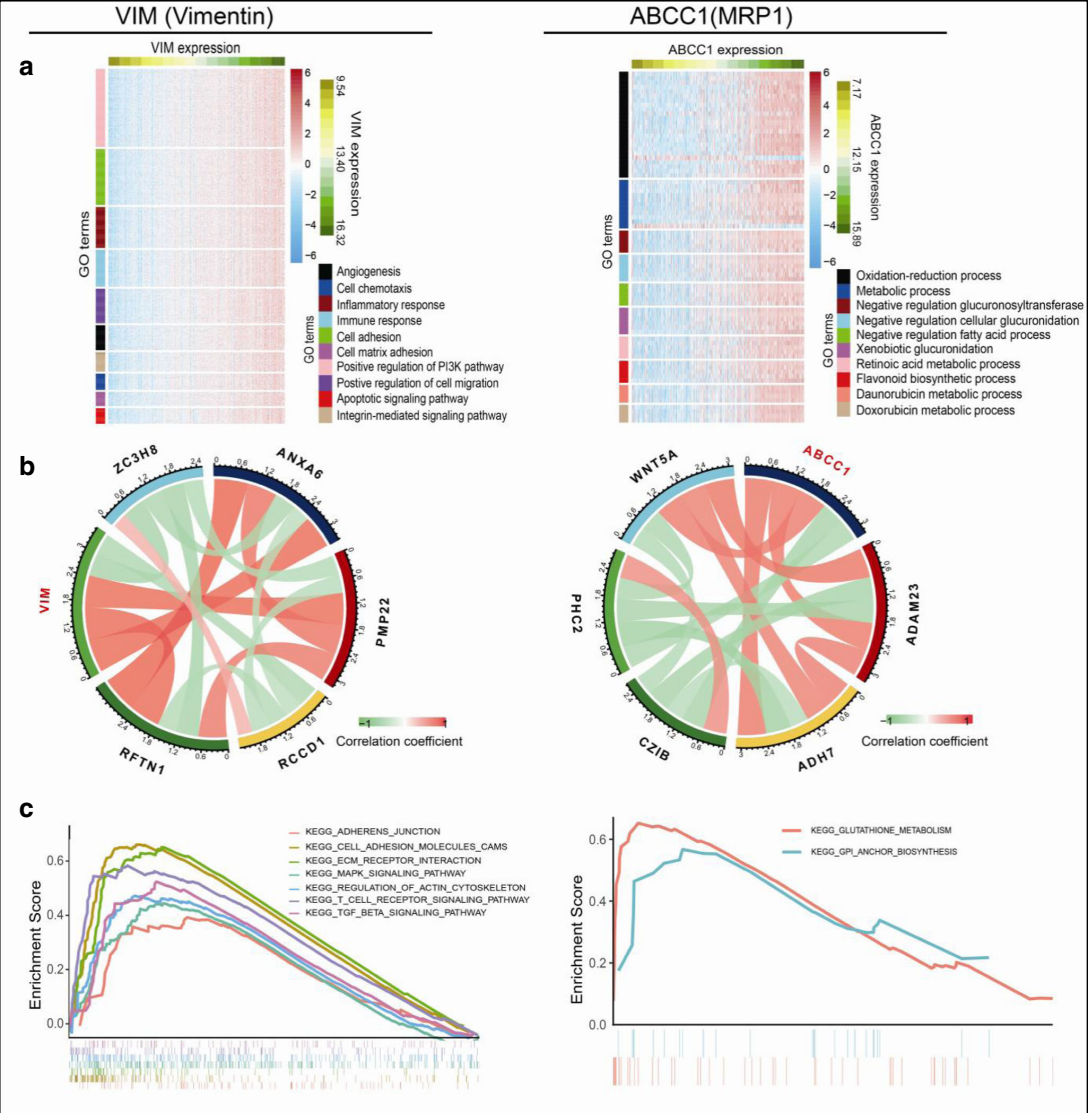
Roles of vimentin and MRP1 in human LSCCs. **a** Based on cBioPortal and TCGA databases, GO analysis revealed important BPs of 1173 co-expressed genes for VIM and 183 co-expressed genes for ABCC1 in heat map. Each row represents the gene enriched in each GO term, and the color intensity is determined by the mRNA expression level of this gene. Each column represents a sample derived from TCGA database of human LSCC. All samples were sorted from low to high according to the mRNA expression levels of VIM and ABCC1. **b** Co-expressed genes of VIM and ABCC1 with high correlation coefficient. **c** GSEA analysis revealed tumor-related KEGG pathways positively correlated with high expression of VIM and ABCC1

To further clarify the roles of the ubiquitinated vimentin and MRP1 in LSCC patient prognosis, the downstream biological processes and pathways mediated by vimentin and MRP1 were predicted. Totally, 1173 co-expressed genes were screened out for VIM (encoded vimentin) (Supplemental Table 4), and 183 co-expressed genes for ABCC1 (encoded MRP1) (Supplemental Table 5). Co-expressed genes of VIM were mainly involved in 65 statistically significant biological processes (Fig. 7a, Supplemental Table 6). Co-expressed genes of ABCC1 were mainly involved in 15 statistically significant biological processes (Fig. 7a, Supplemental Table 7). Among those co-expressed genes, ZC3H8, ANXA6, PMP22, RCCD1, and RFTN1 had high correlation coefficient with VIM, respectively, and WNT5A, ADAM23, ADH7, CZIB, and PHC2 had high correlation coefficient with ABCC1, respectively (Fig. 7b). Moreover, single gene GSEA analysis revealed that VIM was positively related to 15 statistically significant KEGG pathways (Fig. 7c, Supplemental Table 8), including cell migration, ECM receptor interaction, cell adhesion, immunity and inflammation, cytokine, cell apoptosis, MAPK signaling, and Jak–STAT signaling pathway. The high expression of ABCC1 was mainly related to 11 statistically significant KEGG pathways (Fig. 7c, Supplemental Table 9), including glutathione metabolism, porphyrin and chlorophyll metabolism, starch and sucrose metabolism, ascorbate and aldarate metabolism, steroid hormone biosynthesis, glycosaminoglycan biosynthesis, and glycosylphosphatidylinosito GPI anchor biosynthesis.

### Prediction of TRIM2 and NEDD4L as E3 ligases of vimentin and MRP1 in LSCC

**Fig. 8:**
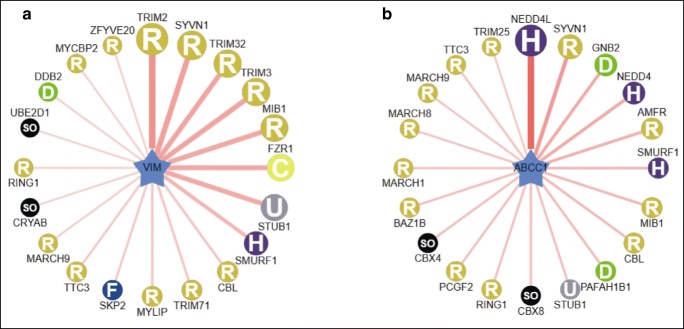
Potential upstream mechanisms that cause ubiquitination of vimentin and of MRP1. **a** Top 20 potential E3s of vimentin. **b** Top 20 potential E3s of ABCC1. Note: MRP1 is coded by gene ABCC1. The solid line means VIM or ABCC1 directly interacts with its E3-substrates. The line thickness means the interaction intensity. TRIM2 has the highest interaction intensity with vimentin. NEDD4L has the highest interaction intensity with MRP1. H, R, D, U, F, and SO are the subfamilies of E3s. H: HECT (homologous to the E6-AP carboxyl terminus) E3 ligases. R: RING-finger E3 ligases. D: CUL4-DDB1-DWD (Cullin 4-Damaged DNA Binding1-DDB1 binding WD40) E3 ligases. U: U-box E3 ligases. F: F-box E3 ligases. SO: single other E3 ligases such as CRYAB, CKS1B, UBE3C, and BRCC3

Totally, 30 E3s were predicted to catalyze the formation of vimentin ubiquitination (Supplemental Table 10), and 45 E3s were predicted to catalyze the formation of MRP1 ubiquitination (Supplemental Table 11). The top 20 E3s were shown for vimentin and MRP1, respectively (Fig. 8). The top 5 E3s for vimentin ubiquitination come from the RING family, including TRIM2, SYVN1, TRIM32, TRIM3, and MIB1, with the confidence score from 0.687 to 0.728, and TRIM2 has the highest confidence score of 0.728. The top 5 E3s for MRP1 were NEDD4L, SYVN1, GNB2, NEDD4, and AMFR, with a confidence score from 0.668 to 0.829, and NEDD4L had the highest confidence score of 0.829. It indicated that TRIM2 and NEDD4L might be the E3 ligases for vimentin and MRP1 in LSCC tissues, respectively.

## Discussion

Ubiquitination is an important molecular event in LSCC, and the study on ubiquitination might lead to clarify new molecular mechanism and promote the PPPM of LSCC. This study reported the first ubiquitinomics study in LSCC tissues and identified 400 DUPs with 654 ubiquitination sites. These DUPs were mainly involved in four molecular network systems, including UPS, cell energy metabolism, cell–cell adhesion, and cell signal transduction, which are the precious resource to identify abnormally ubiquitinated protein biomarkers for PPPM practice in LSCC. Ubiquitination-involved molecular network alterations not only reflect the roles of ubiquitination in the occurrence and development of LSCC, but also provide an important data to mine biomarkers for tumor diagnosis, prognosis, and new therapeutic targets from the components of UPS (such as E3 ligases and proteasome). Those ubiquitinomics data in combination with transcriptomics data and clinical data (*n* = 494 LSCC patients) from the TCGA database discovered two prognosis-related DUPs (vimentin and MRP1). Ubiquitination regulated the intracellular protein abundance of vimentin and MRPI to affect the prognosis of LSCC patients, namely ubiquitination-mediated highly expressed vimentin and MRPI were associated with the poor prognosis of LSCC patient. Here, we will discuss in detail the ubiquitination-related molecular network alterations and clinical application value of ubiquitination in combination with multiomics in LSCC.

### Proteasome subunit ubiquitination contributes to UPS dysfunction

The proteasome is a pivotal component of UPS to degrade the short-lived regulatory proteins, and remove the damaged soluble proteins [46]. Proteasome dysfunction decreases proteolytic activities and increases the accumulation of misfolded or damaged proteins, which may contribute to cancer pathogenesis [47]. The 26S proteasome is consisted of two 19S regulatory cap subunits and one 20S subunit. Two 19S subunits are indispensable for the normal function of 20S subunit. For example, Rpn10 and Rpn13 were important recognition receptors of ubiquitinated target proteins [48, 49]. Further, except for ubiquitination, many other PTMs such as phosphorylation, acetylation, and myristoylation were also identified in those subunits. Thus, these multiple PTMs greatly complicated the mechanisms to modulate proteasome activities. Studies found that mono-ubiquitination in Rpn10 was involved in the recruitment of substrate and interacted proteasome shuttle factor in *Drosophila* [50, 51], while the amino acid deprivation that induced ubiquitination of multiple 19S proteasome subunits (Rpn1, Rpn10, and Rpn13) was essential for autophagy of proteasome [52]. All the six identified DUPs in LSCC tissues belonged to 19S regulatory subunit, namely three ATPase subunits (PSMC1, PSMC4, and PSMC6) and three non-ATPase subunits (PSMD3, PSMD11, and PSMD12), which showed that ubiquitination of these 19S regulatory subunits might affect the assembly and activity of proteasome. Previous studies discovered that PSMD11 was required for proteasome assembly and played a crucial role in elevated proteasome activity in embryonic stem cells [53]. Moreover, acetylation [54], phosphorylation [55], and SUMO modification [56] were also found in PSMD11, and our study found ubiquitination in PSMD11 for the first time and identified one ubiquitination site at residue K_32_ only in LSCC tissues. At present, there are few studies on the effects of ubiquitination on the functions of various subunits of proteasome. However, considering the important functions of these proteasome subunits, these identified DUPs might reflect the functional abnormalities of proteasome in LSCC tissues compared to control tissues and ultimately lead to the imbalance of intracellular proteins. Therefore, our identified ubiquitination of proteasome subunits benefit for the in-depth understanding of UPS functionary regulations.

### Ubiquitination plays an important role in LSCC metabolic reprogramming

Metabolic reprogramming is a hallmark of cancer [57]. Tumor cells remodel their metabolism and energy production through confining energy metabolism mostly to glycolysis even under aerobic conditions, namely “aerobic glycolysis” [58]. This study revealed that DUPs were significantly involved in cellular energy metabolism-related pathways such as glycolysis/gluconeogenesis and central carbon metabolism in cancer. Here, glycolysis/gluconeogenesis pathways were taken for example; seven DUPs were identified, namely triosephosphate, phosphoglycerate kinase 1, pyruvate kinase (PK), fructose-bisphosphate aldolase A, glyceraldehyde-3-phosphate dehydrogenase (GAPDH), enolase 1, and L-lactate dehydrogenase B chain (LDH), and those DUPs were all enzymes and played important roles in the regulation of glycolysis and were associated with cancer. For example, GAPDH (P04406) was a glycolytic enzyme to specifically catalyze glyceraldehyde-3-phosphate (G-3-P) to D-glycerate 1, 3-bisphosphate. Although GAPDH was commonly regarded as a constitutive housekeeping gene, recent studies revealed that its expression status varied in different cancers [59]. Compared to normal lung tissue, GAPDH in both mRNA and proteins levels were upregulated in lung cancer tissue, which might contribute to the increased “aerobic glycolysis” [60]. Besides glycolysis, GAPDH as a multifunctional protein also participated in numerous biological processes [61]. For example, GAPDH bond to another cellular energy metabolism-related DUP-LDH to form transcriptional coactivator complex, which clearly demonstrated the relationship of energy metabolism and gene transcription [62]. Pyruvate kinase (PK, P14618) was another cancer-related protein to catalyze the last irreversible reaction in the glycolytic pathway. PK was overexpressed in lung cancer and necessary for aerobic glycolysis [63]. Moreover, the overexpressed PK promoted tumor growth, and phosphorylation at residue Tyr_105_ in PKM2 might also contribute to the tumor growth [64]. Ubiquitinated K_270_ in PKM was only identified in LSCC tissues, which was important because it was both the substrate-binding site and transition state stabilizer site. The ubiquitination of key enzymes in the glycolysis pathway affected their abundance and (or) functions, which in turn affected the metabolic processes of the entire tumor cells. Further, there was a close relationship between cellular energy metabolism and UPS. Proteasome degradation of proteins required ATP consumption, so insufficient energy production would inevitably impair UPS. Conversely, abnormal UPS function might affect the turnover of key proteins in energy metabolism, which affected the energy production of cells. Abnormal energy metabolism and abnormal function of proteasome formed a vicious circle.

### Abnormal ubiquitination is closely related to tumor invasion and distant metastasis

The abnormal cell adhesion is an important cause of tumor invasion and distant metastasis. Our study found that DUPs were mainly enriched in two cell adhesion-associated pathways—tight junction and adherens junction. Tight junction was taken as an example to explain the effect of ubiquitination on cell adhesion. A variety of proteins in tight junction were associated with invasion and metastasis of tumor. This study identified CLDN18 (P56856), belonging to the large protein family of claudin, participated in the maintenance of epithelial and endothelial tight junctions. Considering the inconsistent changes in CLDN among different tumor types, claudins had a high tissue specificity, which might be specific biomarkers for many types of cancers [65]. In addition, some preclinical studies found that CLDNs could be novel anti-tumor drug targets for cancer cells with high expression of CLDNs [66]. The exact function of CLDN18 in LSCC remains unknown. Ubiquitination at residue K_239_ in CLDN18 was only identified in normal tissue, and the ubiquitination intensity at residue K_257_ in CLDNs was downregulated. However, the effect of ubiquitination at residues K_239_ and K_257_ in CLDN18 remains unclear, and it is worth further exploring in the next step. Another DUP, ITGB1 (P05556) belonged to the integrin family, which is linked with various proteins in ECM and actin cytoskeleton to support cell adhesion and anchorage, which was crucial for tissue maintenance and repair in their structural role [42]. ITGB1 was an important beta subset, because it could regulate cell migration and was regarded as a prometastatic gene for lung cancer [67]. ITGB1 also participated in signal transduction; for example, ITGB1 was an important part of IL1B receptor and essential for IL1B signaling [68]. Those findings clearly demonstrated that abnormal ubiquitination contributed to tumor invasion and distant metastasis.

### Ubiquitination in combination with other PTMs regulates signaling pathways

Ubiquitination as a common PTM also coexists with other PTMs to regulate cell signaling pathways, such as sustaining proliferative signaling that is another hallmark of tumor [56]. The above discussed tumor energy metabolism-related pathways and cell adhesion-related pathways were closely related to cell signal transduction system. The intermediate products of energy metabolism-related pathways and downstream molecules of cell adhesion-related pathway were important cell signaling molecules. For instance, GAPDH could regulate transcription as part of the transcription complex [61], while integrin interacted directly with GTPases family and played an important role in cell signal transduction [69]. This study found multiple tumor-related cell signal transduction pathways, including PI3K-AKT, RAP1 signaling, and cGMP-PKG signaling pathways. Some molecules in these pathways were closely related to tumorigenesis, which were identified as DUPs, including Rac (A0A024R1P2), RhoA (A0A024R324), PP2A (A0A140VJT0, A0A140VJS0), ITGB1 (P05556), and IGF1R (P08069). A lot of literature showed the relationship between these molecules and cancers, and found that ubiquitination and other PTMs such as phosphorylation had some similarities and crosstalk. For example, ubiquitination and phosphorylation all consumed ATPs and were reversible enzyme-catalyzed reactions. Also, phosphorylation could regulate E3 ligase activity, create phospho-degrons, and regulate substrate localization, whereas ubiquitination could degrade protein kinases or activate protein kinases in some circumstances [70]. It is well-known that residues lysine, serine, threonine, and tyrosine often co-exist in a same protein. Thus, multiple PTMs such as ubiquitination or acetylation at residue lysine and phosphorylation at residues serine, threonine, and tyrosine might simultaneously occur in the same protein in a given condition. The cross-talks among multiple PTMs in a protein might greatly complicate biological network regulation processes and protein functions. Therefore, ubiquitination in combination with other PTMs might play important roles in regulating signaling pathway in LSCC.

### Multiomics integration analysis as a powerful tool to promote PPPM

Biological omics is driving the paradigm shift of cancer research and treatment from a single-parameter model to a multiparameter model [10, 11]. Meanwhile, PPPM strategy in cancer requires multiomics integration analysis, which can systematically explore the molecular mechanisms behind tumorigenesis [8, 9, 71–73], and this study is an excellent example. The integration analysis of DUP profile and transcriptomics data with clinical information from the TCGA database (*n* = 494 LSCC patients) identified two prognosis-related DUPs (vimentin and MRP1), which emphasized the important clinical value of ubiquitination for LSCC patient prognosis. Vimentin along with actin microfilaments and microtubules makes up the cytoskeleton to maintain cell shape and integrity of the cytoplasm [74]. Vimentin also functions as an organizer of a number of other important proteins involved in cell adhesion, signal transduction, and migration [74]. Recently, vimentin is identified as an epithelial–mesenchymal transition (EMT) biomarker, but one does not know the exact roles of vimentin in the EMT process [74]. Our study found that vimentin might affect EMT by participating in adherens junction, cell adhesion molecules cams, and MAPK signaling pathway. MRP1 is a member of the superfamily of ATP-binding cassette transporters to deliver various anti-tumor drugs to outside of tumor cells; thus, the decreased drug concentration inside of a cell will weaken its anticancer effect [75]. The UPS plays an important role in the regulation of intracellular abundance of vimentin and MRP1. Therefore, an in-depth analysis of the biological functions mediated by vimentin and MRP1 can benefit for the precise development of UPS-targeted anti-tumor drugs. Also, these analyses can provide a basis for the combined application of anti-tumor drugs. For example, one study demonstrated the important role of MRP1 in doxorubicin (DOX) resistance and the inhibitory effects of MK571 (MRP1 inhibitor) in the DOX efflux and resistance in NSCLCs [76]. Thus, the combination of one drug targeting UPS to promote MRP1 degradation and an inhibitor of MRP1 might improve the prognosis of the patient more than a single drug alone. Similarly, our study found that vimentin was involved in regulating MAPK signaling pathway. It might be more effective to combine the use of a drug promoting vimentin degradation and a drug targeting the MAPK pathway. By now, only two DUPs (vimentin and MRP1) have been identified to be degraded by UPS and be associated with LSCC prognosis. However, we strongly believe that multiomics integration analysis can be used as a powerful tool to explore the role of ubiquitination in carcinogenesis and promote PPPM practice in LSCC.

## Conclusions and expert recommendations

Label-free quantitative ubiquitinomics is an effective approach to identify DUPs and ubiquitination sites in human LSCC tissues. A total of 400 DUPs were identified in human LSCC vs. control tissues. GO and KEGG analyses revealed the important roles of ubiquitination in tumorigenesis and progress, and ubiquitination-involved molecular network alternations in LSCC, including UPS, energy metabolism, cell adhesion, and signal transduction. These findings not only reflect the important roles of ubiquitination in LSCC, but also provide a precious scientific data to mine biomarkers for tumor diagnosis, prognosis, and new therapeutic targets, for example, based on the identified DUPs in UPS (such as E3 ligases and proteasome). Further, integration analysis of DUP profile and transcriptomics data with clinical information from TCGA database (*n* = 494 LSCC patients) identified two DUPs (vimentin and MRP1), which were regulated by UPS to cause the high expressions of vimentin and MRP1 that were associated with poor prognosis of LSCC. Thus, the ubiquitination levels of vimentin and MRP1 might be used as a biomarker for prognosis of LSCC patients. These findings clearly demonstrated that multiomics integration analysis can be regarded as a power tool to mine abnormally ubiquitinated protein biomarkers and new drug targets for PPPM practice in LSCC.

We recommend this article to promote ubiquitinome-based signaling pathway network analysis in LSCC from a systems biology angle, and emphasize the importance of multiomics such as ubiquitinomics in combination with transcriptomics and large-scale clinical data in the basic research and translational research for PPPM in LSCC [74]. Here, we propose that ubiquitination-involved signaling pathway network alterations in combination with multiomics analysis to identify reliable biomarkers are an effective approach to clarify molecular mechanisms and discover effective therapeutic targets for personalized treatment of LSCC. Also, those DUPs and survival-related hub molecules are precious resources to discover real pattern biomarkers for LSCC.

## Electronic supplementary material

ESM 1 (PPT 1245 kb)

ESM 2 (PPT 844 kb)

ESM 3 (PDF 97 kb)

ESM 4 (PDF 41 kb)

ESM 5 (PDF 42 kb)

ESM 6 (PDF 75 kb)

ESM 7 (PDF 40 kb)

ESM 8 (PDF 55 kb)

ESM 9 (PDF 37 kb)

ESM 10 (PDF 33 kb)

ESM 11 (PDF 33 kb)

ESM 12 (PDF 7 kb)

ESM 13 (PDF 8 kb)
