# Supplementary material for: Label-free quantitative identification of abnormally ubiquitinated proteins as useful biomarkers for human lung squamous cell carcinomas
Source: EPMA J. 2020 Jan 4;11(1):73–94. doi: 10.1007/s13167-019-00197-8 (PMC7028901; doi:10.1007/s13167-019-00197-8)
Supplement: Supplementary file 1 — (PPT 1245 kb) [file 13167_2019_197_MOESM1_ESM.ppt]

## Slide 1
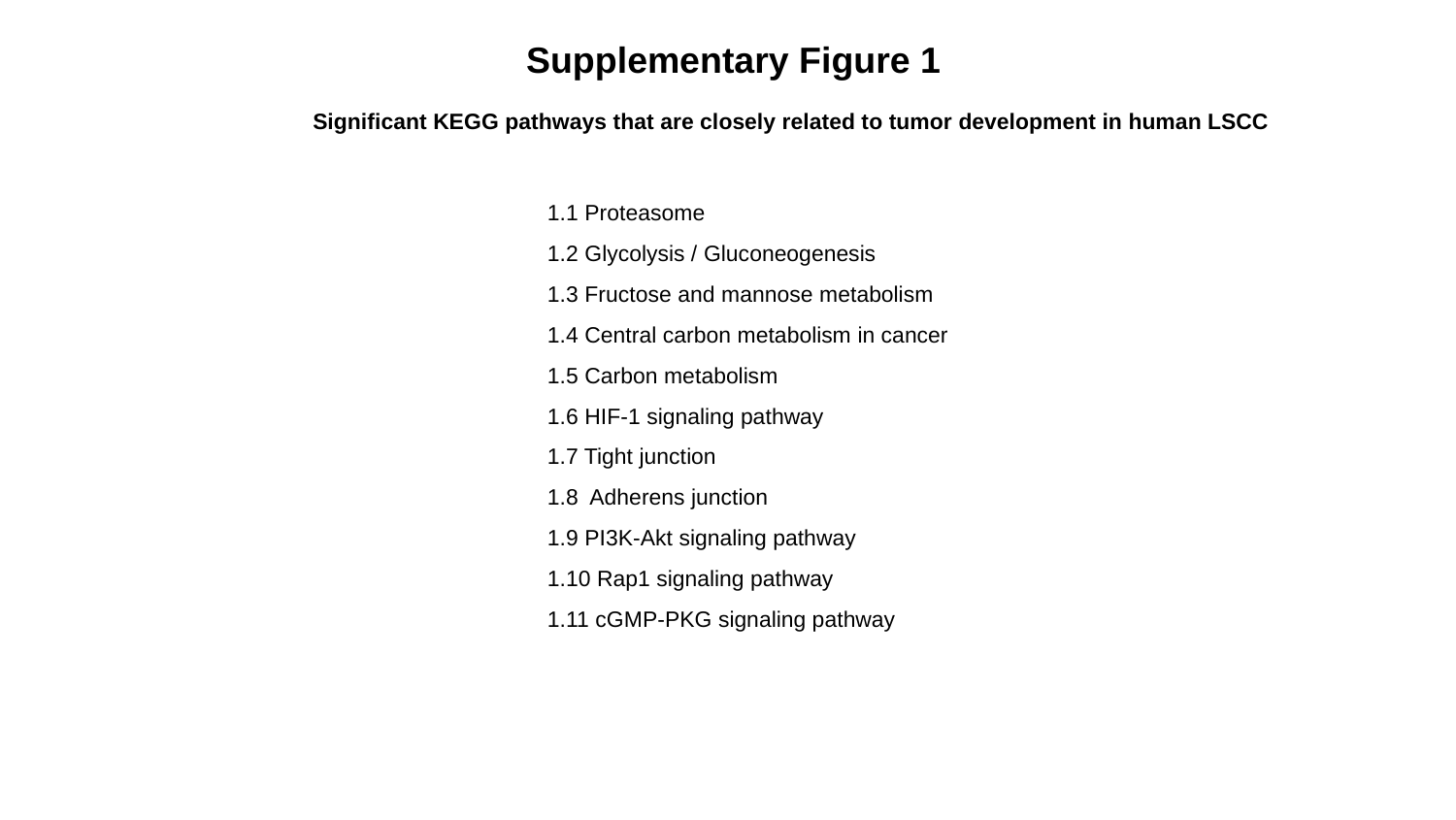

Supplementary Figure 1
Significant KEGG pathways that are closely related to tumor development in human LSCC
1.1 Proteasome
1.2 Glycolysis / Gluconeogenesis
1.3 Fructose and mannose metabolism
1.4 Central carbon metabolism in cancer
1.5 Carbon metabolism
1.6 HIF-1 signaling pathway
1.7 Tight junction
1.8 Adherens junction
1.9 PI3K-Akt signaling pathway
1.10 Rap1 signaling pathway
1.11 cGMP-PKG signaling pathway

## Slide 2
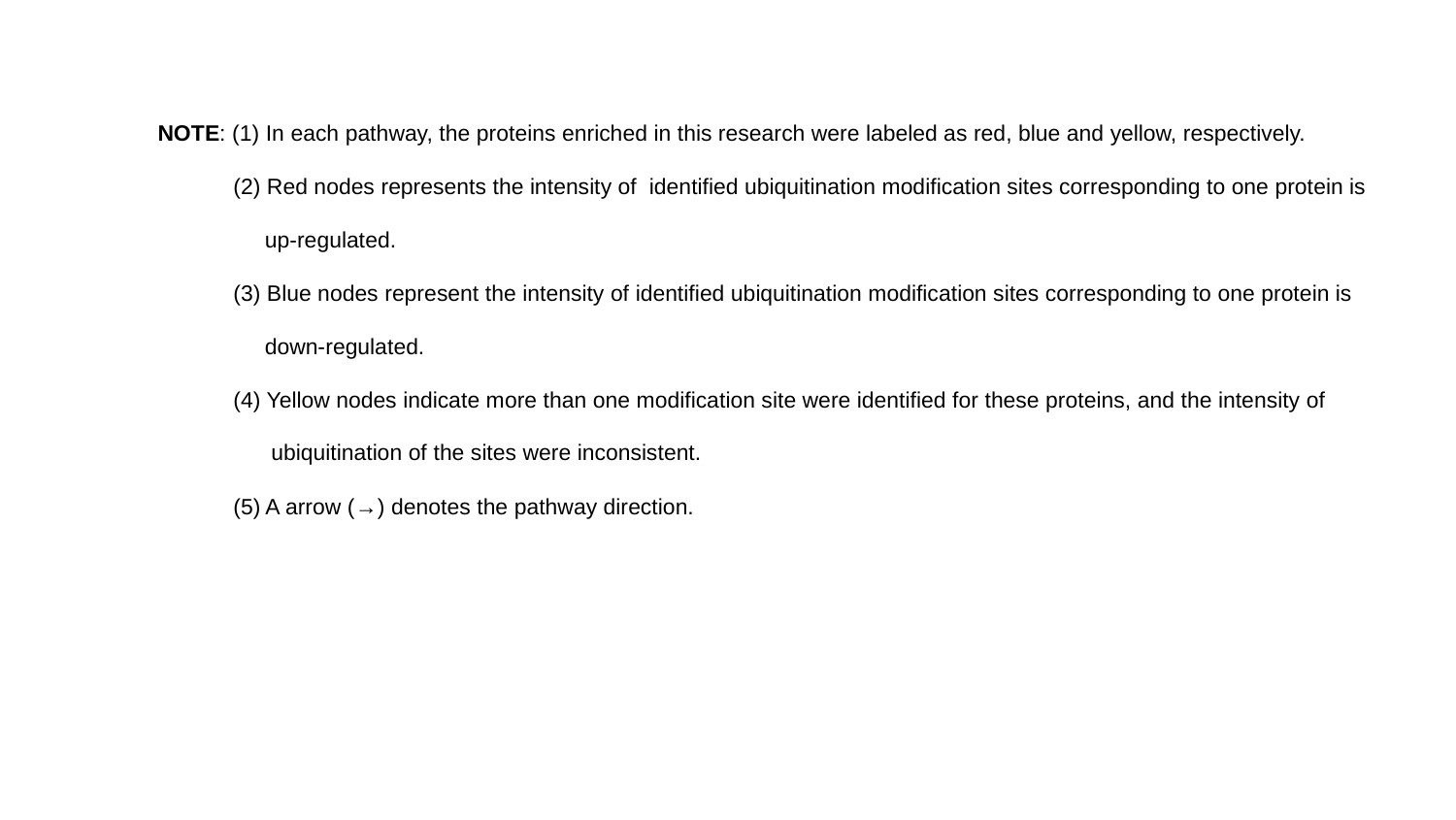

NOTE: (1) In each pathway, the proteins enriched in this research were labeled as red, blue and yellow, respectively.
 (2) Red nodes represents the intensity of identified ubiquitination modification sites corresponding to one protein is
 up-regulated.
 (3) Blue nodes represent the intensity of identified ubiquitination modification sites corresponding to one protein is
 down-regulated.
 (4) Yellow nodes indicate more than one modification site were identified for these proteins, and the intensity of
 ubiquitination of the sites were inconsistent.
 (5) A arrow (→) denotes the pathway direction.

## Slide 3
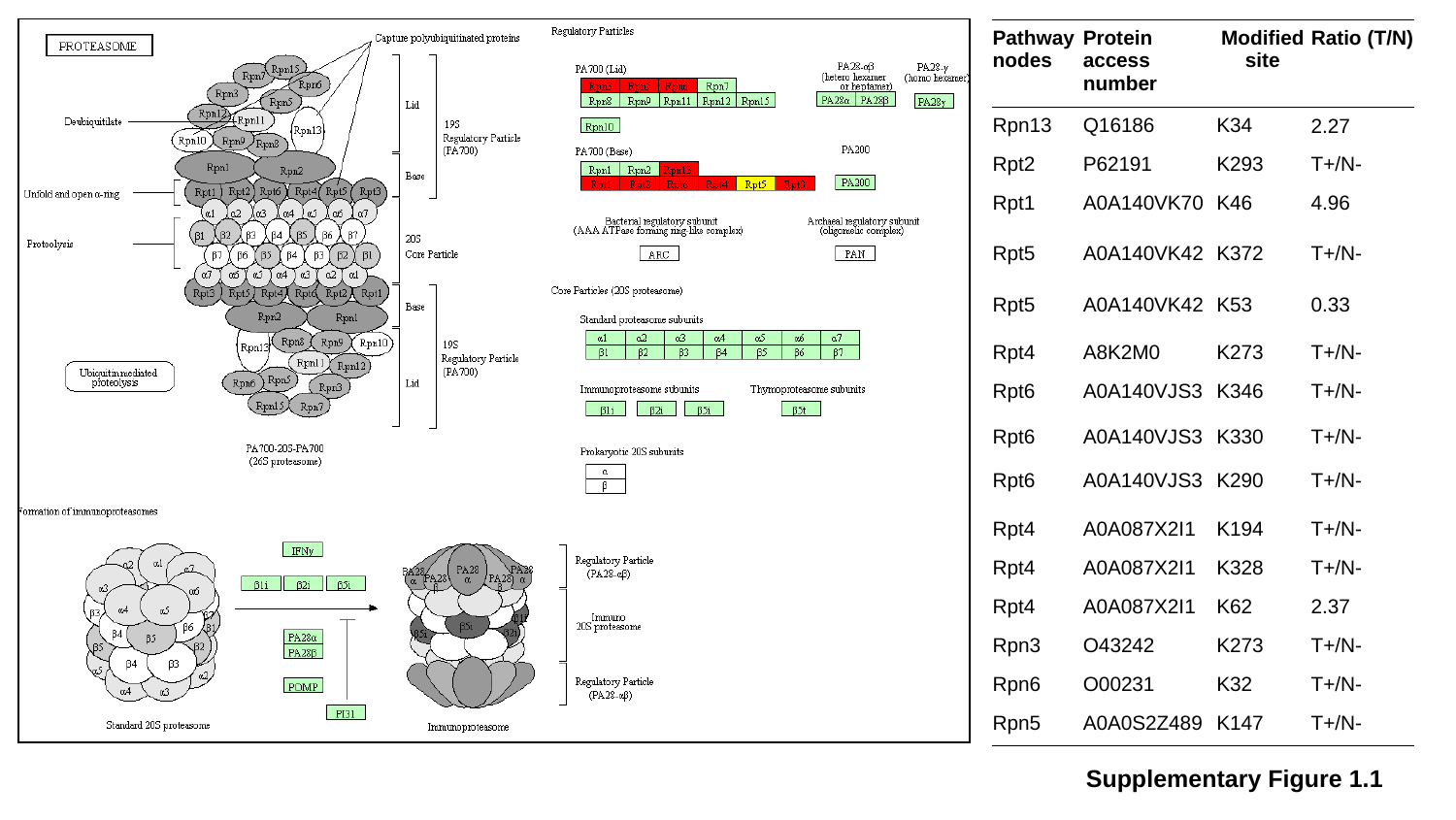

| Pathway nodes | Protein access number | Modified site | Ratio (T/N) |
| --- | --- | --- | --- |
| Rpn13 | Q16186 | K34 | 2.27 |
| Rpt2 | P62191 | K293 | T+/N- |
| Rpt1 | A0A140VK70 | K46 | 4.96 |
| Rpt5 | A0A140VK42 | K372 | T+/N- |
| Rpt5 | A0A140VK42 | K53 | 0.33 |
| Rpt4 | A8K2M0 | K273 | T+/N- |
| Rpt6 | A0A140VJS3 | K346 | T+/N- |
| Rpt6 | A0A140VJS3 | K330 | T+/N- |
| Rpt6 | A0A140VJS3 | K290 | T+/N- |
| Rpt4 | A0A087X2I1 | K194 | T+/N- |
| Rpt4 | A0A087X2I1 | K328 | T+/N- |
| Rpt4 | A0A087X2I1 | K62 | 2.37 |
| Rpn3 | O43242 | K273 | T+/N- |
| Rpn6 | O00231 | K32 | T+/N- |
| Rpn5 | A0A0S2Z489 | K147 | T+/N- |
Supplementary Figure 1.1

## Slide 4
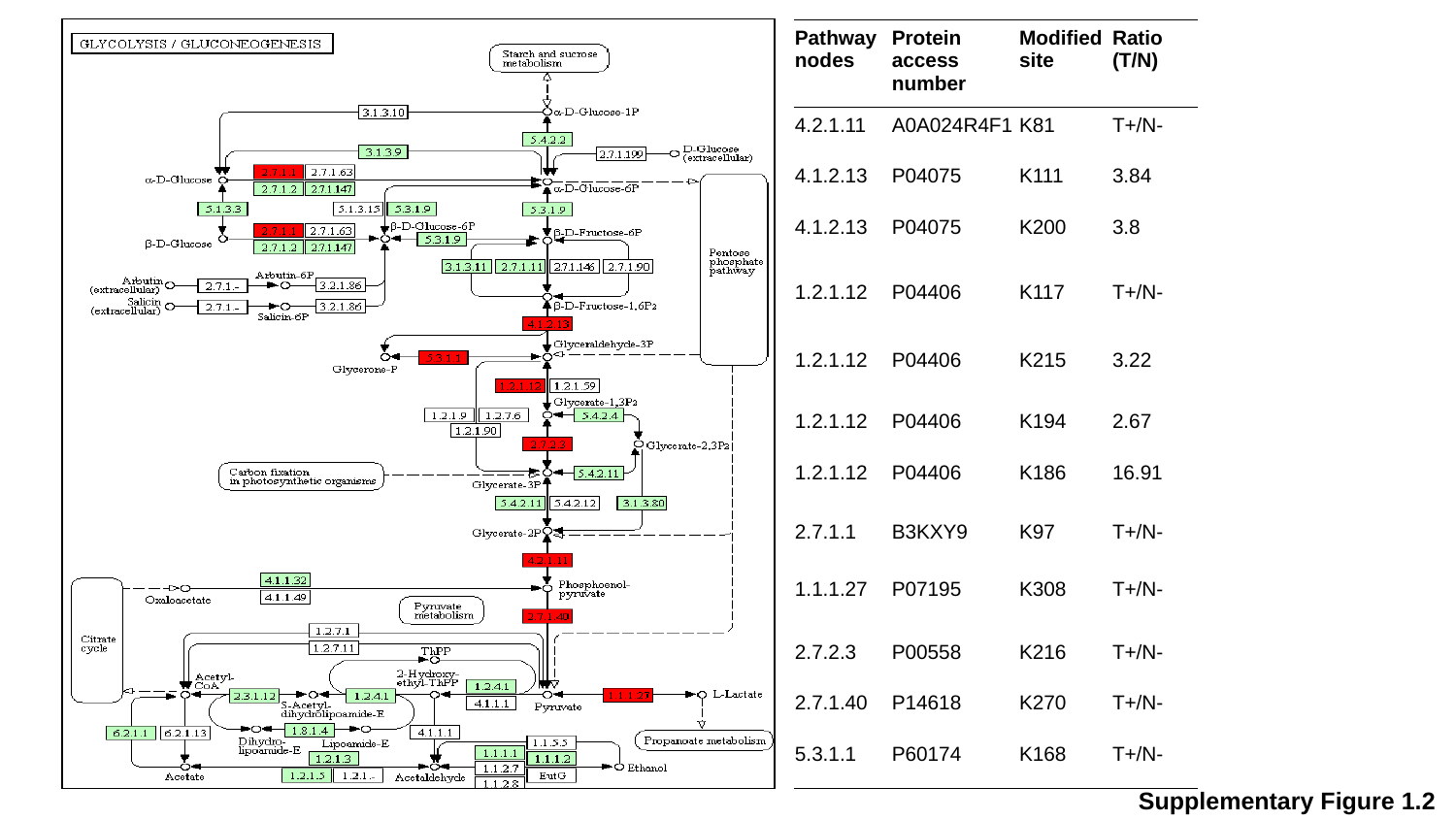

| Pathway nodes | Protein access number | Modified site | Ratio (T/N) |
| --- | --- | --- | --- |
| 4.2.1.11 | A0A024R4F1 | K81 | T+/N- |
| 4.1.2.13 | P04075 | K111 | 3.84 |
| 4.1.2.13 | P04075 | K200 | 3.8 |
| 1.2.1.12 | P04406 | K117 | T+/N- |
| 1.2.1.12 | P04406 | K215 | 3.22 |
| 1.2.1.12 | P04406 | K194 | 2.67 |
| 1.2.1.12 | P04406 | K186 | 16.91 |
| 2.7.1.1 | B3KXY9 | K97 | T+/N- |
| 1.1.1.27 | P07195 | K308 | T+/N- |
| 2.7.2.3 | P00558 | K216 | T+/N- |
| 2.7.1.40 | P14618 | K270 | T+/N- |
| 5.3.1.1 | P60174 | K168 | T+/N- |
Supplementary Figure 1.2

## Slide 5
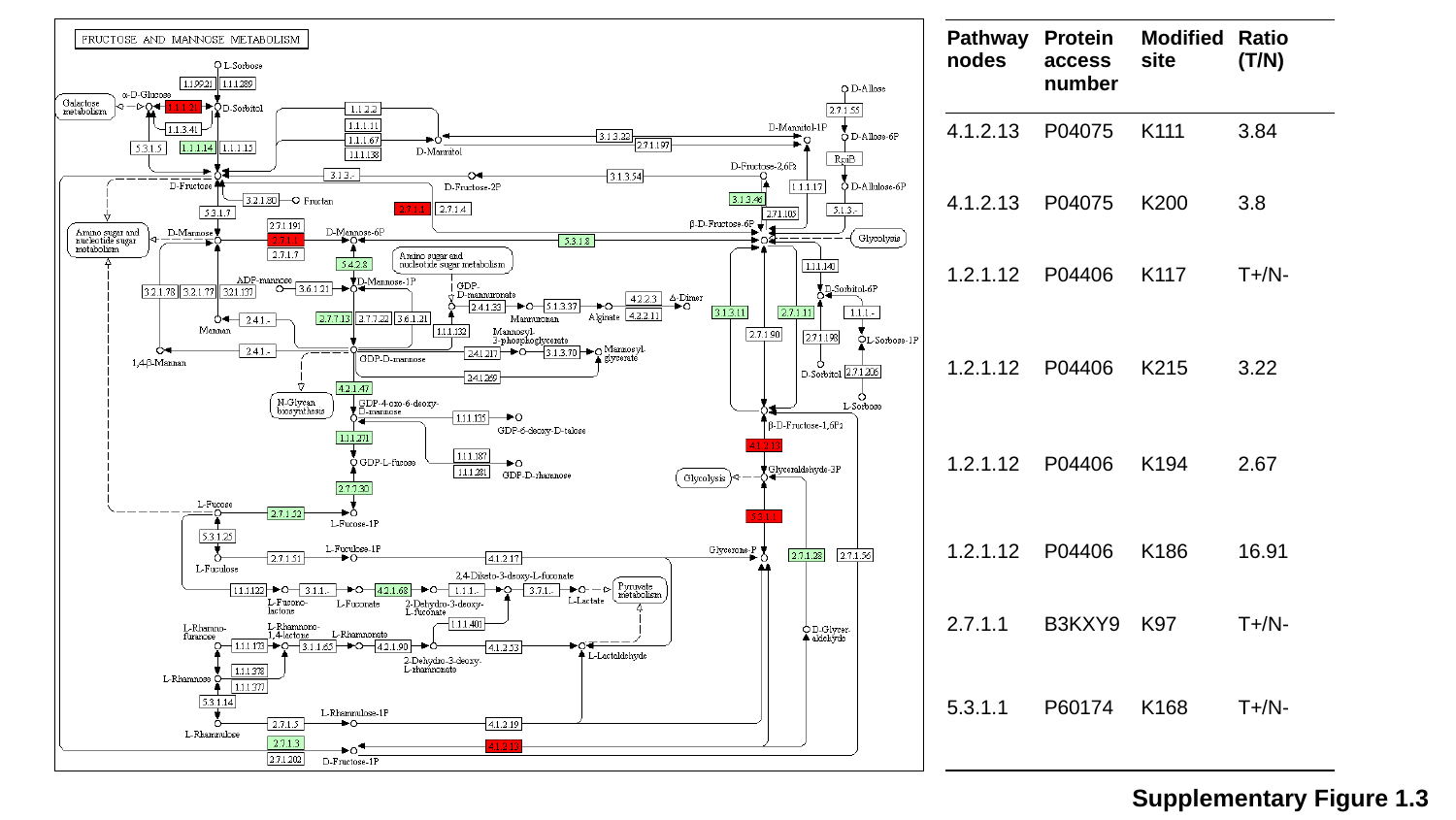

| Pathway nodes | Protein access number | Modified site | Ratio (T/N) |
| --- | --- | --- | --- |
| 4.1.2.13 | P04075 | K111 | 3.84 |
| 4.1.2.13 | P04075 | K200 | 3.8 |
| 1.2.1.12 | P04406 | K117 | T+/N- |
| 1.2.1.12 | P04406 | K215 | 3.22 |
| 1.2.1.12 | P04406 | K194 | 2.67 |
| 1.2.1.12 | P04406 | K186 | 16.91 |
| 2.7.1.1 | B3KXY9 | K97 | T+/N- |
| 5.3.1.1 | P60174 | K168 | T+/N- |
Supplementary Figure 1.3

## Slide 6
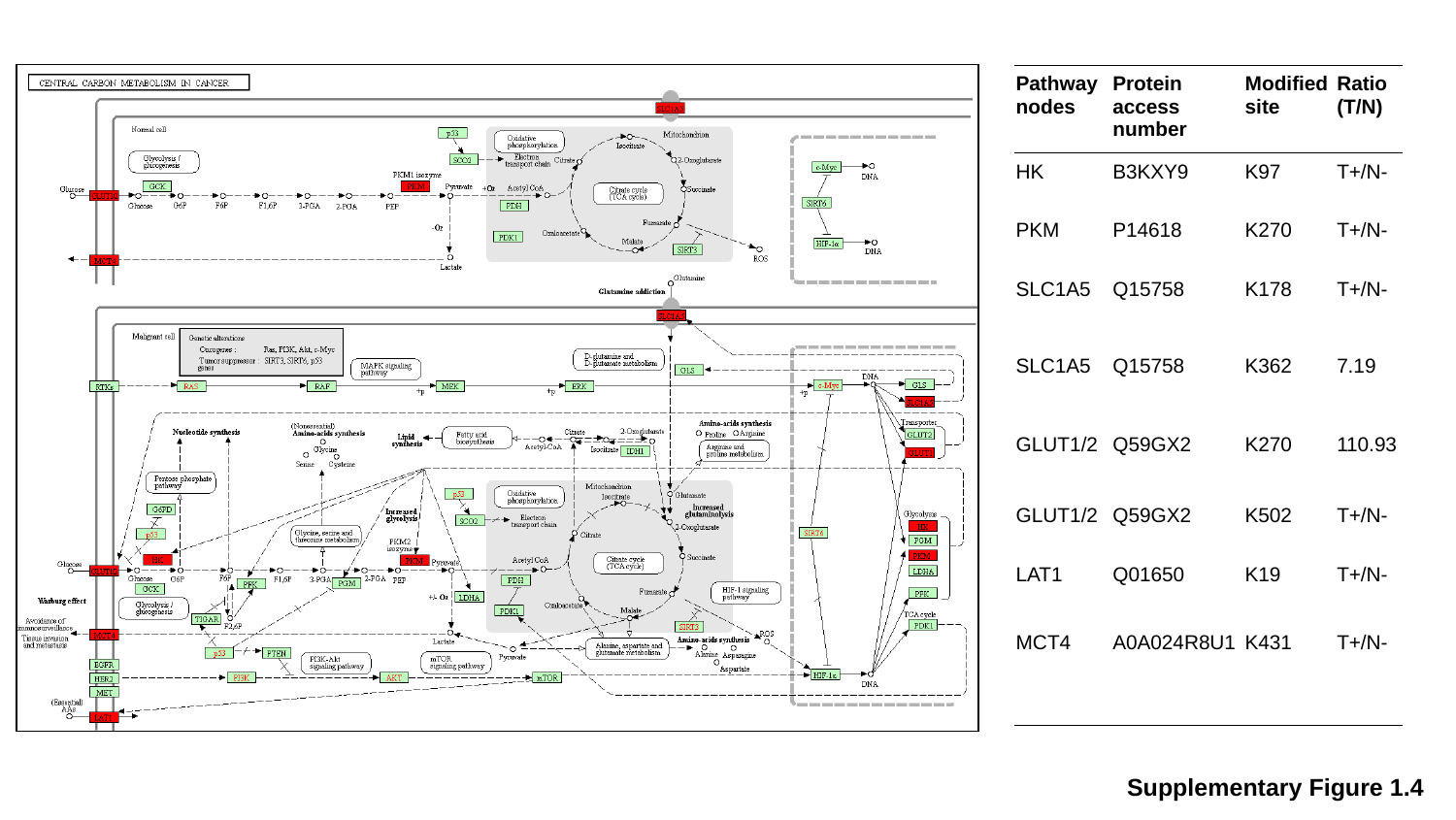

| Pathway nodes | Protein access number | Modified site | Ratio (T/N) |
| --- | --- | --- | --- |
| HK | B3KXY9 | K97 | T+/N- |
| PKM | P14618 | K270 | T+/N- |
| SLC1A5 | Q15758 | K178 | T+/N- |
| SLC1A5 | Q15758 | K362 | 7.19 |
| GLUT1/2 | Q59GX2 | K270 | 110.93 |
| GLUT1/2 | Q59GX2 | K502 | T+/N- |
| LAT1 | Q01650 | K19 | T+/N- |
| MCT4 | A0A024R8U1 | K431 | T+/N- |
Supplementary Figure 1.4

## Slide 7
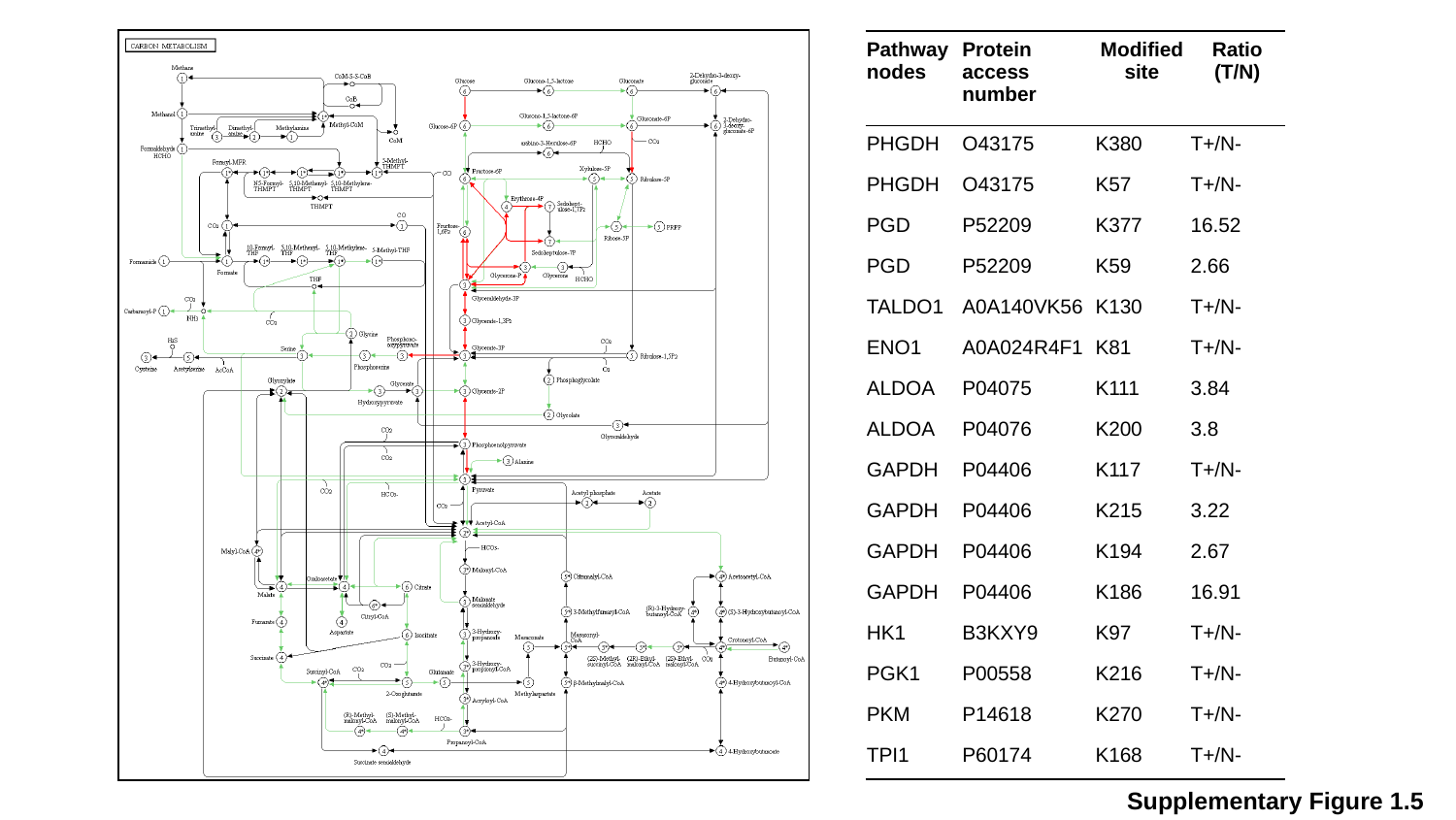

| Pathway nodes | Protein access number | Modified site | Ratio (T/N) |
| --- | --- | --- | --- |
| PHGDH | O43175 | K380 | T+/N- |
| PHGDH | O43175 | K57 | T+/N- |
| PGD | P52209 | K377 | 16.52 |
| PGD | P52209 | K59 | 2.66 |
| TALDO1 | A0A140VK56 | K130 | T+/N- |
| ENO1 | A0A024R4F1 | K81 | T+/N- |
| ALDOA | P04075 | K111 | 3.84 |
| ALDOA | P04076 | K200 | 3.8 |
| GAPDH | P04406 | K117 | T+/N- |
| GAPDH | P04406 | K215 | 3.22 |
| GAPDH | P04406 | K194 | 2.67 |
| GAPDH | P04406 | K186 | 16.91 |
| HK1 | B3KXY9 | K97 | T+/N- |
| PGK1 | P00558 | K216 | T+/N- |
| PKM | P14618 | K270 | T+/N- |
| TPI1 | P60174 | K168 | T+/N- |
Supplementary Figure 1.5

## Slide 8
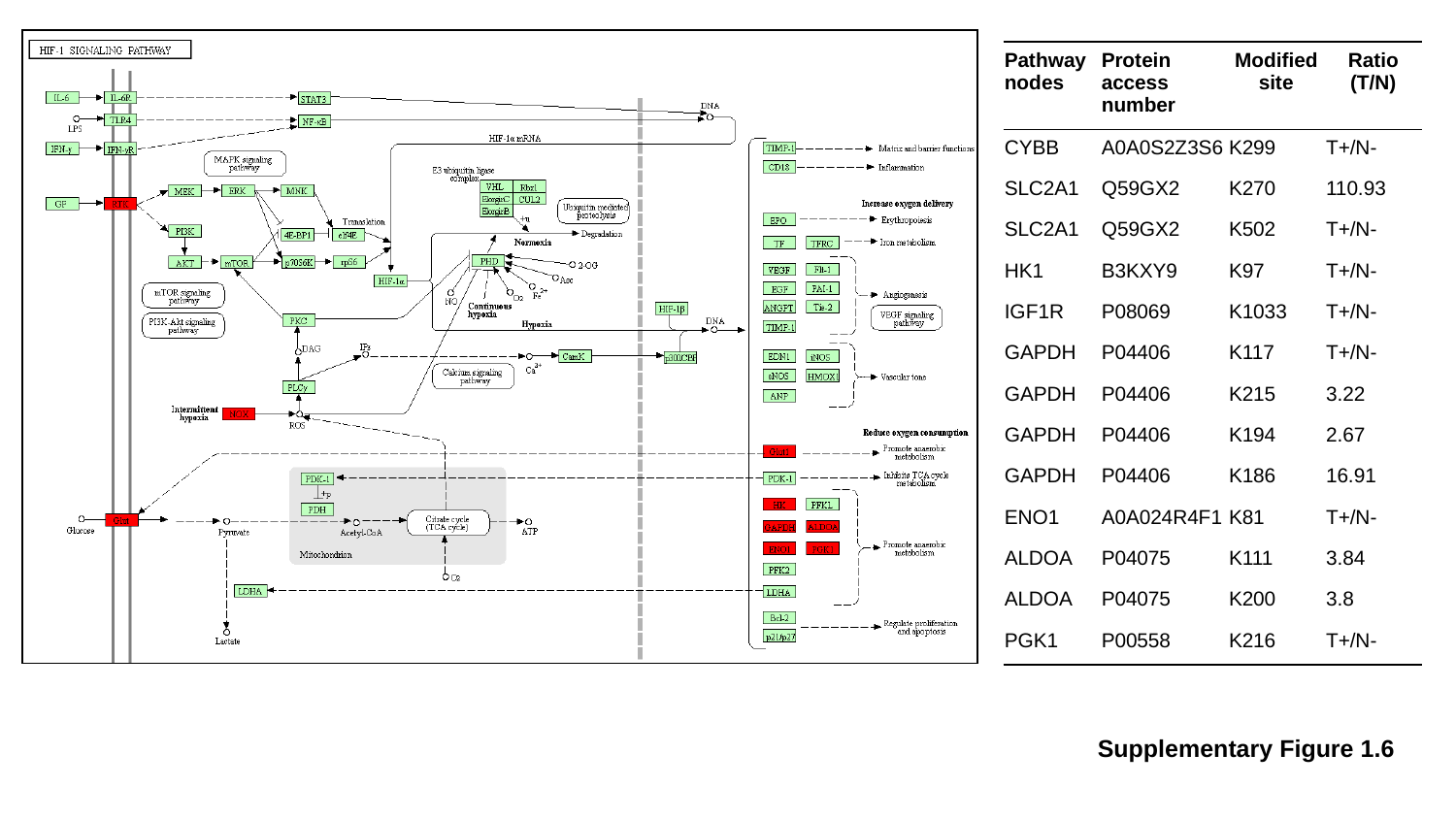

| Pathway nodes | Protein access number | Modified site | Ratio (T/N) |
| --- | --- | --- | --- |
| CYBB | A0A0S2Z3S6 | K299 | T+/N- |
| SLC2A1 | Q59GX2 | K270 | 110.93 |
| SLC2A1 | Q59GX2 | K502 | T+/N- |
| HK1 | B3KXY9 | K97 | T+/N- |
| IGF1R | P08069 | K1033 | T+/N- |
| GAPDH | P04406 | K117 | T+/N- |
| GAPDH | P04406 | K215 | 3.22 |
| GAPDH | P04406 | K194 | 2.67 |
| GAPDH | P04406 | K186 | 16.91 |
| ENO1 | A0A024R4F1 | K81 | T+/N- |
| ALDOA | P04075 | K111 | 3.84 |
| ALDOA | P04075 | K200 | 3.8 |
| PGK1 | P00558 | K216 | T+/N- |
Supplementary Figure 1.6

## Slide 9
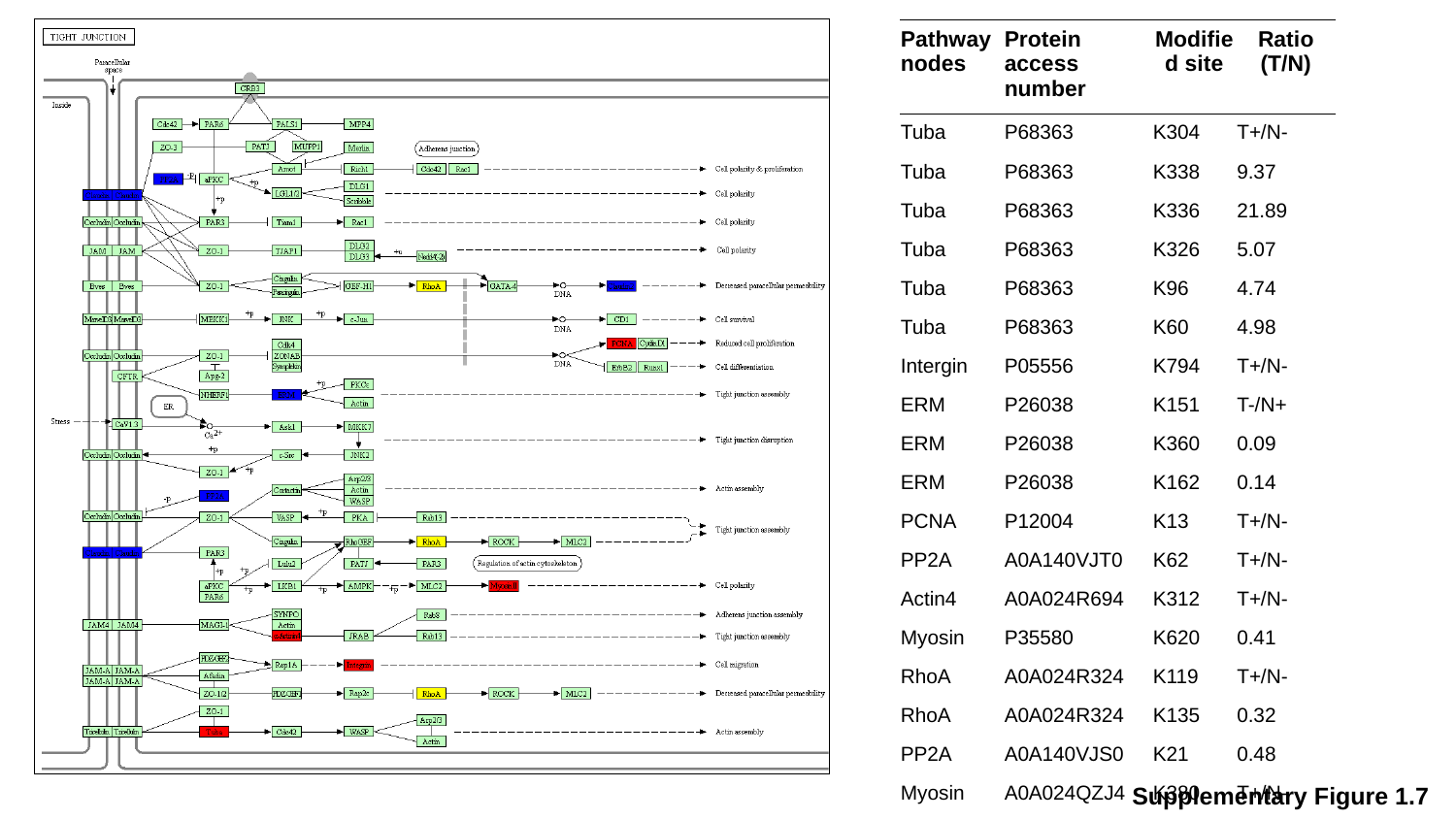

| Pathway nodes | Protein access number | Modified site | Ratio (T/N) |
| --- | --- | --- | --- |
| Tuba | P68363 | K304 | T+/N- |
| Tuba | P68363 | K338 | 9.37 |
| Tuba | P68363 | K336 | 21.89 |
| Tuba | P68363 | K326 | 5.07 |
| Tuba | P68363 | K96 | 4.74 |
| Tuba | P68363 | K60 | 4.98 |
| Intergin | P05556 | K794 | T+/N- |
| ERM | P26038 | K151 | T-/N+ |
| ERM | P26038 | K360 | 0.09 |
| ERM | P26038 | K162 | 0.14 |
| PCNA | P12004 | K13 | T+/N- |
| PP2A | A0A140VJT0 | K62 | T+/N- |
| Actin4 | A0A024R694 | K312 | T+/N- |
| Myosin | P35580 | K620 | 0.41 |
| RhoA | A0A024R324 | K119 | T+/N- |
| RhoA | A0A024R324 | K135 | 0.32 |
| PP2A | A0A140VJS0 | K21 | 0.48 |
| Myosin | A0A024QZJ4 | K380 | T+/N- |
| Claudin | P56856 | K239 | T-/N+ |
| Claudin | P56856 | K257 | 0.05 |
| Myosin | A0A024R1N1 | K679 | T+/N- |
| Myosin | A0A024R1N1 | K1410 | T+/N- |
| Myosin | A0A024R1N1 | K972 | 5.4 |
Supplementary Figure 1.7

## Slide 10
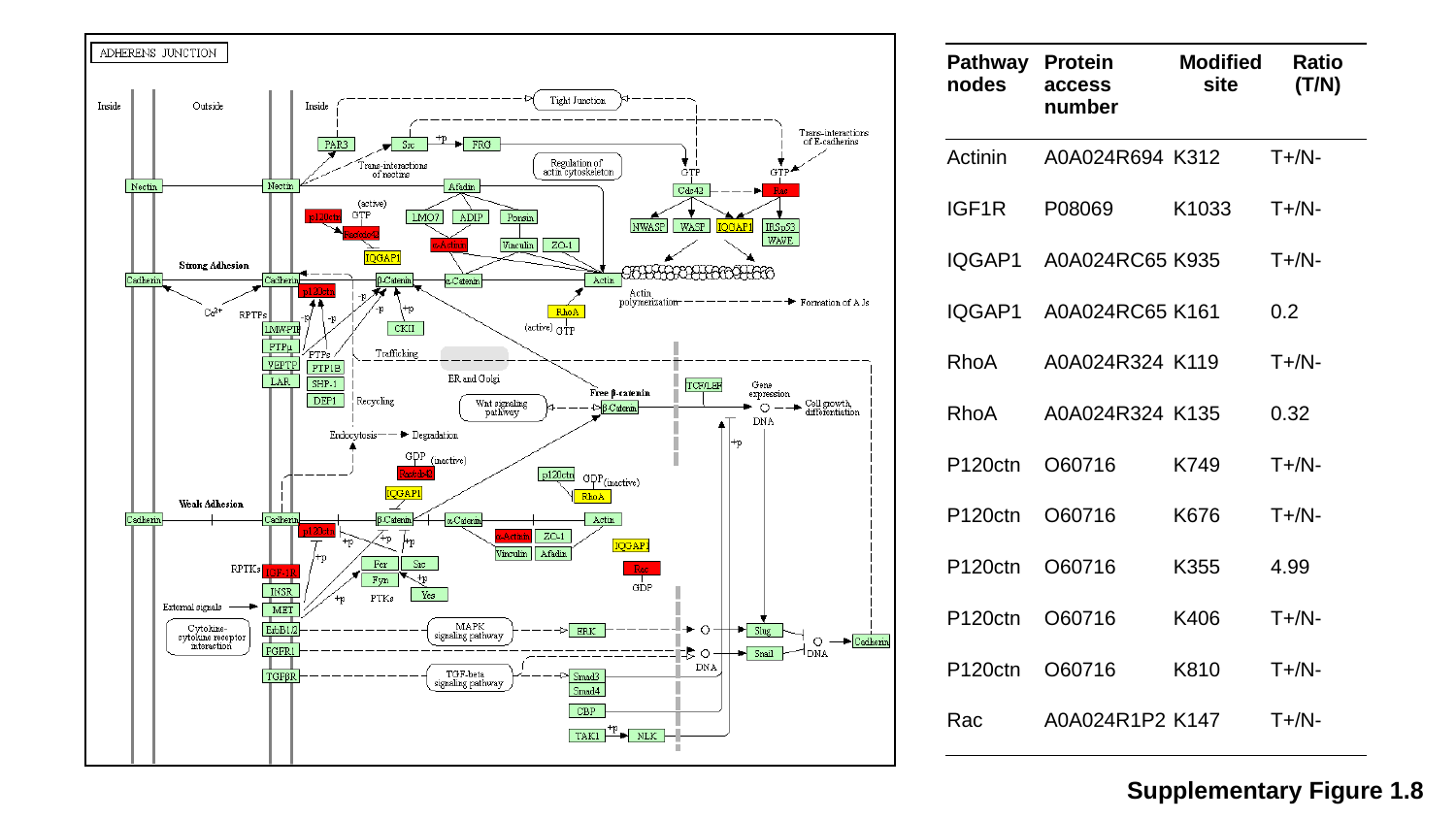

| Pathway nodes | Protein access number | Modified site | Ratio (T/N) |
| --- | --- | --- | --- |
| Actinin | A0A024R694 | K312 | T+/N- |
| IGF1R | P08069 | K1033 | T+/N- |
| IQGAP1 | A0A024RC65 | K935 | T+/N- |
| IQGAP1 | A0A024RC65 | K161 | 0.2 |
| RhoA | A0A024R324 | K119 | T+/N- |
| RhoA | A0A024R324 | K135 | 0.32 |
| P120ctn | O60716 | K749 | T+/N- |
| P120ctn | O60716 | K676 | T+/N- |
| P120ctn | O60716 | K355 | 4.99 |
| P120ctn | O60716 | K406 | T+/N- |
| P120ctn | O60716 | K810 | T+/N- |
| Rac | A0A024R1P2 | K147 | T+/N- |
Supplementary Figure 1.8

## Slide 11
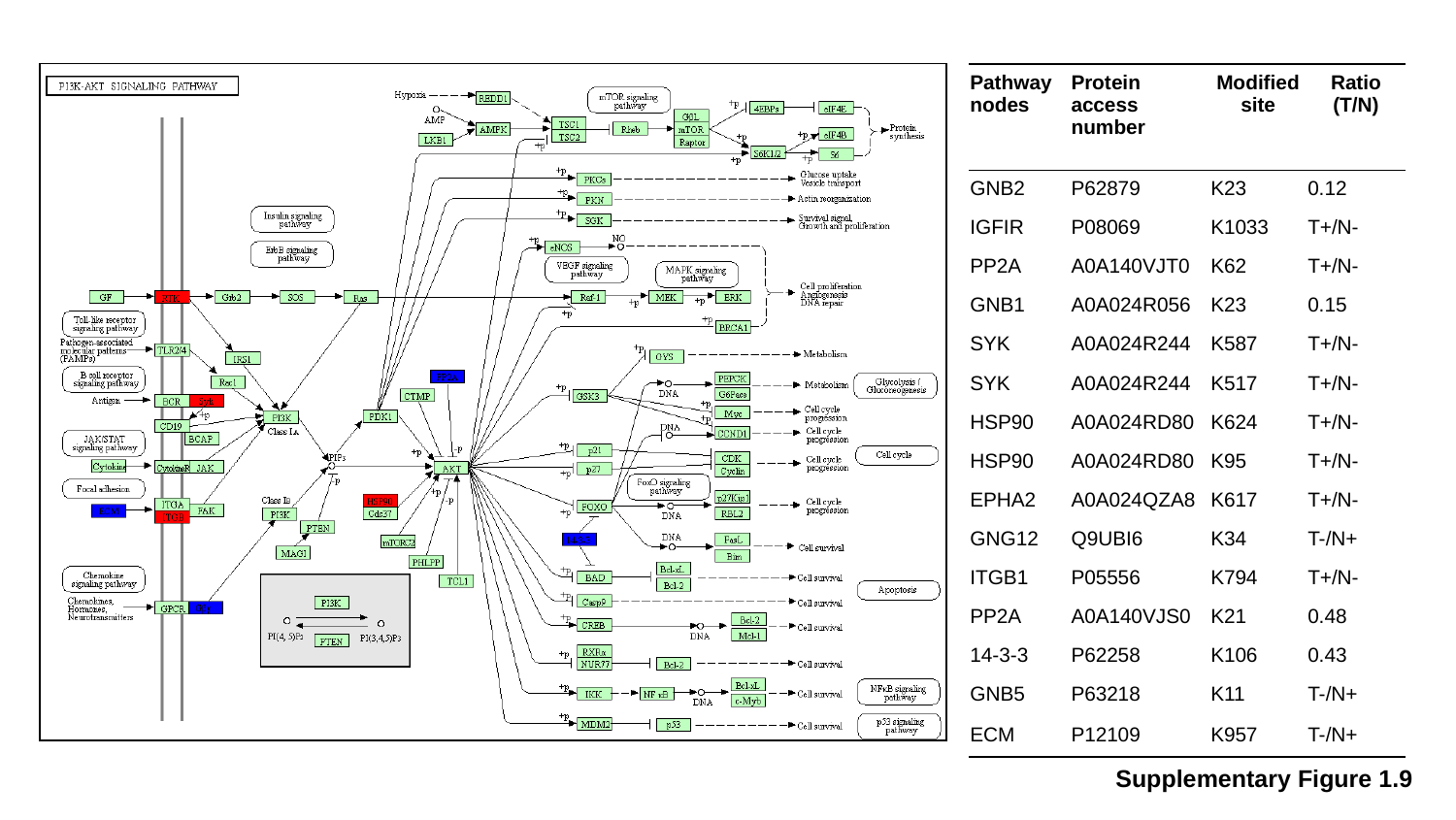

| Pathway nodes | Protein access number | Modified site | Ratio (T/N) |
| --- | --- | --- | --- |
| GNB2 | P62879 | K23 | 0.12 |
| IGFIR | P08069 | K1033 | T+/N- |
| PP2A | A0A140VJT0 | K62 | T+/N- |
| GNB1 | A0A024R056 | K23 | 0.15 |
| SYK | A0A024R244 | K587 | T+/N- |
| SYK | A0A024R244 | K517 | T+/N- |
| HSP90 | A0A024RD80 | K624 | T+/N- |
| HSP90 | A0A024RD80 | K95 | T+/N- |
| EPHA2 | A0A024QZA8 | K617 | T+/N- |
| GNG12 | Q9UBI6 | K34 | T-/N+ |
| ITGB1 | P05556 | K794 | T+/N- |
| PP2A | A0A140VJS0 | K21 | 0.48 |
| 14-3-3 | P62258 | K106 | 0.43 |
| GNB5 | P63218 | K11 | T-/N+ |
| ECM | P12109 | K957 | T-/N+ |
Supplementary Figure 1.9

## Slide 12
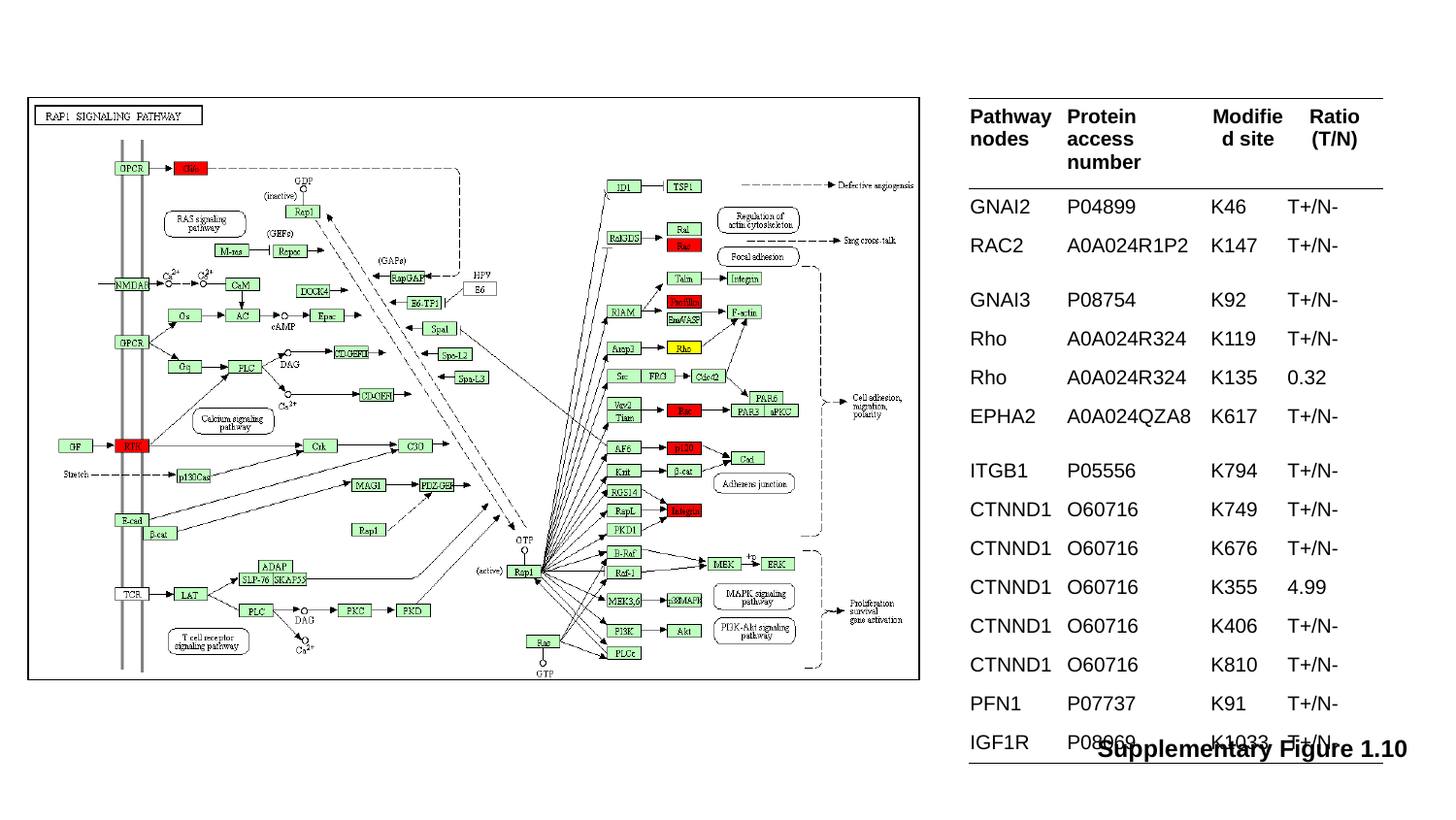

| Pathway nodes | Protein access number | Modified site | Ratio (T/N) |
| --- | --- | --- | --- |
| GNAI2 | P04899 | K46 | T+/N- |
| RAC2 | A0A024R1P2 | K147 | T+/N- |
| GNAI3 | P08754 | K92 | T+/N- |
| Rho | A0A024R324 | K119 | T+/N- |
| Rho | A0A024R324 | K135 | 0.32 |
| EPHA2 | A0A024QZA8 | K617 | T+/N- |
| ITGB1 | P05556 | K794 | T+/N- |
| CTNND1 | O60716 | K749 | T+/N- |
| CTNND1 | O60716 | K676 | T+/N- |
| CTNND1 | O60716 | K355 | 4.99 |
| CTNND1 | O60716 | K406 | T+/N- |
| CTNND1 | O60716 | K810 | T+/N- |
| PFN1 | P07737 | K91 | T+/N- |
| IGF1R | P08069 | K1033 | T+/N- |
Supplementary Figure 1.10

## Slide 13
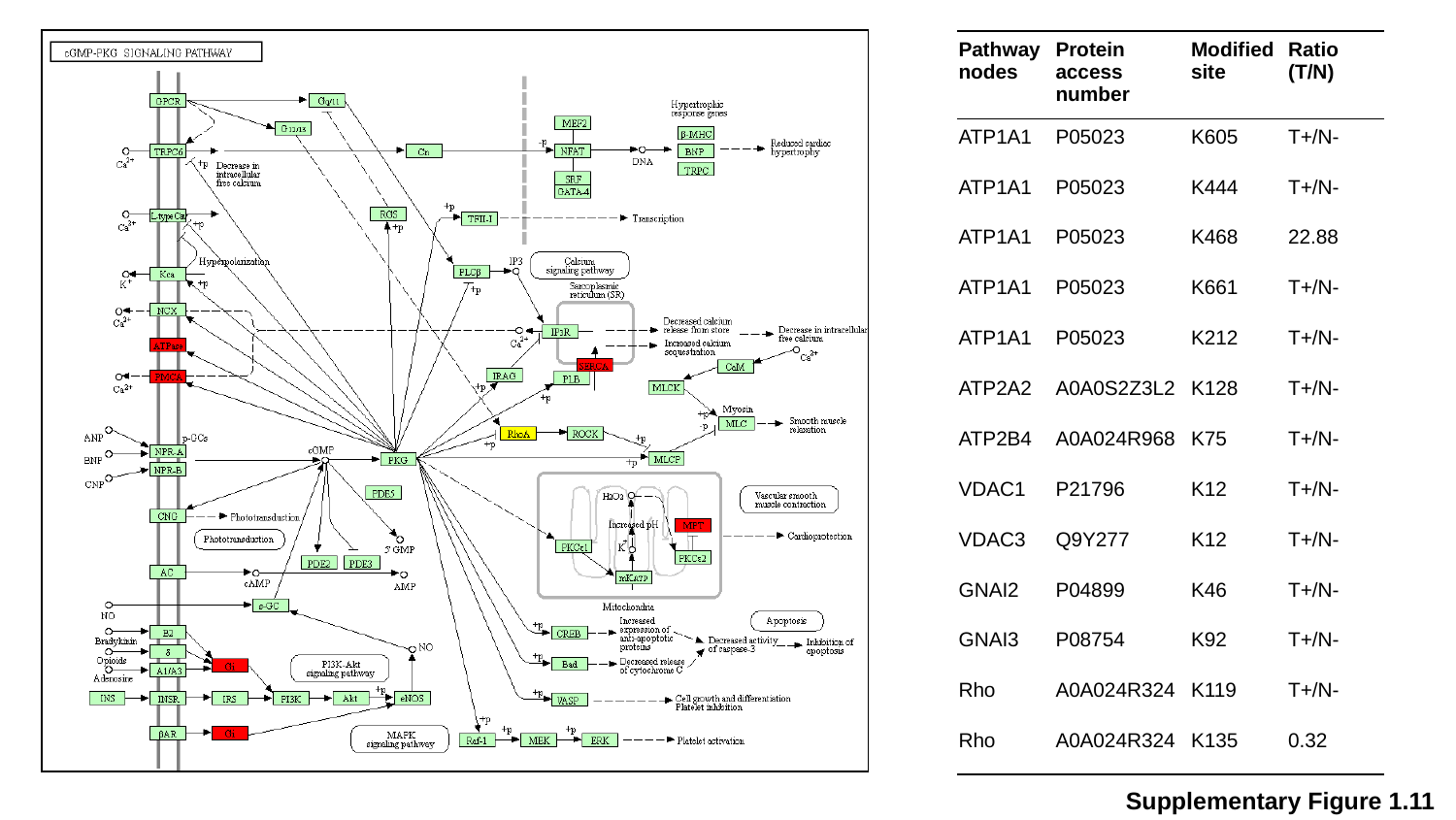

| Pathway nodes | Protein access number | Modified site | Ratio (T/N) |
| --- | --- | --- | --- |
| ATP1A1 | P05023 | K605 | T+/N- |
| ATP1A1 | P05023 | K444 | T+/N- |
| ATP1A1 | P05023 | K468 | 22.88 |
| ATP1A1 | P05023 | K661 | T+/N- |
| ATP1A1 | P05023 | K212 | T+/N- |
| ATP2A2 | A0A0S2Z3L2 | K128 | T+/N- |
| ATP2B4 | A0A024R968 | K75 | T+/N- |
| VDAC1 | P21796 | K12 | T+/N- |
| VDAC3 | Q9Y277 | K12 | T+/N- |
| GNAI2 | P04899 | K46 | T+/N- |
| GNAI3 | P08754 | K92 | T+/N- |
| Rho | A0A024R324 | K119 | T+/N- |
| Rho | A0A024R324 | K135 | 0.32 |
Supplementary Figure 1.11
