# Supplementary material for: Label-free quantitative identification of abnormally ubiquitinated proteins as useful biomarkers for human lung squamous cell carcinomas
Source: EPMA J. 2020 Jan 4;11(1):73–94. doi: 10.1007/s13167-019-00197-8 (PMC7028901; doi:10.1007/s13167-019-00197-8)
Supplement: Supplementary file 2 — (PPT 844 kb) [file 13167_2019_197_MOESM2_ESM.ppt]

## Slide 1
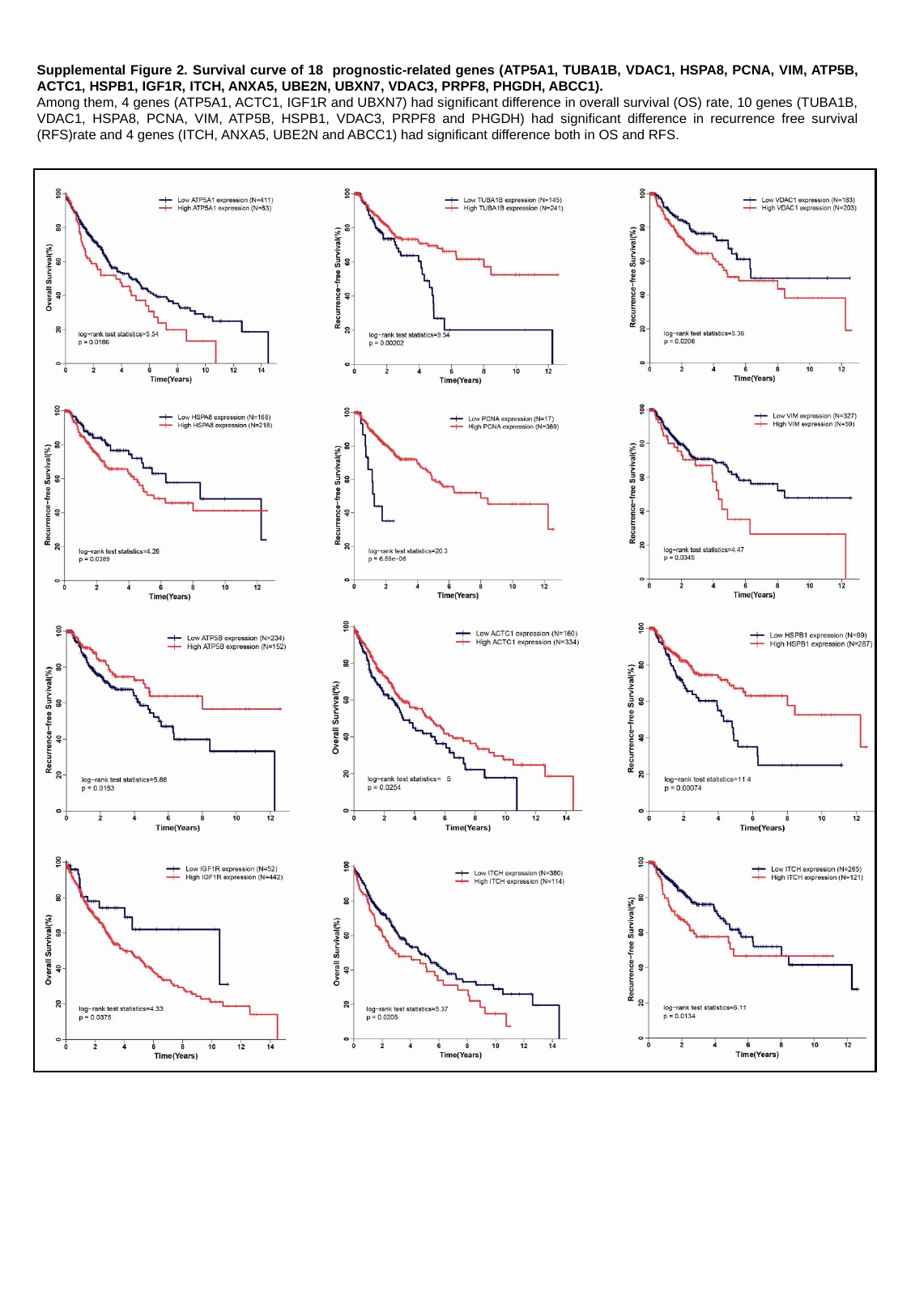

Supplemental Figure 2. Survival curve of 18 prognostic-related genes (ATP5A1, TUBA1B, VDAC1, HSPA8, PCNA, VIM, ATP5B, ACTC1, HSPB1, IGF1R, ITCH, ANXA5, UBE2N, UBXN7, VDAC3, PRPF8, PHGDH, ABCC1).
Among them, 4 genes (ATP5A1, ACTC1, IGF1R and UBXN7) had significant difference in overall survival (OS) rate, 10 genes (TUBA1B, VDAC1, HSPA8, PCNA, VIM, ATP5B, HSPB1, VDAC3, PRPF8 and PHGDH) had significant difference in recurrence free survival (RFS)rate and 4 genes (ITCH, ANXA5, UBE2N and ABCC1) had significant difference both in OS and RFS.

## Slide 2
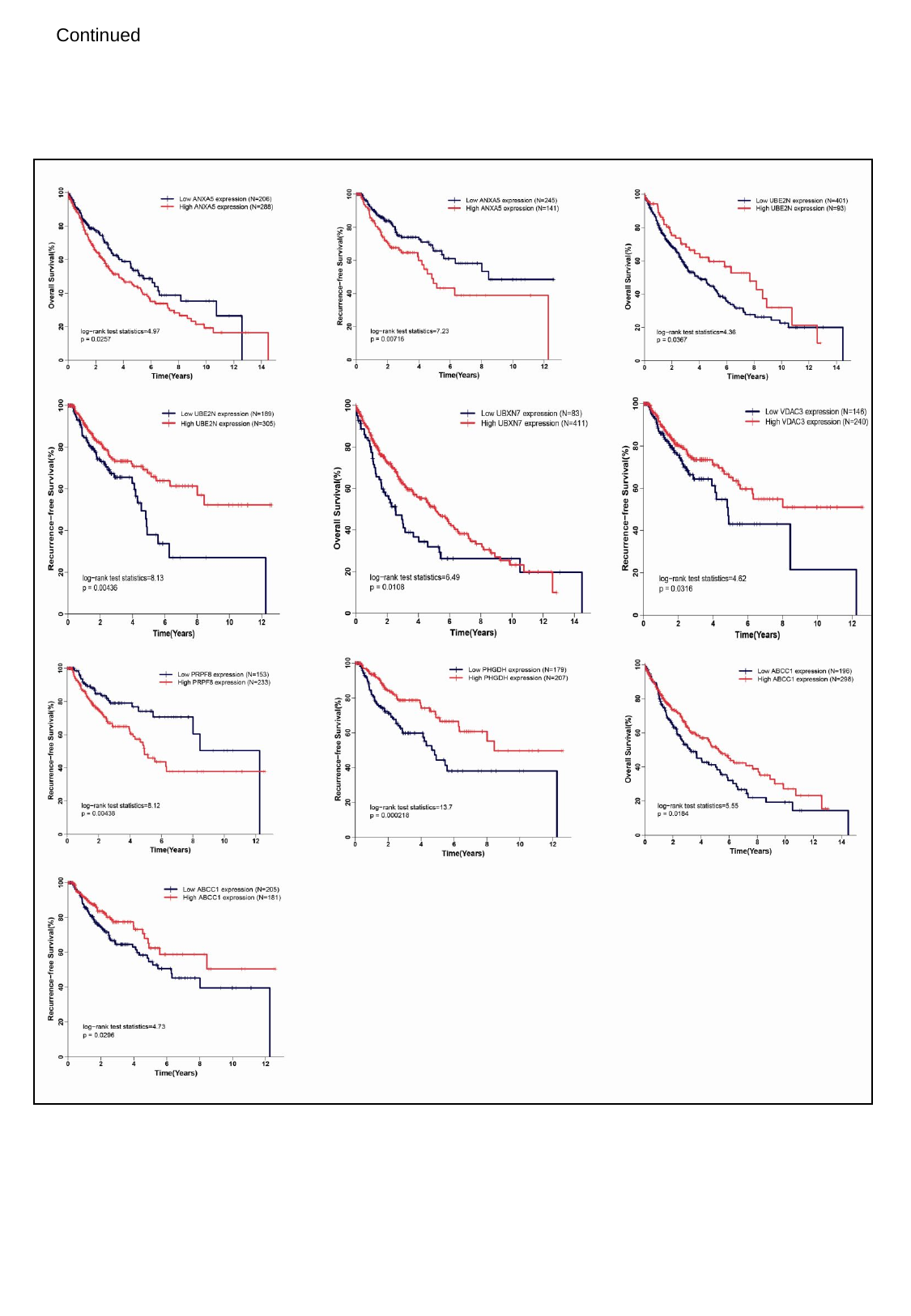

Continued
