# Supplementary material for: Label-free quantitative identification of abnormally ubiquitinated proteins as useful biomarkers for human lung squamous cell carcinomas
Source: EPMA J. 2020 Jan 4;11(1):73–94. doi: 10.1007/s13167-019-00197-8 (PMC7028901; doi:10.1007/s13167-019-00197-8)
Supplement: Supplementary file 3 — (PDF 97 kb) [file 13167_2019_197_MOESM3_ESM.pdf]

Supplemental Table 1. Ubiquitinated proteins identified in LSCCs relative to controls. Ratio (T/N) =Ration of tumors(T) to controls(N). K* = Ubiquitinated lysine residue.

Accession No.	Gene name	Protein name	Modifided peptides	Modifided positions	Modifided probabilitie	Modified level (T)	Modified level (N)	Ratio (T/N)	t test p value
A0A024QZA8	EPHA2	EPH receptor A2, isoform CRA_a	QK*VIGAGEFGEVYK	617	1	3130600			
A0A024QZJ4	MYH11	Myosin, heavy polypeptide 11, smooth muscle, isoform CRA_a	LFTK*VKPLLQVTR	840	1	20753333			
A0A024QZN9	VDAC2	Voltage-dependent anion channel 2, isoform CRA_a	LTFDTTFSPNTGKK*	146	0.536	2601933			
A0A024QZP6	H2AFY2	Core histone macro-H2A	GK*SETILSPPPEKR	123	1	13477667			
A0A024R056	GNB1	Guanine nucleotide binding protein (G protein), beta polypeptide 1, isoform CRA_a	K*ACADATLSQITNNIDPVGR	23	1	3532333	23399000	0.15	7.85E-04
A0A024R0H1	SLC16A1	Solute carrier family 16 (Monocarboxylic acid transporters), member 1, isoform CRA_b	EEETSIDVAGKPNEVTK*AAESPDQK	479	0.999	5299400			
A0A024R0S6	EHD2	EH-domain containing 2, isoform CRA_a	TVTSALK*ELYR	26	1	3048250	64938667	0.05	3.93E-05
A0A024R0Z5	TEGT	Testis enhanced gene transcript (BAX inhibitor 1), isoform CRA_a	K*INFDALLK	7	1	21429000	2414167	8.88	3.43E-02
A0A024R1N1	MYH9	Myosin, heavy polypeptide 9, non-muscle, isoform CRA_a	K*AGKLDPHLVLDQLR	679	0.69	13710333			
			VAAYDKLEKTK*	1413	0.875	11818400			
			VK*PLLQVSR	835	1	16890667	3130050	5.40	3.40E-03

A0A024R1N4	XRCC6	X-ray repair complementing defective repair in Chinese hamster cells 6 (Ku autoantigen, 70kDa), isoform CRA_a	TFNTSTGGLLLPSDTK*R	317	1	4542650			
A0A024R1P2	RAC2	Ras-related C3 botulinum toxin substrate 2 (Rho family, small GTP binding protein Rac2), isoform CRA_a	LAPITYPQGLALAK*EIDSVK	147	0.944	2985667			
A0A024R1T2	KRTHA5	Keratin, hair, acidic, 5, isoform CRA_a	TIEELQK*	156	1	239730000			
A0A024R1X8	JUP	Junction plakoglobin, isoform CRA_a	LLNQPNQWPLVK*ATIGLIR	499	1	3576867			
			NLSDVATK*QEGLESVLK	385	1	3988100			
A0A024R1Z6	VAT1	Vesicle amine transport protein 1 homolog (T californica), isoform CRA_a	ISPK*GVDIVMDPLGGSDTA K	255	1	25786667	60146333	0.43	3.79E-03
A0A024R210	IFITM1	Interferon induced transmembrane protein 1 (9-27), isoform CRA_a	MHK*EEHEVAVLGAPPSTIL PR	3	1	40647000	4662833	8.72	1.48E-03
A0A024R244	SYK	Tyrosine-protein kinase	GSEVTAMLEK*GER	587	1	4398400			
			ISDFGLSK*ALR	517	1	8890667			
A0A024R2M8	XPC	Xeroderma pigmentosum, complementation group C, isoform CRA_a	SLLPVK*PVEIEIETPEQAK	161	1		3078000		
A0A024R324	RHOA	Ras homolog gene family, member A, isoform CRA_a	HFCPNVPIILVGNKK*	119	0.9	8406367			
			MK*QEPVKPEEGR	135	1	4368350	13454600	0.32	4.59E-02

A0A024R3E3	APOA1	Apolipoprotein A-I, isoform CRA_a	DYVSQFEGSALGK*QLNLK	64	1	3406267	88441333	0.04	1.62E-03
			VQPYLDDFQKK*	131	0.563		12304133		
			VSFLSALEEYTKK*	263	0.5		4042000		
			VSFLSALEEYTK*K	262	0.629		5436200		
A0A024R3V8	TSNAX	Translin-associated factor X, isoform CRA_c	TPSSDAQDK*QFGTWR	173	1	10708567			
A0A024R4E2	TARDBP	TAR DNA binding protein, isoform CRA_b	AVQK*TSDLIVLGLPWK	102	1	4941167			
			LPNSK*QSQDEPLR	181	1	5747050			
			WCDCK*LPNSK	176	1	2462500			
A0A024R4F1	ENO1	Enolase 1, (Alpha), isoform CRA_a	K*LNVTEQEK	81	1	3367000			
A0A024R4S1	EPN1	Epsin 1, isoform CRA_a	ENMYAVQTLK*DFQYVDR	107	1	5460700	29727000	0.18	9.53E-05
A0A024R4Y2	hCG_39762	HCG39762, isoform CRA_c	EFLSAK*EETPGAGQK	255	1	3591833	10746633	0.33	2.14E-03
			ELQSK*EFLSAK	249	1		13918033		
A0A024R539	LOC51035	Uncharacterized protein	MLELVAQK*QR	105	1	31278667	7629233	4.10	1.12E-02
A0A024R546	ZDHHC5	Palmitoyltransferase	TVEIK*GIQVR	97	1	1833067			
A0A024R5Z7	ANXA2	Annexin	ASMK*GLGTDEDSLIEIICSR	119	1	4649550	33281333	0.14	4.89E-03

			DLYDAGVK*R	204	1	8685800	46219667	0.19	5.56E-03
			TK*GVDEVTIVNILTNR	49	1	18385667	43711000	0.42	1.31E-03
A0A024R694	ACTN1	Actinin, alpha 1, isoform CRA_a	VPENTMHAMQQK*LEDFR	312	1	5869100			
A0A024R6N1	TRAF3	TNF receptor-associated factor 3, isoform CRA_a	VTELESVDK*SAGQVAR	369	1	2203333			
A0A024R757	CAV1	Caveolin	ADELSEK*QVYDAHTK	8	1	40507000	275663333	0.15	3.32E-03
			QVYDAHTK*EIDLVNR	16	1	17464667	87394667	0.20	1.68E-02
A0A024R7C7	ILF3	Interleukin enhancer binding factor 3, 90kDa, isoform CRA_d	GWPLELLCEK*SIGTANRP MGAGEALR	257	1	6463233			
A0A024R7G8	RAD23A	RAD23 homolog A (S. cerevisiae), isoform CRA_a	EDK*SPSEESAPTTSPESV SGSVPSSGSSGR	122	1		13101500		
			LIYAGK*ILSDDVPIRDYR	53	1	119434000	261560000	0.46	6.28E-03
A0A024R7T3	HNRPF	Heterogeneous nuclear ribonucleoprotein F, isoform [illegible]	YIGIVK*QAGLER	224	1	15064600			
A0A024R816	TTYH3	Protein tweety homolog	AK*YLATSQPRPDSSGSH	508	1	10262267			
A0A024R870	LRSAM1	Leucine rich repeat and sterile alpha motif containing 1, isoform [illegible]	WALSSLLQQLLKEK*	522	0.646	2802300	242230	11.57	3.13E-03
A0A024R8B8	C9orf28	Chromosome 9 open reading frame 28, isoform CRA_a	ENSHLGNVLVDMK*LIDIK	115	0.999	3338433			
A0A024R8N2	ITGB4	Integrin beta	LCTENLLK*PDTR	834	1	16216333			

A0A024R8U1	SLC16A3	Solute carrier family 16 (Monocarboxylic acid transporters), member 3, isoform CRA_a	LHK*PPADSGVDLR	431	1	2378850			
A0A024R936	NCF2	Neutrophil cytosolic factor 2 (65kDa, chronic granulomatous disease, autosomal 2), isoform CRA_a	DLK*EALIQLR	95	1	7897267			
			QK*LYEPVVIPVGR	170	1	4964267			
A0A024R968	ATP2B4	Calcium-transporting ATPase	TSPVEGLSGNPADLEK*R	75	1	18497667			
A0A024R9M9	CHP	Calcium binding protein P22, isoform CRA_a	ASTLLRDEELEEIKK*	19	0.541	10311250			
A0A024R9W5	HUWE1	HECT, UBA and WWE domain containing 1, isoform CRA_b	SSLLTEK*LLR	3452	1	48685000	21275000	2.29	2.93E-03
A0A024R9Z8	AHR	Aryl hydrocarbon receptor, isoform CRA_a	HMQNEK*FFR	560	1	14323000			
			LASLLPFPQDVINKLDK*	66	0.534	4038300			
			LASLLPFPQDVINK*LDK	63	0.5	2493670			
			QVVVEPQQQLCQKMK*	643	0.54	2859100			
			QVVVEPQQQLCQK*MK	641	0.986	10786333			
A0A024RAM0	TNPO1	Transportin 1, isoform CRA_a	K*CSAAALDVLANVYR	385	1	1395467			

			SLSGLILKNNVK*	85	0.562	3631300			
A0A024RAM4	MAP1B	Microtubule-associated protein 1B, isoform CRA_b	DLSTPGLEK*DSGGK	1858	0.706	3974050			
			SPDEEDYDYESYEK*TTR	1894	1	4461733			
A0A024RAU0	OLR1	Oxidised low density lipoprotein (Lectin-like) receptor 1, isoform CRA_a	TFDDLK*IQTVK	7	1	6762533			
A0A024RAY2	KRT18	Keratin 18, isoform CRA_a	NLK*ASLENSLR	317	1	15345333	2478800	6.19	2.81E-02
A0A024RB16	FAM62A	Family with sequence similarity 62 (C2 domain containing), member A, isoform CRA_a	K*LVSIVHGCR	997	1	41265667			
A0A024RBE7	TMPO	Thymopoietin, isoform CRA_c	YVPK*YVPLADVK	393	1	3264300	15731000	0.21	7.34E-03
A0A024RBJ2	KCTD10	Potassium channel tetramerisation domain containing 10, isoform CRA_b	TTSFK*GTSPSSK	25	1	4609000	17874333	0.26	6.97E-03
A0A024RBS2	RPLP0	60S acidic ribosomal protein P0	DMLLANK*VPAAAR	106	1	7666700			
			GHLENNPALEK*LLPHIR	77	1	10692650			
A0A024RBT0	WDR66	WD repeat domain 66, isoform CRA_a	K*TLLGPAYGSPIEQTQVLPVR	837	1	7399500			
			TLSFSK*TPATPPTEK	556	1	1724300			
A0A024RC65	hCG_1991735	HCG1991735, isoform CRA_a	ITLQDVVSHSK*K	935	0.825	2188050			

			LFK*TALQEEIK	1027	1	7545933	37794667	0.20	1.24E-04
A0A024RC92	FES	Tyrosine-protein kinase	NCLVTEK*NVLK	694	0.956	4972600			
			TLLQMVGDAAAGMEYLESK*CCIHR	677	1	2125900			
A0A024RCJ2	HIST1H2BJ	Histone H2B	PEPAK*SAPAPK	6	1	13078700	33421000	0.39	2.84E-02
A0A024RCL1	DDR1	Discoidin domain receptor family, member 1, isoform [illegible]	ILRPDATK*NAR	663	1	616243333			
			K*GHPLLVAVK	646	1	##########	29927667	41.31	6.21E-03
A0A024RD80	HSP90AB1	Heat shock protein 90kDa alpha (Cytosolic), class B member 1, isoform CRA_a	K*HLEINPDHPIVETLR	624	1	2342633			
			TLTLVDTGIGMTK*ADLINNLGTIAK	95	0.995	2597700			
A0A024RDL1	CCT6A	Chaperonin containing TCP1, subunit 6A (Zeta 1), isoform [illegible]	IITEGFEAAKEK*	129	0.636	5513800			
			IITEGFEAAK*EK	127	0.777	3113850			
A0A024RDS4	GJB6	Gap junction protein	NDFK*DIEDIKK	116	1	7856333			
			NHPNHALK*ESK	237	1	11203000			
A0A024RDY4	TFDP1	Transcription factor Dp-1, isoform CRA_a	QK*QSQLQELILQQIAFK	219	1	2210267			
A0A024RE04	RP11-98F14.6	Uncharacterized protein	AIDSSNLK*DDYSTAQR	255	1	10792733			

			K*AMFDSDFK	273	1	6335533			
A0A059WJG5	HLA-DQB1	MHC class I antigen	SQK*GPQGPPPAGLLH	257	1		2032190		
A0A087WUF0	CYP4F3	Docosahexaenoic acid omega-hydroxylase CYP4F3	TLPSQGVDDFLQAKAK*	292	0.588	17476667			
A0A087WUW9	ARL15	ADP-ribosylation factor-like protein 15	K*YFELEPLAR	157	1	1473050			
Q59FP5		Spectrin, beta, erythrocytic (Includes spherocytosis, clinical type I) variant	IHCLENVDK*ALQFLK	122	1		5828450		
A0A087WVQ6	CLTC	Clathrin heavy chain	AHIAQLCEK*AGLLQR	623	1	7236700			
A0A087WYT3	PTGES3	Prostaglandin E synthase 3	DVNVNFEKSK*	39	0.725	7660100			
			DVNVNFEK*SK	37	0.977	9921750			
A0A087X018	SLC2A11	Solute carrier family 2, facilitated glucose transporter member 11	LSSQQNSSPK*	506	1	9515300	33305000	0.29	4.19E-02
A0A087X2I1	PSMC6	26S protease regulatory subunit 10B	GCLLYGPPGTGK*TLLAR	194	1	4502467			
			LDILK*IHAGPITK	328	0.999	15559000			
			SENDLK*ALQSVGQIVGEVLK	62	1	4433833	1867500	2.37	4.51E-03
A0A090N8Q3	C7orf21	Chromosome 7 open reading frame 21	AWPHDTIGSLK*R	129	1	5403200			
			LK*FLNDSEQVAR	108	1	21404667	7453867	2.87	1.78E-02

A0A0A0MRK6	MTX1	Metaxin 1, isoform CRA_b	VHK*ISNPWQSPSGTLPALR	190	1	2670300			
A0A0A6YYL4	CORO7-PAM16	Coronin	VPAEGLEEVLTTPETVLTGHTEK*ICSLR	596	1	10095300			
A0A0A6YZ17	USP13	Ubiquitin specific protease 13	GLQPGEEELPDISPPIVIPDDSK*DR	640	1	10412667	3270850	3.18	3.51E-03
A0A0A7KU15	TSC1-PDGFRB	TSC1-PDGFEB fusion protein (Fragment)	MAQPAHASDEIYEIMQK*CWEEK	1215	0.951	5626267	1943433	2.90	2.15E-03
A0A0C4DFL7	CYP51A1	Lanosterol 14-alpha demethylase	TVCGENLPPLTYDQLK*DLNLLDR	364	1	6333467			
A0A0D9SF60	PKP4	Plakophilin-4	SPSIDSIQK*DPR	517	1	12088300			
A0A0D9SGC1	MYO6	Unconventional myosin-VI	SLDSYPVTSK*NDGTRPK	1045	0.976		8417700		
A0A0G2JIW1	HSPA1B	Heat shock 70 kDa protein 1B	AMTK*DNNLLGR	452	1	49823667	15970000	3.12	4.22E-04
			K*FGDPVVQSDMK	78	1	1198150			
			LIGDAAK*NQVALNPQNTVFDAK	57	1	5952700	2585467	2.30	6.79E-04
			MVQEAEK*YKAEDEVQR	525	1	442610000	126953333	3.49	6.21E-04
			QATK*DAGVIAGLNVLR	160	1	7358333	1015630	7.25	4.69E-02
			RK*ELEQVCNPIISGLYQGAGGPGPGGFGAQGPK	598	1	36014000	4228233	8.52	3.59E-03
			VLDK*CQEVISWLDANTLAEKDEFEHK	574	1	8481900			
			YK*AEDEVQR	527	1	13685000	5775450	2.37	1.08E-02

A0A0S2Z2Z6	ANXA6	Annexin	AMK*GLGTDEDTIIDIITHR	377	1	6458700	22420333	0.29	4.77E-03
			K*AMKGLGTDEDTIIDIITHR	374	0.532	3266750	11707733	0.28	1.10E-02
			TNEQMHQLVAAYK*DAYER	135	1		1858500		
A0A0S2Z3F2	ARAF	V-raf murine sarcoma 3611 viral oncogene-like protein isoform 2	IGDFGLATVK*TR	459	1	1731650			
A0A0S2Z3L2	ATP2A2	ATPase Ca++ transporting cardiac muscle slow twitch 2 isoform 1	NYLEPGK*ECVQPATK	995	0.999	4384300			
A0A0S2Z3S6	CYBB	Cytochrome b-245 beta polypeptide isoform 1	VVITK*VVTHPFK	299	0.999	1367100			
A0A0S2Z428	KRT6A	HCG2039812, isoform CRA_b	EQIK*TLNNK	168	1	98273000			
			FASFIDK*VR	180	1	126376667	23482000	5.38	2.57E-04
			FLEQQNK*VLETK	189	1	##########	10115833	159.87	1.58E-03
			K*DVDAAYMNK	272	1	226880000			
			K*LLEGEECR	467	1	29342667			
			K*QCANLQAAIADAEQR	400	1	18802800			
			LLK*EYQELMNVK	446	1	2116667			
			NTK*QEIAEINR	378	1	70925000			
			TLNNK*FASFIDK	173	1	34488667			

			VELQAK*ADTLTDEINFLR	287	1	12984000			
			WTLLQEQGTK*TVR	204	1	69862333			
A0A0S2Z489	PSMD12	Proteasome (Prosome, macropain) 26S subunit, non-ATPase, 12, isoform CRA_a	LTK*TLATIK	147	1	49735667			
A0A0S2Z4R4	HGS	Hepatocyte growth factor-regulated tyrosine kinase substrate isoform 1	ATSTTELPPEYLTSPLSQQSQLPPK*R	251	1	7627467			
A0A0S2Z4Y4	CLINT1	Clathrin interactor 1 isoform 1	IGSTIDDTISK*FR	218	1	4454667			
A0A0S2Z4Z9	NONO	Non-POU domain containing octamer-binding isoform 1	ALIEMEK*QQQDQVDR	279	1	4609100			
			GIVEFSGK*PAAR	198	1	7352767			
			LVIK*NQQFHK	243	0.999	19713000			
A0A0S2Z5V7	HM13	Histocompatibility 13 isoform 1	GK*NASDMPETITSR	61	1	1839190			
A0A140TA33	TNXB	Tenascin-X	VTISGLEPDNK*YK	2366	0.832		2602633		
A0A140VJG8	COMT	Testicular tissue protein Li 42	K*GTVLLADNVICPGAPDFLAHVR	212	1	27038333	10424400	2.59	8.83E-03
A0A140VJK6	GMPS	Testicular tissue protein Li 82	SGNIVAGIANESKK*	183	0.908	8026933			
A0A140VJL3	HPRT1	Testicular tissue protein Li 89	MVK*VASLLVK	159	1	14026467	5564967	2.52	3.86E-02
A0A140VJQ6	OSBPL11	Oxysterol-binding protein	VVLPTFILEK*R	395	1	3165300			

A0A140VJS0		Serine/threonine-protein phosphatase	ELDQWVEQLNECK*QLNENQVR	21	1	12665000	26560667	0.48	4.19E-02
A0A140VJS3	PSMC5	26S protease regulatory subunit 8	K*IAELMPGASGAEVK	346	1	6329200			
			LDILK*IHSR	330	1	18141000			
			NIK*VIMATNR	290	1	10724233			
A0A140VJT0	PPP2R2A	Serine/threonine-protein phosphatase 2A	VVIFQQEQENK*IQSHSR	62	1	3207467			
A0A140VJT8	RNH1	Ribonuclease inhibitor	AKPDFK*ELTVSNNDINEAGVR	173	0.877		16521000		
			AK*ESLKELSLAGNELGDEGAR	283	1		13921667		
			DLCGIVASK*ASLR	226	1	724120	39561667	0.02	1.53E-03
			DSPCQLEALK*LESCGVTSDNCR	205	1		12000667		
			ELALGSNK*LGDVGMAELCPGLLHPSSR	238	1		35136333		
			LQALEK*DKPSLR	452	0.989		60279000		
			SNELGDVGVHCVLQGLQTPSCK*IQK	86	0.886		9840133		
			VLCQGLK*DSPCQLEALK	195	1		15670800		
A0A140VJZ1	USP5	Ubiquitin carboxyl-terminal hydrolase 5	ALIGK*GHPEFSTNR	423	1	78502667	29308000	2.68	1.07E-02
			IGEWELIQESGVPLK*PLFGPGYTGIR	318	1	4528433			

			SAADSISESVPVGPK*VR	793	1	16568667	5725467	2.89	3.31E-02
A0A140VK42	PSMC3	26S protease regulatory subunit 6A	K*MNVSPDVNYEELAR	372	1	1752400			
			LLDSEIK*IMK	53	1	1727750	5224300	0.33	1.06E-02
A0A140VK56	TALDO1	Transaldolase	LIELYK*EAGISK	130	1	4997800			
A0A140VK70	PSMC2	26S protease regulatory subunit 7	QIK*QVEDDIQQLLK	46	1	6017400	1213663	4.96	4.59E-03
A0A140VKA6	ST13	Hsc70-interacting protein	LSAK*FGGQA	364	1	72780333	10743500	6.77	1.96E-02
			YQSNPK*VMNLISK	353	1	7436167			
A0A1B0GVY1	NEDD4L	E3 ubiquitin-protein ligase NEDD4-like	SLSSPTVTLSAPLEGAK*DSPVRR	741	1	2528067			
A2RU94	RAB27A	RAB27A, member RAS oncogene family	CVDK*SWIPEGVVR	191	1	1586650			
A3R0T8	HIST1H1E	Histone 1	SETAPAAPAAPAPAEK(0.28)TPVK(0.72)	21	0.72	12280167	118173667	0.10	9.18E-03
A4D177	CBX3	Chromobox homolog 3	SLSDSESDDSK(0.413)SK(0.587)	105	0.587	2676050			
A4D2P0	RAC2	Ras-related C3 botulinum toxin substrate 2 (Rho family, small GTP binding protein Rac2)	MQAIK(1)CVVVGDGAVGK	5	1	4380833			
A4D2Q0	UNC84A	Unc-84 homolog A	EQTTVDHFWGLDDDGDLKGGNK*	221	0.558	2794033	13836000	0.20	5.26E-04
			K*DVLTAHPAAPGPVSR	253	1	3653200	8860600	0.41	3.92E-02

A5Y5A3	TPD52	PC1/MRPS28 fusion protein	VEEEIQTLSQVLAAK*EK	100	0.773	8423850			
A7UJ17	DNAJB6	DnaJ homolog subfamily B member 6	QVAEAYEVLSDAKK*R	61	0.55	7500100			
Q5EFE5		Anti-RhD monoclonal T125 gamma1 heavy chain	FNWYVDGVEVHNAKTK*PR	318	0.5		3819950		
		Anti-RhD monoclonal T125 gamma1 heavy chain	FNWYVDGVEVHNAK*TKPR	316	0.818		5002650		
A8K0M6	TMEM87A	Transmembrane protein 87A	FAFSPLSEEEEEDEQK*EPMLK	484	0.996	10838233			
A8K0R1	DNAJB2	DnaJ homolog subfamily B member 2	SASADDIKK*	21	0.579	11633500	5665200	2.05	1.53E-02
A8K287	SNAP23	Synaptosomal-associated protein	TITMLDEQK*EQLNR	49	1	13061500			
A8K2G0		cDNA FLJ76605, highly similar to Homo sapiens secretory carrier membrane protein 1 (SCAMP1), transcript variant 1, mRNA	TVQTAAANAASTAASSAAQNAFK*GNQI	334	1	4723133	14354500	0.33	2.40E-03
A8K2M0	PSMC4	26S protease regulatory subunit 6B	ENAPAIIFIDEIDAIATK*R	273	1	8303567			
A8K2T7	EGFR	Epidermal growth factor receptor	GLWIPEGEK*VKIPVAIK	737	0.986	72020333	2878400	25.02	2.76E-03
			IK*VLGSGAFGTVYK	716	1	7182967			
			ITDFGLAK*LLGAEEK	860	1	13363000			
			VK*IPVAIK	739	1	76911000	4852300	15.85	2.08E-03
A8K2U2	HK2	Hexokinase-2	EELLFGGK*LSPELLNTGR	323	1	1455850			

			TLEHLQLSHDQLLEVK*R	488	1	2250000			
A8K2W3	SDPR	Serum deprivation-response protein	QISLEGSVK*GIQNDLTK	91	1	2499520	50769000	0.05	1.58E-03
			YQASTSNTVSK*LLEK	113	0.997	8479300	89404667	0.09	4.35E-05
A8K556	GPRC5A	Retinoic acid-induced protein 3	AHAWPSPYKDYEVKK*	354	0.563		37251333		
			K*SYGVENR	291	1	7761800	254813333	0.03	2.45E-02
			QRNPMDYPVEDAFCK*PQLVK	285	1	6283200	136276667	0.05	2.70E-03
A8K5D4	MPZL1	Myelin protein zero-like 1, isoform CRA_b	DYTGCSTSESLSPVK*QAPR	213	1	2699967			
A8K7F6	EIF4A1	Eukaryotic initiation factor 4A-I	DFTVSAMHGDMDQK*ER	309	1	34460333			
			K*GVAINMVTEEDKR	369	1	20796000			
			KGVAINMVTEEDK*R	381	1	4192700			
A8K9G0		cDNA FLJ78634, highly similar to Homo sapiens solute carrier family 34 (sodium phosphate), member 2 (SLC34A2), mRNA	ETNK*NNTEAPVTK	37	1		13147800		
A8K9K6	NOP56	Nucleolar protein 56	SK*MSQVAPSLSALIGEAVGAR	288	1	1498667			
B0I1T2	MYO1G	Unconventional myosin-Ig	AK*VAAMGALQGLR	815	1	6919933			
B0QZ18	CPNE1	Copine-1	AQGWAPLKPLPPSAK*DPAQAPQA	534	0.896	8956100			

B0YJ88	RDX	Radixin	K*QLQALSSELAQAR	526	1	2675933			
			VLEQHK*LTK	162	1	8518533	136634333	0.06	4.57E-02
B2R4P9	H3F3A	Histone H3	SAPSTGGVK*KPHR	37	0.91	6369150	18078667	0.35	2.85E-02
B2R4R0	HIST1H4H	Histone H4	DNIQGITK*PAIR	32	1	187833333	66228333	2.84	4.90E-03
			GVLK*VFLENVIR	60	1	1905350			
B2R6J2	EZR	Ezrin	FGDYNK*EVHK	139	1	3616200	15984667	0.23	1.70E-03
B2R6U9	JAG1	Delta-like protein	EQLNQIK*NPIEK	1123	0.989	7615333			
B2R761	SCP2	Non-specific lipid-transfer protein	GSVLPNSDKK*	492	0.573		6877400		
			GSVLPNSDK*K	491	0.981	3078500			
B2R914	SCP2	Non-specific lipid-transfer protein	DIEEIK*TQK	122	0.999	42979667			
			SEFK*DIEEIK	116	1	16215033			
B2R960	TXNL1	Thioredoxin-like protein 1	IK*QHLENDPGSNEDTDIPK	104	1	4758500			
B2RBP3	UBA3	NEDD8-activating enzyme E1 catalytic subunit	VPNCNVVPHFNK*IQDFND TFYR	147	1	8662067	29567333	0.29	8.35E-03
B2RE75	MERTK	Tyrosine-protein kinase Mer	LQLEK*LLESLPDVR	856	1	3696900	579933	6.37	3.33E-03
B3KR37	EPS15	Epidermal growth factor receptor substrate 15	EK*DPEIFCDPFTSATTTTN K	430	1		14547333		

B3KXY9		cDNA FLJ46359 fis, clone TESTI4049786, highly similar to Hexokinase-1 (EC 2.7.1.1)	DFNPTATVK*MLPTFVR	97	1	1027970			
B4DE59	KRT19 Keratin	Keratin, type I cytoskeletal 19 OS=Homo s	SLLEGQEDHYNNLSASK*VL	561	1	5327300			
B4DGC6		cDNA FLJ58177, highly similar to Transmembrane protein 94	ALSVLK*EQLEAVLEGHLR	37	1	9129300			
B4DIH5	COPS2	COP9 signalosome complex subunit 2	IDQVNQLLELDHQK*R	351	1	3918433			
B4DKQ5	TOM1	Target of Myb protein 1	KEVK*YEAPQATDGLAGALDAR	394	0.8	5936433			
			K*GLEFPMTDLDMLSPIHTPQR	156	1	2437100			
B4DNB9	AP2M1	AP-2 complex subunit	VVIK*SNFKPSLLAQK	333	1	52845000			
B4DPJ8		cDNA FLJ52344, highly similar to T-complex protein 1 subunit zeta	AAVK*TLNPK	5	0.999	26940000	10010133	2.69	9.12E-03
B4DWA6	CAPZB	Capping protein (Actin filament) muscle Z-line, beta, isoform CRA_d	STLNEIYFGKTK*	300	0.702		9646300		
			STLNEIYFGK*TK	298	0.927	16524500			
B4DZF2		cDNA FLJ59571, highly similar to Eukaryotic translation initiation factor 4gamma 2	LLISK*LQDEFENR	243	1	2475067			
B4E0B1	SLFN5	Schlafen family member 5	NSECLK*EQQK	371	1	2123500			
B4E0Y9	STK24	Serine/threonine-protein kinase 24	LADFGVAGQLTDTQIK*R	194	1	4478600			
B4E1U9	CDC42	Cell division cycle 42 (GTP binding protein, 25kDa), isoform CRA_a	AVK*YVECSALTQK	198	1	15904333			

B4E2S3	RFTN1	RFTN1 protein	K*IQEAASQGLK	361	1	6675933	19214333	0.35	1.97E-02
B4E3D4	GPNMB	Transmembrane glycoprotein NMB	AVFFPGNQEKDPLLKNQEFK*	604	0.631	10884233			
B4E3I3	UFD1L	Ubiquitin fusion degradation 1 like, isoform CRA_b	GGK*IIMPPSALDQLSR	45	1	4806900			
B5MCA4	EPCAM	Epithelial cell adhesion molecule	AEIK*EMGEMHR	331	1	16770667			
B7Z3K9	ALDOC	Fructose-bisphosphate aldolase	VDK*GVVPLAGTDGETTTQGLDGLSER	198	1	12935100			
B7Z4C3	EPB42	Erythrocyte membrane protein band 4.2	QVEK*WSQPVHVAR	204	1		8997367		
B7Z6Z4	MYL6	Myosin light polypeptide 6	NK*DQGTYEDYVEGLR	174	1		3850550		
B7ZKM5	EPN2	Epsin-2	QLVALLK*DEER	135	1	24416667	67412667	0.36	7.34E-04
B7ZLH8	EVPL	Envoplakin, isoform CRA_a	SLK*EAEVLLK	88	1		13392367		
D1MGQ2	HBA2	Alpha-2 globin chain	AAWGK*VGAHAGEYGAEALER	17	1	3086200	60241333	0.05	2.86E-03
			MFLSFPTTK*TYFPHFDLSHGSAQVK	41	1	6943233	47453333	0.15	7.49E-04
			TNVK*AAWGK	12	1	9067100	148336667	0.06	2.92E-03
			TYFPHFDLSHGSAQVKGHGK*	61	0.573	1313967	40544000	0.03	2.88E-02
			TYFPHFDLSHGSAQVK*GHGK	57	0.953	3317650	49644333	0.07	3.80E-02
			VADALTNAVAHVDDMPNALSALSDLHAHK*LR	91	1	188843333	##########	0.05	1.10E-06

			VLSPADK*TNVK	8	0.999		29599333		
D3DNI3	PFN2	Profilin	EGVHGGTLNK*K	326	0.997	11854133			
			TK*SQGGEPTYNVAVGR	285	1	61698000			
D3DRP5	GLIPR2	Golgi-associated plant pathogenesis-related protein 1	QFHNEVLK*AHNEYR	212	1		3835267		
D3DS14	ARHGEF40	Rho guanine nucleotide exchange factor 40	FVHK*EGLMVGHQPSTLPPELPSGPPGLPSPPLPEEALGTP	189	1		6237267		
D3DV26	S100A10	S100 calcium binding protein A10 (Annexin II ligand, calpactin I, light polypeptide (P11)), isoform CRA_b	EFPGFLENQK*DPLAVDK	155	1	24818667	55644667	0.45	5.29E-03
			VLMEK*EFPGFLENQKDPLAVDK	145	1	12729700	49501333	0.26	3.06E-04
D9YZU5	HBB	Beta-globin	FFESFGDLSTPDAVMGNPKVK*	62	0.632	3042233	54282000	0.06	6.30E-03
			FFESFGDLSTPDAVMGNPK*VK	60	0.842		41211233		
			GTFATLSELHCDK*LHVDPENFR	96	1	59983667	621670000	0.10	1.84E-04
			K*VLGAFSDGLAHLDNLK	67	1	2427133	75085667	0.03	4.71E-05
			VHLTPEEK*SAVTALWGK	9	1	1973433	37851667	0.05	1.22E-04
			VLGAFSDGLAHLDNLK*GTFATLSELHCDK	83	1		4813267		
			VVAGVANALAHK*YH	145	1	28533333	172665000	0.17	1.84E-02

E1NZA1	GCN1L1	Peroxisome proliferator activated receptor interacting complex protein	DLAPYLPSVTPGLK*ASLLD PVPEVR	1663;	1	1122950			
E3UVQ2	BCOR-RARA	BCL6 corepressor-cyclin B3 fusion protein	RLIVNK*NAGETLLQR	1408	1	98486667	47728333	2.06	8.69E-03
E3WH17	SQSTM1	Tyrosine-protein kinase receptor	CSVCPDYDLCSVCEGK*GL HR	157	1	21644000			
E5KRG5	TYMP	Thymidine phosphorylase	VSLVLAPALAACGCK*VPMI SGR	139	1	2244900			
E7EMK3	FLOT2	Flotillin-2	NVQDIK*NVVLQTLEGHLR	155	1	5600533	354415	15.80	1.99E-02
E7EX29	YWHAZ	14-3-3 protein zeta/delta	VVSSIEQK*TEGAEK	68	0.996		2416007		
E9PAV3	NACA	Nascent polypeptide-associated complex subunit alpha, muscle-specific form	IEDLSQQAQLAAAEKFK*	2007	0.588	16473667			
E9PEJ6	ATP11A	Phospholipid-transporting ATPase	AADTIEALQK*AGIK	678	0.999	69929333	31607667	2.21	4.45E-03
			VIEGK*VDQIR	590	1	8181033	25840333	0.32	1.02E-02
E9PIE3	PRKCDBP	Protein kinase C delta-binding protein	SHDTTSNTLAQLLAK*AER	84	1	42051333	216750000	0.19	7.51E-04
E9PKP7	UBTF	Nucleolar transcription factor 1	LK*WVEISNEVR	71	1	2564750			
F1DSG4	AQP4	Aquaporin 4 transcript variant	AAQQTK*GSYMEVEDNR	274	1	8524267	84575333	0.10	1.10E-02
			KGK*DQSGEVLSSV	313	0.5		8009200		
			K*GKDQSGEVLSSV	311	0.588		12877933		
			SQVETDDLILK*PGVVHVID VDRGEEK	295	1	15621567	325490000	0.05	4.08E-02

F8VYN9	ARL1	ADP-ribosylation factor-like protein 1	IGISK*SELVAMLEEEELR	104	1	7795367			
F8VZU9	MYL6	Myosin light polypeptide 6	NK*DQGTYEDYVEGLR	33	1		3850550		
F8W930	IGF2BP2	Insulin-like growth factor 2 mRNA-binding protein 2	FAGASIK*IAPAEGPDVSER	468	1	11153450			
			NITK*QTQSR	227	1	5516800			
F8WCF6	ARPC4-TTLL3	Actin-related protein 2/3 complex subunit 4	SSK*ELLLQPVTISR	44	1	1434650			
G3V1N2	HBA2	HCG1745306, isoform CRA_a	MFLSFPTTK*TYFPHFDLSHGSAQVK	9	1	6943233	48808000	0.14	7.02E-04
			TYFPHFDLSHGSAQVK*GHGK	25	0.953	3317650	49644333	0.07	3.80E-02
G3V4X1	PSMC1	26S protease regulatory subunit 4	TLLAK*AVANQTSATF	74	1	7941300			
G3V5Z7	PSMA6	Proteasome subunit alpha type	K*VPDKLLDSSTVTHLFK	55	0.999	14948333	2045967	7.31	1.75E-03
H0Y300	HP	Haptoglobin	HYEGSTVPEKK*	358	0.568		19669000		
H0Y4R2	POR	NADPH--cytochrome P450 reductase	SYENQKPPFDAK*NPFLAAVTTNR	333	0.96	8968033			
			SYENQK*PPFDAK	327	1	13784467			
H0Y7A7	CALM2	Calmodulin	DGDGTITTK*ELGTVMR	69	1		5066167		
I4AY87	MIF	Macrophage migration inhibitory factor	SYSK*LLCGLLAER	78	1	7423533			
J3KPF3	SLC3A2	4F2 cell-surface antigen heavy chain	FTGLSK*EELLK	167	1	53691667			

J3KPQ4	ARHGAP9	Rho GTPase activating protein 9, isoform CRA_a	VSGNLAVVQK*LR	659	1	5802000			
J3QLH6	PSMC5	26S protease regulatory subunit 8	VSGSELVQK*F	214	1	19623333			
K7ELC2	RPS15	40S ribosomal protein S15	EAPPMEKPEVVK*THLR	84	1	15901000			
K7EPF9	APOC1	Apolipoprotein C-I	LKEFGNTLEDK*AR	47	1	20476000	67669667	0.30	4.65E-02
L7N2F9	VAMP2	Vesicle-associated membrane protein 2	DQK*LSELDDR	59	1	5907633	29678333	0.20	5.53E-03
M0QYS1	RPL13A	60S ribosomal protein L13a	LAAIVAK*QVLLGR	24	1	6425000			
O00231	PSMD11	26S proteasome non-ATPase regulatory subunit 11	EASIDILHSIVK*R	32	1	2674600			
O14495	PLPP3	Phospholipid phosphatase 3	K*EILSPVDIIDR	293	1		1490600		
O14727	APAF1	Apoptotic protease-activating factor 1	LPLNIEEAK*DR	224	1	7756200			
O15162	PLSCR1	Phospholipid scramblase 1	FTIQNEK*R	223	1	25526333	4755200	5.37	3.56E-02
			SLDEQCVVGK*ISK	258	0.981	2242650			
O15247	CLIC2	Chloride intracellular channel protein 2	LNIIK*VAAK	194	1	7966300	74717667	0.11	5.17E-03
O43175	PHGDH	D-3-phosphoglycerate dehydrogenase	NAGNCLSPAVIVGLLK*EASK	380	0.8	2405550			
			SATK*VTADVINAAEK	58	1	2080250			
O43242	PSMD3	26S proteasome non-ATPase regulatory subunit 3	LVSK*SVFPEQANNNEWAR	273	1	2132100			

O43291	SPINT2	Kunitz-type protease inhibitor 2	TVWSSGDDK*EQLVK	242	1	9592600			
O43491	EPB41L2	Band 4.1-like protein 2	ELEEK*VAELHK	374	1		4330550		
			GLSPAQADSQFLENAK*R	399	1	2512290	11551600	0.22	3.44E-02
			GQVLFDK*VCEHLNLLEK	246	1	2988733	32127000	0.09	2.64E-04
O43567	EPB41L2	Band 4.1-like protein 2	CVDPWLTK*TK	273	0.865	10592367			
			K*GDEYDVCAICLDEYEDG DKLR	233	1	8548267			
O60218	AKR1B15	Aldo-keto reductase family 1 member B15	LLNK*PGLK	173	1	128918000	8640000	14.92	3.19E-02
O60493	SNX3	Sorting nexin-3	K*QGLEQFINK	119	1	5734033			
			VVVPPLPGK*AFLR	95	1	3824000			
O60716	CTNND1	Catenin delta-1	ELIGK*HAIPNLVK	749	1	2544467			
			ESK*TPAILEASAGAIQNLC AGR	676	1	6375033			
			K*GGPPPPNWR	355	1	107280333	21515333	4.99	3.17E-02
			LK*GIPVLVGLLDHPK	408	1	1862533			
			LVLINK*SGNR	810	1	14877000			
O75312	ZPR1	Zinc finger protein ZPR1	TNEVK*SGGAVEPLGTR	299	1	19857333	5542150	3.58	2.51E-02

O75592	MYCBP2	E3 ubiquitin-protein ligase MYCBP2	NITINCVK*GINAR	3750	1	3419700			
O75832	PSMD10	26S proteasome non-ATPase regulatory subunit 10	ESILADK*SLATR	30	1	2463450			
O94888	UBXN7	UBX domain-containing protein 7	DVWSNEAVK*NIIR	194	1	2257067			
O95183	VAMP5	Vesicle-associated membrane protein 5	GVK*LAELQQR	33	1		2264350		
O95236	APOL3	Apolipoprotein L3	TYAAIEDEYVQQK*DEQFR	144	1	3457967			
O95436	SLC34A2	Sodium-dependent phosphate transport protein 2B	EAQGEVPASDSK*TECTAL	684	1	8349467	30182333	0.28	3.01E-03
P00558	PGK1	Phosphoglycerate kinase 1	ALESPERPFLAILGGAK*VADK	216	0.813	4220333			
P00915	CA1	Carbonic anhydrase 1	ASPDWGYDDK*NGPEQWSK	11	1		4795467		
			HDTSLK*PISVSYNPATAK	46	1	3181933	41008667	0.08	5.18E-03
			TSETK*HDTSLKPISVSYNPATAK	40	0.991		6111050		
P00918	CA2	Carbonic anhydrase 2	VGSAKPGLQK*VVDVLDSIK	158	0.936	744560	3857250	0.19	2.74E-02
P02545	LMNA	Prelamin-A/C	GQVAK*LEAALGEAK	171	1	22987667	118320000	0.19	4.49E-04
			IDSLSAQLSQLQK*QLAAK	311	1	7646000	26026667	0.29	6.97E-03
			K*LLEGEEER	378	1		8320900		
			LEAALGEAK*K	180	0.995	6680100	29638333	0.23	7.38E-03

			TYSAK*LDNAR	270	1	10729333	35418333	0.30	3.33E-02
P02549	SPTA1	Spectrin alpha chain, erythrocytic 1	VNILTDK*SYEDPTNIQGK	79	1		26577000		
P04075	ALDOA	Fructose-bisphosphate aldolase A	VDK*GVVPLAGTNGETTTQ GLDGLSER	111	1	27479333	7161500	3.84	4.25E-04
			YASICQQNGIVPIVEPEILPD GDHDLK*R	200	1	27157000	7144767	3.80	1.30E-04
P04083	ANXA1	Annexin A1	AAMK*GLGTDEDTLIEILAS R	128	1	1397500	3597767	0.39	5.46E-03
			CATSK*PAFFAEK	274	1	34771000	5508333	6.31	1.45E-03
P04406	GAPDH	Glyceraldehyde-3-phosphate dehydrogenase	AGAHLQGGAK*R	117	1	9381300			
			GALQNIIPASTGAAK*AVGK	215	0.995	283630000	88213000	3.22	4.19E-02
			TVDGPSGK*LWR	194	1	42049333	15757333	2.67	8.38E-03
			VIHDNFGIVEGLMTTVHAIT ATQK*TVDGPSGK	186	0.999	6037967	357165	16.91	1.75E-04
P04792	HSPB1	Heat shock protein beta-1	AQLGGPEAAK*SDETAAK	198	1	23705000			
P04899	GNAS	Guanine nucleotide-binding protein G(s) subunit alpha isoforms Xlas [illegible]	LLLLGAGESGK*STIVK	46;	0.973	1358200			
P05023	ATP1A3	Sodium/potassium-transporting ATPase subunit alpha-3	AAVPDAVGK*CR	605	1	18617333			
			AVFQANQENLPILK*R	444	1	6603633			
			CIELCCGSVK*EMR	468	1	52544333	2296233	22.88	1.05E-02

			DAK*ACVVHGSDLK	661	1	28561667			
			IISANGCK*VDNSSLTGESE PQTR	212	1	11508333			
P05556	ITGB1	Integrin beta-1	SAVTTVVNPK*YEGK	794	0.986	7310567			
P06396	GSN	Gelsolin	LFACSNK*IGR	675;	1	6679967			
			TASDFITK*MDYPK	368;	1		6990867		
P06454	PTMA	Prothymosin alpha	SDAAVDTSSEITTK*DLK	15	0.878	2048167			
P06576	ATP5B	ATP synthase subunit beta, mitochondrial	VLDSGAPIKIPVGPETLGR	133	1	3885700			
P06702	S100A9	Protein S100-A9	LGHPDTLNQGEFK*ELVR	38	1	2582133			
			TCK*MSQLER	4	1	11086000	64394333	0.17	3.48E-03
P07108	DBI	Acyl-CoA-binding protein	AYINK*VEELK	77	1	311980000	9395567	33.21	1.79E-03
P07195	LDHB	L-lactate dehydrogenase B chain	GLTSVINQKLK*	310	0.561	10249850			
P07737	PFN1	Profilin-1	TK*STGGAPTFNVTVTK	91	1	5343800			
P08069	IGF1R	Insulin-like growth factor 1 receptor	VAIK*TVNEAASMR	1033	1	2638400			
P08670	VIM	Vimentin	ETNLDSLPLVDTHSK*R	439	1	95412667	264336667	0.36	3.53E-03
			FLEQQNK*ILLAELEQLKGQ GK	129	1	11532667	39313667	0.29	1.00E-02

			ILLAELEQLK*GQGK	139	0.999	32149333	65454667	0.49	3.80E-03
			K*LLEGEESR	402	1	27038667	102537333	0.26	6.83E-03
			K*VESLQEEIAFLK	223	1	2201700	13038633	0.17	4.65E-02
			LREK*LQEEMLQR	188	1	322010000	792220000	0.41	2.40E-02
			RQVDQLTNDK*AR	168	1	103283000	492683333	0.21	2.34E-03
			RQVQSLTCEVDALK*GTNE SLER	334	1	59539667	163993333	0.36	2.55E-03
			TLLIK*TVETR	445	1	32237333	124916667	0.26	1.11E-03
P08754	GNAI3	Guanine nucleotide-binding protein G(k) subunit alpha	LK*IDFGEAAR	92	1	5316000			
P08758	ANXA5	Annexin A5	GAGTNEK*VLTEIIASR	108	1	12766467	33655667	0.38	1.62E-02
			HALK*GAGTNEK	101	1	15834000	35630000	0.44	1.43E-02
			LIVALMK*PSR	86	1		2179367		
			LYDAYELK*HALK	97	0.996	1474205	8769333	0.17	2.33E-02
P09769	FGR	Tyrosine-protein kinase Fgr	TEDDLTFTK*GEK	100	0.992	8070733			
			TLKPGTMSPK*AFLEEAQV MK	301	0.993	10635333			
			VAVK*TLKPGTMSPK	291	0.998	10641333			

P09936	UCHL1	Ubiquitin carboxyl-terminal hydrolase isozyme L1	CFEK*NEAIQAAHDAVAQEGQCR	135	1	4274567			
	UCHL1	Ubiquitin carboxyl-terminal hydrolase isozyme L1	MQLK*PMEINPEMLNK	4	1	45972667	4225100	10.88	2.63E-02
P11142	HSPA2	Heat shock 70kDa protein 2, isoform CRA_a	AMTK*DNNLLGK	451	1	6690833			
			CNEIINWLDK*NQTAEKEEFEHQQK	583	1	153980000	75430000	2.04	4.64E-02
			ELEK*VCNPIITK	601	1	77584333	11764400	6.59	2.22E-04
			GTLDPVEK*ALR	319	1	21934500			
			LDK*SQIHDIVLVGGSTR	328	1	992625			
			MVQEAEK*YKAEDEK	524	0.999	326256667	127954667	2.55	4.69E-04
			NQTAEKEEFEHQQK*ELEK	597	1	85154667			
P11169	SLC2A3	Solute carrier family 2, facilitated glucose transporter member 3	LWGTQDVSQDIQEMK*DESAR	243	1		6226133		
P11686	SFTPC	Surfactant protein C isoform 1	MDVGSK*EVLMESPPDYSAAPR	6	1	82885000	930846667	0.09	2.73E-04
P12004	PCNA	Proliferating cell nuclear antigen	ILK*CAGNEDIITLR	80	1	3214800			
P12109	COL6A1	Collagen alpha-1(VI) chain	LLLFSDGNSQGATPAAIEK*AVQEAQR	957	1		6629500		
P12268	IMPDH2	Inosine-5'-monophosphate dehydrogenase 2	GK*LPIVNEDDELVAIIAR	208	1	25047333	3244533	7.72	6.51E-04
			K*GKLPIVNEDDELVAIIAR	206	0.859	14432667	2532100	5.70	3.61E-03

P13010	XRCC5	X-ray repair cross-complementing protein 5	LGGHGPSFPLK*GITEQQK	195	0.999	7101433			
P13584	CYP4B1	Cytochrome P450 4B1	DEDDIK*LSDADLR	299	1		47775333		
			QLSK*PVTFVDGR	389	1		4433367		
P14618	PKM	Pyruvate kinase PKM	IISK*IENHEGVR	270	1	1450950			
P14735	IDE	Insulin-degrading enzyme	EVNAVDSEHEK*NVMNDAWR	192	1	3216700	55274333	0.06	3.90E-02
P15924	DSP	Desmoplakin	FGEK*VTQLTDR	859	1	6918233			
P16152	CBR1	Carbonyl reductase [NADPH] 1	ALK*SCSPELQQK	148	1	3773633			
P16157	ANK1	Ankyrin-1	LVK*PQKLSTPPPLAEEEGLASR	955	0.5		12458367		
			LVKPQK*LSTPPPLAEEEGLASR	958	0.5		12458367		
P16403	HIST1H1C	Histone H1.2	SETAPAAPAAAPPAEK*APVK	17	0.5	2421350	7058767	0.34	2.27E-02
			SETAPAAPAAAPPAEKAPVK*	21	0.5	2421350	7058767	0.34	2.27E-02
			SLVSK*GTLVQTK	93	1		2534700		
P17302	GJA1	Gap junction alpha-1 protein	GK*SDPYHATSGALSPAK	243	1	63351000			
			SDPYHATSGALSPAK*DCGSQK	258	1	187933333	22145333	8.49	5.56E-04
			VAQTDGVNVDMHLK*QIEIK	128	1	146180000	28758667	5.08	1.41E-02

P20700	LMNB1	Lamin-B1	IESLSSQLSNLQK*ESR	312	1	5992067			
			K*QLADETLLK	182	1	5773150			
P21796	VDAC1	Voltage-dependent anion-selective channel protein 1	AVPPTYADLGK*SAR	12	1	1752300			
P22626	HNRNPA2B1	Heterogeneous nuclear ribonucleoproteins A2/B1	LTDCVVMRDPASK*R	59	1	6247500			
P23396	RPS3	40S ribosomal protein S3	K*PLPDHVSIVEPK	202	1	5864067			
P25685	DNAJB1	DnaJ homolog subfamily B member 1	GASDEEIK*R	21	1	8437400			
P25705	ATP5A1	ATP synthase subunit alpha, mitochondrial	VGLK*APGIIPR	175	1	2023067			
P26038	MSN	Moesin	SGYLAGDK*LLPQR	151	1		6165033		
			VLEQHK*LNK	162	0.999	9876950	107870333	0.09	1.38E-02
			YGDFNK*EVHK	139	1	8246533	59866333	0.14	6.26E-03
P26447	S100A4	Protein S100-A4	ELPSFLGK*R	48	1	9840867	76290333	0.13	1.57E-02
P27708	CAD	CAD protein	DDQLK*VIECNVR	1211	1	2219300			
			LGPGK*GEVRPELGSR	1657	1	5368733			
			LSSFVTK*GYR	1411	1	4507267			
P28838	LAP3	Cytosol aminopeptidase	AAGIDEQENWHEGK*ENIR	118	1	4237050			

P29034	S100A2	Protein S100-A2	ELLHK*ELPSFVGEK	41	1	21743333			
			ELPSFVGEK*VDEEGLK	50	1	70906667			
P30050	RPL12	60S ribosomal protein L12	ELSGTIK*EILGTAQSVGCN VDGR	130	1	3166167			
P30876	POLR2B	DNA-directed RNA polymerase II subunit RPB2	EVLQK*EMLPHVGVSDFCE TK	345	1	3576033			
			HAIYDK*LDDDGLIAPGVR	847	1	7363967			
			HGQK*GTCGIQYR	942	1	4001150			
			TFIGK*IPIMLR	151	1	14207100			
			VSANK*GEIGDATPFNDAV NVQK	993	1	8308333			
			YSLATGNWGDQK*K	457	0.964	10849633			
P31949	S100A11	Protein S100-A11	AK*ISSPTETER	3	1	204976667	43570667	4.70	1.66E-02
			NQK*DPGVLDR	55	1	74838000	20146333	3.71	1.25E-03
P32320	CDA	Cytidine deaminase	IFK*GCNIENACYPLGICAE R	51	1	45002333	6573533	6.85	3.02E-04
P33527	ABCC1	Multidrug resistance-associated protein 1	RPVK*DGGGTNSITVR	636	1	36377333			
			TYQVAHMKSK*	498	0.713	11079067			
			TYQVAHMK*SK	496	1	68751667			

P35580	MYH10	Myosin-10	QLLQANPILESFGNAK*TVK	232	0.85	7519567	18467000	0.41	2.88E-03
P36382	GJA5	Gap junction alpha-5 protein	GSGSYEYPVAEK*AELSCWEEGNGR	130	1		6036133		
P38606	ATP6V1A	V-type proton ATPase catalytic subunit A	AK*EILQEEEDLAEIVQLVGK	480	1	2227567			
			IK*SDYAQLLEDMQNAFR	598	1	481775			
P47929	LGALS7	Galectin-7	SNVPHK*SSLPEGIRPGTVLR	7	1	12726333			
P49327	FASN	Fatty acid synthase	VVVQVLAEEPEAVLKGAK*	1869	0.582	4248267			
			VVVQVLAEEPEAVLK*GAKPK	1866	0.982	182153333	35315667	5.16	6.54E-05
P51572	BCAP31	B-cell receptor-associated protein 31	K*QAESASEAAK	138	1	1668200			
P51674	GPM6A	Neuronal membrane glycoprotein M6-a	SK*EEQELHDIHSTR	257	1		15875667		
P51970	NDUFA8	NADH dehydrogenase [ubiquinone] 1 alpha subcomplex subunit 8	KQQAK*FDECVLDK	106	1	101315000			
P52209	PGD	6-phosphogluconate dehydrogenase, decarboxylating	IK*DAFDRNPELQNLLLDDFFK	377	1	15993000	967870	16.52	1.66E-02
			VVGAQSLK*EMVSK	59	1	36890667	13871767	2.66	5.07E-03
P52272	HNRNPM	Heterogeneous nuclear ribonucleoprotein M	RGEIIAK*QGGGGGGGSVPGIER	388	1	44328333	2930050	15.13	4.82E-03
P52895	AKR1C2	Aldo-keto reductase family 1 member C2	LLEMILNK*PGLK	179	0.998	24153000			
P53621	COPA	Coatomer subunit alpha	GFFEGTIASKGK*	827	0.625	2039500	8681750	0.23	1.19E-02

			GFFEGTIASK*GK	825	0.803	6390333			
P55011	SLC12A2	Solute carrier family 12 member 2	GPIVPLNVADQK*LLEASTQFQK	1007	1	2620850	826250	3.17	5.26E-04
P55072	VCP	Transitional endoplasmic reticulum ATPase	ASGADSK*GDDLSTAILK	8	1	22679667	78176667	0.29	1.23E-04
			ETVVEVPQVTWEDIGGLEDVK*R	486	1	4555400	1937933	2.35	1.54E-02
			GDDLSTAILK*QK	18	0.896		18149667		
			KSPVAK*DVDLEFLAK	668	1	4631667	168396667	0.03	2.07E-04
			LDQLIYIPLPDEK*SR	651	1	836925	9470867	0.09	3.90E-02
			QLAQIK*EMVELPLR	217	1	3167100			
			VAILK*ANLR	658	1	36725000	99697667	0.37	4.95E-02
			VINQILTEMDGMSTK*K	614	0.5	13202150			
P56856	CLDN18	Claudin-18	K*IYDGGAR	239	1		18071600		
			TEDEVQSYPSK*HDYV	257	1	10223467	207346667	0.05	2.03E-04
P57735	RAB25	Ras-related protein Rab-25	VVLIGESGVGK*TNLLSR	25	1	2687900			
P58107	EPPK1	Epiplakin	DLAQGTK*TLQEVTEMDSVKR	2739	1	4445250			
			TLQEVTEMDSVK*R	2751	1	4555567			

P60174	TPI1	Triosephosphate isomerase	VAHALAEGLGVIACIGEK*LDER	168	1	1307000			
P61088	UBE2N	Ubiquitin-conjugating enzyme E2 N	DK*WSPALQIR	94	1	7546600	52940333	0.14	4.12E-02
			ICLDILK*DK	92	0.999	357116667	##########	0.26	2.52E-04
P62191	PSMC1	26S protease regulatory subunit 4	VAEEHAPSIVFIDEIDAIGTK*R	293	1	9735900			
P62249	RPS16	40S ribosomal protein S16	LLEPVLLLGK*ER	60	1	1385957			
P62258	YWHAE	14-3-3 protein epsilon	LICCDILDVLDK*HLIPAANTGESK	106	1	3620150	8381567	0.43	2.94E-02
P62879	GNB2	Guanine nucleotide-binding protein G(I)/G(S)/G(T) subunit beta 2	K*ACGDSTLTQITAGLDPVGR	23	1	2867800	23105667	0.12	2.99E-02
P62913	RPL11	60S ribosomal protein L11 OS=Homo sapiens	AAK*VLEQLTGQTPVFSK	38	1	5761600			
			VLEQLTGQTPVFSK*AR	52	1	15306667			
P62987	HEL112	Epididymis luminal protein 112	IQDK*EGIPPDQQR	33	1	40500333	13950000	2.90	3.75E-04
			MQIFVK*TLTGK	6	1	56149000	207173333	0.27	5.07E-03
			TITLEVEPSDTIENVK*AK	27	0.98	265603333	69675000	3.81	4.53E-05
			TLTGK*TITLEVEPSDTIENVKAK	11	1	##########	##########	0.50	1.28E-02
P63218	GNG5	Guanine nucleotide-binding protein G(I)/G(S)/G(O) subunit gamma 5	SGSSSVAAMKK*	12	0.556		4508700		
P68032	ACTA2	Actin, alpha 2, smooth muscle, aorta	VAPEEHPTLLTEAPLNPK*ANR	115	1	1822000	3951100	0.46	2.23E-02

P68104	EEF1A1	Elongation factor 1-alpha	FEK*EAAEMGK	44	1	17672667			
			KLEDGPK*FLK	392	1	25883333			
			VETGVLK*PGMVVTFAPVN VTTEVK	273	1	25590333			
P68363	TUBA1B	Tubulin alpha-1B chain	AYHEQLSVAEITNACFEPA NQMVK*CDPR	304	1	16050333			
			DVNAAIATIK*TK*R	338	1	209013333	22301333	9.37	1.68E-02
			DVNAAIATIK*TK*R	336	1	282603333	12912867	21.89	2.62E-02
			GDVVPK*DVNAAIATIK	326	1	47385333	9355267	5.07	8.93E-03
			QLFHPEQLITGK*EDAANN YAR	96	1	13905000	2932533	4.74	1.04E-03
			TIGGGDDSFNTFFSETGAG K*HVPR	60	1	29265000	5874100	4.98	1.15E-02
P68371	TUBB2A	Tubulin beta-2A chain	K*LAVNMVPFPR	252	1	68115667			
			MSMK*EVDEQMLNVQNK	324	1	39707667	11285733	3.52	4.20E-04
			NSSYFVEWIPNNVK*TAVC DIPPR	350	1	3353800			
			TLK*LTTPTYGDLNHLVSAT MSGVTTCLR	216	1	14417333			
P78527	PRKDC	DNA-dependent protein kinase catalytic subunit	K*GGSWIQEINVAEK	4023	1	9697100			
			LAAVVSACK*QLHR	1456	1	2935033			

P83916	CBX1	Chromobox protein homolog 1	NSDEADLVPAK*EANVK	150	0.829	1524210				
P98172	EFNB1	Ephrin-B1	AAALSLSTLASPK*GGSGT AGTEPSDIIIPLR	289	1	8524167				
Q01105	SET	Protein SET	EFHLNESGDPSSK*STEIK	167	0.99	3463767				
			VLSK*EFHLNESGDPSSK	154	1	9531500				
Q01118	SCN7A	Sodium channel protein type 7 subunit alpha	ELQEGNETDEAK*TIQIEMK	432	1		10371500			
			FQQTGK*ELQEGNETDEAK	420	1	3332633	40679000	0.08	2.89E-03	
			K*QEAVSATIIQR	1623	1		4159100			
Q01650	SLC7A5	Large neutral amino acids transporter small subunit 1	ALAAPAAEEK*EEAR	19	1	9955433				
Q01844	EWSR1	RNA-binding protein EWS	GDATVSYEDPPTAK*AAVE WFDGK	424	1	3285300				
Q02543	RPL18A	60S ribosomal protein L18a	VEEIAASK*CR	136	1	7088100	3118167	2.27	3.31E-03	
Q04828	AKR1C2	Aldo-keto reductase family 1 member C2	EEPWVDPNSPVLLEDPVLC ALAK*K	246	0.876	39880333				
			GVVVLAK*SYNEQR	270	1	24766567				
			LNDGHFMPVLGFGTYAPA EVPK*SK	31	0.738	5498150				
			MDSK*YQCVK	4	1	3861500				
			QLEMILNK*PGLK	179	0.999	13998967				

			SK*ALEATK	33	1	37584750			
			YQCVK*LNDGHFMPVLGF GTYAPAEVPK	9	1	16470000			
Q05682	CALD1	Caldesmon	SAK*PTKPAASDLPVPAEG VR	693	0.964	2321800			
Q09666	AHNAK	Neuroblast differentiation-associated protein AHNAK	VESEIK*VPDVELK	848	1		5705933		
			VPDVDIK*GPK	1360	0.993	4504750	38279000	0.12	9.85E-03
Q0JRZ9	FCHO2	F-BAR domain only protein 2	ADFEQK*MTETAQK	194	1	1981667			
Q12846	STX4	Syntaxin-4	LGNK*VQELEK	58	1	7973833			
Q12965	MYO1E	Unconventional myosin-Ie	DIILQSNPLLEAFGNAK*TV R	160	1	1250067			
Q13228	SELENBP1	Selenium-binding protein 1	TK*LVLPSLISSR	93	1	8129900	43616000	0.19	2.76E-04
Q13393	PLD1	Phospholipase D1	MPWHDIASAVHGK*AAR	688	1	11002833			
Q13571	LAPTM5	Lysosomal-associated transmembrane protein 5	MLQK*VVLPSYEEALSLPSK	233	1	3291467	10645333	0.31	2.48E-02
Q13619	CUL4A	Cullin-4A	LLVGK*SASVDAEK	465	1	2315400			
			TLQSLACGK*AR	635	1	4478633			
Q13740	ALCAM	CD166 antigen	DLGNMEENK*K	572	0.508	8123900			
Q14126	DSG2	Desmoglein-2	FLDDLGLK*FK	832	0.988	15528333			

			GSSSASIVK*GQHEMSEMDGR	706	1	15502333			
			SLQEANAEK*VTQEIVTER	901	1	3085767			
Q15012	LAPTM4A	Lysosomal-associated transmembrane protein 4A	MPEK*EPPPPYLPA	224	1	22777667	10513000	2.17	1.21E-04
Q15080	NCF4	Neutrophil cytosol factor 4	VYVGVK*QEIAEMR	98	1	2276600			
Q15365	PCBP1	Poly(rC)-binding protein 1	QMSGAQIK*IANPVEGSSGR	314	1	43432333	6048600	7.18	5.73E-05
Q15366	PCBP2	Poly(rC)-binding protein 2	LVVPASQCGSLIGKGGCK*	119	0.654	7953000	1729250	4.60	2.22E-03
			QMSGAQIK*IANPVEGSTDR	322	1	6048000			
Q15645	TRIP13	Pachytene checkpoint protein 2 homolog	VVNAVLTQIDQIK*R	288	1	6287167			
Q15742	NAB2	NGFI-A-binding protein 2	SPLELGEK*LSPLPGGPGAGDPR	169	1	7001633			
			SVGHIFEMDDNDSQK*EEEIR	291	1	2317733			
Q15758	SLC1A5	Neutral amino acid transporter B(0)	SELPLDPLPVPTEEGNPLLK*HYR	522	1	11790767			
			STEPELIQVK*SELPLDPLPVPTEEGNPLLK	502	1	27506000	3823467	7.19	3.69E-03
Q16186	ADRM1	Proteasomal ubiquitin receptor ADRM1	MSLK*GTTVTPDKR	34	1	143170000	63045000	2.27	1.24E-02
Q32Q12	NME1	Nucleoside diphosphate kinase A	TFIAIK*PDGVQR	37	1	28956667	6122550	4.73	2.02E-02
			VMLGETNPADSK*PGTIR	125	1	30203333	4233800	7.13	4.03E-03

Q3ZTS7	ANKRD13	Ankyrin repeat domain 13, isoform CRA_b	LTLDLMK*PK	217	0.915	37975000	76818667	0.49	4.75E-03
Q4LE27	ABCA3	ABCA3 variant protein	AVAGK*EEEDSDPEKALR	507	1	3561200	15788000	0.23	2.11E-02
			AVAGKEEEDSDPEK*ALR	516	1	3561200	17928667	0.20	3.59E-02
Q4LE36	ACLY	ACLY variant protein	SMGFIGHYLDQK*R	1113	1	2589850			
Q4LE69	PIK4CA	PIK4CA variant protein	ITNVSAIIK*PYPK	1768	0.995	2949500			
Q4W4Y1	DRIP4	Dopamine receptor interacting protein 4	MK*QSNNEANLR	640	1	27086333	9787567	2.77	6.55E-03
Q53EM5	TKT	Transketolase	ESWHGK*PLPK	260	1	53386333			
			GITGVEDK*ESWHGKPLPK	254	1	100778000			
			K*KILATPPQEDAPSVDIANIR	282	0.5	96477000			
			K*ILATPPQEDAPSVDIANIR	283	1	111254000			
			K*ISSDLDGHPVPK	102	1	32103667			
Q53F64	HNRNPAB	Heterogeneous nuclear ribonucleoprotein AB isoform a variant	MFVGGLSWDTSKK*	84	0.548	9201367			
			MFVGGLSWDTSK*K	83	0.5	4167550			
Q53G42	DCPS	mRNA decapping enzyme variant	QLNDVK*TTVVYPATEK	128	1	1612400			

Q53GL6	RALY	RNA binding protein (Autoantigenic, hnRNP-associated with lethal yellow) long isoform variant	VFIGNLNTALVK*K	34	0.843	7092933			
Q53GY0	PLS3	Plastin-3	VYALPEDLVEVKPK*	613	0.565	5461400	1444963	3.78	1.61E-02
Q549N5	SRPRB	Signal recognition particle receptor beta subunit	SAK*LIQQQLEK	191	1	4177867			
Q562R1	ACTBL2	Beta-actin-like protein 2	IK*IIAPPERK	329	1	204406667	21053667	9.71	5.56E-04
Q59E85	CAV1	Caveolin	AMADELSEK*QVYDAHTK	78	1	49055000	213376667	0.23	2.17E-02
			EIDLVNRDPK*HLNDDVVK	96	1		15908667		
			EQGNIYKPNNK*AMADELSEK	69	0.986	24800000	106507333	0.23	1.88E-02
			EQGNIYK*PNNK	65	0.999	8902500	72841000	0.12	1.66E-02
			QVYDAHTK*EIDLVNR	86	1	17464667	87394667	0.20	1.68E-02
Q59EG8	PSMD2	Proteasome 26S non-ATPase subunit 2 variant	EWQELDDAEK*VQR	183	1	30879667			
Q59EV7	SLC6A8	Transporter	GPLIAPGPDGAPAK*GDGPVGLGTPGGR	52	1	1941450			
Q59GM9	PYGM	Alpha-1,4 glucan phosphorylase	VLYPNDNFFEGK*ELR	312	1	3498133			
Q59GX2	SLC2A1	Solute carrier family 2 (Facilitated glucose transporter), member 1 variant	LRGTADVTHDLQEMK*EESR	270	1	321283333	2896200	110.93	1.17E-03
			QGGASQSDK*TPEELFHPLGADSQV	502	1	48302333			

Q59H06	TAP2	Transporter 2, ATP-binding cassette, sub-family B isoform 1 variant	QDLGFFQETK*TGELNSR	246	1	15477667			
Q5H9Q6	IMPDH	Inosine-5'-monophosphate dehydrogenase	GK*LPIVNDCDELVAIIAR	283	1	11673933	2667533	4.38	7.31E-03
			K*GKLPIVNDCDELVAIIAR	281	0.538	8916400	1489603	5.99	2.66E-02
Q5JSH3	WDR44	WD repeat-containing protein 44	ITGIEPLPGENK*ILVTSNDS R	756	1	10931867			
Q5JTV8	TOR1AIP1	Torsin-1A-interacting protein 1	MQNDSILK*SELGNQSPST SSR	308	1		5729200		
Q5TDH0	DDI2	Protein DDI1 homolog 2	DMLLANPHELSLLK*ER	153	1	6049800	1885500	3.21	6.04E-03
			WAGIAK*GVGTQK	283	0.997	18009000	4850167	3.71	2.45E-02
Q5VVQ6	YOD1	Ubiquitin thioesterase OTU1	FGEDAGYTK*R	257	1		43418333		
Q5VWC4	PSMD4	26S proteasome non-ATPase regulatory subunit 4	ILSK*LHTVQPK	74	1	28412667	5504733	5.16	1.79E-02
Q5VXU3	CHIC1	Cysteine-rich hydrophobic domain-containing protein 1	SIQK*LIEWENNR	176	1	18900000			
Q6IA69	NADSYN1	Glutamine-dependent NAD(+) synthetase	HK*MTTLTPAYHAENYSPE DNR	649	1	3991867			
Q6IBN1	HNRPK	HNRPK protein	HESGASIK*IDEPLEGSEDR	422	1		2469600		
			ILLQSK*NAGAVIGK	52	1	4639700			
			LLIHQSLAGGIIGVK*GAK	163	0.843	8439633			
Q6NX51	EXOC4	Exocyst complex component 4	QVPQK*LEQCMASK	140	1	3258167	1228630	2.65	1.85E-02

Q6NZI2	PTRF	Polymerase I and transcript release factor	AHATTSNTVSK*LLEK	109	1	63869333	235120000	0.27	5.94E-03
			K*LEVNEAELLR	137	1	850775	24746000	0.03	7.81E-03
			LGK*AHATTSNTVSK	98	1	34534333	162360000	0.21	1.44E-02
			QAEMEGAVQSIQGELSK*LGK	95	0.989	4869600	39774333	0.12	1.12E-02
			SLK*ESEALPEK	173	1	4153000	39582000	0.10	1.28E-02
			VMIYQDEVK*LPAK	161	0.999	15963367	127745667	0.12	3.11E-03
			VSVNVK*TVR	122	1	1538500	13701867	0.11	4.54E-02
Q6P2H0	DTX2	DTX2 protein	VQQALAGATPK*	19	1	202566667	5718400	35.42	2.44E-04
Q6P2Q9	PRPF8	Pre-mRNA-processing-splicing factor 8	DLILADYGKK*	1994	0.567	3791067			
			DLILADYGK*K	1993	0.5	2859700			
Q6ZN40	TPM1	Tropomyosin 1 (Alpha), isoform CRA_a	AELSEGK*CAELEEELK	231	1		2491600		
Q6ZRV2	FAM83H	Protein FAM83H	EGPEEPGLAK*QDSFR	664	1	5626633			
			VPAAFPTK*VPVPGPGSGGNGPER	639	1	4468067			
Q71DI3	HIST1H3A	Histone H3.1	K*SAPATGGVK	28	1	8967200	4219000	2.13	4.43E-03
			YQK*STELLIR	57	1	41441333	14149033	2.93	2.22E-02

Q7Z3R8	DKFZp686 D10173	DNA-directed RNA polymerase subunit beta	LSYISALGMMTRISSQFEK*	461	1	15817000			
Q86YT6	MIB1	E3 ubiquitin-protein ligase MIB1	DKDNTNVNADVQK*LQQQ LQDIK	949	1	11359100	3042550	3.73	7.08E-03
			GHGEWAEAMLPTLGK*VG R	368	1	2975367			
			KGQSPLDLCPDPNLCKALA K*	791	0.62	7456967			
			KGQSPLDLCPDPNLCK*AL AK	787	1	26019667	4204300	6.19	2.13E-02
			VDAAWEPSK*NTLIMGLGT QGAEK	735	1	1597667			
			WGK*QDGGEGHVGTVR	28	1	49775000	18184333	2.74	4.32E-02
Q8IUE6	HIST2H2A B	Histone H2A type 2-B	LLGGVTIAQGGVLPNIQAVL LPKK*	120	0.583	69120000	195746667	0.35	1.05E-03
Q8IY22	CMIP	C-Maf-inducing protein	TFLSK*ILTSK	74	1	43080333	16645000	2.59	5.82E-03
Q8N3F0	MTURN	Maturin	K*TLHEVLEK	80	1		5784500		
Q8NBQ5	HSD17B11	Estradiol 17-beta-dehydrogenase 11	EDIYSSAK*K	105	0.986		8450133		
Q8ND82	ZNF280C	Zinc finger protein 280C	GITLVCLK*CDFLADSSGLD R	689	1	2265067			
Q96AP7	ESAM	Endothelial cell-selective adhesion molecule	ALEEPANDIK*EDAIAPR	286	1	3897250	16438667	0.24	1.00E-02
Q96EI5	TCEAL4	Transcription elongation factor A protein-like 4	EYK*EAIHDMNFSNEDMIR	142	1	386800000	186510000	2.07	1.92E-03
Q96GG9	DCUN1D1	DCN1-like protein 1	AQIPK*MEQELKEPGR	143	1	59123667	5320033	11.11	1.18E-02

Q96GS6	ABHD17A	Protein ABHD17A	ELDTIEVFPTK*SAR	92	1	1859200			
Q96J02	ITCH	E3 ubiquitin-protein ligase Itchy homolog	FIYGNQDLFATSQSK*EFDPLGPLPPGWEK	432	1	76975000	25237000	3.05	4.60E-04
			VYYVDHVEK*R	350	1	4031000			
Q96RG5	IRS2	Insulin receptor substrate 2 insertion mutant	GLGQSK*NLTGVYR	213	1	11543867			
Q96S55	WRNIP1	ATPase WRNIP1	VLITENDVK*EGLQR	482	1	10548233			
Q99755	PIP5K1A	Phosphatidylinositol 4-phosphate 5-kinase type-1 alpha	GAIQLGITHTVGSLSTK*PER	103	1	36940667	103449667	0.36	8.36E-04
Q99808	SLC29A1	Equilibrative nucleoside transporter 1	LDLISK*GEEPR	255	1	12712467			
Q9BRK3	MXRA8	Matrix-remodeling-associated protein 8	SEDIQLDYK*NNILK	410	0.996	2986367			
Q9BRX8	FAM213A	OS=Homo sapiens GN=FAM213A PE=1 SV=3	AALEYLEDIDLK*TLEKEPR	55	0.976	3340267			
Q9BTT0	ANP32E	Acidic leucine-rich nuclear phosphoprotein 32 family member E	DLSTVEALQNLKNLK*	116	0.618	3933700			
			DLSTVEALQNLK*NLK	113	0.877	4239800			
Q9BYM8	RBCK1	RanBP-type and C3HC4-type zinc finger-containing protein 1	GPLEPGPPK*PGVPQEPGR	174	1		3173867		
Q9HBI6	CYP4F11	Phylloquinone omega-hydroxylase CYP4F11	DEDGK*ELSDEDIR	311	1	5823667			
			SVNIMHDK*WQR	177	1	3195600			
			TLPTQGIDDFLKNK*	290	0.578	28006667			

Q9NVH2	INTS7	Integrator complex subunit 7	SGQDYK*IPIDNMTNEMEQR	867	1	1429280	12624667	0.11	9.62E-03
Q9NX12	ITM2B	Integral membrane protein 2B	VTFNSALAQK*EAK	13	1	70378333	27793133	2.53	4.63E-02
Q9NX57	RAB20	Ras-related protein Rab-20	IVLLGDMNVGK*TSLLQR	18	1	2770633			
Q9NZA1	CLIC5	Chloride intracellular channel protein 5	LHVVK*IVAK	355	1	13495267	81066667	0.17	3.88E-03
Q9NZM1	MYOF	Myoferlin	LDMIPDLK*AMNPLK	1928	0.997	2661000	7307567	0.36	6.91E-03
Q9P225	DNAH2	Dynein heavy chain 2, axonemal	FIEPPVLNMK*SVLEDSTPR	3825	1	3902967			
Q9UBI6	GNG12	Guanine nucleotide-binding protein G(I)/G(S)/G(O) subunit gamma 12	VSK*ASADLMSYCEEHAR	34	1		5240967		
Q9UKJ5	CHIC2	Cysteine-rich hydrophobic domain-containing protein 2	SIEK*LLEWENNR	117	1	159696667	44920333	3.56	2.07E-03
Q9UM47	NOTCH3	Neurogenic locus notch homolog protein 3	GESLMGEVATDWMDTECPEAK*R	1730	1	2163600			
Q9Y277	VDAC3	Voltage-dependent anion-selective channel protein 3	CNTPTYCDLGK*AAK	12	0.976	2763350			
Q9Y3I0	RTCB	tRNA-splicing ligase RtcB homolog	GGGVGGFLPAMK*QIGNVAALPGIVHR	67	1	4917467			
Q9Y4R8	TELO2	Telomere length regulation protein TEL2 homolog	ETLLGK*VVALPDHLGNR	159	1	4636233			
U3PXP0	HBA2	Alpha globin chain	AAWGK*VGAHAGEYGAEALER	17	1	3086200	60241333	0.05	2.86E-03
U6FSN9	MPRIP	Tyrosine-protein kinase receptor	GWLTK*QYEDGQWK	396	1	39000333	101817667	0.38	4.48E-03
U6FVB0	CD74	Tyrosine-protein kinase receptor	RPGAPESK*CSR	43	1	13839667	40798000	0.34	2.81E-02

V9HW25	TPM2b	Epididymis secretory protein Li 273	CK*QLEEEQQALQK	37	1	12962433	49697000	0.26	2.15E-02
X6RAL5	SAP18	Histone deacetylase complex subunit SAP18	ELTSLVK*EVYPEAR	93	1	14797667	5697550	2.60	6.01E-03
