# Supplementary material for: Label-free quantitative identification of abnormally ubiquitinated proteins as useful biomarkers for human lung squamous cell carcinomas
Source: EPMA J. 2020 Jan 4;11(1):73–94. doi: 10.1007/s13167-019-00197-8 (PMC7028901; doi:10.1007/s13167-019-00197-8)
Supplement: Supplementary file 4 — (PDF 41 kb) [file 13167_2019_197_MOESM4_ESM.pdf]

Supplemental Table 2. Statistically significant KEGG pathways identified from differentially ubiquitinated proteins (DUPs) in LSCCs relative to controls.

| KEGG pathway | ID | Input number | Background number | P-Value | Corrected P-Value | DUPs |
|---|---|---|---|---|---|---|
| Epstein-Barr virus infection | hsa05169 | 18 | 312 | 1.57E-08 | 3.31E-06 | A0A140VJS3、A0A140VK42、A8K2M0、O00231、A0A140VK70、A0A087X2I1、P62191、P11142、A0A024R244、A0A0S2Z489、Q7Z3R8、P08670、P04792、P62258、P09769、O43242、P06454、P30876 |
| Proteasome | hsa03050 | 10 | 70 | 1.48E-07 | 1.57E-05 | O00231、A0A140VK70、P62191、A0A140VK42、A0A0S2Z489、A0A140VJS3、A0A087X2I1、O43242、A8K2M0、Q16186 |
| Leukocyte transendothelial migration | hsa04670 | 12 | 176 | 7.88E-07 | 5.54E-05 | Q96AP7、P04899、A0A024R694、A0A024R1P2、P26038、P08754、A0A024R324、P05556、P56856、O60716、A0A0S2Z3S6、Q15080 |
| Tight junction | hsa04530 | 12 | 197 | 1.37E-05 | 6.62E-04 | P68363、P05556 、P26038 P12004 A0A140VJT0、A0A024R694、P35580、A0A024R324、A0A140VJS0、A0A024QZJ4、P56856、A0A024R1N1 |
| Alcoholism | hsa05034 | 13 | 281 | 1.57E-05 | 6.62E-04 | P04899、B2R4P9、P62879、Q71DI3、P08754、B2R4R0、Q9UBI6、A0A024QZP6、A0A024RCJ2、Q8IUE6、A0A024R056、P63218、Q99808 |
| Glycolysis / Gluconeogenesis | hsa00010 | 8 | 104 | 2.48E-05 | 8.74E-04 | P60174、P14618、P04406、A0A024R4F1、P04075、P00558、P07195、B3KXY9 |
| Carbon metabolism | hsa01200 | 10 | 185 | 4.39E-05 | 1.32E-03 | B3KXY9、P52209、P60174、P14618、O43175、P04406、A0A024R4F1、P04075、A0A140VK56、P00558 |
| Regulation of actin cytoskeleton | hsa04810 | 13 | 327 | 7.03E-05 | 1.69E-03 | A0A024R694、B0YJ88、A0A024RC65、P35580、Q99755、P26038、P06396、Q9UBI6、P05556、A0A024R1N1、A0A024R324、P07737、A0A024R1P2 |
| Biosynthesis of amino acids | hsa01230 | 8 | 122 | 7.21E-05 | 1.69E-03 | P60174、P14618、O43175、P04406、A0A024R4F1、P04075、A0A140VK56、P00558 |

| | | | | | | |
|---|---|---|---|---|---|---|
| Arrhythmogenic right ventricular cardiomyopathy | hsa05412 | 7 | 105 | 1.86E-04 | 3.91E-03 | A0A024R694、P02545、Q14126、A0A0S2Z3L2、P05556、P17302、P15924 |
| Dopaminergic synapse | hsa04728 | 9 | 207 | 4.93E-04 | 9.46E-03 | P04899、P62879、A0A140VJT0、P08754、Q9UBI6、A0A140VJS0、A0A140VJG8、A0A024R056、P63218 |
| HIF-1 signaling pathway | hsa04066 | 8 | 170 | 6.15E-04 | 9.65E-03 | B3KXY9、P08069、P04406、A0A024R4F1、P04075、P00558、A0A0S2Z3S6、Q59GX2 |
| Metabolic pathways | hsa01100 | 35 | 1832 | 6.34E-04 | 9.65E-03 | P12268、Q13393、Q4LE69、O43175、P16152、A0A140VJL3、P49327、B2R761、P30876、P25705、P28838、E5KRG5、P60174、P14618、P04406、A0A024R4F1、P27708、A0A140VK56、P00558、A0A140VJG8、P52209、P51970、Q6IA69、A0A140VJK6、B3KXY9、A0A0C4DFL7、P06576、P07195、Q99755、P38606、P32320、Q7Z3R8、O60218、O14495、P04075 |
| Parkinson's disease | hsa05012 | 9 | 215 | 6.40E-04 | 9.65E-03 | P04899、P06576、P08754、P51970、P25705、O14727、Q9Y277、P21796、P09936 |
| Sphingolipid signaling pathway | hsa04071 | 8 | 184 | 1.01E-03 | 1.34E-02 | P04899、Q13393、A0A024R1P2、A0A140VJT0、P08754、A0A024R324、A0A140VJS0、P33527 |
| Adrenergic signaling in cardiomyocytes | hsa04261 | 9 | 230 | 1.01E-03 | 1.34E-02 | P04899、A0A024R968、A0A140VJT0、P05023、P08754、A0A0S2Z3L2、Q01118、A0A140VJS0、P68032 |
| Drug metabolism - other enzymes | hsa00983 | 5 | 71 | 1.24E-03 | 1.54E-02 | A0A140VJL3、P32320、E5KRG5、A0A140VJK6、P12268 |
| Endocytosis | hsa04144 | 12 | 394 | 1.31E-03 | 1.54E-02 | A0A024R8B8、A0A0S2Z4R4、A0A024R4S1、P08069、Q96J02、A0A024R0S6、Q99755、A0A024R324、Q13393、Q59E85、P11142、O60493 |
| Central carbon metabolism in cancer | hsa05230 | 6 | 111 | 1.51E-03 | 1.67E-02 | B3KXY9、Q15758、P14618、A0A024R8U1、Q59GX2、Q01650 |

| | | | | | | |
|---|---|---|---|---|---|---|
| Non-homologous end-joining | hsa03450 | 3 | 20 | 1.74E-03 | 1.83E-02 | A0A024R1N4、P13010、P78527 |
| Adherens junction | hsa04520 | 6 | 120 | 2.19E-03 | 2.20E-02 | A0A024R694、P08069、A0A024RC65、A0A024R324、O60716、A0A024R1P2 |
| Pathogenic Escherichia coli infection | hsa05130 | 5 | 84 | 2.50E-03 | 2.40E-02 | P05556、A0A024RAY2、A0A024R324、P68363、P68371 |
| Fructose and mannose metabolism | hsa00051 | 4 | 52 | 2.85E-03 | 2.62E-02 | O60218、B3KXY9、P04075、P60174 |
| GABAergic synapse | hsa04727 | 6 | 129 | 3.09E-03 | 2.72E-02 | P04899、P62879、P08754、Q9UBI6、A0A024R056、P63218 |
| Viral carcinogenesis | hsa05203 | 10 | 328 | 3.23E-03 | 2.73E-02 | A0A024R694、P14618、P62191、A0A024R324、A0A024R244、P06396、B2R4R0、A0A024RCJ2、Q9Y277、P62258 |
| Morphine addiction | hsa05032 | 6 | 133 | 3.57E-03 | 2.88E-02 | P04899、P62879、P08754、Q9UBI6、A0A024R056、P63218 |
| PI3K-Akt signaling pathway | hsa04151 | 13 | 512 | 3.93E-03 | 2.88E-02 | P62879、P08069、A0A140VJT0、A0A024R056、A0A024R244、A0A024RD80、A0A024QZA8、Q9UBI6、P05556、A0A140VJS0、P62258、P63218、P12109 |
| Glutamatergic synapse | hsa04724 | 7 | 182 | 3.96E-03 | 2.88E-02 | P04899、Q13393、P08754、P62879、Q9UBI6、A0A024R056、P63218 |
| Ribosome | hsa03010 | 7 | 182 | 3.96E-03 | 2.88E-02 | P23396、P62987、A0A024RBS2、P62249、P30050、P62913、Q02543 |
| Chemokine signaling pathway | hsa04062 | 9 | 285 | 4.10E-03 | 2.88E-02 | P04899、P62879、A0A024R1P2、P08754、A0A024R324、Q9UBI6、A0A024R056、P63218、P09769 |
| Phagosome | hsa04145 | 8 | 237 | 4.55E-03 | 3.10E-02 | A0A0S2Z4R4、A0A024RAU0、P68363、P68371、P38606、P05556、A0A0S2Z3S6、Q15080 |

| | | | | | | |
|---|---|---|---|---|---|---|
| Fc gamma R-mediated phagocytosis | hsa04666 | 6 | 144 | 5.17E-03 | 3.41E-02 | Q13393、A0A024R1P2、Q99755、A0A024R244、P06396、O14495 |
| Pancreatic secretion | hsa04972 | 6 | 149 | 6.05E-03 | 3.87E-02 | P00918、A0A024R968、P05023、A0A024R324、A0A0S2Z3L2、P55011 |
| Retrograde endocannabinoid signaling | hsa04723 | 6 | 151 | 6.43E-03 | 3.99E-02 | P04899、P62879、P08754、Q9UBI6、A0A024R056、P63218 |
| Circadian entrainment | hsa04713 | 6 | 155 | 7.25E-03 | 4.36E-02 | P04899、P62879、P08754、Q9UBI6、A0A024R056、P63218 |
| Rap1 signaling pathway | hsa04015 | 9 | 315 | 7.60E-03 | 4.36E-02 | P04899、A0A024R1P2、P08754、A0A024R324、A0A024QZA8、P05556、O60716、P07737、P08069 |
| Systemic lupus erythematosus | hsa05322 | 7 | 207 | 7.69E-03 | 4.36E-02 | B2R4P9、A0A024R694、Q71DI3、B2R4R0、A0A024QZP6、A0A024RCJ2、Q8IUE6 |
| cGMP-PKG signaling pathway | hsa04022 | 8 | 261 | 7.85E-03 | 4.36E-02 | P04899、A0A024R968、P05023、P08754、A0A024R324、A0A0S2Z3L2、Q9Y277、P21796 |
| Axon guidance | hsa04360 | 8 | 268 | 9.09E-03 | 4.92E-02 | P04899、A0A024R1P2、P98172、P08754、A0A024R324、A0A024RC92、A0A024QZA8、P05556 |
