# Supplementary material for: Label-free quantitative identification of abnormally ubiquitinated proteins as useful biomarkers for human lung squamous cell carcinomas
Source: EPMA J. 2020 Jan 4;11(1):73–94. doi: 10.1007/s13167-019-00197-8 (PMC7028901; doi:10.1007/s13167-019-00197-8)
Supplement: Supplementary file 5 — (PDF 42 kb) [file 13167_2019_197_MOESM5_ESM.pdf]

Supplemental Table 3. Topological properties of the nodes from PPI network with Cystoscope.

SUID	Average Shortest Path Length	Betweenness Centrality	Closeness Centrality	Clustering Coefficient	Column 2	Degree	Eccentricity	Is Single Node	MCODE Cluster	MCODE Node Status	MCODE Score	name	Neighborhood Connectivity	Number Of Directed Edges	Number Of Undirected Edges	Partner Of Multi-Edged Node Pairs	Radiality	selected	Self Loops	shared name	Stress	Topological Coefficient
86	1.8493	0.2093	0.5407	0.1735	UP	49	6	FALSE	Cluster 2|Cluster 3	Clustered	4.724	GAPDH	13.694	49	0	0	0.90563	FALSE	0	GAPDH	18872	0.1107
77	2.0068	0.1573	0.4983	0.1661	X	43	6	FALSE	Cluster 1	Clustered	7.418	UBA52	13.512	43	0	0	0.88813	TRUE	0	UBA52	16296	0.1224
83	2.0548	0.0591	0.4867	0.2646	UP	38	6	FALSE	Cluster 1	Clustered	7.457	HSPA8	16.421	38	0	0	0.8828	TRUE	0	HSPA8	8184	0.1466
108	2.1438	0.0662	0.4665	0.3057	UP	30	6	FALSE	Cluster 1	Clustered	6.949	CAD	16.900	30	0	0	0.87291	TRUE	0	CAD	6674	0.1603
106	2.1849	0.0522	0.4577	0.3148	UP	28	6	FALSE	Cluster 5|Cluster 2	Seed	6.171	EEF1A1	17.536	28	0	0	0.86834	FALSE	0	EEF1A1	6286	0.1746
123	2.1301	0.0822	0.4695	0.2063	UP	28	6	FALSE	Cluster 1	Clustered	6.611	PCNA	15.643	28	0	0	0.87443	TRUE	0	PCNA	8718	0.1442
87	2.1712	0.0458	0.4606	0.3675	UP	27	5	FALSE	Cluster 1	Clustered	7.813	PGK1	18.815	27	0	0	0.86986	TRUE	0	PGK1	5842	0.1809
125	2.2877	0.0309	0.4371	0.3504	UP	27	6	FALSE	Cluster 1	Clustered	7.962	POLR2B	17.259	27	0	0	0.85693	TRUE	0	POLR2B	3958	0.1813
100	2.2877	0.0217	0.4371	0.3636	UP	22	6	FALSE	Cluster 5|Cluster 2	Clustered	5.308	PKM	18.091	22	0	0	0.85693	FALSE	0	PKM	3552	0.1920
107	2.3493	0.0276	0.4257	0.4329	UP	22	6	FALSE	Cluster 1	Clustered	7.813	IMPDH2	18.136	22	0	0	0.85008	TRUE	0	IMPDH2	3106	0.2148
113	2.3082	0.0457	0.4332	0.2286	UP	21	6	FALSE	Cluster 2|Cluster 3	Clustered	5.000	VDAC1	14.524	21	0	0	0.85464	FALSE	0	VDAC1	5264	0.1603
141	2.2466	0.0132	0.4451	0.4667	UP	21	6	FALSE		Unclustered	5.933	NME2	21.190	21	0	0	0.86149	FALSE	0	NME2	1954	0.2098
182	2.1027	0.1028	0.4756	0.1905	DOWN	21	5	FALSE	Cluster 4	Clustered	2.303	VIM	14.095	21	0	0	0.87747	FALSE	0	VIM	7838	0.1211
79	2.2808	0.0229	0.4384	0.5556	UP	19	6	FALSE	Cluster 1	Clustered	7.457	RPS3	21.947	19	0	0	0.85769	TRUE	0	RPS3	2564	0.2189
89	2.3767	0.0067	0.4207	0.5380	X	19	6	FALSE	Cluster 1	Clustered	7.813	ATP5B	21.105	19	0	0	0.84703	TRUE	0	ATP5B	1470	0.2454
105	2.4110	0.0080	0.4148	0.4641	UP	18	6	FALSE	Cluster 1	Clustered	7.644	LDHB	18.778	18	0	0	0.84323	TRUE	0	LDHB	1316	0.2209

129	2.3014	0.0418	0.4345	0.1765	DOWN	18	6	FALSE	Cluster 5|Cluster 2	Clustered	5.000	ACTC1	14.389	18	0	0	0.8554	FALSE	0	ACTC1	6550	0.1557
144	2.2877	0.0438	0.4371	0.1961	UP	18	6	FALSE	Cluster 2|Cluster 3	Clustered	4.167	ACTBL2	15.722	18	0	0	0.85693	FALSE	0	ACTBL2	7068	0.1667
88	2.3973	0.0081	0.4171	0.5074	UP	17	6	FALSE	Cluster 1	Clustered	7.030	ATP5A1	20.412	17	0	0	0.84475	TRUE	0	ATP5A1	1566	0.2430
76	2.4658	0.0017	0.4056	0.7333	UP	15	6	FALSE	Cluster 1	Seed	7.962	RPL11	22.267	15	0	0	0.83714	TRUE	0	RPL11	432	0.2651
85	2.3562	0.0087	0.4244	0.5055	UP	14	6	FALSE	Cluster 1	Clustered	6.611	TPI1	23.000	14	0	0	0.84932	TRUE	0	TPI1	1174	0.2414
92	2.3425	0.0082	0.4269	0.4615	UP	14	7	FALSE	Cluster 2|Cluster 3	Clustered	4.933	TUBB4B	22.429	14	0	0	0.85084	FALSE	0	TUBB4B	1634	0.2289
95	2.3904	0.0179	0.4183	0.3077	X	14	6	FALSE		Unclustered	2.889	VCP	20.000	14	0	0	0.84551	FALSE	0	VCP	2506	0.2198
112	2.2466	0.0302	0.4451	0.3077	UP	14	5	FALSE	Cluster 4	Clustered	2.400	HSPB1	19.786	14	0	0	0.86149	FALSE	0	HSPB1	3406	0.1921
142	2.4726	0.0210	0.4044	0.1099	DOWN	14	6	FALSE		Unclustered	0.564	YWHAE	12.786	14	0	0	0.83638	FALSE	0	YWHAE	2332	0.1642
93	2.3151	0.0065	0.4320	0.5256	UP	13	6	FALSE	Cluster 2|Cluster 3	Clustered	4.933	TUBA1B	23.000	13	0	0	0.85388	FALSE	0	TUBA1B	1240	0.2323
115	2.6781	0.0131	0.3734	0.3077	UP	13	7	FALSE	Cluster 5|Cluster 2	Clustered	6.000	HNRNPM	11.692	13	0	0	0.81355	FALSE	0	HNRNPM	1298	0.2024
146	2.2260	0.0409	0.4492	0.2436	UP	13	6	FALSE		Unclustered	3.143	IGF1R	18.769	13	0	0	0.86377	FALSE	0	IGF1R	4350	0.1731
72	2.5685	0.0013	0.3893	0.7424	UP	12	7	FALSE	Cluster 1	Clustered	7.644	RPL12	21.417	12	0	0	0.82572	TRUE	0	RPL12	298	0.2818
81	2.5616	0.0006	0.3904	0.8182	UP	12	7	FALSE	Cluster 1	Clustered	7.152	RPS16	22.583	12	0	0	0.82648	TRUE	0	RPS16	164	0.2895
91	2.4589	0.0273	0.4067	0.2576	UP	12	6	FALSE		Unclustered	3.733	PRKDC	15.750	12	0	0	0.8379	FALSE	0	PRKDC	2730	0.1948
109	2.6644	0.0193	0.3753	0.2576	UP	12	7	FALSE		Unclustered	5.000	ITCH	10.500	12	0	0	0.81507	FALSE	0	ITCH	1760	0.1708
118	2.5685	0.0049	0.3893	0.3939	UP	12	6	FALSE	Cluster 5|Cluster 2	Clustered	6.000	HNRNPA2B1	18.833	12	0	0	0.82572	FALSE	0	HNRNPA2B1	736	0.2456
162	2.3151	0.0106	0.4320	0.4394	DOWN	12	6	FALSE	Cluster 4	Clustered	2.303	ANXA5	19.167	12	0	0	0.85388	FALSE	0	ANXA5	1550	0.1917
102	2.5274	0.0092	0.3957	0.2909	DOWN	11	6	FALSE		Unclustered	1.750	UBE2N	14.455	11	0	0	0.83029	FALSE	0	UBE2N	1032	0.1830
73	2.6164	0.0008	0.3822	0.7778	UP	10	7	FALSE	Cluster 1	Clustered	6.533	RPL18A	21.000	10	0	0	0.8204	TRUE	0	RPL18A	168	0.2917

99	2.6164	0.0096	0.3822	0.2000	UP	10	7	FALSE		Unclustered	2.000	UBXN7	15.000	10	0	0	0.8204	FALSE	0	UBXN7	1428	0.2130
110	2.4041	0.0040	0.4160	0.6000	UP	10	7	FALSE		Unclustered	3.889	HIST2H3A	24.000	10	0	0	0.84399	FALSE	0	HIST2H3A	714	0.2553
114	2.6986	0.0048	0.3706	0.4000	UP	10	7	FALSE	Cluster 5	Seed	3.429	VDAC3	14.800	10	0	0	0.81126	FALSE	0	VDAC3	758	0.2242
116	2.7466	0.0039	0.3641	0.4000	UP	10	7	FALSE	Cluster 5|Cluster 2	Clustered	6.000	PRPF8	14.000	10	0	0	0.80594	FALSE	0	PRPF8	328	0.2242
117	2.6301	0.0160	0.3802	0.3778	UP	10	7	FALSE		Unclustered	2.556	CUL4A	16.400	10	0	0	0.81887	FALSE	0	CUL4A	1686	0.2233
156	2.5685	0.0355	0.3893	0.3556	X	10	6	FALSE	Cluster 2	Clustered	5.000	ANXA1	13.000	10	0	0	0.82572	FALSE	0	ANXA1	3146	0.1925
176	2.4795	0.0039	0.4033	0.5111	UP	10	7	FALSE		Unclustered	4.762	PHGDH	23.000	10	0	0	0.83562	FALSE	0	PHGDH	638	0.2644
184	2.5068	0.0310	0.3989	0.2667	UP	10	7	FALSE		Unclustered	2.143	ABCC1	18.400	10	0	0	0.83257	FALSE	0	ABCC1	3048	0.2259
78	2.7808	0.0021	0.3596	0.5278	UP	9	7	FALSE	Cluster 3	Clustered	4.762	PSMC1	13.000	9	0	0	0.80213	FALSE	0	PSMC1	264	0.2241
82	2.4452	0.0007	0.4090	0.6944	UP	9	6	FALSE		Unclustered	3.778	DNAJB1	28.778	9	0	0	0.83942	FALSE	0	DNAJB1	228	0.3233
119	2.7329	0.0018	0.3659	0.6111	UP	9	7	FALSE	Cluster 2	Clustered	6.000	PCBP1	18.222	9	0	0	0.80746	FALSE	0	PCBP1	320	0.3037
120	2.8151	0.0309	0.3552	0.1111	DOWN	9	7	FALSE	Cluster 3	Unclustered	0.952	SPTA1	8.111	9	0	0	0.79833	FALSE	0	SPTA1	2896	0.1897
122	2.7397	0.0020	0.3650	0.6111	UP	9	7	FALSE	Cluster 1	Clustered	7.000	NACA	17.889	9	0	0	0.8067	TRUE	0	NACA	190	0.2914
148	2.5068	0.0508	0.3989	0.1667	UP	9	6	FALSE	Cluster 3	Unclustered	4.000	ITGB1	12.556	9	0	0	0.83257	FALSE	0	ITGB1	4016	0.1714
151	2.6781	0.0143	0.3734	0.1944	X	9	5	FALSE	Cluster 3	Unclustered	3.000	GSN	12.000	9	0	0	0.81355	FALSE	0	GSN	2110	0.2162
74	2.8082	0.0007	0.3561	0.6786	UP	8	7	FALSE	Cluster 3	Clustered	4.762	PSMD11	13.875	8	0	0	0.79909	FALSE	0	PSMD11	98	0.2523
80	2.5548	0.0034	0.3914	0.5714	UP	8	7	FALSE	Cluster 3	Clustered	4.762	PSMD10	17.750	8	0	0	0.82725	FALSE	0	PSMD10	564	0.2289
84	2.5274	0.0057	0.3957	0.5714	UP	8	7	FALSE	Cluster 3	Clustered	4.762	PSMD3	18.000	8	0	0	0.83029	FALSE	0	PSMD3	854	0.2207
97	2.9795	0.0061	0.3356	0.4286	UP	8	7	FALSE	Cluster 3|Cluster 2	Clustered	5.000	GNAI3	7.875	8	0	0	0.78006	FALSE	0	GNAI3	736	0.2583
101	2.9110	0.0081	0.3435	0.4286	UP	8	7	FALSE	Cluster 3|Cluster 2	Clustered	5.000	GNAI2	7.750	8	0	0	0.78767	FALSE	0	GNAI2	920	0.2460

124	2.7123	0.0025	0.3687	0.6429	UP	8	7	FALSE	Cluster 2	Clustered	6.000	PCBP2	18.625	8	0	0	0.80974	FALSE	0	PCBP2	304	0.2891
135	2.4932	0.0162	0.4011	0.3214	UP	8	6	FALSE	Cluster 3	Unclustered	1.714	LMNB1	20.000	8	0	0	0.83409	FALSE	0	LMNB1	1698	0.2424
138	2.5342	0.0186	0.3946	0.3214	UP	8	6	FALSE	Cluster 3	Clustered	4.000	GJA1	18.375	8	0	0	0.82953	FALSE	0	GJA1	1764	0.2433
178	2.6233	0.0033	0.3812	0.7143	UP	8	5	FALSE	Cluster 2	Clustered	6.000	PGD	20.125	8	0	0	0.81963	FALSE	0	PGD	386	0.3096
75	2.8356	0.0010	0.3527	0.6190	UP	7	7	FALSE	Cluster 3	Seed	5.000	ADRM1	13.286	7	0	0	0.79604	FALSE	0	ADRM1	114	0.2507
94	2.6712	0.0000	0.3744	0.9048	UP	7	6	FALSE	Cluster 2	Clustered	6.000	ALDOA	22.429	7	0	0	0.81431	FALSE	0	ALDOA	8	0.3560
104	3.0479	0.0055	0.3281	0.4762	DOWN	7	7	FALSE	Cluster 3|Cluster 2	Clustered	5.000	GNG12	8.571	7	0	0	0.77245	FALSE	0	GNG12	572	0.2511
130	2.7740	0.0189	0.3605	0.0952	DOWN	7	6	FALSE	Cluster 3	Unclustered	1.667	MYH10	10.714	7	0	0	0.80289	FALSE	0	MYH10	1324	0.2113
139	2.6027	0.0348	0.3842	0.0952	UP	7	7	FALSE	Cluster 3	Unclustered	1.200	FGR	14.571	7	0	0	0.82192	FALSE	0	FGR	2828	0.1964
173	2.5616	0.0001	0.3904	0.9524	UP	7	6	FALSE		Unclustered	5.786	NADSYN1	30.571	7	0	0	0.82648	FALSE	0	NADSYN1	48	0.4076
187	2.6575	0.0581	0.3763	0.1429	DOWN	7	5	FALSE	Cluster 3	Unclustered	1.067	MSN	12.429	7	0	0	0.81583	FALSE	0	MSN	4874	0.2291
191	2.6507	0.0032	0.3773	0.2857	UP	7	7	FALSE		Unclustered	3.000	SET	17.143	7	0	0	0.81659	FALSE	0	SET	384	0.2537
134	2.8562	0.0072	0.3501	0.2667	DOWN	6	7	FALSE	Cluster 3	Unclustered	3.000	LMNA	13.000	6	0	0	0.79376	FALSE	0	LMNA	560	0.2390
136	2.6849	0.0066	0.3724	0.2667	UP	6	7	FALSE		Unclustered	3.000	MIB1	20.500	6	0	0	0.81279	FALSE	0	MIB1	828	0.3010
137	3.0068	0.0148	0.3326	0.1333	UP	6	7	FALSE	Cluster 3	Unclustered	1.667	CTNND1	6.000	6	0	0	0.77702	FALSE	0	CTNND1	1454	0.2200
143	2.9041	0.0002	0.3443	0.7333	UP	6	7	FALSE	Cluster 5	Clustered	3.238	ATP6V1A	17.000	6	0	0	0.78843	FALSE	0	ATP6V1A	54	0.3696
150	2.7466	0.0013	0.3641	0.4667	DOWN	6	6	FALSE	Cluster 3	Clustered	4.000	IDE	15.833	6	0	0	0.80594	FALSE	0	IDE	154	0.3013
183	2.5822	0.0008	0.3873	0.5333	UP	6	6	FALSE	Cluster 4	Seed	2.400	APAF1	23.000	6	0	0	0.8242	FALSE	0	APAF1	172	0.3108
198	3.0616	0.0132	0.3266	0.0000	UP	6	5	FALSE	Cluster 3	Unclustered	0.286	RAB25	5.833	6	0	0	0.77093	FALSE	0	RAB25	880	0.2101
199	2.6781	0.0137	0.3734	0.6000	UP	6	7	FALSE		Unclustered	3.733	LAP3	22.167	6	0	0	0.81355	FALSE	0	LAP3	1160	0.3492

98	3.3767	0.0000	0.2961	1.0000	DOWN	5	7	FALSE	Cluster 3|Cluster 2	Clustered	5.000	GNB2	7.600	5	0	0	0.73592	FALSE	0	GNB2	0	0.4750
103	3.3767	0.0000	0.2961	1.0000	DOWN	5	7	FALSE	Cluster 3|Cluster 2	Clustered	5.000	GNG5	7.600	5	0	0	0.73592	FALSE	0	GNG5	0	0.4750
126	3.1644	0.0288	0.3160	0.0000	UP	5	6	FALSE	Cluster 3	Unclustered	0.400	STX4	4.800	5	0	0	0.75951	FALSE	0	STX4	2370	0.2111
145	2.6644	0.0010	0.3753	0.5000	UP	5	6	FALSE		Unclustered	2.400	PFN1	20.200	5	0	0	0.81507	FALSE	0	PFN1	164	0.3206
210	2.8973	0.0005	0.3452	0.5000	UP	5	7	FALSE	Cluster 5	Clustered	3.000	ATP1A1	16.200	5	0	0	0.78919	FALSE	0	ATP1A1	92	0.3375
121	3.0616	0.0074	0.3266	0.3333	DOWN	4	7	FALSE	Cluster 3	Unclustered	1.667	ANK1	8.000	4	0	0	0.77093	FALSE	0	ANK1	792	0.2981
128	3.1712	0.0155	0.3153	0.0000	UP	4	7	FALSE		Unclustered	0.400	PLD1	10.250	4	0	0	0.75875	FALSE	0	PLD1	2454	0.4625
152	3.4247	0.0032	0.2920	0.0000	DOWN	4	6	FALSE	Cluster 3	Unclustered	0.400	PIP5K1A	6.250	4	0	0	0.73059	FALSE	0	PIP5K1A	284	0.3088
180	2.9521	0.0075	0.3387	0.5000	UP	4	6	FALSE	Cluster 3	Unclustered	1.400	DSP	9.250	4	0	0	0.78311	FALSE	0	DSP	658	0.3190
190	2.8082	0.0017	0.3561	0.5000	UP	4	7	FALSE		Unclustered	1.400	TRIP13	21.000	4	0	0	0.79909	FALSE	0	TRIP13	258	0.3500
197	2.6986	0.0009	0.3706	0.5000	UP	4	7	FALSE		Unclustered	1.400	FASN	21.000	4	0	0	0.81126	FALSE	0	FASN	94	0.3443
202	2.7123	0.0137	0.3687	0.5000	UP	4	7	FALSE		Unclustered	3.000	CDA	25.500	4	0	0	0.80974	FALSE	0	CDA	964	0.4208
204	3.0411	0.0000	0.3288	0.8333	UP	4	7	FALSE		Unclustered	2.700	DNAH2	15.750	4	0	0	0.77321	FALSE	0	DNAH2	2	0.5625
90	3.0137	0.0000	0.3318	1.0000	UP	3	7	FALSE		Unclustered	3.000	XRCC5	17.000	3	0	0	0.77626	FALSE	0	XRCC5	0	0.4595
96	3.2534	0.0001	0.3074	0.6667	DOWN	3	7	FALSE		Unclustered	1.667	YOD1	9.333	3	0	0	0.74962	FALSE	0	YOD1	14	0.4667
131	2.9178	0.0000	0.3427	1.0000	DOWN	3	7	FALSE		Unclustered	3.000	RBCK1	22.000	3	0	0	0.78691	FALSE	0	RBCK1	0	0.4400
132	2.6507	0.0001	0.3773	0.6667	UP	3	6	FALSE		Unclustered	1.667	UCHL1	34.000	3	0	0	0.81659	FALSE	0	UCHL1	18	0.5075
161	2.9589	0.0003	0.3380	0.0000	UP	3	7	FALSE		Unclustered	0.500	ZNF259	17.333	3	0	0	0.78234	FALSE	0	ZNF259	44	0.3984
165	3.3630	0.0139	0.2974	0.0000	DOWN	3	8	FALSE		Unclustered	0.500	LAPTM5	6.667	3	0	0	0.73744	FALSE	0	LAPTM5	1086	0.3542
167	2.9658	0.0000	0.3372	1.0000	UP	3	7	FALSE	Cluster 3	Unclustered	3.000	DDI2	19.667	3	0	0	0.78158	FALSE	0	DDI2	0	0.4470

174	2.9863	0.0137	0.3349	0.3333	DOWN	3	6	FALSE		Unclustered	2.000	AHNAK	14.333	3	0	0	0.7793	FALSE	0	AHNAK	1234	0.4000
179	3.0479	0.0003	0.3281	0.0000	UP	3	7	FALSE		Unclustered	0.500	MYO1E	16.667	3	0	0	0.77245	FALSE	0	MYO1E	98	0.5595
185	3.1233	0.0008	0.3202	0.0000	UP	3	6	FALSE		Unclustered	0.500	MYO1G	14.000	3	0	0	0.76408	FALSE	0	MYO1G	140	0.5909
193	3.1712	0.0004	0.3153	0.3333	UP	3	7	FALSE		Unclustered	2.000	BCAP31	12.333	3	0	0	0.75875	FALSE	0	BCAP31	64	0.4800
194	2.9658	0.0001	0.3372	0.3333	UP	3	7	FALSE		Unclustered	2.000	ANP32E	16.333	3	0	0	0.78158	FALSE	0	ANP32E	14	0.4211
201	2.9247	0.0277	0.3419	0.0000	DOWN	3	6	FALSE		Unclustered	0.500	S100A4	10.000	3	0	0	0.78615	FALSE	0	S100A4	1300	0.3333
216	2.7123	0.0002	0.3687	0.3333	DOWN	3	7	FALSE		Unclustered	2.000	SLC2A3	30.333	3	0	0	0.80974	FALSE	0	SLC2A3	56	0.4918
218	3.2603	0.0000	0.3067	1.0000	DOWN	3	7	FALSE	Cluster 3	Unclustered	3.000	TOR1AIP1	9.000	3	0	0	0.74886	FALSE	0	TOR1AIP1	0	0.4737
111	2.9452	0.0000	0.3395	1.0000	DOWN	2	7	FALSE		Unclustered	2.000	HIST2H2AB	26.500	2	0	0	0.78387	FALSE	0	HIST2H2AB	0	0.5889
149	3.7055	0.0000	0.2699	1.0000	X	2	8	FALSE	Cluster 3	Unclustered	2.000	COPA	6.500	2	0	0	0.69939	FALSE	0	COPA	0	0.6500
153	3.3836	0.0001	0.2955	0.0000	DOWN	2	7	FALSE		Unclustered	0.667	HIST1H1C	10.000	2	0	0	0.73516	FALSE	0	HIST1H1C	12	0.6429
157	3.7055	0.0000	0.2699	1.0000	DOWN	2	8	FALSE	Cluster 3	Unclustered	2.000	SCN7A	6.500	2	0	0	0.69939	FALSE	0	SCN7A	0	0.6500
163	3.3288	0.0003	0.3004	0.0000	DOWN	2	7	FALSE	Cluster 3	Unclustered	0.667	GJA5	7.000	2	0	0	0.74125	FALSE	0	GJA5	22	0.5000
172	5.5548	0.0137	0.1800	0.0000	UP	2	8	FALSE		Unclustered	0.667	AKR1C2	1.500	2	0	0	0.49391	FALSE	0	AKR1C2	1082	0.5000
181	3.8082	0.0000	0.2626	1.0000	UP	2	7	FALSE	Cluster 3	Unclustered	2.000	DSG2	5.000	2	0	0	0.68798	FALSE	0	DSG2	0	0.7143
186	3.6096	0.0405	0.2770	0.0000	UP	2	6	FALSE		Unclustered	0.667	NCF4	4.500	2	0	0	0.71005	FALSE	0	NCF4	3234	0.5000
188	3.9041	0.0137	0.2561	0.0000	DOWN	2	7	FALSE		Unclustered	0.667	SELENBP1	2.000	2	0	0	0.67732	FALSE	0	SELENBP1	622	0.5000
192	3.1027	0.0000	0.3223	1.0000	UP	2	7	FALSE		Unclustered	2.000	PTMA	17.500	2	0	0	0.76636	FALSE	0	PTMA	0	0.5833
195	3.1233	0.0003	0.3202	0.0000	UP	2	7	FALSE		Unclustered	0.667	SLC7A5	16.000	2	0	0	0.76408	FALSE	0	SLC7A5	24	0.5172
196	3.0616	0.0005	0.3266	0.0000	UP	2	7	FALSE		Unclustered	0.667	SLC12A2	18.500	2	0	0	0.77093	FALSE	0	SLC12A2	96	0.5147

200	3.0342	0.0000	0.3296	1.0000	UP	2	6	FALSE	Unclustered	2.000	CALD1	15.000	2	0	0	0.77397	FALSE	0	CALD1	0	0.6000
203	2.7740	0.0003	0.3605	0.0000	X	2	7	FALSE	Unclustered	0.667	S100A9	29.500	2	0	0	0.80289	FALSE	0	S100A9	62	0.5182
208	2.7945	0.0003	0.3578	0.0000	DOWN	2	7	FALSE	Unclustered	0.667	CA2	29.000	2	0	0	0.80061	FALSE	0	CA2	64	0.5283
209	2.8288	0.0012	0.3535	0.0000	UP	2	7	FALSE	Unclustered	0.667	SLC1A5	25.500	2	0	0	0.7968	FALSE	0	SLC1A5	134	0.5104
212	2.9932	0.0000	0.3341	1.0000	UP	2	6	FALSE	Unclustered	2.000	AKR1B10	20.500	2	0	0	0.77854	FALSE	0	AKR1B10	0	0.5541
213	2.7534	0.0005	0.3632	0.0000	UP	2	7	FALSE	Unclustered	0.667	NOTCH3	30.500	2	0	0	0.80518	FALSE	0	NOTCH3	116	0.5364
220	1.0000	1.0000	1.0000	0.0000	UP	2	1	FALSE	Unclustered	0.667	WDR44	1.000	2	0	0	1	FALSE	0	WDR44	2	0.0000
221	4.5753	0.0272	0.2186	0.0000	UP	2	7	FALSE	Unclustered	0.667	SNX3	2.000	2	0	0	0.60274	FALSE	0	SNX3	2160	0.5000
222	3.0822	0.0001	0.3244	0.0000	UP	2	7	FALSE	Unclustered	0.667	CBR1	21.500	2	0	0	0.76865	FALSE	0	CBR1	20	0.6029
127	4.1644	0.0000	0.2401	0.0000	DOWN	1	8	FALSE	Unclustered	0.000	PPAP2B	4.000	1	0	0	0.6484	FALSE	0	PPAP2B	0	0.0000
133	4.1575	0.0000	0.2405	0.0000	DOWN	1	7	FALSE	Unclustered	0.000	VAMP5	5.000	1	0	0	0.64916	FALSE	0	VAMP5	0	0.0000
140	3.5959	0.0000	0.2781	0.0000	UP	1	8	FALSE	Unclustered	0.000	EFNB1	7.000	1	0	0	0.71157	FALSE	0	EFNB1	0	0.0000
147	3.5000	0.0000	0.2857	0.0000	DOWN	1	7	FALSE	Unclustered	0.000	COL6A1	9.000	1	0	0	0.72222	FALSE	0	COL6A1	0	0.0000
154	3.2808	0.0000	0.3048	0.0000	DOWN	1	7	FALSE	Unclustered	0.000	INTS7	27.000	1	0	0	0.74658	FALSE	0	INTS7	0	0.0000
155	3.0000	0.0000	0.3333	0.0000	UP	1	7	FALSE	Unclustered	0.000	FCHO2	43.000	1	0	0	0.77778	FALSE	0	FCHO2	0	0.0000
158	1.0000	0.0000	1.0000	0.0000	DOWN	1	1	FALSE	Unclustered	0.000	CLIC2	1.000	1	0	0	1	FALSE	0	CLIC2	0	0.0000
159	1.0000	0.0000	1.0000	0.0000	DOWN	1	1	FALSE	Unclustered	0.000	CLIC5	1.000	1	0	0	1	FALSE	0	CLIC5	0	0.0000
160	3.5616	0.0000	0.2808	0.0000	UP	1	7	FALSE	Unclustered	0.000	S100A11	10.000	1	0	0	0.71537	FALSE	0	S100A11	0	0.0000
164	3.6233	0.0000	0.2760	0.0000	UP	1	8	FALSE	Unclustered	0.000	DCUN1D1	10.000	1	0	0	0.70852	FALSE	0	DCUN1D1	0	0.0000
166	4.3562	0.0000	0.2296	0.0000	UP	1	9	FALSE	Unclustered	0.000	LAPTM4A	3.000	1	0	0	0.62709	FALSE	0	LAPTM4A	0	0.0000

168	1.0000	0.0000	1.0000	0.0000	DOWN	1	1	FALSE	Unclustered	0.000	ESAM	1.000	1	0	0	1	FALSE	0	ESAM	0	0.0000
169	1.0000	0.0000	1.0000	0.0000	DOWN	1	1	FALSE	Unclustered	0.000	CLDN18	1.000	1	0	0	1	FALSE	0	CLDN18	0	0.0000
170	3.4521	0.0000	0.2897	0.0000	UP	1	7	FALSE	Unclustered	0.000	TELO2	12.000	1	0	0	0.72755	FALSE	0	TELO2	0	0.0000
171	6.5479	0.0000	0.1527	0.0000	UP	1	9	FALSE	Unclustered	0.000	AKR1C1	2.000	1	0	0	0.38356	FALSE	0	AKR1C1	0	0.0000
175	3.9795	0.0000	0.2513	0.0000	DOWN	1	7	FALSE	Unclustered	0.000	MYOF	3.000	1	0	0	0.66895	FALSE	0	MYOF	0	0.0000
177	3.5000	0.0000	0.2857	0.0000	UP	1	7	FALSE	Unclustered	0.000	ALCAM	9.000	1	0	0	0.72222	FALSE	0	ALCAM	0	0.0000
189	4.8973	0.0000	0.2042	0.0000	DOWN	1	8	FALSE	Unclustered	0.000	CA1	2.000	1	0	0	0.56697	FALSE	0	CA1	0	0.0000
205	3.1233	0.0000	0.3202	0.0000	UP	1	7	FALSE	Unclustered	0.000	WRNIP1	28.000	1	0	0	0.76408	FALSE	0	WRNIP1	0	0.0000
206	3.1233	0.0000	0.3202	0.0000	UP	1	7	FALSE	Unclustered	0.000	CBX1	28.000	1	0	0	0.76408	FALSE	0	CBX1	0	0.0000
207	3.6712	0.0000	0.2724	0.0000	UP	1	8	FALSE	Unclustered	0.000	PLSCR1	6.000	1	0	0	0.7032	FALSE	0	PLSCR1	0	0.0000
211	3.7055	0.0000	0.2699	0.0000	UP	1	8	FALSE	Unclustered	0.000	SLC29A1	4.000	1	0	0	0.69939	FALSE	0	SLC29A1	0	0.0000
214	1.0000	0.0000	1.0000	0.0000	DOWN	1	1	FALSE	Unclustered	0.000	SLC34A2	1.000	1	0	0	1	FALSE	0	SLC34A2	0	0.0000
215	1.0000	0.0000	1.0000	0.0000	DOWN	1	1	FALSE	Unclustered	0.000	SFTPC	1.000	1	0	0	1	FALSE	0	SFTPC	0	0.0000
217	3.3014	0.0000	0.3029	0.0000	UP	1	7	FALSE	Unclustered	0.000	NDUFA8	21.000	1	0	0	0.74429	FALSE	0	NDUFA8	0	0.0000
219	1.5000	0.0000	0.6667	0.0000	UP	1	2	FALSE	Unclustered	0.000	CHIC1	2.000	1	0	0	0.75	FALSE	0	CHIC1	0	0.0000
223	1.5000	0.0000	0.6667	0.0000	UP	1	2	FALSE	Unclustered	0.000	ZNF280C	2.000	1	0	0	0.75	FALSE	0	ZNF280C	0	0.0000
224	3.3425	0.0000	0.2992	0.0000	DOWN	1	7	FALSE	Unclustered	0.000	HSD17B11	22.000	1	0	0	0.73973	FALSE	0	HSD17B11	0	0.0000
225	3.5000	0.0000	0.2857	0.0000	UP	1	7	FALSE	Unclustered	0.000	EPPK1	9.000	1	0	0	0.72222	FALSE	0	EPPK1	0	0.0000
