# Supplementary material for: Label-free quantitative identification of abnormally ubiquitinated proteins as useful biomarkers for human lung squamous cell carcinomas
Source: EPMA J. 2020 Jan 4;11(1):73–94. doi: 10.1007/s13167-019-00197-8 (PMC7028901; doi:10.1007/s13167-019-00197-8)
Supplement: Supplementary file 6 — (PDF 75 kb) [file 13167_2019_197_MOESM6_ESM.pdf]

Supplemental Table 4. Co-expressed genes of VIM based on TCGA database of human LSCC.

Correlated Gene	Cytoband	Spearman's Correlation	p-Value	q-Value
ANXA6	5q33.1	0.81	5.61E-120	1.13E-115
RFTN1	3p24.3	0.81	1.85E-119	1.87E-115
PMP22	17p12	0.80	7.49E-113	5.04E-109
BGN	Xq28	0.80	5.40E-111	2.50E-107
ATP10A	15q12	0.80	6.20E-111	2.50E-107
FN1	2q35	0.79	1.89E-108	6.34E-105
CNRIP1	2p14	0.79	2.28E-108	6.56E-105
ZEB2	2q22.3	0.79	4.89E-107	1.23E-103
OLFML1	11p15.4	0.79	2.86E-106	6.41E-103
OLFML2B	1q23.3	0.78	3.55E-105	7.16E-102
TRPV2	17p11.2	0.78	2.12E-102	3.89E-99
SPARC	5q33.1	0.78	2.55E-102	4.29E-99
GASK1B	4q32.1	0.78	6.15E-102	9.54E-99
EMP3	19q13.33	0.78	8.06E-102	1.16E-98
ZCCHC24	10q22.3	0.77	1.62E-101	2.18E-98
STARD13	13q13.1-q13.[illegible]	0.77	8.84E-101	1.11E-97
TIMP2	17q25.3	0.77	3.68E-99	4.37E-96
FMOD	1q32.1	0.77	1.25E-98	1.40E-95
GNAI2	3p21.31	0.77	1.55E-98	1.64E-95
TSPAN4	11p15.5	0.77	4.03E-98	4.06E-95
PDGFRB	5q32	0.77	7.41E-98	7.12E-95
COLEC12	18p11.32	0.76	8.48E-97	7.78E-94
TMEM119	12q23.3	0.76	2.10E-94	1.84E-91
SERPING1	11q12.1	0.75	1.89E-93	1.59E-90
ZNF521	18q11.2	0.75	2.19E-93	1.77E-90
EMILIN1	2p23.3	0.75	2.57E-93	1.99E-90
THY1	11q23.3	0.75	8.03E-93	6.00E-90
ADGRA2	8p11.23	0.75	2.16E-92	1.56E-89
ETS1	11q24.3	0.75	3.22E-92	2.24E-89
LAMA4	6q21	0.75	4.26E-91	2.86E-88
ACVRL1	12q13.13	0.75	5.55E-91	3.61E-88
ANGPTL2	9q33.3	0.75	1.16E-90	7.18E-88
RASGRF2	5q14.1	0.75	1.17E-90	7.18E-88
NID2	14q22.1	0.75	1.40E-90	8.32E-88
COL6A2	21q22.3	0.75	2.41E-90	1.39E-87
FBN1	15q21.1	0.75	2.77E-90	1.55E-87
COL6A3	2q37.3	0.74	8.87E-90	4.84E-87
FLI1	11q24.3	0.74	1.02E-89	5.39E-87
BICC1	10q21.1	0.74	3.08E-89	1.59E-86
RHOJ	14q23.2	0.74	3.28E-88	1.65E-85
FMNL3	12q13.12	0.74	2.74E-87	1.35E-84
HEPH	Xq12	0.74	5.82E-87	2.80E-84
PCOLCE	7q22.1	0.74	6.51E-87	3.05E-84
ISLR	15q24.1	0.74	7.25E-87	3.32E-84
GNG2	14q22.1	0.74	8.63E-87	3.87E-84
GGT5	22q11.23	0.74	1.23E-86	5.41E-84
ITGA1	5q11.2	0.73	3.78E-86	1.62E-83
TNS3	7p12.3	0.73	4.83E-86	2.03E-83
OLFML3	1p13.2	0.73	5.48E-86	2.26E-83

COL1A2	7q21.3	0.73	9.66E-86	3.90E-83
PALM2-AKAP2	9q31.3	0.73	1.07E-85	4.22E-83
FILIP1L	3q12.1	0.73	1.10E-85	4.26E-83
RCAN2	6p12.3	0.73	2.28E-85	8.69E-83
DACT1	14q23.1	0.73	3.27E-85	1.22E-82
WIPF1	2q31.1	0.73	3.85E-85	1.41E-82
UNC5C	4q22.3	0.73	5.83E-85	2.10E-82
MEF2C	5q14.3	0.73	8.22E-85	2.91E-82
STARD8	Xq13.1	0.73	8.41E-85	2.93E-82
JCAD	10p11.23	0.73	1.06E-84	3.62E-82
POSTN	13q13.3	0.73	1.16E-84	3.91E-82
ACTA2	10q23.31	0.73	1.86E-84	6.16E-82
TMEM204	16p13.3	0.73	3.14E-84	1.02E-81
COL8A1	3q12.1	0.73	7.41E-84	2.37E-81
CLMP	11q24.1	0.73	8.52E-84	2.69E-81
MICAL2	11p15.3	0.73	1.95E-83	6.05E-81
FGD5	3p25.1	0.73	2.15E-83	6.56E-81
PTGIR	19q13.32	0.73	3.19E-83	9.59E-81
THBS2	6q27	0.73	4.65E-83	1.38E-80
CDH11	16q21	0.73	5.01E-83	1.46E-80
KCNMB1	5q35.1	0.73	5.59E-83	1.61E-80
COL3A1	2q32.2	0.72	7.85E-83	2.23E-80
DACT3	19q13.32	0.72	1.36E-82	3.80E-80
LRRC32	11q13.5	0.72	1.62E-82	4.49E-80
AEBP1	7p13	0.72	1.71E-82	4.65E-80
CD4	12p13.31	0.72	1.73E-82	4.65E-80
TAGLN	11q23.3	0.72	3.03E-82	8.04E-80
BNC2	9p22.3-p22.2	0.72	6.18E-82	1.62E-79
CRISPLD2	16q24.1	0.72	6.66E-82	1.72E-79
MRGPRF	11q13.3	0.72	6.85E-82	1.75E-79
CSF1R	5q32	0.72	8.42E-82	2.12E-79
FAP	2q24.2	0.72	1.59E-81	3.95E-79
AXL	19q13.2	0.72	2.04E-81	5.03E-79
ZFPM2	8q23.1	0.72	2.45E-81	5.95E-79
MFRP	11q23.3	0.72	3.03E-81	7.28E-79
ENG	9q34.11	0.72	3.67E-81	8.71E-79
PLEKHO2	15q22.31	0.72	4.11E-81	9.64E-79
CALD1	7q33	0.72	4.42E-81	1.02E-78
GPC6	13q31.3-q32.	0.72	7.32E-81	1.68E-78
MITF	3p13	0.72	9.02E-81	2.04E-78
TIMP3	22q12.3	0.72	9.64E-81	2.16E-78
EDNRA	4q31.22-q31.[illegible]	0.72	1.55E-80	3.43E-78
PLXNC1	12q22	0.72	2.53E-80	5.55E-78
GLT8D2	12q23.3	0.72	2.74E-80	5.94E-78
JAM2	21q21.3	0.72	3.28E-80	7.04E-78
PTGER2	14q22.1	0.72	1.02E-79	2.17E-77
KANK2	19p13.2	0.72	1.18E-79	2.47E-77
MXRA8	1p36.33	0.71	1.51E-79	3.13E-77
CD248	11q13.2	0.71	1.28E-78	2.63E-76
LHFPL2	5q14.1	0.71	1.74E-78	3.55E-76
PODN	1p32.3	0.71	3.29E-78	6.63E-76
ANTXR2	4q21.21	0.71	4.33E-78	8.65E-76
NNMT	11q23.2	0.71	7.12E-78	1.41E-75
IL7R	5p13.2	0.71	1.08E-77	2.11E-75

SLC7A7	14q11.2	0.71	1.17E-77	2.26E-75
PCDHGA12	5q31.3	0.71	1.42E-77	2.73E-75
AKAP2	9q31.3	0.71	1.92E-77	3.66E-75
ECM2	9q22.31	0.71	3.34E-77	6.30E-75
SELPLG	12q24.11	0.71	6.12E-77	1.14E-74
FBLN5	14q32.12	0.71	9.21E-77	1.70E-74
SH2B3	12q24.12	0.70	3.52E-76	6.45E-74
SPON1	11p15.2	0.70	3.57E-76	6.48E-74
GAS7	17p13.1	0.70	4.27E-76	7.69E-74
GFPT2	5q35.3	0.70	6.57E-76	1.17E-73
CCN2	6q23.2	0.70	8.73E-76	1.55E-73
ADAM19	5q33.3	0.70	9.54E-76	1.67E-73
LGALS1	22q13.1	0.70	1.17E-75	2.03E-73
FCGR2A	1q23.3	0.70	1.20E-75	2.08E-73
COL5A1	9q34.3	0.70	1.26E-75	2.15E-73
GYPC	2q14.3	0.70	4.24E-75	7.20E-73
CYP1B1	2p22.2	0.70	5.06E-75	8.52E-73
OSCAR	19q13.42	0.70	1.01E-74	1.68E-72
CD93	20p11.21	0.70	1.22E-74	2.02E-72
LHFPL6	13q13.3-q14.	0.70	1.51E-74	2.47E-72
KIAA1755	20q11.23	0.70	1.64E-74	2.68E-72
NLRP3	1q44	0.70	1.73E-74	2.79E-72
COL1A1	17q21.33	0.70	1.96E-74	3.13E-72
SPI1	11p11.2	0.70	2.44E-74	3.87E-72
DOCK11	Xq24	0.70	3.79E-74	5.97E-72
LAPTM5	1p35.2	0.70	6.01E-74	9.40E-72
FBLN2	3p25.1	0.70	6.17E-74	9.58E-72
RUBCNL	13q14.13	0.70	6.59E-74	1.01E-71
SNED1	2q37.3	0.70	6.70E-74	1.02E-71
CD300C	17q25.1	0.70	8.78E-74	1.33E-71
KCTD12	13q22.3	0.70	1.00E-73	1.51E-71
MMP2	16q12.2	0.70	1.16E-73	1.73E-71
TGFBR2	3p24.1	0.70	1.17E-73	1.73E-71
CTSK	1q21.3	0.70	1.46E-73	2.15E-71
PLA2G5	1p36.13	0.69	1.78E-73	2.59E-71
SFXN3	10q24.31	0.69	1.79E-73	2.59E-71
MRVI1	11p15.4	0.69	1.89E-73	2.72E-71
NEXN	1p31.1	0.69	1.96E-73	2.80E-71
SIGLEC9	19q13.41	0.69	3.68E-73	5.23E-71
ROR1	1p31.3	0.69	5.62E-73	7.93E-71
GEM	8q22.1	0.69	6.38E-73	8.94E-71
ITGB2	21q22.3	0.69	6.52E-73	9.08E-71
MIR100HG	11q24.1	0.69	7.32E-73	1.01E-70
DAB2	5p13.1	0.69	8.92E-73	1.22E-70
SLAMF8	1q23.2	0.69	1.00E-72	1.36E-70
PDE1B	12q13.2	0.69	1.13E-72	1.52E-70
TMEM176A	7q36.1	0.69	1.13E-72	1.52E-70
DOK5	20q13.2	0.69	2.37E-72	3.17E-70
MSRB3	12q14.3	0.69	2.41E-72	3.21E-70
SYNE1	6q25.2	0.69	2.59E-72	3.41E-70
VSTM4	10q11.23	0.69	3.26E-72	4.27E-70
RASL12	15q22.31	0.69	4.09E-72	5.32E-70
C1QTNF1	17q25.3	0.69	7.71E-72	9.97E-70
ITGAM	16p11.2	0.69	8.32E-72	1.07E-69

TNFRSF1B	1p36.22	0.69	1.54E-71	1.96E-69
CD14	5q31.3	0.69	2.00E-71	2.53E-69
VGLL3	3p12.1	0.69	2.51E-71	3.17E-69
SSC5D	19q13.42	0.69	2.81E-71	3.52E-69
IL3RA	Xp22.33 and `	0.69	3.08E-71	3.84E-69
ADAMTS2	5q35.3	0.69	5.87E-71	7.27E-69
PDZRN3	3p13	0.69	7.22E-71	8.88E-69
LTBP2	14q24.3	0.68	1.52E-70	1.85E-68
ITGB3	17q21.32	0.68	1.90E-70	2.31E-68
INHBA	7p14.1	0.68	2.00E-70	2.41E-68
VCAM1	1p21.2	0.68	2.10E-70	2.52E-68
TMEM176B	7q36.1	0.68	2.62E-70	3.13E-68
CPXM1	20p13	0.68	2.94E-70	3.49E-68
NAV3	12q21.2	0.68	3.22E-70	3.80E-68
HIC1	17p13.3	0.68	4.19E-70	4.91E-68
THEMIS2	1p35.3	0.68	5.35E-70	6.24E-68
MEOX2	7p21.2	0.68	7.68E-70	8.90E-68
ITGBL1	13q33.1	0.68	1.15E-69	1.33E-67
FCGR2B	1q23.3	0.68	1.64E-69	1.88E-67
CYTH4	22q13.1	0.68	2.44E-69	2.78E-67
LMOD1	1q32.1	0.68	2.86E-69	3.24E-67
RASGRP3	2p22.3	0.68	3.04E-69	3.43E-67
VCAN	5q14.2-q14.3	0.68	5.56E-69	6.23E-67
C3AR1	12p13.31	0.68	1.05E-68	1.17E-66
PGR	11q22.1	0.68	1.09E-68	1.21E-66
CSGALNACT2	10q11.21	0.68	1.47E-68	1.62E-66
CLEC11A	19q13.33	0.68	1.48E-68	1.62E-66
TGFB3	14q24.3	0.68	1.50E-68	1.63E-66
TBXA2R	19p13.3	0.68	1.59E-68	1.73E-66
DLC1	8p22	0.68	3.25E-68	3.51E-66
COL6A1	21q22.3	0.68	5.81E-68	6.24E-66
DAAM2	6p21.2	0.68	6.48E-68	6.92E-66
RASGRP4	19q13.2	0.67	6.75E-68	7.16E-66
COL5A2	2q32.2	0.67	9.96E-68	1.05E-65
ST3GAL2	16q22.1	0.67	1.24E-67	1.30E-65
LDB2	4p15.32	0.67	1.27E-67	1.33E-65
ARHGAP31	3q13.32-q13.:	0.67	1.62E-67	1.69E-65
SMIM3	5q33.1	0.67	2.18E-67	2.26E-65
FPR3	19q13.41	0.67	3.49E-67	3.59E-65
GLIPR1	12q21.2	0.67	3.58E-67	3.67E-65
RUNX1T1	8q21.3	0.67	4.05E-67	4.13E-65
LOXL2	8p21.3	0.67	7.56E-67	7.67E-65
DOCK2	5q35.1	0.67	1.79E-66	1.81E-64
FCGR2C	1q23.3	0.67	2.48E-66	2.49E-64
PILRA	7q22.1	0.67	2.75E-66	2.75E-64
LAIR1	19q13.42	0.67	2.95E-66	2.93E-64
PECAM1	17q23.3	0.67	3.77E-66	3.73E-64
CHN1	2q31.1	0.67	4.39E-66	4.32E-64
COL15A1	9q22.33	0.67	4.65E-66	4.55E-64
IGFBP7	4q12	0.67	5.19E-66	5.06E-64
ALOX5AP	13q12.3	0.67	5.48E-66	5.31E-64
VEGFC	4q34.3	0.67	6.41E-66	6.19E-64
AOC3	17q21.31	0.67	8.75E-66	8.41E-64
MYL9	20q11.23	0.67	1.19E-65	1.14E-63

DCHS1	11p15.4	0.67	1.57E-65	1.49E-63
GIPC3	19p13.3	0.67	1.76E-65	1.67E-63
SMAD7	18q21.1	0.67	2.14E-65	2.02E-63
PNMA2	8p21.2	0.67	2.18E-65	2.05E-63
SLCO2B1	11q13.4	0.66	3.02E-65	2.82E-63
LRRC25	19p13.11	0.66	3.09E-65	2.87E-63
CPZ	4p16.1	0.66	3.62E-65	3.35E-63
CMTM3	16q22.1	0.66	3.84E-65	3.54E-63
CD74	5q33.1	0.66	4.12E-65	3.78E-63
GPR183	13q32.3	0.66	4.26E-65	3.89E-63
TGFB1I1	16p11.2	0.66	4.45E-65	4.05E-63
CYBB	Xp21.1-p11.4	0.66	6.46E-65	5.85E-63
ARHGEF6	Xq26.3	0.66	7.60E-65	6.84E-63
DDR2	1q23.3	0.66	9.05E-65	8.12E-63
RASSF2	20p13	0.66	1.14E-64	1.02E-62
EVI2A	17q11.2	0.66	1.63E-64	1.45E-62
CD33	19q13.41	0.66	2.50E-64	2.21E-62
MFAP4	17p11.2	0.66	2.93E-64	2.58E-62
ADGRL4	1p31.1	0.66	3.15E-64	2.76E-62
MYADM	19q13.42	0.66	3.62E-64	3.17E-62
GPSM3	6p21.32	0.66	4.37E-64	3.80E-62
P4HA3	11q13.4	0.66	4.50E-64	3.90E-62
RARRES2	7q36.1	0.66	5.15E-64	4.42E-62
NCKAP1L	12q13.13-q13.	0.66	5.15E-64	4.42E-62
SGCD	5q33.2-q33.3	0.66	6.36E-64	5.44E-62
LILRB3	19q13.42	0.66	6.41E-64	5.45E-62
SFRP2	4q31.3	0.66	9.47E-64	8.03E-62
S1PR2	19p13.2	0.66	1.29E-63	1.09E-61
HLA-DPB1	6p21.32	0.66	1.54E-63	1.29E-61
CD28	2q33.2	0.66	1.76E-63	1.47E-61
ARSB	5q14.1	0.66	1.94E-63	1.62E-61
LAT2	7q11.23	0.66	2.00E-63	1.66E-61
ANPEP	15q26.1	0.66	2.06E-63	1.71E-61
GREM1	15q13.3	0.66	2.23E-63	1.84E-61
GPR84	12q13.13	0.66	2.52E-63	2.07E-61
CLEC5A	7q34	0.66	2.58E-63	2.11E-61
CMKLR1	12q23.3	0.66	2.97E-63	2.41E-61
ADAMTS12	5p13.3-p13.2	0.66	2.98E-63	2.41E-61
ILK	11p15.4	0.66	3.30E-63	2.66E-61
CAMK1G	1q32.2	0.66	3.34E-63	2.68E-61
RSPO3	6q22.33	0.66	4.75E-63	3.80E-61
NFAM1	22q13.2	0.66	4.77E-63	3.81E-61
CCR1	3p21.31	0.66	5.89E-63	4.68E-61
FERMT2	14q22.1	0.66	7.01E-63	5.55E-61
SIGLEC7	19q13.41	0.66	9.17E-63	7.22E-61
TMEM200A	6q23.1	0.66	9.39E-63	7.37E-61
DIPK2B	Xp11.3	0.66	9.80E-63	7.67E-61
A2M	12p13.31	0.65	2.31E-62	1.80E-60
MYO1G	7p13	0.65	2.41E-62	1.87E-60
COL10A1	6q22.1	0.65	4.49E-62	3.47E-60
LPXN	11q12.1	0.65	5.97E-62	4.60E-60
CALHM5	6q22.1	0.65	6.33E-62	4.86E-60
CNN1	19p13.2	0.65	6.57E-62	5.02E-60
EFEMP2	11q13.1	0.65	6.98E-62	5.32E-60

TBXAS1	7q34	0.65	7.06E-62	5.35E-60
TYROBP	19q13.12	0.65	8.13E-62	6.14E-60
IL10RA	11q23.3	0.65	8.56E-62	6.45E-60
THBS1	15q14	0.65	9.16E-62	6.87E-60
PRRX1	1q24.2	0.65	1.27E-61	9.52E-60
PLXNA4	7q32.3	0.65	1.67E-61	1.24E-59
GLIPR2	9p13.3	0.65	1.76E-61	1.30E-59
PIK3R5	17p13.1	0.65	1.87E-61	1.38E-59
TNFSF8	9q32-q33.1	0.65	2.06E-61	1.52E-59
MAN1A1	6q22.31	0.65	3.23E-61	2.37E-59
COL8A2	1p34.3	0.65	3.72E-61	2.72E-59
CTHRC1	8q22.3	0.65	3.76E-61	2.74E-59
ENTPD1	10q24.1	0.65	3.97E-61	2.88E-59
VASN	16p13.3	0.65	4.22E-61	3.05E-59
DPT	1q24.2	0.65	5.18E-61	3.73E-59
ISM1	20p12.1	0.65	6.88E-61	4.94E-59
FMNL1	17q21.31	0.65	7.65E-61	5.48E-59
PAG1	8q21.13	0.65	7.97E-61	5.68E-59
CD34	1q32.2	0.65	9.05E-61	6.43E-59
C5AR1	19q13.32	0.65	9.12E-61	6.46E-59
SYNPO	5q33.1	0.65	1.05E-60	7.44E-59
BTK	Xq22.1	0.65	1.08E-60	7.62E-59
ENPP2	8q24.12	0.65	1.27E-60	8.88E-59
DYSF	2p13.2	0.65	1.36E-60	9.51E-59
MRAS	3q22.3	0.65	1.59E-60	1.10E-58
GPR132	14q32.33	0.65	1.60E-60	1.11E-58
LCP2	5q35.1	0.65	1.61E-60	1.12E-58
COX7A1	19q13.12	0.65	1.81E-60	1.25E-58
CCN1	1p22.3	0.65	2.10E-60	1.44E-58
TNFSF12	17p13.1	0.64	3.04E-60	2.08E-58
CHST11	12q23.3	0.64	3.24E-60	2.21E-58
EVI2B	17q11.2	0.64	3.27E-60	2.22E-58
NLRP12	19q13.42	0.64	3.42E-60	2.32E-58
LATS2	13q12.11	0.64	3.97E-60	2.68E-58
PTPRE	10q26.2	0.64	5.41E-60	3.64E-58
DOK2	8p21.3	0.64	5.56E-60	3.73E-58
SULF1	8q13.2-q13.3	0.64	6.39E-60	4.27E-58
CSF2RB	22q12.3	0.64	7.48E-60	4.98E-58
LILRB2	19q13.42	0.64	7.56E-60	5.01E-58
SNX20	16q12.1	0.64	7.59E-60	5.01E-58
HAVCR2	5q33.3	0.64	7.60E-60	5.01E-58
CREB3L1	11p11.2	0.64	8.45E-60	5.56E-58
CD86	3q13.33	0.64	8.69E-60	5.69E-58
AIF1	6p21.33	0.64	9.78E-60	6.39E-58
HLA-DPA1	6p21.32	0.64	1.03E-59	6.72E-58
VASH1	14q24.3	0.64	1.48E-59	9.59E-58
MRC1	10p12.33	0.64	1.58E-59	1.02E-57
CD53	1p13.3	0.64	1.90E-59	1.23E-57
TIMP1	Xp11.3	0.64	1.99E-59	1.28E-57
ITGA11	15q23	0.64	2.75E-59	1.76E-57
CD163	12p13.31	0.64	2.94E-59	1.88E-57
HLX	1q41	0.64	3.12E-59	1.98E-57
DOCK10	2q36.2	0.64	3.48E-59	2.21E-57
C1ORF162	1p13.2	0.64	3.82E-59	2.42E-57

C1ORF54	1q21.2	0.64	4.62E-59	2.91E-57
PCDH12	5q31.3	0.64	5.64E-59	3.55E-57
SNAI1	20q13.13	0.64	5.76E-59	3.61E-57
CXORF21	Xp21.2	0.64	6.75E-59	4.22E-57
CYTIP	2q24.1	0.64	7.53E-59	4.69E-57
RNASE6	14q11.2	0.64	8.07E-59	5.01E-57
RFLNA	12q24.31	0.64	1.02E-58	6.32E-57
IFFO1	12p13.31	0.64	1.06E-58	6.53E-57
CD37	19q13.33	0.64	1.11E-58	6.81E-57
TIE1	1p34.2	0.64	1.37E-58	8.43E-57
BHLHE22	8q12.3	0.64	1.63E-58	9.95E-57
TCF21	6q23.2	0.64	2.11E-58	1.28E-56
PLXND1	3q22.1	0.64	2.31E-58	1.40E-56
WNT2	7q31.2	0.64	2.53E-58	1.53E-56
PRELP	1q32.1	0.64	2.78E-58	1.68E-56
FCER1G	1q23.3	0.64	4.16E-58	2.50E-56
FOXP1	3p13	0.64	4.28E-58	2.57E-56
FIBIN	11p14.2	0.64	5.44E-58	3.26E-56
CCR2	3p21.31	0.64	5.88E-58	3.51E-56
FAM78A	9q34.13	0.63	6.94E-58	4.13E-56
TNFAIP8L2	1q21.3	0.63	7.37E-58	4.37E-56
CASS4	20q13.31	0.63	9.55E-58	5.65E-56
MNDA	1q23.1	0.63	9.66E-58	5.70E-56
HLA-DRA	6p21.32	0.63	9.71E-58	5.71E-56
REM1	20q11.21	0.63	1.10E-57	6.45E-56
SGIP1	1p31.3	0.63	1.20E-57	7.00E-56
WAS	Xp11.23	0.63	1.35E-57	7.85E-56
LRRC15	3q29	0.63	1.37E-57	7.95E-56
GRAP	17p11.2	0.63	1.38E-57	7.99E-56
GXYLT2	3p13	0.63	1.40E-57	8.08E-56
GJD3	17q21.2	0.63	1.43E-57	8.22E-56
MILR1	17q23.3	0.63	1.48E-57	8.51E-56
MEDAG	13q12.3	0.63	1.51E-57	8.64E-56
SCARF1	17p13.3	0.63	1.56E-57	8.91E-56
PLPP7	9q34.13	0.63	1.74E-57	9.94E-56
GMFG	19q13.2	0.63	1.78E-57	1.01E-55
ANXA5	4q27	0.63	2.33E-57	1.32E-55
LILRB1	19q13.42	0.63	2.54E-57	1.44E-55
CELF2	10p14	0.63	2.59E-57	1.46E-55
TNFAIP6	2q23.3	0.63	2.68E-57	1.51E-55
FNDC1	6q25.3	0.63	2.96E-57	1.66E-55
FAM180A	7q33	0.63	3.35E-57	1.87E-55
ENC1	5q13.3	0.63	3.69E-57	2.06E-55
PDLIM3	4q35.1	0.63	4.62E-57	2.57E-55
SLA	8q24.22	0.63	4.86E-57	2.69E-55
LOXL3	2p13.1	0.63	5.66E-57	3.13E-55
DPYSL2	8p21.2	0.63	5.81E-57	3.20E-55
ZNF423	16q12.1	0.63	5.93E-57	3.26E-55
IQGAP2	5q13.3	0.63	6.31E-57	3.46E-55
CD80	3q13.33	0.63	6.35E-57	3.47E-55
CSMD2	1p35.1	0.63	7.23E-57	3.94E-55
APBB1IP	10p12.1	0.63	7.40E-57	4.03E-55
LAMP5	20p12.2	0.63	8.18E-57	4.44E-55
PLPPR4	1p21.3-p21.2	0.63	9.00E-57	4.87E-55

PABPC5	Xq21.31	0.63	9.57E-57	5.16E-55
DOCK8	9p24.3	0.63	9.59E-57	5.16E-55
FAM20A	17q24.2	0.63	1.43E-56	7.67E-55
SLIT3	5q34-q35.1	0.63	1.48E-56	7.92E-55
FERMT3	11q13.1	0.63	1.53E-56	8.16E-55
ADAMTS10	19p13.2	0.63	1.57E-56	8.35E-55
PDGFRA	4q12	0.63	1.90E-56	1.01E-54
S1PR1	1p21.2	0.63	2.04E-56	1.08E-54
TNFSF12-TNFSF13	17p13.1	0.63	2.60E-56	1.37E-54
FCN1	9q34.3	0.63	3.27E-56	1.72E-54
SHISAL1	22q13.31	0.63	3.73E-56	1.96E-54
CCL2	17q12	0.63	4.08E-56	2.14E-54
CTSO	4q32.1	0.63	5.18E-56	2.71E-54
RGS3	9q32	0.63	5.37E-56	2.80E-54
CCDC80	3q13.2	0.63	5.50E-56	2.86E-54
SOD3	4p15.2	0.63	6.00E-56	3.11E-54
CACNA1C	12p13.33	0.63	6.55E-56	3.39E-54
TNFSF13B	13q33.3	0.63	6.77E-56	3.49E-54
PHLDB1	11q23.3	0.63	6.80E-56	3.50E-54
FCGR3A	1q23.3	0.63	8.25E-56	4.24E-54
PIK3R6	17p13.1	0.63	8.86E-56	4.54E-54
NRP1	10p11.22	0.62	1.32E-55	6.74E-54
HGF	7q21.11	0.62	1.86E-55	9.47E-54
GPRIN3	4q22.1	0.62	1.90E-55	9.66E-54
APBB2	4p14-p13	0.62	1.93E-55	9.76E-54
WDFY4	10q11.23	0.62	2.07E-55	1.05E-53
MYO1F	19p13.2	0.62	2.40E-55	1.21E-53
SH3KBP1	Xp22.12	0.62	2.48E-55	1.25E-53
FOLR2	11q13.4	0.62	2.49E-55	1.25E-53
C1R	12p13.31	0.62	2.64E-55	1.32E-53
CTSZ	20q13.32	0.62	2.65E-55	1.32E-53
LILRA6	19q13.42	0.62	2.91E-55	1.45E-53
PLCL2	3p24.3	0.62	2.94E-55	1.46E-53
XPNPEP2	Xq26.1	0.62	2.95E-55	1.46E-53
CALHM2	10q24.33	0.62	3.38E-55	1.67E-53
SCARF2	22q11.21	0.62	3.95E-55	1.95E-53
CILP	15q22.31	0.62	4.15E-55	2.04E-53
GIMAP4	7q36.1	0.62	4.49E-55	2.21E-53
LY86	6p25.1	0.62	4.87E-55	2.38E-53
ADAMTS16	5p15.32	0.62	5.23E-55	2.56E-53
ABCA6	17q24.2-q24.3	0.62	5.34E-55	2.60E-53
ANGPTL1	1q25.2	0.62	5.84E-55	2.84E-53
ADAMTS4	1q23.3	0.62	6.29E-55	3.05E-53
MPEG1	11q12.1	0.62	9.46E-55	4.58E-53
RFLNB	17p13.3	0.62	1.03E-54	4.99E-53
LILRB4	19q13.42	0.62	1.30E-54	6.27E-53
DENND2A	7q34	0.62	1.43E-54	6.89E-53
COL4A1	13q34	0.62	1.44E-54	6.89E-53
RCN3	19q13.33	0.62	1.64E-54	7.86E-53
CLEC4A	12p13.31	0.62	1.72E-54	8.22E-53
BTBD19	1p34.1	0.62	1.80E-54	8.59E-53
SEMA6B	19p13.3	0.62	2.02E-54	9.61E-53
C1QTNF2	5q33.3	0.62	2.12E-54	1.00E-52
ALOX5	10q11.21	0.62	2.28E-54	1.08E-52

PIK3CG	7q22.3	0.62	2.30E-54	1.09E-52
CPED1	7q31.31	0.62	2.36E-54	1.11E-52
PLN	6q22.31	0.62	2.47E-54	1.16E-52
ZEB1	10p11.22	0.62	2.60E-54	1.22E-52
HSPB7	1p36.13	0.62	2.82E-54	1.32E-52
ADORA1	1q32.1	0.62	2.94E-54	1.37E-52
TBX5	12q24.21	0.62	3.00E-54	1.39E-52
SIRPB2	20p13	0.62	3.21E-54	1.49E-52
RCSD1	1q24.2	0.62	3.24E-54	1.50E-52
HMCN1	1q25.3-q31.1	0.62	4.50E-54	2.08E-52
ST6GALNAC5	1p31.1	0.62	4.64E-54	2.14E-52
IL21R	16p12.1	0.62	5.05E-54	2.32E-52
IRF8	16q24.1	0.62	6.10E-54	2.80E-52
PTGDR	14q22.1	0.62	6.23E-54	2.85E-52
STK10	5q35.1	0.62	6.78E-54	3.09E-52
VSIG4	Xq12	0.62	7.06E-54	3.22E-52
GPR4	19q13.32	0.62	7.16E-54	3.26E-52
GIMAP8	7q36.1	0.62	7.65E-54	3.47E-52
TLR7	Xp22.2	0.62	1.11E-53	5.04E-52
SIGLEC5	19q13.41	0.62	1.19E-53	5.36E-52
SRGN	10q22.1	0.62	1.21E-53	5.44E-52
GJA5	1q21.2	0.62	1.48E-53	6.66E-52
F2R	5q13.3	0.62	1.49E-53	6.68E-52
CSF1	1p13.3	0.62	1.54E-53	6.87E-52
PTH1R	3p21.31	0.62	1.56E-53	6.98E-52
PEG3	19q13.43	0.62	1.60E-53	7.13E-52
SASH3	Xq26.1	0.62	1.83E-53	8.14E-52
PTPRC	1q31.3-q32.1	0.61	1.95E-53	8.66E-52
RAB8B	15q22.2	0.61	2.08E-53	9.23E-52
MSR1	8p22	0.61	2.13E-53	9.43E-52
ADRA2A	10q25.2	0.61	2.37E-53	1.05E-51
LILRA2	19q13.42	0.61	2.46E-53	1.08E-51
MMP9	20q13.12	0.61	3.02E-53	1.33E-51
NXPH3	17q21.33	0.61	3.15E-53	1.38E-51
CD84	1q23.3	0.61	3.22E-53	1.41E-51
SPN	16p11.2	0.61	3.25E-53	1.42E-51
KCNE4	2q36.1	0.61	4.48E-53	1.95E-51
MS4A4A	11q12.2	0.61	4.95E-53	2.15E-51
ABI3	17q21.32	0.61	5.06E-53	2.19E-51
LDLRAD4	18p11.21	0.61	5.08E-53	2.20E-51
IL16	15q25.1	0.61	5.22E-53	2.25E-51
TM4SF18	3q25.1	0.61	5.44E-53	2.34E-51
ICAM1	19p13.2	0.61	5.87E-53	2.52E-51
C1QC	1p36.12	0.61	6.17E-53	2.64E-51
IL32	16p13.3	0.61	7.46E-53	3.19E-51
CCR4	3p22.3	0.61	7.73E-53	3.30E-51
CD180	5q12.3	0.61	8.09E-53	3.44E-51
CDH5	16q21	0.61	8.70E-53	3.69E-51
RECK	9p13.3	0.61	8.98E-53	3.81E-51
COL14A1	8q24.12	0.61	1.06E-52	4.50E-51
MAGEL2	15q11.2	0.61	1.09E-52	4.60E-51
GIMAP1	7q36.1	0.61	1.10E-52	4.65E-51
PLVAP	19p13.11	0.61	1.11E-52	4.66E-51
GALNT17	7q11.22	0.61	1.15E-52	4.82E-51

NLRC4	2p22.3	0.61	1.28E-52	5.35E-51
CUBN	10p13	0.61	1.63E-52	6.82E-51
IL2RA	10p15.1	0.61	1.83E-52	7.62E-51
GNG11	7q21.3	0.61	1.96E-52	8.15E-51
CCBE1	18q21.32	0.61	2.01E-52	8.35E-51
MYCT1	6q25.2	0.61	2.72E-52	1.13E-50
LCP1	13q14.13	0.61	2.77E-52	1.15E-50
GIMAP6	7q36.1	0.61	3.37E-52	1.39E-50
F13A1	6p25.1	0.61	3.99E-52	1.64E-50
TMEM150B	19q13.42	0.61	4.24E-52	1.74E-50
TENM3	4q34.3-q35.1	0.61	6.82E-52	2.80E-50
MRC2	17q23.2	0.61	8.12E-52	3.32E-50
HSPB2	11q23.1	0.61	8.68E-52	3.54E-50
HLA-DOA	6p21.32	0.61	1.18E-51	4.83E-50
GGTA1P	9q33.2	0.61	1.20E-51	4.89E-50
IGDCC4	15q22.31	0.61	1.34E-51	5.46E-50
FLT4	5q35.3	0.61	1.35E-51	5.47E-50
WBP1L	10q24.32	0.61	1.47E-51	5.93E-50
TREM2	6p21.1	0.61	1.75E-51	7.06E-50
DEPP1	10q11.21	0.61	1.77E-51	7.11E-50
NTM	11q25	0.61	1.78E-51	7.14E-50
DCN	12q21.33	0.61	1.79E-51	7.18E-50
IKZF1	7p12.2	0.61	1.85E-51	7.42E-50
CAVIN3	11p15.4	0.61	1.89E-51	7.57E-50
IL4I1	19q13.33	0.61	2.19E-51	8.72E-50
PREX1	20q13.13	0.61	2.20E-51	8.75E-50
SAMSN1	21q11.2	0.60	3.06E-51	1.22E-49
TPM1	15q22.2	0.60	3.20E-51	1.27E-49
PRR16	5q23.1	0.60	3.24E-51	1.28E-49
SYNE3	14q32.13	0.60	3.35E-51	1.32E-49
STX2	12q24.33	0.60	3.36E-51	1.33E-49
POU2F2	19q13.2	0.60	3.58E-51	1.41E-49
FOXP3	Xp11.23	0.60	4.09E-51	1.60E-49
ARHGDIB	12p12.3	0.60	4.15E-51	1.63E-49
LAMA2	6q22.33	0.60	4.55E-51	1.78E-49
ENOX1	13q14.11	0.60	4.58E-51	1.79E-49
CYTL1	4p16.2	0.60	4.71E-51	1.83E-49
HLA-DRB1	6p21.32	0.60	5.06E-51	1.97E-49
CCDC102B	18q22.1-q22.[illegible]	0.60	5.19E-51	2.01E-49
4-Sep	17q22	0.60	5.25E-51	2.03E-49
CCR5	3p21.31	0.60	5.28E-51	2.04E-49
FKBP7	2q31.2	0.60	6.02E-51	2.32E-49
ZBTB46	20q13.33	0.60	6.50E-51	2.50E-49
TBC1D2B	15q24.3-q25.	0.60	6.72E-51	2.58E-49
FPR1	19q13.41	0.60	8.06E-51	3.09E-49
CD300A	17q25.1	0.60	8.13E-51	3.11E-49
CSDC2	22q13.2	0.60	9.75E-51	3.73E-49
COMP	19p13.11	0.60	9.78E-51	3.73E-49
LST1	6p21.33	0.60	1.11E-50	4.24E-49
CXCR4	2q22.1	0.60	1.30E-50	4.94E-49
ABI3BP	3q12.2	0.60	1.31E-50	4.96E-49
COL5A3	19p13.2	0.60	1.33E-50	5.02E-49
ZNF385D	3p24.3	0.60	1.41E-50	5.31E-49
TAGAP	6q25.3	0.60	1.74E-50	6.58E-49

RAI2	Xp22.13	0.60	1.79E-50	6.75E-49
ANTXR1	2p13.3	0.60	1.80E-50	6.75E-49
HCST	19q13.12	0.60	1.87E-50	7.03E-49
KCNJ8	12p12.1	0.60	1.89E-50	7.07E-49
TMEM273	10q11.23	0.60	2.08E-50	7.77E-49
OMD	9q22.31	0.60	2.30E-50	8.57E-49
GALNT15	3p25.1	0.60	2.37E-50	8.82E-49
ALPK2	18q21.31-q21.	0.60	2.44E-50	9.06E-49
JAML	11q23.3	0.60	2.46E-50	9.14E-49
RSU1	10p13	0.60	2.59E-50	9.57E-49
RNASE4	14q11.2	0.60	3.03E-50	1.12E-48
NCF4	22q12.3	0.60	3.20E-50	1.18E-48
PLXDC1	17q12	0.60	3.56E-50	1.31E-48
LIPC	15q21.3	0.60	3.65E-50	1.34E-48
CST7	20p11.21	0.60	3.84E-50	1.41E-48
P2RY8	Xp22.33 and `	0.60	4.61E-50	1.69E-48
RASA3	13q34	0.60	4.79E-50	1.75E-48
PPP1R18	6p21.33	0.60	5.34E-50	1.95E-48
LIX1L	1q21.1	0.60	5.44E-50	1.98E-48
EMILIN2	18p11.32-p11.	0.60	5.76E-50	2.09E-48
SLC49A3	4p16.3	0.60	5.86E-50	2.13E-48
MS4A6A	11q12.2	0.60	6.09E-50	2.21E-48
C1QA	1p36.12	0.60	7.18E-50	2.60E-48
BCL6B	17p13.1	0.60	7.24E-50	2.62E-48
TNFSF4	1q25.1	0.60	7.32E-50	2.64E-48
TLR8	Xp22.2	0.60	8.17E-50	2.94E-48
CSF2RA	Xp22.33 and `	0.60	8.45E-50	3.04E-48
LOC100129034	9q33.3	0.60	9.28E-50	3.33E-48
C2	6p21.33	0.60	1.09E-49	3.91E-48
MMP23B	1p36.33	0.60	1.18E-49	4.22E-48
HLA-DMB	6p21.32	0.60	1.18E-49	4.22E-48
HSD11B1	1q32.2	0.60	1.20E-49	4.27E-48
TMEM106A	17q21.31	0.60	1.51E-49	5.38E-48
ARL6IP5	3p14.1	0.60	1.81E-49	6.43E-48
TESPA1	12q13.2	0.60	2.06E-49	7.28E-48
TSPAN2	1p13.2	0.60	2.11E-49	7.45E-48
BIN2	12q13.13	0.60	2.15E-49	7.58E-48
JPH2	20q13.12	0.60	2.62E-49	9.24E-48
SIGLEC10	19q13.41	0.59	2.84E-49	9.99E-48
IL1R1	2q11.2-q12.1	0.59	3.09E-49	1.08E-47
EBF1	5q33.3	0.59	3.19E-49	1.12E-47
MGP	12p12.3	0.59	3.24E-49	1.13E-47
SLC16A4	1p13.3	0.59	3.31E-49	1.16E-47
HK3	5q35.2	0.59	3.64E-49	1.27E-47
MS4A7	11q12.2	0.59	3.82E-49	1.33E-47
SLCO2A1	3q22.1-q22.2	0.59	3.84E-49	1.33E-47
PDGFRL	8p22	0.59	3.98E-49	1.38E-47
TMEM255B	13q34	0.59	4.21E-49	1.46E-47
SLC16A2	Xq13.2	0.59	4.46E-49	1.54E-47
MXRA5	Xp22.33	0.59	4.54E-49	1.57E-47
NAP1L3	Xq21.32	0.59	4.66E-49	1.60E-47
OTULINL	5p15.2	0.59	5.07E-49	1.74E-47
CD300E	17q25.1	0.59	5.79E-49	1.99E-47
SORBS1	10q24.1	0.59	6.41E-49	2.19E-47

FCGR1A	1q21.2	0.59	8.36E-49	2.86E-47
PKIG	20q13.12	0.59	1.03E-48	3.51E-47
SPOCK2	10q22.1	0.59	1.14E-48	3.88E-47
KLF17	1p34.1	0.59	1.14E-48	3.88E-47
MFNG	22q13.1	0.59	1.26E-48	4.28E-47
ARHGAP15	2q22.2-q22.3	0.59	1.44E-48	4.87E-47
GPR65	14q31.3	0.59	1.51E-48	5.11E-47
TMEM26	10q21.2	0.59	1.53E-48	5.16E-47
NCF2	1q25.3	0.59	1.77E-48	5.97E-47
ROBO4	11q24.2	0.59	1.79E-48	6.01E-47
ARHGAP18	6q22.33	0.59	2.01E-48	6.75E-47
IFI30	19p13.11	0.59	2.12E-48	7.13E-47
AGTR1	3q24	0.59	2.16E-48	7.24E-47
TGM2	20q11.23	0.59	2.35E-48	7.88E-47
NFATC2	20q13.2	0.59	2.43E-48	8.13E-47
HEYL	1p34.2	0.59	2.50E-48	8.32E-47
NOX4	11q14.3	0.59	2.74E-48	9.11E-47
ADGRE5	19p13.12	0.59	2.95E-48	9.81E-47
FAM155A	13q33.3	0.59	3.12E-48	1.04E-46
ACTG2	2p13.1	0.59	3.14E-48	1.04E-46
MARCO	2q14.2	0.59	3.19E-48	1.05E-46
PRKCB	16p12.2-p12.	0.59	3.31E-48	1.09E-46
SUSD5	3p22.3	0.59	3.78E-48	1.25E-46
HTRA3	4p16.1	0.59	4.37E-48	1.44E-46
DRAM1	12q23.2	0.59	4.43E-48	1.46E-46
C16ORF54	16p11.2	0.59	4.47E-48	1.47E-46
CD68	17p13.1	0.59	4.72E-48	1.55E-46
BHLHE41	12p12.1	0.59	5.14E-48	1.68E-46
FGR	1p35.3	0.59	5.40E-48	1.76E-46
C1QB	1p36.12	0.59	6.11E-48	1.99E-46
CXCL12	10q11.21	0.59	6.20E-48	2.02E-46
NR5A2	1q32.1	0.59	6.21E-48	2.02E-46
GALNT10	5q33.2	0.59	6.41E-48	2.08E-46
HLA-DMA	6p21.32	0.59	7.92E-48	2.57E-46
GSTM5	1p13.3	0.59	9.22E-48	2.98E-46
SMAP2	1p34.2	0.59	9.77E-48	3.16E-46
ESAM	11q24.2	0.59	1.07E-47	3.44E-46
TLR4	9q33.1	0.59	1.12E-47	3.60E-46
SYT11	1q22	0.59	1.32E-47	4.23E-46
PTGS1	9q33.2	0.59	1.43E-47	4.59E-46
CCR8	3p22.1	0.59	1.76E-47	5.63E-46
CD300LF	17q25.1	0.59	1.96E-47	6.28E-46
CORIN	4p12	0.59	1.99E-47	6.37E-46
PIEZO2	18p11.22-p11.	0.59	2.15E-47	6.86E-46
PTPN22	1p13.2	0.58	2.48E-47	7.90E-46
AKAP12	6q25.1	0.58	2.55E-47	8.11E-46
CTSS	1q21.3	0.58	2.64E-47	8.37E-46
STAB1	3p21.1	0.58	2.72E-47	8.61E-46
ASPN	9q22.31	0.58	2.82E-47	8.91E-46
AZIN2	1p35.1	0.58	2.82E-47	8.91E-46
HRH2	5q35.2	0.58	3.20E-47	1.01E-45
CETP	16q13	0.58	3.70E-47	1.17E-45
C3	19p13.3	0.58	3.79E-47	1.19E-45
PLAUR	19q13.31	0.58	3.84E-47	1.20E-45

LGI2	4p15.2	0.58	4.08E-47	1.28E-45
TNFRSF9	1p36.23	0.58	4.86E-47	1.52E-45
KDR	4q12	0.58	5.56E-47	1.74E-45
ICAM2	17q23.3	0.58	6.05E-47	1.89E-45
SSPN	12p12.1	0.58	6.20E-47	1.93E-45
NKAPL	6p22.1	0.58	6.31E-47	1.96E-45
NALCN	13q32.3-q33.	0.58	6.65E-47	2.07E-45
CCR6	6q27	0.58	7.32E-47	2.27E-45
FAT4	4q28.1	0.58	9.39E-47	2.91E-45
BCL2A1	15q25.1	0.58	9.60E-47	2.97E-45
HAS2	8q24.13	0.58	9.61E-47	2.97E-45
PTPRM	18p11.23	0.58	9.77E-47	3.01E-45
CD5	11q12.2	0.58	1.03E-46	3.16E-45
CCN4	8q24.22	0.58	1.07E-46	3.30E-45
ITGAX	16p11.2	0.58	1.12E-46	3.44E-45
TSPAN11	12p11.21	0.58	1.22E-46	3.75E-45
DOK3	5q35.3	0.58	1.58E-46	4.84E-45
GAS1	9q21.33	0.58	1.94E-46	5.93E-45
SPON2	4p16.3	0.58	2.04E-46	6.22E-45
MOXD1	6q23.2	0.58	2.56E-46	7.79E-45
F5	1q24.2	0.58	2.77E-46	8.41E-45
CR1	1q32.2	0.58	2.82E-46	8.55E-45
ADAMTSL2	9q34.2	0.58	2.90E-46	8.79E-45
TEK	9p21.2	0.58	2.91E-46	8.82E-45
MCHR1	22q13.2	0.58	2.97E-46	8.97E-45
ZNF366	5q13.2|5q13.:	0.58	3.01E-46	9.08E-45
TNN	1q25.1	0.58	3.21E-46	9.67E-45
FGF7	15q21.2	0.58	3.62E-46	1.09E-44
CSRNP1	3p22.2	0.58	3.94E-46	1.18E-44
GPR68	14q32.11	0.58	4.00E-46	1.20E-44
SLC31A2	9q32	0.58	4.87E-46	1.46E-44
LILRB5	19q13.42	0.58	4.91E-46	1.47E-44
IGSF6	16p12.2	0.58	4.98E-46	1.49E-44
SLC41A2	12q23.3	0.58	5.05E-46	1.50E-44
FCGR1CP	1q21.1	0.58	5.06E-46	1.51E-44
CISH	3p21.2	0.58	5.11E-46	1.52E-44
CCDC170	6q25.1	0.58	5.58E-46	1.66E-44
AQP9	15q21.3	0.58	5.76E-46	1.71E-44
LPL	8p21.3	0.58	5.78E-46	1.71E-44
LRRC17	7q22.1	0.58	5.81E-46	1.72E-44
RAB33A	Xq26.1	0.58	5.88E-46	1.73E-44
TGFBI	5q31.1	0.58	6.38E-46	1.88E-44
TSHZ3	19q12	0.58	6.55E-46	1.93E-44
ARHGEF17	11q13.4	0.58	7.17E-46	2.11E-44
PLA2G7	6p12.3	0.58	7.60E-46	2.23E-44
SLC43A3	11q12.1	0.58	7.74E-46	2.27E-44
FGL2	7q11.23	0.58	8.10E-46	2.37E-44
LZTS1	8p21.3	0.58	9.00E-46	2.63E-44
COL4A2	13q34	0.58	9.09E-46	2.65E-44
LSAMP	3q13.31	0.58	9.26E-46	2.70E-44
ARHGEF15	17p13.1	0.58	1.23E-45	3.56E-44
CDK14	7q21.13	0.58	1.28E-45	3.71E-44
ITGA9	3p22.2	0.58	1.37E-45	3.97E-44
PLEK	2p14	0.58	1.54E-45	4.47E-44

PODNL1	19p13.12	0.58	1.59E-45	4.61E-44
ADGRE2	19p13.12	0.58	1.74E-45	5.02E-44
COL13A1	10q22.1	0.58	1.84E-45	5.30E-44
CCRL2	3p21.31	0.58	1.87E-45	5.38E-44
SIRPB1	20p13	0.58	1.97E-45	5.67E-44
GNGT2	17q21.32	0.57	2.04E-45	5.86E-44
FSTL1	3q13.33	0.57	2.25E-45	6.44E-44
INPP5D	2q37.1	0.57	2.33E-45	6.66E-44
APLNR	11q12.1	0.57	2.56E-45	7.32E-44
FCGR1B	1p11.2	0.57	2.71E-45	7.72E-44
DPEP2	16q22.1	0.57	2.78E-45	7.91E-44
GIMAP5	7q36.1	0.57	2.89E-45	8.21E-44
IL10	1q32.1	0.57	3.10E-45	8.80E-44
S1PR3	9q22.1	0.57	3.24E-45	9.19E-44
PARVG	22q13.31	0.57	3.26E-45	9.25E-44
LILRA1	19q13.42	0.57	3.65E-45	1.03E-43
TM6SF1	15q25.2	0.57	4.14E-45	1.17E-43
FZD4	11q14.2	0.57	4.45E-45	1.26E-43
RIN3	14q32.12	0.57	5.70E-45	1.61E-43
TCEAL7	Xq22.2	0.57	6.26E-45	1.76E-43
ICOS	2q33.2	0.57	6.66E-45	1.87E-43
SIGLEC14	19q13.41	0.57	7.09E-45	1.99E-43
RGS16	1q25.3	0.57	8.18E-45	2.29E-43
RHOH	4p14	0.57	8.23E-45	2.30E-43
APCDD1L	20q13.32	0.57	9.50E-45	2.66E-43
SLC15A3	11q12.2	0.57	9.60E-45	2.68E-43
MMP19	12q13.2	0.57	9.97E-45	2.78E-43
C1QTNF7	4p15.32	0.57	1.00E-44	2.80E-43
DLL4	15q15.1	0.57	1.06E-44	2.94E-43
HTRA1	10q26.13	0.57	1.09E-44	3.02E-43
PHC2	1p35.1	0.57	1.11E-44	3.08E-43
BEAN1	16q21	0.57	1.15E-44	3.17E-43
ASGR2	17p13.1	0.57	1.15E-44	3.18E-43
SOGA3	6q22.33	0.57	1.16E-44	3.21E-43
CPQ	8q22.1	0.57	1.17E-44	3.23E-43
PDLIM2	8p21.3	0.57	1.19E-44	3.27E-43
GJA4	1p34.3	0.57	1.42E-44	3.90E-43
ADAMTS14	10q22.1	0.57	1.57E-44	4.30E-43
CYSLTR1	Xq21.1	0.57	2.10E-44	5.75E-43
FUT7	9q34.3	0.57	2.16E-44	5.93E-43
CORO1A	16p11.2	0.57	2.17E-44	5.93E-43
DPP4	2q24.2	0.57	2.63E-44	7.19E-43
ABCC9	12p12.1	0.57	2.65E-44	7.21E-43
CEACAM21	19q13.2	0.57	2.72E-44	7.41E-43
IGSF21	1p36.13	0.57	2.79E-44	7.58E-43
VDR	12q13.11	0.57	2.88E-44	7.83E-43
IL12RB1	19p13.11	0.57	3.49E-44	9.46E-43
OLR1	12p13.2	0.57	3.75E-44	1.01E-42
PLCL1	2q33.1	0.57	3.82E-44	1.03E-42
IL2RG	Xq13.1	0.57	3.89E-44	1.05E-42
GIMAP7	7q36.1	0.57	4.13E-44	1.11E-42
CCN5	20q13.12	0.57	4.18E-44	1.13E-42
PMEPA1	20q13.31	0.57	4.41E-44	1.19E-42
ACOX2	3p14.3	0.57	5.77E-44	1.55E-42

HSPA12B	20p13	0.57	5.86E-44	1.57E-42
PPP1R16B	20q11.23	0.57	6.73E-44	1.80E-42
IRAK3	12q14.3	0.57	6.78E-44	1.81E-42
SLC1A7	1p32.3	0.57	8.23E-44	2.20E-42
MEIS3	19q13.32	0.57	9.33E-44	2.49E-42
CCDC69	5q33.1	0.57	9.43E-44	2.51E-42
TRIM61	4q32.3	0.57	1.00E-43	2.66E-42
PEAK1	15q24.3	0.57	1.15E-43	3.06E-42
SCIMP	17p13.2	0.57	1.20E-43	3.18E-42
SIGLEC1	20p13	0.57	1.28E-43	3.38E-42
PTGDS	9q34.3	0.56	1.45E-43	3.85E-42
LOC100126784	11p15.1	0.56	1.52E-43	4.02E-42
ITGA5	12q13.13	0.56	1.57E-43	4.13E-42
ITGA8	10p13	0.56	1.73E-43	4.57E-42
ADAM8	10q26.3	0.56	1.78E-43	4.68E-42
COL11A1	1p21.1	0.56	2.06E-43	5.42E-42
IFITM2	11p15.5	0.56	2.43E-43	6.38E-42
MCAM	11q23.3	0.56	2.59E-43	6.79E-42
ADORA2A	22q11.23	0.56	2.60E-43	6.81E-42
CCL23	17q12	0.56	2.78E-43	7.29E-42
KCNA3	1p13.3	0.56	2.92E-43	7.64E-42
LUM	12q21.33	0.56	2.94E-43	7.68E-42
SORCS2	4p16.1	0.56	3.37E-43	8.78E-42
GUCY1A2	11q22.3	0.56	3.54E-43	9.21E-42
ZC3H8	2q14.1	-0.56	4.48E-43	1.16E-41
MFGE8	15q26.1	0.56	4.55E-43	1.18E-41
FYB1	5p13.1	0.56	5.65E-43	1.47E-41
PEAK3	19p13.3	0.56	5.97E-43	1.55E-41
AP1S2	Xp22.2	0.56	6.25E-43	1.62E-41
MAP4	3p21.31	0.56	6.38E-43	1.65E-41
ADARB1	21q22.3	0.56	7.70E-43	1.99E-41
JAK1	1p31.3	0.56	8.19E-43	2.11E-41
CD209	19p13.2	0.56	8.92E-43	2.30E-41
FLVCR2	14q24.3	0.56	8.93E-43	2.30E-41
XYLT1	16p12.3	0.56	9.76E-43	2.51E-41
PGM5	9q21.11	0.56	9.77E-43	2.51E-41
SMIM10	Xq26.3	0.56	9.81E-43	2.51E-41
PLXDC2	10p12.31	0.56	9.88E-43	2.53E-41
EPSTI1	13q14.11	0.56	1.11E-42	2.82E-41
P2RY10	Xq21.1	0.56	1.16E-42	2.95E-41
KMO	1q43	0.56	1.22E-42	3.11E-41
HPGDS	4q22.3	0.56	1.32E-42	3.35E-41
ITGAL	16p11.2	0.56	1.34E-42	3.41E-41
IFNAR2	21q22.11	0.56	1.41E-42	3.57E-41
C19ORF38	19p13.2	0.56	1.41E-42	3.57E-41
OGN	9q22.31	0.56	1.74E-42	4.41E-41
S100A4	1q21.3	0.56	1.75E-42	4.43E-41
ADAMTS5	21q21.3	0.56	1.85E-42	4.67E-41
SH2D3C	9q34.11	0.56	2.11E-42	5.33E-41
CHST15	10q26.13	0.56	2.45E-42	6.18E-41
CD69	12p13.31	0.56	2.49E-42	6.25E-41
GPR176	15q14-q15.1	0.56	3.05E-42	7.67E-41
RASSF4	10q11.21	0.56	3.10E-42	7.78E-41
NEK6	9q33.3	0.56	3.11E-42	7.80E-41

PRICKLE1	12q12	0.56	3.12E-42	7.82E-41
PDE2A	11q13.4	0.56	3.37E-42	8.43E-41
ARHGAP6	Xp22.2	0.56	3.50E-42	8.75E-41
ICAM3	19p13.2	0.56	3.84E-42	9.58E-41
LINC00341	14q32.13	0.56	3.85E-42	9.58E-41
RNASE1	14q11.2	0.56	4.33E-42	1.08E-40
PLEKHO1	1q21.2	0.56	4.52E-42	1.12E-40
CD52	1p36.11	0.56	5.08E-42	1.26E-40
ARRB1	11q13.4	0.56	5.08E-42	1.26E-40
CCL13	17q12	0.56	5.45E-42	1.35E-40
DNM3	1q24.3	0.56	5.45E-42	1.35E-40
ARHGAP25	2p13.3	0.56	5.45E-42	1.35E-40
ITK	5q33.3	0.56	5.52E-42	1.36E-40
GAB3	Xq28	0.56	5.94E-42	1.46E-40
SHANK1	19q13.33	0.56	5.97E-42	1.47E-40
IQSEC1	3p25.2-p25.1	0.56	6.44E-42	1.58E-40
PLK3	1p34.1	0.56	6.45E-42	1.58E-40
S1PR4	19p13.3	0.56	7.57E-42	1.86E-40
RTL3	Xq21.1	0.55	9.22E-42	2.26E-40
GREM2	1q43	0.55	9.56E-42	2.34E-40
MMP14	14q11.2	0.55	1.04E-41	2.55E-40
CRYBB1	22q12.1	0.55	1.08E-41	2.65E-40
HLA-DQA1	6p21.32	0.55	1.22E-41	2.96E-40
GPR34	Xp11.4	0.55	1.30E-41	3.16E-40
PCED1B-AS1	12q13.11	0.55	1.33E-41	3.24E-40
COTL1	16q24.1	0.55	1.34E-41	3.27E-40
CIITA	16p13.13	0.55	1.37E-41	3.33E-40
MGAT1	5q35.3	0.55	1.67E-41	4.05E-40
PRDM6	5q23.2	0.55	1.73E-41	4.17E-40
ITGA4	2q31.3	0.55	1.75E-41	4.22E-40
TRABD2A	2p11.2	0.55	1.77E-41	4.26E-40
P2RY13	3q25.1	0.55	1.80E-41	4.35E-40
NOVA2	19q13.32	0.55	2.01E-41	4.85E-40
VAMP5	2p11.2	0.55	2.16E-41	5.20E-40
CYP27A1	2q35	0.55	2.26E-41	5.43E-40
MAP1LC3C	1q43	0.55	2.27E-41	5.44E-40
TPK1	7q35	0.55	2.39E-41	5.73E-40
CDH23	10q22.1	0.55	2.61E-41	6.25E-40
DPYD	1p21.3	0.55	2.62E-41	6.28E-40
PLAC9	10q22.3	0.55	2.65E-41	6.32E-40
HTR2A	13q14.2	0.55	2.83E-41	6.76E-40
FCMR	1q32.1	0.55	2.88E-41	6.85E-40
ADGRF5	6p12.3	0.55	2.90E-41	6.91E-40
AGAP2	12q14.1	0.55	2.93E-41	6.96E-40
P2RX7	12q24.31	0.55	3.14E-41	7.46E-40
C11ORF96	11p11.2	0.55	3.20E-41	7.58E-40
TMEM173	5q31.2	0.55	3.60E-41	8.52E-40
LPAR4	Xq21.1	0.55	3.81E-41	9.01E-40
ECSCR	5q31.2	0.55	3.93E-41	9.28E-40
CATSPER1	11q13.1	0.55	4.00E-41	9.43E-40
MATN3	2p24.1	0.55	4.13E-41	9.72E-40
MYLK	3q21.1	0.55	4.16E-41	9.80E-40
KCNJ5	11q24.3	0.55	4.30E-41	1.01E-39
BHMT2	5q14.1	0.55	4.55E-41	1.07E-39

TPM2	9p13.3	0.55	5.49E-41	1.29E-39
APOBR	16p12.1	0.55	5.76E-41	1.35E-39
KCNIP1	5q35.1	0.55	5.94E-41	1.39E-39
COL12A1	6q13-q14.1	0.55	5.94E-41	1.39E-39
STAT4	2q32.2-q32.3	0.55	6.81E-41	1.59E-39
HIVEP3	1p34.2	0.55	7.26E-41	1.69E-39
MMRN2	10q23.2	0.55	7.60E-41	1.77E-39
EMB	5q11.1	0.55	7.82E-41	1.82E-39
SAMD14	17q21.33	0.55	8.71E-41	2.02E-39
TUBA1A	12q13.12	0.55	8.90E-41	2.07E-39
ABCB4	7q21.12	0.55	9.01E-41	2.09E-39
SHE	1q21.3	0.55	9.90E-41	2.29E-39
SEC24D	4q26	0.55	9.98E-41	2.31E-39
ADAMTS7	15q25.1	0.55	1.01E-40	2.33E-39
ELK3	12q23.1	0.55	1.04E-40	2.39E-39
SYDE1	19p13.12	0.55	1.27E-40	2.92E-39
ADORA3	1p13.2	0.55	1.29E-40	2.97E-39
KLF2	19p13.11	0.55	1.31E-40	3.02E-39
PCDH18	4q28.3	0.55	1.38E-40	3.17E-39
LOC401463	8q12.3	0.55	1.38E-40	3.18E-39
EVA1A	2p12	0.55	1.55E-40	3.56E-39
ZNF469	16q24.2	0.55	1.95E-40	4.47E-39
JAM3	11q25	0.55	2.08E-40	4.76E-39
NTNG2	9q34.13	0.55	2.18E-40	4.99E-39
SLC39A8	4q24	0.55	2.21E-40	5.05E-39
CGNL1	15q21.3	0.55	2.25E-40	5.13E-39
SCN1B	19q13.11	0.55	2.31E-40	5.26E-39
LILRA5	19q13.42	0.55	2.76E-40	6.27E-39
SHROOM4	Xp11.22	0.55	2.89E-40	6.56E-39
GLIS2	16p13.3	0.55	3.09E-40	7.01E-39
SH3BGRL	Xq21.1	0.55	3.16E-40	7.17E-39
STK17B	2q32.3	0.55	3.60E-40	8.15E-39
RTN1	14q23.1	0.55	3.78E-40	8.56E-39
XIRP1	3p22.2	0.55	4.03E-40	9.10E-39
SH3BP5	3p25.1	0.55	4.06E-40	9.17E-39
TRAF3IP3	1q32.2	0.55	4.14E-40	9.34E-39
C1S	12p13.31	0.54	4.39E-40	9.88E-39
HLA-DQB1	6p21.32	0.54	4.77E-40	1.07E-38
SLAMF1	1q23.3	0.54	4.81E-40	1.08E-38
HSPB6	19q13.12	0.54	5.00E-40	1.12E-38
HLA-DQA2	6p21.32	0.54	5.06E-40	1.13E-38
RNASE2	14q11.2	0.54	5.13E-40	1.15E-38
LYZ	12q15	0.54	5.45E-40	1.22E-38
PDE3A	12p12.2	0.54	5.68E-40	1.27E-38
FYN	6q21	0.54	6.04E-40	1.35E-38
APOE	19q13.32	0.54	6.64E-40	1.48E-38
PODXL	7q32.3	0.54	6.82E-40	1.52E-38
PRICKLE2	3p14.1	0.54	7.88E-40	1.75E-38
STAT5A	17q21.2	0.54	7.95E-40	1.77E-38
LY6H	8q24.3	0.54	8.12E-40	1.80E-38
LRRN4CL	11q12.3	0.54	8.46E-40	1.88E-38
CRIP2	14q32.33	0.54	9.12E-40	2.02E-38
NCF1	7q11.23	0.54	9.63E-40	2.13E-38
CD300LB	17q25.1	0.54	1.06E-39	2.34E-38

MICAL1	6q21	0.54	1.08E-39	2.39E-38
FSTL3	19p13.3	0.54	1.11E-39	2.44E-38
SIRPA	20p13	0.54	1.12E-39	2.46E-38
NBL1	1p36.13	0.54	1.17E-39	2.58E-38
TFEC	7q31.2	0.54	1.18E-39	2.59E-38
SLAMF6	1q23.2-q23.3	0.54	1.24E-39	2.73E-38
PTGER3	1p31.1	0.54	1.39E-39	3.06E-38
FGD2	6p21.2	0.54	1.53E-39	3.34E-38
VENTX	10q26.3	0.54	1.53E-39	3.35E-38
PRKG1	10q11.23-q21.	0.54	1.65E-39	3.62E-38
ADAMTS6	5q12.3	0.54	1.93E-39	4.22E-38
SLC6A1	3p25.3	0.54	1.94E-39	4.24E-38
EXTL1	1p36.11	0.54	2.01E-39	4.38E-38
HLA-DQB2	6p21.32	0.54	2.08E-39	4.52E-38
UNC5B	10q22.1	0.54	2.17E-39	4.71E-38
FLT1	13q12.3	0.54	2.44E-39	5.30E-38
TNFRSF8	1p36.22	0.54	2.98E-39	6.46E-38
F10	13q34	0.54	2.99E-39	6.49E-38
ZNF804A	2q32.1	0.54	3.39E-39	7.33E-38
SYPL2	1p13.3	0.54	3.40E-39	7.36E-38
ITPR2	12p11.23	0.54	3.49E-39	7.55E-38
DLG2	11q14.1	0.54	4.11E-39	8.87E-38
GUCY1A1	4q32.1	0.54	4.59E-39	9.89E-38
HLA-DRB6	6p21.32	0.54	4.66E-39	1.00E-37
KLHL6	3q27.1	0.54	4.68E-39	1.01E-37
GRK5	10q26.11	0.54	4.70E-39	1.01E-37
NRP2	2q33.3	0.54	4.80E-39	1.03E-37
CLEC1A	12p13.2	0.54	5.22E-39	1.12E-37
CLEC4D	12p13.31	0.54	5.51E-39	1.18E-37
SDC3	1p35.2	0.54	5.70E-39	1.22E-37
IQSEC3	12p13.33	0.54	6.06E-39	1.29E-37
RHOA	3p21.31	0.54	6.23E-39	1.33E-37
RGS18	1q31.2	0.54	6.57E-39	1.40E-37
SFRP4	7p14.1	0.54	6.76E-39	1.44E-37
DIXDC1	11q23.1	0.54	6.77E-39	1.44E-37
MAPRE2	18q12.1-q12.	0.54	6.95E-39	1.48E-37
C5AR2	19q13.32	0.54	7.34E-39	1.56E-37
DNAJC5B	8q13.1	0.54	7.47E-39	1.58E-37
PTPRB	12q15	0.54	7.87E-39	1.67E-37
PTGFR	1p31.1	0.54	9.76E-39	2.07E-37
CLEC14A	14q21.1	0.54	1.02E-38	2.16E-37
HAPLN3	15q26.1	0.54	1.06E-38	2.23E-37
RUNX2	6p21.1	0.54	1.13E-38	2.39E-37
ITIH5	10p14	0.54	1.16E-38	2.44E-37
TREM1	6p21.1	0.54	1.22E-38	2.57E-37
PAPPA	9q33.1	0.54	1.22E-38	2.57E-37
ZNF300P1	5q33.1	0.54	1.28E-38	2.69E-37
SEMA7A	15q24.1	0.54	1.36E-38	2.85E-37
C16ORF45	16p13.11	0.54	1.50E-38	3.15E-37
COL18A1	21q22.3	0.54	1.54E-38	3.23E-37
LIMS3	2q13	0.54	1.58E-38	3.31E-37
MAP3K3	17q23.3	0.54	1.60E-38	3.34E-37
IL2RB	22q12.3	0.54	1.70E-38	3.56E-37
PPFIA2	12q21.31	0.54	1.73E-38	3.60E-37

PLA2G15	16q22.1	0.53	2.42E-38	5.04E-37
GATA6	18q11.2	0.53	2.43E-38	5.05E-37
CLIP3	19q13.12	0.53	2.90E-38	6.04E-37
TMEM233	12q24.23	0.53	2.93E-38	6.08E-37
TRANK1	3p22.2	0.53	3.27E-38	6.78E-37
NPR1	1q21.3	0.53	3.46E-38	7.17E-37
ATP8B4	15q21.2	0.53	3.66E-38	7.59E-37
TMEM130	7q22.1	0.53	3.80E-38	7.86E-37
GVINP1	11p15.4	0.53	3.81E-38	7.88E-37
DCSTAMP	8q22.3	0.53	3.95E-38	8.16E-37
JAK3	19p13.11	0.53	4.00E-38	8.25E-37
TPP1	11p15.4	0.53	4.24E-38	8.74E-37
ARHGEF40	14q11.2	0.53	4.53E-38	9.32E-37
LRFN5	14q21.1	0.53	4.91E-38	1.01E-36
NECAP2	1p36.13	0.53	5.71E-38	1.17E-36
TICAM2	5q22.3	0.53	5.92E-38	1.21E-36
SNTB1	8q24.12	0.53	6.01E-38	1.23E-36
DOCK4	7q31.1	0.53	6.49E-38	1.33E-36
CD3E	11q23.3	0.53	6.56E-38	1.34E-36
PCDHGB6	5q31.3	0.53	7.97E-38	1.63E-36
LYVE1	11p15.4	0.53	8.43E-38	1.72E-36
ADAMTS9	3p14.1	0.53	8.62E-38	1.76E-36
CSGALNACT1	8p21.3	0.53	9.50E-38	1.94E-36
C5ORF46	5q32	0.53	1.02E-37	2.07E-36
PTAFR	1p35.3	0.53	1.04E-37	2.12E-36
CLEC10A	17p13.1	0.53	1.11E-37	2.26E-36
ADAP2	17q11.2	0.53	1.17E-37	2.37E-36
HEG1	3q21.2	0.53	1.18E-37	2.40E-36
GZMK	5q11.2	0.53	1.22E-37	2.47E-36
WWC2	4q35.1	0.53	1.23E-37	2.50E-36
FABP3	1p35.2	0.53	1.27E-37	2.56E-36
MAMDC2	9q21.12	0.53	1.39E-37	2.80E-36
MPP1	Xq28	0.53	1.46E-37	2.94E-36
FAM13C	10q21.1	0.53	1.60E-37	3.22E-36
SPRY4	5q31.3	0.53	1.86E-37	3.75E-36
KANK4	1p31.3	0.53	2.11E-37	4.24E-36
LIMS2	2q14.3	0.53	2.12E-37	4.26E-36
ANKRD44	2q33.1	0.53	2.33E-37	4.68E-36
NCF1C	7q11.23	0.53	2.38E-37	4.78E-36
CD40LG	Xq26.3	0.53	2.84E-37	5.69E-36
ACAP1	17p13.1	0.53	2.86E-37	5.73E-36
SYTL2	11q14.1	0.53	3.00E-37	6.00E-36
ZYX	7q34	0.53	3.19E-37	6.37E-36
PAM	5q21.1	0.53	3.85E-37	7.69E-36
KIF26B	1q44	0.53	4.99E-37	9.94E-36
EPYC	12q21.33	0.53	5.47E-37	1.09E-35
LRCH1	13q14.13-q14.	0.53	5.63E-37	1.12E-35
SAMHD1	20q11.23	0.53	6.11E-37	1.22E-35
TWF2	3p21.2	0.53	6.72E-37	1.34E-35
CPM	12q15	0.53	6.85E-37	1.36E-35
CD48	1q23.3	0.53	7.00E-37	1.39E-35
CCL21	9p13.3	0.52	7.86E-37	1.56E-35
KCNK13	14q32.11	0.52	8.67E-37	1.72E-35
ATP6V1B2	8p21.3	0.52	8.86E-37	1.75E-35

SPRED1	15q14	0.52	9.31E-37	1.84E-35
ANK2	4q25-q26	0.52	9.75E-37	1.92E-35
RGS4	1q23.3	0.52	9.93E-37	1.96E-35
SH3PXD2B	5q35.1	0.52	1.01E-36	1.99E-35
PLPP4	10q26.12	0.52	1.02E-36	2.01E-35
IL6ST	5q11.2	0.52	1.06E-36	2.08E-35
AOX1	2q33.1	0.52	1.08E-36	2.13E-35
CEACAM4	19q13.2	0.52	1.11E-36	2.18E-35
ADA2	22q11.1	0.52	1.14E-36	2.24E-35
SOX17	8q11.23	0.52	1.19E-36	2.33E-35
WFDC1	16q24.1	0.52	1.26E-36	2.46E-35
FBXL7	5p15.1	0.52	1.30E-36	2.53E-35
CLEC4E	12p13.31	0.52	1.37E-36	2.68E-35
ITPR1	3p26.1	0.52	1.65E-36	3.22E-35
RGS1	1q31.2	0.52	1.70E-36	3.30E-35
INSYN2B	5q35.1	0.52	1.80E-36	3.51E-35
CYS1	2p25.1	0.52	1.93E-36	3.76E-35
RGCC	13q14.11	0.52	1.99E-36	3.87E-35
ARHGAP9	12q13.3	0.52	2.05E-36	3.99E-35
FHL3	1p34.3	0.52	2.06E-36	3.99E-35
SLC11A1	2q35	0.52	2.12E-36	4.10E-35
MYL3	3p21.31	0.52	2.13E-36	4.12E-35
ENPEP	4q25	0.52	2.74E-36	5.30E-35
GPR88	1p21.2	0.52	2.76E-36	5.34E-35
FRZB	2q32.1	0.52	2.95E-36	5.69E-35
RCCD1	15q26.1	-0.52	3.13E-36	6.04E-35
PAPSS2	10q23.2-q23.[illegible]	0.52	3.39E-36	6.53E-35
LMCD1	3p25.3	0.52	3.47E-36	6.67E-35
SLC2A5	1p36.23	0.52	4.05E-36	7.78E-35
NKD1	16q12.1	0.52	4.11E-36	7.90E-35
FAIM2	12q13.12	0.52	4.44E-36	8.51E-35
RAC2	22q13.1	0.52	5.14E-36	9.86E-35
P2RX1	17p13.2	0.52	5.22E-36	9.99E-35
CAMK2A	5q32	0.52	5.24E-36	1.00E-34
1-Mar	4q32.2-q32.3	0.52	5.27E-36	1.01E-34
ZC4H2	Xq11.2	0.52	5.99E-36	1.14E-34
SLC29A3	10q22.1	0.52	7.27E-36	1.39E-34
MMP16	8q21.3	0.52	7.79E-36	1.48E-34
PTGIS	20q13.13	0.52	7.97E-36	1.52E-34
PKNOX2	11q24.2	0.52	8.47E-36	1.61E-34
SLC39A13	11p11.2	0.52	8.78E-36	1.67E-34
PIK3CD	1p36.22	0.52	8.88E-36	1.69E-34
PCDHGB7	5q31.3	0.52	8.91E-36	1.69E-34
LCK	1p35.2	0.52	9.04E-36	1.71E-34
TMEM86A	11p15.1	0.52	9.04E-36	1.71E-34
LYL1	19p13.13	0.52	9.28E-36	1.75E-34
ZBTB47	3p22.1	0.52	1.02E-35	1.93E-34
PDE1A	2q32.1	0.52	1.13E-35	2.13E-34
PLA1A	3q13.33	0.52	1.14E-35	2.15E-34
ORAI2	7q22.1	0.52	1.17E-35	2.20E-34
GPR141	7p14.1	0.52	1.20E-35	2.26E-34
TNFRSF4	1p36.33	0.52	1.27E-35	2.39E-34
FRMD4A	10p13	0.52	1.35E-35	2.54E-34
CTLA4	2q33.2	0.52	1.39E-35	2.62E-34

LRRC55	11q12.1	0.52	1.45E-35	2.73E-34
GUCA1A	6p21.1	0.52	1.50E-35	2.81E-34
FLT3	13q12.2	0.52	1.78E-35	3.34E-34
ACP5	19p13.2	0.52	1.91E-35	3.56E-34
RELN	7q22.1	0.52	1.97E-35	3.68E-34
AKNA	9q32	0.52	2.07E-35	3.86E-34
CLIC4	1p36.11	0.52	2.28E-35	4.26E-34
PDCD1LG2	9p24.1	0.52	2.32E-35	4.32E-34
LSP1	11p15.5	0.52	2.49E-35	4.63E-34
SAMD11	1p36.33	0.52	2.61E-35	4.85E-34
SIGLEC15	18q12.3	0.51	2.81E-35	5.22E-34
LOX	5q23.1	0.51	2.83E-35	5.26E-34
LINC00922	16q21	0.51	2.87E-35	5.32E-34
FAM225B	9q32	0.51	3.03E-35	5.62E-34
CHI3L2	1p13.2	0.51	3.09E-35	5.73E-34
HNMT	2q22.1	0.51	3.14E-35	5.81E-34
HLA-DRB5	6p21.32	0.51	3.46E-35	6.39E-34
GRAP2	22q13.1	0.51	3.48E-35	6.42E-34
PCDHGA9	5q31.3	0.51	3.49E-35	6.43E-34
GIMAP2	7q36.1	0.51	3.53E-35	6.50E-34
RAB42	1p35.3	0.51	3.89E-35	7.17E-34
ARHGAP30	1q23.3	0.51	3.99E-35	7.34E-34
SLC2A6	9q34.2	0.51	4.10E-35	7.54E-34
EXOC3L2	19q13.32	0.51	4.45E-35	8.17E-34
LOC100130872	4p16.3	0.51	5.29E-35	9.71E-34
RASAL3	19p13.12	0.51	5.41E-35	9.91E-34
VAT1L	16q23.1	0.51	5.62E-35	1.03E-33
SSTR2	17q25.1	0.51	5.75E-35	1.05E-33
CD2	1p13.1	0.51	5.95E-35	1.09E-33
LGMN	14q32.12	0.51	6.12E-35	1.12E-33
RASGEF1B	4q21.21	0.51	6.67E-35	1.22E-33
SLC12A4	16q22.1	0.51	7.21E-35	1.31E-33
CD226	18q22.2	0.51	7.23E-35	1.32E-33
RSPO1	1p34.3	0.51	8.54E-35	1.55E-33
TOX	8q12.1	0.51	1.03E-34	1.87E-33
TMEM47	Xp21.1	0.51	1.04E-34	1.88E-33
CXCR3	Xq13.1	0.51	1.05E-34	1.90E-33
SPRY1	4q28.1	0.51	1.05E-34	1.91E-33
SCT	11p15.5	0.51	1.09E-34	1.98E-33
COL16A1	1p35.2	0.51	1.25E-34	2.26E-33
CHSY3	5q23.3	0.51	1.29E-34	2.33E-33
CCL18	17q12	0.51	1.35E-34	2.44E-33
GNG7	19p13.3	0.51	1.45E-34	2.62E-33
NCAM2	21q21.1	0.51	1.49E-34	2.68E-33
BTN3A3	6p22.2	0.51	1.72E-34	3.09E-33
AMZ1	7p22.3	0.51	1.73E-34	3.11E-33
RRAS	19q13.33	0.51	1.73E-34	3.11E-33
CCIN	9p13.3	0.51	1.78E-34	3.20E-33
NAALADL1	11q13.1	0.51	1.99E-34	3.58E-33
TM6SF2	19p13.11	0.51	2.74E-34	4.91E-33
ASXL3	18q12.1	0.51	2.82E-34	5.06E-33
FPR2	19q13.41	0.51	2.87E-34	5.14E-33
ITIH3	3p21.1	0.51	3.22E-34	5.76E-33
SLC2A3	12p13.31	0.51	3.23E-34	5.78E-33

NEGR1	1p31.1	0.51	3.35E-34	5.98E-33
GAS6	13q34	0.51	3.38E-34	6.03E-33
KCNAB2	1p36.31	0.51	3.48E-34	6.20E-33
ZNF154	19q13.43	0.51	3.54E-34	6.31E-33
PSAP	10q22.1	0.51	3.80E-34	6.77E-33
CCL7	17q12	0.51	4.03E-34	7.17E-33
GADD45B	19p13.3	0.51	4.38E-34	7.78E-33
RAB31	18p11.22	0.51	4.45E-34	7.90E-33
GP5	3q29	0.51	4.48E-34	7.95E-33
MS4A14	11q12.2	0.51	4.53E-34	8.02E-33
MMD	17q22	0.51	4.65E-34	8.22E-33
EDA2R	Xq12	0.51	5.64E-34	9.98E-33
PEA15	1q23.2	0.51	7.33E-34	1.29E-32
NETO1	18q22.3	0.51	7.36E-34	1.30E-32
GPX8	5q11.2	0.51	8.01E-34	1.41E-32
OTOA	16p12.2|16p1	0.51	8.59E-34	1.51E-32
ITGB7	12q13.13	0.50	1.06E-33	1.87E-32
MAN2B1	19p13.13	0.50	1.07E-33	1.88E-32
GBGT1	9q34.2	0.50	1.09E-33	1.92E-32
EHD1	11q13.1	0.50	1.12E-33	1.97E-32
PDLIM7	5q35.3	0.50	1.15E-33	2.02E-32
PDE4DIP	1q21.2	0.50	1.16E-33	2.03E-32
TRAT1	3q13.13	0.50	1.22E-33	2.14E-32
CD72	9p13.3	0.50	1.49E-33	2.61E-32
NAPSB	19q13.33	0.50	1.60E-33	2.79E-32
TREML1	6p21.1	0.50	2.04E-33	3.57E-32
SLFN11	17q12	0.50	2.07E-33	3.61E-32
PPM1M	3p21.2	0.50	2.22E-33	3.86E-32
DKC1	Xq28	-0.50	2.29E-33	4.00E-32
ATP6V0D2	8q21.3	0.50	2.35E-33	4.09E-32
BST1	4p15.32	0.50	2.37E-33	4.12E-32
KLRG1	12p13.31	0.50	2.38E-33	4.14E-32
WSCD1	17p13.2	0.50	2.50E-33	4.34E-32
PALD1	10q22.1	0.50	2.80E-33	4.86E-32
DCLK2	4q31.23-q31.3	0.50	2.82E-33	4.88E-32
SAMD4A	14q22.2	0.50	2.86E-33	4.95E-32
6-Sep	Xq24	0.50	3.12E-33	5.39E-32
INHBA-AS1	7p14.1	0.50	3.37E-33	5.82E-32
F2RL3	19p13.11	0.50	4.12E-33	7.12E-32
TNIP1	5q33.1	0.50	4.12E-33	7.12E-32
CD63	12q13.2	0.50	4.35E-33	7.50E-32
PCED1B	12q13.11	0.50	4.57E-33	7.87E-32
ZFP36L2	2p21	0.50	4.57E-33	7.87E-32
MT1M	16q13	0.50	4.68E-33	8.04E-32
