# Supplementary material for: Label-free quantitative identification of abnormally ubiquitinated proteins as useful biomarkers for human lung squamous cell carcinomas
Source: EPMA J. 2020 Jan 4;11(1):73–94. doi: 10.1007/s13167-019-00197-8 (PMC7028901; doi:10.1007/s13167-019-00197-8)
Supplement: Supplementary file 7 — (PDF 40 kb) [file 13167_2019_197_MOESM7_ESM.pdf]

Supplemental Table 5. Co-expressed genes of ABCC1 based on TCGA database of human LSCC.

Correlated Gene	Cytoband	Spearman's Correlation	p-Value	q-Value
ADAM23	2q33.3	0.692278885	9.98E-27	2.01E-22
GCLC	6p12.1	0.683199476	8.14E-26	6.07E-22
CDK5RAP2	9q33.2	0.682731355	9.05E-26	6.07E-22
MAP1B	5q13.2	0.674037082	6.27E-25	2.36E-21
MAFG	17q25.3	0.673739187	6.69E-25	2.36E-21
RBM19	12q24.13-q24.[illegible]	0.673502999	7.04E-25	2.36E-21
PTDSS1	8q22.1	0.671115584	1.18E-24	3.40E-21
ADH7	4q23	0.664863329	4.50E-24	1.13E-20
MDGA1	6p21.2	0.663097957	6.53E-24	1.46E-20
TSPAN7	Xp11.4	0.661702107	8.75E-24	1.75E-20
NTRK2	9q21.33	0.661285055	9.54E-24	1.75E-20
WNT5A	3p14.3	0.65562718	3.06E-23	5.14E-20
PGD	1p36.22	0.651450269	7.13E-23	1.10E-19
DIP2B	12q13.12	0.650539562	8.55E-23	1.23E-19
WASF1	6q21	0.644790192	2.67E-22	3.58E-19
SLC7A11	4q28.3	0.643562439	3.39E-22	4.27E-19
GLI2	2q14.2	0.641868694	4.71E-22	5.58E-19
PTGR1	9q31.3	0.639749385	7.09E-22	7.93E-19
SIPA1L2	1q42.2	0.636644896	1.28E-21	1.36E-18
CYP4F3	19p13.12	0.635446933	1.61E-21	1.62E-18
SOST	17q21.31	0.631629791	3.30E-21	3.16E-18
TXNRD1	12q23.3	0.628110208	6.33E-21	5.55E-18
ALDH3A1	17p11.2	0.628105953	6.34E-21	5.55E-18
G6PD	Xq28	0.624020538	1.34E-20	1.12E-17
SCN9A	2q24.3	0.620220251	2.65E-20	2.14E-17
SAMD12	8q24.11-q24.1[illegible]	0.619130807	3.22E-20	2.49E-17
AKR1C1	10p15.1	0.618169032	3.82E-20	2.85E-17
JAKMIP3	10q26.3	0.617720062	4.14E-20	2.98E-17
LRP8	1p32.3	0.616092279	5.52E-20	3.83E-17
CORO2A	9q22.33	0.613347391	8.94E-20	5.94E-17
CYP4F11	19p13.12	0.613217594	9.14E-20	5.94E-17
TRIM16L	17p11.2	0.611577045	1.22E-19	7.65E-17
RNF168	3q29	0.610766345	1.40E-19	8.54E-17
FAM102A	9q34.11	0.609664134	1.69E-19	1.00E-16
PRKX	Xp22.33	0.602480613	5.75E-19	3.31E-16
UGDH	4p14	0.600746439	7.69E-19	4.30E-16
TALDO1	11p15.5	0.596484415	1.56E-18	8.48E-16
MRAP2	6q14.2	0.595704886	1.77E-18	9.39E-16
UNKL	16p13.3	0.594554482	2.14E-18	1.10E-15
UGT1A6	2q37.1	0.593862941	2.40E-18	1.21E-15
UGT1A7	2q37.1	0.593028169	2.74E-18	1.35E-15
GCLM	1p22.1	0.591347857	3.60E-18	1.73E-15
SLC47A1	17p11.2	0.589681774	4.71E-18	2.21E-15
GPAT3	4q21.23	0.588428488	5.76E-18	2.64E-15
AKR1C2	10p15.1	0.588185916	5.99E-18	2.68E-15
MEGF9	9q33.2	0.587939089	6.23E-18	2.73E-15
FOXE1	9q22.33	0.586597683	7.72E-18	3.31E-15
GSTM4	1p13.3	0.586451743	7.90E-18	3.31E-15
RFFL	17q12	0.5857219	8.87E-18	3.60E-15
UGT1A9	2q37.1	0.585669045	8.95E-18	3.60E-15

SDK1	7p22.2	0.584181358	1.13E-17	4.47E-15
GSR	8p12	0.583483433	1.26E-17	4.87E-15
RIT1	1q22	0.583396193	1.28E-17	4.87E-15
ARHGEF26	3q25.2	0.582276959	1.53E-17	5.69E-15
AKR1B10	7q33	0.581857779	1.63E-17	5.97E-15
ARHGEF35	7q35	0.581459876	1.74E-17	6.18E-15
ABCC5	3q27.1	0.581413064	1.75E-17	6.18E-15
DNAJB3	2q37.1	0.581263004	1.79E-17	6.21E-15
DIAPH2	Xq21.33	0.581032184	1.86E-17	6.33E-15
TP63	3q28	0.579908695	2.21E-17	7.42E-15
CABYR	18q11.2	0.578961815	2.56E-17	8.32E-15
TTC22	1p32.3	0.57895756	2.56E-17	8.32E-15
STRBP	9q33.3	0.577710657	3.11E-17	9.93E-15
RNF217	6q22.31	0.576267995	3.88E-17	1.22E-14
PSMD11	17q11.2	0.572940084	6.45E-17	2.00E-14
SLC2A12	6q23.2	0.572682618	6.70E-17	2.04E-14
CZIB	1p32.3	-0.571959159	7.48E-17	2.25E-14
ZNF746	7q36.1	0.569720692	1.05E-16	3.10E-14
ME1	6q14.2	0.569512165	1.08E-16	3.16E-14
ATP2B1	12q21.33	0.567556698	1.45E-16	4.17E-14
CCSER1	4q22.1	0.565738777	1.90E-16	5.38E-14
CES1P1	16q12.2	0.565126542	2.08E-16	5.81E-14
F2RL2	5q13.3	0.562096712	3.24E-16	8.84E-14
GPX2	14q23.3	0.562060539	3.26E-16	8.84E-14
SLC47A2	17p11.2	0.561995939	3.29E-16	8.84E-14
RNF217-AS1	6q22.31	0.561610803	3.48E-16	9.23E-14
ALDH3A2	17p11.2	0.559962508	4.43E-16	1.16E-13
KIAA0319	6p22.3	0.559521283	4.72E-16	1.22E-13
OSGIN1	16q23.3	0.558673049	5.34E-16	1.36E-13
BICD2	9q22.31	0.558311319	5.63E-16	1.42E-13
UGT1A3	2q37.1	0.558166405	5.75E-16	1.42E-13
ADCY10	1q24.2	0.558136295	5.77E-16	1.42E-13
UROD	1p34.1	-0.556277123	7.54E-16	1.83E-13
CES1	16q12.2	0.55461104	9.57E-16	2.29E-13
UBE2H	7q32.2	0.553802468	1.07E-15	2.54E-13
SRXN1	20p13	0.553655648	1.10E-15	2.57E-13
SELENOI	2p23.3	0.553489678	1.12E-15	2.60E-13
GAB1	4q31.21	0.552840693	1.23E-15	2.82E-13
YWHAG	7q11.23	0.551302279	1.53E-15	3.46E-13
C1ORF50	1p34.2	-0.550319226	1.76E-15	3.93E-13
HRG	3q27.3	0.549542121	1.96E-15	4.34E-13
SLCO3A1	15q26.1	0.548795707	2.18E-15	4.77E-13
PANX2	22q13.33	0.547793504	2.51E-15	5.40E-13
STARD7	2q11.2	0.547748819	2.52E-15	5.40E-13
NDRG4	16q21	0.547623278	2.57E-15	5.44E-13
TRIM16	17p12	0.547282827	2.69E-15	5.64E-13
UBXN7	3q29	0.547174308	2.73E-15	5.67E-13
DMRT2	9p24.3	0.546185162	3.13E-15	6.44E-13
UGT1A1	2q37.1	0.545965183	3.23E-15	6.57E-13
SLC9A3R1	17q25.1	0.544723059	3.84E-15	7.72E-13
RAD23B	9q31.2	0.542378201	5.30E-15	1.06E-12
EIF4G1	3q27.1	0.541503667	5.97E-15	1.18E-12
BLMH	17q11.2	0.541352592	6.09E-15	1.19E-12
KIF13A	6p22.3	0.540665306	6.69E-15	1.30E-12

AKR1C3	10p15.1	0.540154629	7.17E-15	1.37E-12
HENMT1	1p13.3	-0.540107817	7.22E-15	1.37E-12
ADAM17	2p25.1	0.539639696	7.69E-15	1.45E-12
ODF2L	1p22.3	-0.538973688	8.42E-15	1.57E-12
HK1	10q22.1	0.538509824	8.97E-15	1.66E-12
PTPRD	9p24.1-p23	0.537973613	9.64E-15	1.77E-12
DCC	18q21.2	0.536800512	1.13E-14	2.05E-12
NFE2L2	2q31.2	0.535554281	1.34E-14	2.40E-12
MGMT	10q26.3	-0.53478401	1.48E-14	2.64E-12
TTBK2	15q15.2	0.533941393	1.66E-14	2.93E-12
GAPVD1	9q33.3	0.533760529	1.70E-14	2.97E-12
TMEM51	1p36.21	-0.532394468	2.03E-14	3.53E-12
SOX2	3q26.33	0.5314944	2.29E-14	3.93E-12
TMEM116	12q24.12-q24.	0.531458227	2.30E-14	3.93E-12
ZNF684	1p34.2	-0.530696467	2.55E-14	4.31E-12
ZNF280C	Xq26.1	0.529819806	2.86E-14	4.80E-12
TBL1X	Xp22.31-p22.2	0.529755971	2.88E-14	4.80E-12
DVL3	3q27.1	0.529162309	3.12E-14	5.12E-12
MSI2	17q22	0.529134647	3.13E-14	5.12E-12
ABHD4	14q11.2	0.529068685	3.15E-14	5.12E-12
F11R	1q23.3	0.528902715	3.22E-14	5.19E-12
OLFM1	9q34.3	0.52853673	3.38E-14	5.40E-12
KLHL35	11q13.4	-0.528364376	3.46E-14	5.48E-12
NSRP1	17q11.2	0.526855752	4.21E-14	6.62E-12
FREM2	13q13.3	0.526753616	4.27E-14	6.66E-12
SMO	7q32.1	0.526636586	4.33E-14	6.71E-12
GSTM2	1p13.3	0.526423804	4.45E-14	6.80E-12
MFHAS1	8p23.1	0.526421677	4.46E-14	6.80E-12
NHSL1	6q24.1	0.524555578	5.67E-14	8.59E-12
RRM1	11p15.4	0.52413427	5.99E-14	9.00E-12
ADCY8	8q24.22	0.522600056	7.30E-14	1.09E-11
QPCT	2p22.2	-0.522219231	7.67E-14	1.13E-11
PCYT1A	3q29	0.522214976	7.67E-14	1.13E-11
UGT1A4	2q37.1	0.521988565	7.90E-14	1.15E-11
MTMR1	Xq28	0.521770261	8.12E-14	1.18E-11
PCYT1B	Xp22.11	0.52051272	9.53E-14	1.37E-11
MT1G	16q13	-0.520359517	9.72E-14	1.39E-11
ZFAND2B	2q35	-0.520157374	9.98E-14	1.41E-11
NAV1	1q32.1	0.520155246	9.98E-14	1.41E-11
PTCH1	9q22.32	0.519131764	1.14E-13	1.59E-11
PSMD2	3q27.1	0.518727478	1.20E-13	1.66E-11
YEATS2	3q27.1	0.518561508	1.22E-13	1.69E-11
COLGALT2	1q25.3	0.517363546	1.42E-13	1.95E-11
TXN	9q31.3	0.517106079	1.47E-13	2.00E-11
CCL28	5p12	-0.516806057	1.53E-13	2.06E-11
TUFT1	1q21.3	0.516414538	1.60E-13	2.15E-11
GPR107	9q34.11	0.515588943	1.78E-13	2.37E-11
HRNR	1q21.3	0.515360235	1.83E-13	2.42E-11
RAD54L2	3p21.2	0.515267643	1.85E-13	2.44E-11
DIRAS2	9q22.2	0.512432053	2.64E-13	3.45E-11
MMACHC	1p34.1	-0.511941859	2.80E-13	3.64E-11
LRRC4	7q32.1	0.511701416	2.89E-13	3.72E-11
ATP2A2	12q24.11	0.511639709	2.91E-13	3.73E-11
FXR1	3q26.33	0.511060942	3.12E-13	3.98E-11

CLDN20	6q25.3	0.510797145	3.23E-13	4.09E-11
SLC20A2	8p11.21	0.51051622	3.34E-13	4.19E-11
ABCB6	2q35	0.510465152	3.36E-13	4.19E-11
GCNT2	6p24.3-p24.2	0.510435363	3.37E-13	4.19E-11
CYP26A1	10q23.33	0.510306651	3.43E-13	4.24E-11
ABCC3	17q21.33	0.509571468	3.75E-13	4.61E-11
FNDC8	17q12	0.50947146	3.80E-13	4.64E-11
TMTC1	12p11.22	0.507786226	4.67E-13	5.67E-11
PYCR2	1q42.12	0.507303211	4.96E-13	5.96E-11
KIAA1549L	11p13	0.507271294	4.98E-13	5.96E-11
KAT7	17q21.33	0.506556346	5.43E-13	6.47E-11
LRRC8D	1p22.2	0.506283985	5.61E-13	6.65E-11
EPS8	12p12.3	0.505875444	5.90E-13	6.95E-11
KIAA1671	22q11.23	0.505456263	6.21E-13	7.27E-11
CORO1C	12q24.11	0.504430654	7.03E-13	8.18E-11
EGF	4q25	0.503919977	7.48E-13	8.65E-11
ACTR1A	10q24.32	-0.503830608	7.56E-13	8.69E-11
ACSF2	17q21.33	-0.503156089	8.20E-13	9.38E-11
PKP1	1q32.1	0.502115585	9.29E-13	1.06E-10
TNK2	3q29	0.501785773	9.66E-13	1.09E-10
BTBD11	12q23.3	0.501585758	9.90E-13	1.11E-10
SPATA7	14q31.3	-0.50101763	1.06E-12	1.19E-10
FLYWCH1	16p13.3	0.500647389	1.11E-12	1.23E-10
TMEM158	3p21.31	0.50030481	1.15E-12	1.28E-10
FAM120A	9q22.31	0.500164374	1.17E-12	1.29E-10
