# Supplementary material for: Label-free quantitative identification of abnormally ubiquitinated proteins as useful biomarkers for human lung squamous cell carcinomas
Source: EPMA J. 2020 Jan 4;11(1):73–94. doi: 10.1007/s13167-019-00197-8 (PMC7028901; doi:10.1007/s13167-019-00197-8)
Supplement: Supplementary file 8 — (PDF 55 kb) [file 13167_2019_197_MOESM8_ESM.pdf]

Supplemental Table 6. The biological process (BP) of co-expressed genes of VIM.

GO_ID	GO Term_Description	PValue	Enriched Genes	FDR
GO:0007155	cell adhesion	7.32E-37	POSTN, CXCL12, GP5, S1PR1, FAP, TGFBI, CLEC4A, COL12A1, SPON2, SPON1, MFGE8, SSPN, SIRPA, THY1, TNFAIP6, NCAM2, CCR8, PGM5, CD34, LSAMP, CD33, RELN, COL1A1, TGFB1I1, MFAP4, CD226, ITGAL, PLXNC1, SCN1B, CYP1B1, CCL2, ITGA11, IL32, ITGB2, ITGB3, CD72, ITGAM, ITGBL1, ITGAX, ITGB7, ENTPD1, FN1, COL18A1, PODXL, ITGA4, EMILIN2, COL16A1, MCAM, SLAMF1, GAS6, PCDH18, EMILIN1, LAMA2, ITGA9, OMD, LYVE1, LAMA4, CASS4, ITGA5, CD300A, ITGA8, ENG, NRP2, IGFBP7, CXCR3, DDR2, ZYX, LOXL2, PARVG, ICAM1, ADGRE2, ICAM2, ADGRE5, ICAM3, SIGLEC10, SIGLEC14, SIGLEC5, SIGLEC7, SUSD5, VCAN, AOC3, CCR1, FPR2, SCARF1, CDH5, SIGLEC9, VCAM1, ISLR, LPXN, SORBS1, COMP, COL6A3, COL6A2, CD2, COL6A1, CD4, COL8A1, THBS1, THBS2, SELPLG, DPT, HAPLN3, OLR1, COL15A1, NID2, COL5A1, STAB1, PECAM1, THEMIS2, NTM, CDH11	1.34E-33
GO:0030198	extracellular matrix organization	9.30E-33	POSTN, DDR2, TGFBI, LOX, COL11A1, COL10A1, RECK, ICAM1, MATN3, ICAM2, OLFML2B, ICAM3, BGN, COL1A2, VCAN, COL1A1, JAM2, JAM3, ITGAL, ADAMTSL2, SPOCK2, LUM, COL3A1, ITGA11, ITGB2, DCN, ITGB3, ITGAM, ABI3BP, VCAM1, ITGAX, CRISPLD2, ITGB7, COMP, COL6A3, COL6A2, COL6A1, COL8A1, THBS1, COL8A2, FN1, COL18A1, COL4A2, COL4A1, COL13A1, FBN1, CCDC80, ITGA1, NID2, ITGA4, SPARC, COL5A3, COL16A1, ECM2, COL5A2, COL5A1, EMILIN1, KDR, LAMA2, CSGALNACT1, ITGA9, LAMA4, COL14A1, ITGA5, FBLN5, ITGA8, PECAM1, APBB2	1.70E-29

GO:0006954	inflammatory response	4.08E-25	RARRES2, TSPAN2, AIF1, LY86, PTGS1, TLR4, CXCR3, TLR7, ADORA1, CXCL12, IL10, TLR8, CCRL2, S1PR3, NLRC4, PTGIR, CXCR4, SEMA7A, TICAM2, ADAM8, TNIP1, CIITA, PIK3CG, PTGER2, C5AR2, C5AR1, ADGRE2, PTGER3, ADGRE5, PIK3CD, LYZ, NFAM1, NLRP3, SIGLEC1, TNFRSF9, TNFAIP6, KLRG1, CCR5, CCR4, CD40LG, CCR2, PTGDR, PTAFR, AOC3, C3AR1, ITGAL, CCL2, C3, ADORA2A, CCR1, CSF1, FPR1, TNFRSF8, ITGB2, FPR3, GPR68, FPR2, TNFRSF4, CCL7, SLC11A1, TNFRSF1B, CCL23, FOLR2, CCL21, THBS1, CSF1R, NOX4, HAVCR2, IL2RA, OLR1, AXL, PTGFR, CCL18, CD180, GGT5, CYBB, CCL13, P2RX7, STAB1, AOX1, TBXA2R, THEMIS2, CD14, F2R	7.48E-22
GO:0006955	immune response	3.51E-23	AQP9, IL16, TLR4, ZEB1, TNFSF12, HLA-DMB, HLA-DMA, C1QC, CXCL12, IL10, SEMA7A, S1PR4, FCGR3A, SPN, CIITA, C5AR1, CMKLR1, ADGRE5, NCF4, GEM, CTSS, WAS, HLA-DQA2, HLA-DQA1, PDCD1LG2, IGSF6, TNFRSF9, LILRB2, CCR8, CD86, CCR6, TNFSF13B, CCR5, CCR4, CD40LG, CST7, CCR2, HLA-DPA1, TNFSF12-TNFSF13, PTAFR, HLA-DRA, LCP2, HLA-DQB1, HLA-DQB2, GPR183, IL1R1, CCL2, LST1, HLA-DRB1, C3, ENPP2, IFITM2, CCR1, GPR65, TNFRSF8, C1R, IL32, IL7R, TNFRSF4, CD74, SLC11A1, TNFRSF1B, CCL23, CCL21, HRH2, FCGR1A, ICOS, FCGR1B, HLA-DRB5, CD4, IL2RG, HLA-DPB1, HLA-DOA, THBS1, TNFSF4, IL2RA, CTLA4, SAMHD1, CCL18, TNFSF8, CCL13, RGS1, FCGR2B, FCGR2C, ETS1, IRF8	6.43E-20
GO:0050900	leukocyte migration	2.59E-19	ITGAL, C3AR1, MMP9, FPR1, FPR3, ITGB2, JAML, FPR2, ITGB3, CD74, ITGAM, SLC7A7, CD48, ITGAX, TEK, CD2, FCER1G, ESAM, INPP5D, SELPLG, SPN, FN1, ICAM1, C5AR1, OLR1, PODXL, ITGA4, SIRPA, GAS6, CD84, DOK2, CD34, ITGA5, FYN, LCK, PECAM1, COL1A2, COL1A1, TREM1, JAM2, JAM3	4.74E-16

GO:0007165 signal transduction	6.51E-19	DLC1, F2RL3, PLPPR4, IQGAP2, CD53, TNFSF12, PRKG1, SHE, CXCL12, ADORA1, CD48, PGR, LILRA2, ANK2, UNC5B, STARD8, UNC5C, SPN, PAG1, C5AR1, PTPRM, PIK3CD, PKIG, GEM, TRAT1, PLAUR, LILRB1, VEGFC, LILRB2, TNFAIP6, CCR6, ARRB1, CD34, DLL4, CD33, LILRB4, PDGFRB, CLEC5A, CD226, NEK6, ITGAL, CCL2, GNAI2, CYTL1, ELK3, CD72, GREM1, CD74, CCL7, VDR, TAGAP, IL12RB1, RAC2, CD69, TEK, RASAL3, ADRA2A, HLA-DOA, CSF1R, TYROBP, MRC1, ITK, LGALS1, MRC2, CD300C, SLAMF1, GAS6, LYVE1, RGS1, P2RX1, CD300A, HIVEP3, ANTXR1, FGF7, NRP1, ACVRL1, ARHGAP15, DDR2, SIRPB1, EDNRA, ARHGAP6, EVI2A, GUCY1A2, CSF2RB, MICAL1, ZYX, TIE1, ARHGAP9, PILRA, RSU1, MPP1, STX2, NFAM1, NLRP3, ARHGAP25, PRKCB, ARHGAP30, ARHGAP31, DOK2, TNFSF13B, MAPRE2, TNFSF12-TNFSF13, RIN3, PLPP4, C3, SPOCK2, FPR1, TNFRSF8, FPR3, GNG11, IL7R, APBB1IP, SCARF1, STARD13, LPXN, CCL23, PDE1B, FCGR1A, PDE1A, SH2B3, CD4, IL2RG, INPP5D, RASA3, HPGDS, GUCA1A, IL2RB, TNFSF4, COL15A1, AXL, DPYSL2, SPARC, ANXA5, CCL18, ITPR1, ITPR2, TNFSF8, SH3BP5, LSP1, RASSF4, CCL13, ABCC9, FCGR2B,	1.19E-15
GO:0030574 collagen catabolic process	9.52E-18	ADAMTS14, MMP9, COL3A1, MMP2, COL6A3, COL6A2, COL12A1, COL6A1, COL8A1, COL11A1, COL8A2, COL10A1, COL18A1, COL4A2, COL4A1, COL13A1, MRC2, MMP19, COL15A1, MMP16, CTSS, MMP14, COL5A3, COL5A2, COL5A1, CTSK, COL1A2, COL1A1, ADAMTS2	1.74E-14
GO:0001525 angiogenesis	5.76E-16	NRP2, NRP1, ACVRL1, ANPEP, CXCR3, ENPEP, TNFSF12, MMP2, GJA5, MMRN2, S1PR1, UNC5B, FAP, TGFBI, ROBO4, TIE1, SOX17, ADAM8, PIK3CG, FMNL3, MMP19, MFGE8, MMP14, VASH1, THY1, VEGFC, CLIC4, DLL4, PLXDC1, TNFSF12-TNFSF13, JAM3, CCL2, CYP1B1, ELK3, TEK, PIK3R6, PLXND1, COL8A1, COL8A2, FN1, COL18A1, PTPRB, COL4A2, FLT1, COL15A1, MCAM, KDR, MEOX2, ITGA5, ECSCR, PECAM1	1.02E-12
GO:0006935 chemotaxis	3.38E-14	C3AR1, RARRES2, CCL2, CYSLTR1, ENPP2, CCR1, FPR1, FPR2, CXCR3, CXCL12, CCL7, CCRL2, DOCK2, S1PR1, CCL23, RAC2, CXCR4, SPN, C5AR2, RNASE2, C5AR1, CMKLR1, NCKAP1L, CCL18, PLAUR, LSP1, CCR8, CCL13, CCR6, CCR5, CCR4, CCR2, ECSCR, CMTM3, PTAFR	6.19E-11

GO:0007160	cell-matrix adhesion	4.14E-14	PPFIA2, ITGAL, FERMT2, COL3A1, ITGA11, ITGB2, ITGB3, VCAM1, SORBS1, ITGB7, ILK, TNN, ZYX, ADAMTS12, PARVG, COL13A1, ITGA1, ITGA4, NID2, CD63, ECM2, COL5A3, SIGLEC1, LYVE1, SNED1, CD34, FBLN5, ITGA8, JAM3, OTOA	7.59E-11
GO:0030199	collagen fibril organization	1.65E-13	FMOD, CYP1B1, ADAMTS14, LUM, COL3A1, COL5A3, GREM1, DDR2, COL5A2, COL5A1, COL14A1, SFRP2, COL1A2, COL12A1, COL1A1, LOX, LOXL2, ADAMTS2, COL11A1, DPT	3.03E-10
GO:0031295	T cell costimulation	3.49E-13	HLA-DQB1, HLA-DQB2, HLA-DRB1, CCL21, ICOS, HLA-DRB5, CD4, HLA-DPB1, CD5, SPN, DPP4, CD28, CD3E, LGALS1, CTLA4, HLA-DQA2, PDCD1LG2, HLA-DQA1, CD86, TNFSF13B, CD80, CD40LG, FYN, LCK, HLA-DPA1, GRAP2, HLA-DRA	6.40E-10
GO:0050776	regulation of immune response	9.35E-12	ITGAL, C3, COL3A1, ITGB2, JAML, SIGLEC9, VCAM1, LILRA1, FCGR1A, ITGB7, OSCAR, FCGR3A, TYROBP, ICAM1, LAIR1, CD3E, ICAM2, ICAM3, SLAMF6, CD300E, CD300C, ITGA4, HCST, LILRB1, LILRB2, FCGR2B, CD300A, CD34, CD40LG, CD33, SIGLEC7, COL1A2, CD300LF, COL1A1, TREM1, TREML1, TREM2, CD300LB, CD226	1.71E-08
GO:0007204	positive regulation of cytosolic calcium ion concentration	1.82E-11	MCHR1, C3AR1, CYSLTR1, CCR1, PTH1R, FPR1, FPR3, CD52, FPR2, CXCR3, EDNRA, S1PR3, AGTR1, PTGIR, CXCR4, C1QTNF1, S1PR4, PIK3CG, C5AR2, PTGER2, PTGER3, C5AR1, PTGFR, CCR8, CCR6, CCR5, CCR4, CCR2, PTGDR, PDGFRA, TBXA2R, CACNA1C, F2R	3.34E-08
GO:0007229	integrin-mediated signaling pathway	1.50E-10	ITGAL, FGR, FERMT3, FERMT2, COL3A1, ITGA11, ITGB2, ITGB3, ITGAM, ITGBL1, DAB2, ITGAX, ITGB7, SEMA7A, ILK, ADAMTS10, FCER1G, ZYX, ADAM8, TYROBP, PLEK, ITGA1, ITGA4, COL16A1, ITGA9, ITGA5, ITGA8	2.75E-07
GO:0042102	positive regulation of T cell proliferation	1.86E-10	HAVCR2, PTPRC, TNFSF4, AIF1, CD3E, IL6ST, NCKAP1L, HLA-DMB, PDCD1LG2, VCAM1, LILRB2, CORO1A, TNFSF13B, CD40LG, HLA-DPA1, CD4, JAK3, HLA-DPB1, SPN, SASH3, CD28	3.41E-07
GO:0045766	positive regulation of angiogenesis	2.04E-10	C3AR1, ACVRL1, CYP1B1, C3, ITGB2, CXCR3, TNFSF12, GREM1, PTGIS, GATA6, CCBE1, TEK, RRAS, PIK3R6, THBS1, FLT1, C5AR1, TGFBR2, HGF, KDR, PRKCB, VEGFC, CYBB, SFRP2, ETS1, CD34, TBXA2R, TNFSF12-TNFSF13, ENG	3.75E-07
GO:0002504	antigen processing and presentation of peptide or polysaccharide antigen via MHC class II	3.82E-10	HLA-DQB1, HLA-DQB2, HLA-DRB1, HLA-DRB5, HLA-DPA1, HLA-DPB1, HLA-DMB, HLA-DOA, HLA-DMA, HLA-DQA2, HLA-DQA1, HLA-DRA	7.00E-07

GO:0045087	innate immune response	6.59E-10	LY86, TLR4, TLR7, C1QC, TLR8, SIRPB1, BTK, MARCO, NLRC4, TMEM173, CLEC4E, LILRA5, TICAM2, CLEC4A, CLEC4D, SPON2, PIK3CG, NCF2, NCF1, PIK3CD, SERPING1, COLEC12, NLRP3, SIGLEC14, SIGLEC15, CD84, C1QA, C1QB, KLRG1, LCK, TREM1, TREM2, SSC5D, CD300LB, CLEC5A, TNFAIP8L2, FGR, CSF1, C1R, C1S, CLEC10A, FCER1G, C2, TYROBP, CSF1R, HAVCR2, ITK, CR1, ADARB1, SLAMF6, AXL, CD300E, SLAMF1, CD180, CYBB, CORO1A, FYN, CD209, JAK1, JAK3, TREML1, CD14	1.21E-06
GO:0043547	positive regulation of GTPase activity	9.84E-10	DLC1, A2M, FGF7, PREX1, RASGEF1B, ARHGAP18, ARHGAP15, PTGIR, ARHGAP6, SMAP2, S1PR1, STARD8, ARHGEF40, CSF2RB, DOCK10, AGAP2, DOCK11, CSF2RA, ARHGAP9, ICAM1, RSU1, ARHGEF6, ARHGEF17, ARHGEF15, ARHGAP25, THY1, ARHGAP30, ARHGAP31, SH2D3C, RASGRF2, ARRB1, ACAP1, PDGFRA, PDGFRB, RIN3, FGD2, CCL2, CYTH4, DENND2A, CCL7, STARD13, DOCK2, TAGAP, CCL23, RASGRP3, RASGRP4, TEK, RASAL3, IL2RG, FGD5, IQSEC3, RASA3, CAMK2A, IQSEC1, ARHGDIB, IL2RB, IL2RA, NCKAP1L, RGS18, DOCK8, RGS16, CCL18, DOCK4, CCL13, RGS1, ADAP2, FYN, RGS3, RGS4, CHN1, TBXA2R, JAK1, JAK3, IL3RA	1.80E-06
GO:0030168	platelet activation	1.03E-09	F2RL3, COL3A1, ITGB3, GP5, RAC2, RHOA, ADRA2A, FCER1G, GNG2, PIK3R5, PIK3R6, PIK3CG, AXL, GAS6, ITPR1, PRKCB, ITPR2, F5, P2RX1, CD40LG, ARRB1, FYN, LCK, COL1A2, COL1A1, TREML1, LCP2, F2R	1.89E-06
GO:0007166	cell surface receptor signaling pathway	1.22E-09	MCHR1, IL1R1, CCL2, TSPAN2, CYSLTR1, CCR1, TSPAN4, PTH1R, ADGRF5, CD53, CXCR3, IL7R, GPR88, SIRPB1, SIGLEC9, MARCO, TSPAN11, ASGR2, LILRA1, CD2, CLEC4A, CD4, SPN, CD28, PTPRC, ADGRE2, IL2RA, CD3E, ADGRE5, NPR1, GEM, IGSF6, CLEC1A, IFNAR2, INHBA, LILRB2, P2RX7, DOK2, CD37, KLRG1, CCR5, LILRB5, LILRB3, ADGRA2, ADGRL4, CD14	2.23E-06
GO:0002250	adaptive immune response	1.25E-09	GPR183, CLEC10A, BTK, LILRA1, LILRA2, FCGR1B, CLEC4A, LILRA6, CD4, CLEC4D, PAG1, HAVCR2, PIK3CG, ITK, LAIR1, PIK3CD, CTLA4, CTSS, TRAT1, PRKCB, LILRB1, CD84, LILRB2, LAT2, CD86, LILRB5, FYN, LILRB3, CD209, LILRB4, JAK3, JAM3	2.29E-06

GO:0030335	positive regulation of cell migration	6.24E-09	FGR, AIF1, FERMT3, CCR1, CSF1, LRRC15, CCL7, DAB2, S1PR1, SEMA7A, ILK, ADRA2A, THBS1, CSF1R, COL18A1, F10, FLT1, PODXL, PIK3CD, HGF, MCAM, MMP14, SNAI1, MYADM, KDR, TNFAIP6, SEMA6B, CORO1A, ITGA5, PDGFRA, PDGFRB, HAS2, COL1A1, MYLK, F2R	1.14E-05
GO:0007169	transmembrane receptor protein tyrosine kinase signaling pathway	1.17E-08	ITK, FLT1, FGR, FLT3, CD3E, FLT4, CSF1, TRAT1, KDR, BTK, DOK2, RASGRP4, DOK5, FYN, LCK, TEK, ROR1, JAK1, ANGPTL1, CD4, PAG1, PILRA, LCP2, CSF1R	2.14E-05
GO:0035987	endodermal cell differentiation	1.94E-08	COL4A2, MMP9, ITGB2, ITGA4, MMP14, MMP2, INHBA, ITGA5, COL6A1, COL12A1, COL8A1, COL11A1, FN1	3.55E-05
GO:0070374	positive regulation of ERK1 and ERK2 cascade	2.25E-08	GPR183, CCL2, NRP1, CCR1, PTPN22, TLR4, SCIMP, CD74, CCL7, GLIPR2, CCL23, CCL21, SEMA7A, TEK, CSF1R, HAVCR2, NOX4, ICAM1, C5AR2, C5AR1, FLT4, SLAMF1, GAS6, CCL18, KDR, CCL13, ARRB1, PDGFRA, PDGFRB, TREM2, PLA2G5, F2R, HTR2A	4.13E-05
GO:0008360	regulation of cell shape	8.98E-08	RHOJ, FMNL1, DLC1, FGD2, ICAM1, FMNL3, CCL2, LST1, PALM2-AKAP2, FGR, ATP10A, FERMT2, ARHGAP18, ITGB2, ARHGAP15, TPM1, CCL7, KDR, SYNE3, CCL13, CORO1A, FYN, SH3KBP1, PLEKHO1, WIPF1, FGD5, FN1, CSF1R	1.65E-04
GO:0022617	extracellular matrix disassembly	1.05E-07	SH3PXD2B, A2M, MMP9, FBN1, MMP19, MMP16, CTSS, DCN, TIMP2, MMP14, MMP2, TIMP1, CTSK, HTRA1, ADAM8, ENG, ADAMTS5, LCP1, FN1, ADAMTS4	1.92E-04
GO:0006968	cellular defense response	1.14E-07	ITK, C5AR1, NCF2, NCF1, ADORA2A, CD300C, TRAT1, LSP1, LILRB2, KLRG1, CCR6, CCR5, FCMR, CCR2, MNDA, CLEC5A, SPN, TYROBP	2.09E-04
GO:0016525	negative regulation of angiogenesis	1.14E-07	COL4A2, CCL2, PTPRM, NPR1, SPARC, DCN, CXCR3, VASH1, GPR4, STAB1, CCR2, TEK, RGCC, ECSCR, SULF1, TIE1, THBS1, THBS2	2.09E-04
GO:0060333	interferon-gamma-mediated signaling pathway	1.80E-07	HLA-DQB1, CIITA, ICAM1, HLA-DQB2, HLA-DRB1, IFI30, HLA-DQA2, HLA-DQA1, VCAM1, FCGR1A, FCGR1B, IRF8, HLA-DRB5, JAK1, HLA-DPA1, HLA-DPB1, CAMK2A, PTAFR, HLA-DRA	3.29E-04
GO:0060326	cell chemotaxis	2.42E-07	C3AR1, CCL2, C5AR1, FPR1, FPR3, FPR2, HGF, CXCL12, DOCK4, VCAM1, AGTR1, CCL13, CCR6, CCL21, PDGFRA, PDGFRB, ENG, BIN2	4.43E-04
GO:0050852	T cell receptor signaling pathway	2.93E-07	HLA-DQB1, HLA-DQB2, HLA-DRB1, PTPN22, HLA-DRB5, CD4, INPP5D, HLA-DPB1, PAG1, CD28, PTPRC, ITK, CD3E, PIK3CD, RFTN1, FOXP3, WAS, HLA-DQA2, HLA-DQA1, TRAT1, THY1, FYN, LCK, HLA-DPA1, GRAP2, THEMIS2, LCP2, HLA-DRA	5.37E-04

GO:0032729	positive regulation of interferon-gamma production	3.64E-07	HAVCR2, TNFSF4, CD3E, SLAMF6, TLR4, SLC11A1, IL12RB1, CCR2, IRF8, TICAM2, HLA-DPA1, HLA-DPB1, CD226, SASH3, CD14	6.68E-04
GO:0043542	endothelial cell migration	5.39E-07	CYP1B1, FAP, PECAM1, RHOA, ITGB2, TNFSF12-TNFSF13, TNFSF12, PLXND1, LOXL2, ADGRA2, DPP4, STARD13	9.88E-04
GO:0016477	cell migration	6.09E-07	CTHRC1, FGR, CD248, PEAK1, ITGB3, ENPEP, SDC3, S1PR1, GPC6, RHOA, TNN, ADAMTS12, NFATC2, THBS1, FMNL3, FLT1, ADGRE2, PODXL, CD63, SNAI1, COL5A1, GAS6, TNS3, FYN, SH3KBP1, JAK1, PDGFRB, JAK3, ENG, LCP1	1.12E-03
GO:0006909	phagocytosis	6.53E-07	ITGAL, ADORA2A, ICAM3, AXL, ITGB2, ADORA1, SLAMF1, GAS6, SLC11A1, CORO1A, CD93, IRF8, PECAM1, CEACAM4, CD14	1.20E-03
GO:0001501	skeletal system development	7.96E-07	AEBP1, MMP9, COL3A1, PTH1R, POSTN, EXTL1, GJA5, VDR, COMP, COL12A1, PAPSS2, COL10A1, SH3PXD2B, MATN3, HAPLN3, CMKLR1, FBN1, FRZB, COL5A2, PRELP, RASSF2, COL1A2, VCAN, COL1A1, CDH11, ADAMTS4	1.46E-03
GO:0002576	platelet degranulation	9.20E-07	RARRES2, A2M, PLEK, PSAP, F13A1, TGFB3, SERPING1, HGF, ITGB3, SPARC, CD63, TIMP3, GAS6, TIMP1, ISLR, VEGFC, F5, PECAM1, ITIH3, THBS1, SRGN, FN1	1.69E-03
GO:0034446	substrate adhesion-dependent cell spreading	1.61E-06	FERMT3, PEAK1, FERMT2, AXL, ITGB3, ITGA4, FZD4, ITGA8, ITGB7, TEK, ILK, ANTXR1, FN1	2.95E-03
GO:0006952	defense response	2.04E-06	CYSLTR1, RNASE6, IL32, COLEC12, NLRP3, CXCL12, WAS, CD74, CLEC1A, CD48, CD84, INHBA, LILRA1, LILRA2, LILRB5, LILRB3, TNIP1	3.74E-03
GO:0032715	negative regulation of interleukin-6 production	3.40E-06	HAVCR2, IRAK3, ARRB1, NCKAP1L, TLR4, HGF, KLF2, FOXP3, SLAMF1, IL10, GAS6	6.23E-03
GO:0032689	negative regulation of interferon-	3.40E-06	HAVCR2, LILRB1, TNFSF4, HLA-DRB1, AXL, HLA-DRB5, TLR4,	6.23E-03
GO:0071222	cellular response to lipopolysaccharide	4.41E-06	HAVCR2, MRC1, MEF2C, ICAM1, CCL2, TNFSF4, AXL, TLR4, NLRP3, IL10, CD180, LILRB1, LILRB2, TNFRSF1B, CD86, CD80, CCR5, IRF8, TICAM2, TBXA2R, SPON2, CD14	8.09E-03
GO:0070098	chemokine-mediated signaling pathway	4.61E-06	CCL2, CMKLR1, CCR1, CXCR3, CXCL12, CCL18, CCL7, CCRL2, CCR8, CCL13, CCL23, CCR6, CCR5, CXCR4, CCL21, CCR4, CCR2	8.45E-03
GO:0097190	apoptotic signaling pathway	4.61E-06	CD3E, TNFRSF8, TLR4, TNFSF12, TNFRSF4, ADORA1, BTK, ANXA6, P2RX7, TNFRSF1B, PTGIS, TICAM2, TNFSF12-TNFSF13, CD5, CD14, SPN, CD28	8.45E-03
GO:0046718	viral entry into host cell	5.49E-06	MRC1, ICAM1, CR1, AXL, ANPEP, ITGB3, TNFRSF4, SLAMF1, GAS6, CD86, CD80, ITGA5, ITGB7, CD209, SELPLG, CLEC5A, DPP4, HTR2A	1.01E-02

GO:0048010	vascular endothelial growth factor receptor signaling pathway	5.60E-06	NRP2, NRP1, FLT1, CCL2, NCF2, NCF1, NCF4, FLT4, AXL, NCKAP1L, ITGB3, KDR, VEGFC, CYBB, FYN, SULF1, RHOA	1.03E-02
GO:0007267	cell-cell signaling	5.72E-06	NRP1, GRAP, ADORA2A, CCR1, ITGB2, ENPEP, GJA4, GREM1, ADORA1, IL10, CCL7, PGR, WNT2, PTGIR, CCL23, CCL21, TEK, ZYX, ADGRE5, TBX5, CCL18, TNFSF8, C1QA, INHBA, TNFAIP6, LILRB2, SSTR2, CCL13, CD86, CCR5, CD80, SFRP2, STAB1, CD33, SH3KBP1, GRAP2	1.05E-02
GO:0014068	positive regulation of phosphatidylinositol 3-kinase signaling	6.68E-06	FLT1, FGR, FLT3, HGF, DCN, KDR, HCST, UNC5B, FYN, TEK, PDGFRA, PDGFRB, RELN, AGAP2, CD28, F2R	1.22E-02
GO:0050729	positive regulation of inflammatory response	6.76E-06	LPL, CCL2, TNFSF4, TLR4, TLR7, CCL18, CCL7, AGTR1, CCL13, CCL23, PDE2A, ETS1, CCR2, TGM2, PLA2G7, NLRP12, TNIP1	1.24E-02
GO:0030334	regulation of cell migration	8.15E-06	PLXNC1, PLXNA4, ENPP2, ABI3, LDB2, ITGB3, NEXN, THY1, LAMA2, LAMA4, CXCR4, PECAM1, ROBO4, RHOA, UNC5C, DOCK10, PLXND1	1.49E-02
GO:0030593	neutrophil chemotaxis	8.16E-06	PIK3CG, C5AR1, CCL2, PREX1, PIK3CD, ITGA1, NCKAP1L, ITGB2, JAML, CCL7, CCL18, ITGA9, CCL13, CCL23, FCER1G, TREM1	1.49E-02
GO:0007159	leukocyte cell-cell adhesion	9.83E-06	VCAM1, ICAM1, ITGAL, ITGA5, CD40LG, FERMT3, CD209, ITGB2, ITGA4, TNIP1	1.80E-02
GO:0019886	antigen processing and presentation of exogenous peptide antigen via MHC class II	1.00E-05	HLA-DQB1, HLA-DQB2, HLA-DRB1, LGMN, IFI30, CTSS, HLA-DMB, HLA-DMA, HLA-DQA2, CD74, HLA-DQA1, AP1S2, FCER1G, HLA-DRB5, HLA-DPA1, HLA-DPB1, HLA-DOA, SEC24D, HLA-DRA	1.83E-02
GO:0016337	single organismal cell-cell adhesion	1.04E-05	ICAM1, LIMS2, COL13A1, ICAM2, ICAM3, CDH5, THY1, VCAM1, SIGLEC1, COL14A1, CD93, CD34, ICOS, ITGA8, CD2, ESAM, JAM2, ADAM8, NEGR1, COL8A2	1.90E-02
GO:0032720	negative regulation of tumor necrosis factor production	1.13E-05	HAVCR2, IRAK3, C5AR2, CD34, AXL, ACP5, PTPN22, TLR4, FOXP3, SLAMF1, IL10, GAS6	2.06E-02
GO:0035025	positive regulation of Rho protein signal transduction	1.42E-05	P2RY8, F2RL3, P2RY10, ARRB1, COL3A1, GPR65, LPAR4, PDGFRB, GPR4, F2R	2.61E-02
GO:0050853	B cell receptor signaling pathway	1.68E-05	MEF2C, PTPRC, PIK3CD, CTLA4, NCKAP1L, NFAM1, RFTN1, PRKCB, BTK, LAT2, KLHL6, LCK, MNDA, NFATC2	3.07E-02
GO:0010759	positive regulation of macrophage chemotaxis	1.85E-05	C3AR1, RARRES2, CCL2, C5AR1, CMKLR1, CSF1, THBS1	3.38E-02
GO:0018108	peptidyl-tyrosine phosphorylation	1.96E-05	FGF7, FGR, STAT5A, PEAK1, ABI3, DDR2, BTK, IL12RB1, TEK, CSF2RB, TIE1, CSF2RA, CSF1R, FLT1, FLT3, FLT4, AXL, KDR, FYN, ROR1, PDGFRA, PDGFRB, RELN, JAK3, IL3RA	3.59E-02

GO:0019882	antigen processing and presentation	2.07E-05	HLA-DQB1, RAB8B, HLA-DRB1, CTSS, HLA-DMB, HLA-DQA2, HLA-DQA1, RAB33A, CD74, CD209, HLA-DRB5, HLA-DPA1, HLA-DPB1, HLA-DRA	3.80E-02
GO:0051056	regulation of small GTPase mediated signal transduction	2.08E-05	DLC1, FGD2, RHOJ, A2M, ARHGEF6, ARHGEF17, ARHGAP18, ARHGAP15, ARHGAP25, STARD13, ARHGAP30, ARHGAP31, ARHGAP6, TAGAP, RAC2, RASGRF2, SYDE1, STARD8, RHOA, CHN1, ARHGDIB, RHOH, ARHGAP9	3.82E-02
GO:0042060	wound healing	2.31E-05	COL3A1, TGFBR2, TGFB3, SPARC, ELK3, DCN, ITGB3, TPM1, TIMP1, ITGA9, SLC11A1, PECAM1, PDGFRA, PDGFRB, LOX, ENG, FN1	4.23E-02
