# Supplementary material for: Label-free quantitative identification of abnormally ubiquitinated proteins as useful biomarkers for human lung squamous cell carcinomas
Source: EPMA J. 2020 Jan 4;11(1):73–94. doi: 10.1007/s13167-019-00197-8 (PMC7028901; doi:10.1007/s13167-019-00197-8)
Supplement: Supplementary file 9 — (PDF 37 kb) [file 13167_2019_197_MOESM9_ESM.pdf]

Supplemental Table 7. The biological process (BP) of co-expressed genes of ABCC1.

GO_ID	GO Term_Description	PValue	Genes	FDR
GO_0055114	oxidation-reduction process	6.09E-09	ME1, PTGR1, PGD, UGDH, CYP26A1, OSGIN1, CYP4F11, ADH7, ALDH3A2, ALDH3A1, AKR1C3, GPX2, GSR, AKR1C2, PYCR2, G6PD, MMACHC, AKR1B10, RRM1, TXN, CYP4F3, TXNRD1, SRXN1, AKR1C1	3.48E-06
GO_0008152	metabolic process	3.84E-06	UGT1A7, GSTM2, UGT1A6, UGT1A9, GSTM4, DIP2B, CES1, UGT1A3, UGT1A4, UGT1A1, ACSF2	6.26E-04
GO_1904224	negative regulation of glucuronosyltransferase activity	3.55E-09	UGT1A7, UGT1A6, UGT1A9, UGT1A3, UGT1A4, UGT1A1	4.06E-06
GO_2001030	negative regulation of cellular glucuronidation	3.55E-09	UGT1A7, UGT1A6, UGT1A9, UGT1A3, UGT1A4, UGT1A1	4.06E-06
GO_0045922	negative regulation of fatty acid metabolic process	7.94E-09	UGT1A7, UGT1A6, UGT1A9, UGT1A3, UGT1A4, UGT1A1	3.02E-06
GO_0052697	xenobiotic glucuronidation	7.94E-09	UGT1A7, UGT1A6, UGT1A9, UGT1A3, UGT1A4, UGT1A1	3.02E-06
GO_0042573	retinoic acid metabolic process	1.21E-07	UGT1A7, UGT1A9, UGT1A3, CYP26A1, ADH7, UGT1A1	3.47E-05
GO_0052695	cellular glucuronidation	2.61E-07	UGT1A7, UGT1A6, UGT1A9, UGT1A3, UGT1A4, UGT1A1	5.96E-05
GO_0052696	flavonoid glucuronidation	1.50E-06	UGT1A7, UGT1A6, UGT1A9, UGT1A3, UGT1A4, UGT1A1	2.86E-04
GO_0034599	cellular response to oxidative stress	3.18E-04	PYCR2, G6PD, ATP2A2, MGMT, NFE2L2, SRXN1	3.57E-02
GO_0098869	cellular oxidant detoxification	4.83E-04	GPX2, GSTM2, GSR, TXN, TXNRD1, SRXN1	4.89E-02
GO_0009813	flavonoid biosynthetic process	3.09E-05	UGT1A7, UGT1A6, UGT1A3, UGT1A4, UGT1A1	4.40E-03
GO_0044597	daunorubicin metabolic process	4.26E-05	AKR1C3, AKR1C2, AKR1B10, AKR1C1	5.39E-03
GO_0044598	doxorubicin metabolic process	4.26E-05	AKR1C3, AKR1C2, AKR1B10, AKR1C1	5.39E-03
GO_0071395	cellular response to jasmonic acid stimulus	5.08E-04	AKR1C3, AKR1C2, AKR1C1	4.72E-02
