# Supplementary material for: Label-free quantitative identification of abnormally ubiquitinated proteins as useful biomarkers for human lung squamous cell carcinomas
Source: EPMA J. 2020 Jan 4;11(1):73–94. doi: 10.1007/s13167-019-00197-8 (PMC7028901; doi:10.1007/s13167-019-00197-8)
Supplement: Supplementary file 10 — (PDF 33 kb) [file 13167_2019_197_MOESM10_ESM.pdf]

Supplemental Table 8. GSEA result of VIM based on TCGA database.

KEGG Term	ES	NES	NOM p va	FDR q val	LEADING EDGE
KEGG_LEUKOCYTE_TRANSENDOTHELIAL_MIGRATION	6.08E-01	1.77E+00	0.00E+00	4.00E-02	tags=50%, list=23%, signal=64%
KEGG_ECM_RECEPTOR_INTERACTION	7.02E-01	1.76E+00	0.00E+00	4.14E-02	tags=70%, list=22%, signal=90%
KEGG_MELANOMA	5.81E-01	1.78E+00	0.00E+00	4.24E-02	tags=34%, list=17%, signal=41%
KEGG_VASCULAR_SMOOTH_MUSCLE_CONTRACTION	6.13E-01	1.80E+00	0.00E+00	4.26E-02	tags=44%, list=21%, signal=55%
KEGG_CELL_ADHESION_MOLECULES_CAMS	7.11E-01	1.76E+00	0.00E+00	4.28E-02	tags=61%, list=17%, signal=73%
KEGG_HYPERTROPHIC_CARDIOMYOPATHY_HCM	6.42E-01	1.75E+00	0.00E+00	4.32E-02	tags=55%, list=23%, signal=72%
KEGG_PATHWAYS_IN_CANCER	4.69E-01	1.80E+00	0.00E+00	4.36E-02	tags=33%, list=25%, signal=43%
KEGG_AUTOIMMUNE_THYROID_DISEASE	7.55E-01	1.73E+00	0.00E+00	4.40E-02	tags=52%, list=13%, signal=59%
KEGG_LEISHMANIA_INFECTION	7.24E-01	1.71E+00	0.00E+00	4.40E-02	tags=53%, list=16%, signal=62%
KEGG_LONG_TERM_DEPRESSION	5.75E-01	1.74E+00	0.00E+00	4.42E-02	tags=32%, list=20%, signal=40%
KEGG_APOPTOSIS	5.23E-01	1.81E+00	0.00E+00	4.43E-02	tags=41%, list=30%, signal=59%
KEGG_DILATED_CARDIOMYOPATHY	6.37E-01	1.72E+00	0.00E+00	4.50E-02	tags=56%, list=23%, signal=72%
KEGG_CYTOKINE_CYTOKINE_RECEPTOR_INTERACTION	7.17E-01	1.81E+00	0.00E+00	4.65E-02	tags=57%, list=16%, signal=67%
KEGG_MAPK_SIGNALING_PATHWAY	4.95E-01	1.81E+00	0.00E+00	4.85E-02	tags=33%, list=21%, signal=42%
KEGG_JAK_STAT_SIGNALING_PATHWAY	6.06E-01	1.81E+00	0.00E+00	4.96E-02	tags=35%, list=16%, signal=42%
