# Supplementary material for: Label-free quantitative identification of abnormally ubiquitinated proteins as useful biomarkers for human lung squamous cell carcinomas
Source: EPMA J. 2020 Jan 4;11(1):73–94. doi: 10.1007/s13167-019-00197-8 (PMC7028901; doi:10.1007/s13167-019-00197-8)
Supplement: Supplementary file 11 — (PDF 33 kb) [file 13167_2019_197_MOESM11_ESM.pdf]

Supplemental Table 9. GSEA result of ABCC1 based on TCGA database.

KEGG Term	ES	NES	NOM p va	FDR q val	LEADING EDGE
KEGG_GLUTATHIONE_METABOLISM	6.52E-01	1.93E+00	0.00E+00	2.12E-01	tags=28%, list=5%, signal=30%
KEGG_GLYCOSYLPHOSPHATIDYLINOSITOL_GPI_ANCHOR_BIOSYNTHESIS	5.67E-01	1.93E+00	1.93E-03	1.09E-01	tags=32%, list=15%, signal=37%
KEGG_PORPHYRIN_AND_CHLOROPHYLL_METABOLISM	6.58E-01	1.71E+00	1.37E-02	4.12E-01	tags=20%, list=1%, signal=20%
KEGG_METABOLISM_OF_XENOBIOTICS_BY_CYTOCHROME_P450	6.76E-01	1.64E+00	1.62E-02	5.31E-01	tags=35%, list=6%, signal=37%
KEGG_STARCH_AND_SUCROSE_METABOLISM	5.66E-01	1.57E+00	1.83E-02	4.82E-01	tags=27%, list=7%, signal=29%
KEGG_PROGESTERONE_MEDIATED_OOCYTE_MATURATION	3.96E-01	1.57E+00	1.83E-02	4.29E-01	tags=39%, list=22%, signal=50%
KEGG_PENTOSE_AND_GLUCURONATE_INTERCONVERSIONS	7.28E-01	1.62E+00	2.39E-02	4.81E-01	tags=33%, list=2%, signal=34%
KEGG_ASCORBATE_AND_ALDARATE_METABOLISM	7.19E-01	1.56E+00	3.32E-02	4.13E-01	tags=46%, list=6%, signal=48%
KEGG_BASAL_CELL_CARCINOMA	5.46E-01	1.47E+00	4.85E-02	5.77E-01	tags=31%, list=6%, signal=33%
KEGG_STEROID_HORMONE_BIOSYNTHESIS	5.57E-01	1.42E+00	4.89E-02	5.40E-01	tags=20%, list=1%, signal=20%
KEGG_GLYCOSAMINOGLYCAN_BIOSYNTHESIS_KERATAN_SULFATE	6.24E-01	1.59E+00	4.99E-02	5.04E-01	tags=33%, list=7%, signal=36%
