# Supplementary material for: Label-free quantitative identification of abnormally ubiquitinated proteins as useful biomarkers for human lung squamous cell carcinomas
Source: EPMA J. 2020 Jan 4;11(1):73–94. doi: 10.1007/s13167-019-00197-8 (PMC7028901; doi:10.1007/s13167-019-00197-8)
Supplement: Supplementary file 12 — (PDF 7 kb) [file 13167_2019_197_MOESM12_ESM.pdf]

Supplemental Table 10. Predicted E3s of vimentin.

E3	E3_GENE	SUB	SUB_GENE	HOMO	PFAM	GO	NET	MOTIF	SCORE
Q9C040	TRIM2	P08670	VIM	1	3.170	1.000	1.440	2.120	0.728
Q86TM6	SYVN1	P08670	VIM	1	1.000	1.250	2.200	3.410	0.702
O75382	TRIM3	P08670	VIM	1	3.170	1.130	1.770	1.000	0.690
Q13049	TRIM32	P08670	VIM	1	3.170	1.130	1.770	1.000	0.690
Q86YT6	MIB1	P08670	VIM	1	1.000	1.510	1.440	2.800	0.687
Q9UM11	FZR1	P08670	VIM	1	1.000	1.510	1.840	2.120	0.684
Q9UNE7	STUB1	P08670	VIM	1	1.000	3.980	1.870	1.000	0.681
Q9HCE7	SMURF1	P08670	VIM	1	1.000	2.880	1.870	1.060	0.655
P22681	CBL	P08670	VIM	1	1.000	2.330	1.550	1.060	0.625
Q2Q1W2	TRIM71	P08670	VIM	1	3.170	1.000	1.000	1.000	0.623
Q8WY64	MYLIP	P08670	VIM	1	1.000	1.130	1.000	2.800	0.623
Q13309	SKP2	P08670	VIM	1	1.000	1.000	1.440	2.120	0.619
P53804	TTC3	P08670	VIM	1	1.000	1.000	1.440	2.120	0.619
Q86YJ5	MARCH9	P08670	VIM	1	1.000	1.000	1.000	2.800	0.610
Q06587	RING1	P08670	VIM	1	1.000	1.510	1.840	1.000	0.609
O75592	MYCBP2	P08670	VIM	1	1.000	1.510	1.840	1.000	0.609
P02511	CRYAB	P08670	VIM	1	1.000	1.510	1.840	1.000	0.609
P51668	UBE2D1	P08670	VIM	1	1.000	1.510	1.840	1.000	0.609
Q9H1K0	ZFYVE20	P08670	VIM	1	1.000	1.510	1.840	1.000	0.609

Q92466	DDB2	P08670	VIM	1	1.000	1.510	1.840	1.000	0.609
Q9NWF9	RNF216	P08670	VIM	1	1.000	1.000	1.290	2.120	0.608
Q9H992	MARCH7	P08670	VIM	1	1.000	1.000	1.290	2.120	0.608
Q15542	TAF5	P08670	VIM	1	1.000	1.510	1.770	1.000	0.605
Q14241	TCEB3	P08670	VIM	1	1.000	1.510	1.770	1.000	0.605
Q13702	RAPSN	P08670	VIM	1	1.000	1.510	1.770	1.000	0.605
P61024	CKS1B	P08670	VIM	1	1.000	1.510	1.770	1.000	0.605
P46736	BRCC3	P08670	VIM	1	1.000	1.510	1.770	1.000	0.605
P35227	PCGF2	P08670	VIM	1	1.000	1.510	1.770	1.000	0.605
Q9Y2K7	KDM2A	P08670	VIM	1	1.000	1.510	2.300	1.000	0.605
Q15386	UBE3C	P08670	VIM	1	1.000	1.250	1.000	2.120	0.604
