# Supplementary material for: Label-free quantitative identification of abnormally ubiquitinated proteins as useful biomarkers for human lung squamous cell carcinomas
Source: EPMA J. 2020 Jan 4;11(1):73–94. doi: 10.1007/s13167-019-00197-8 (PMC7028901; doi:10.1007/s13167-019-00197-8)
Supplement: Supplementary file 13 — (PDF 8 kb) [file 13167_2019_197_MOESM13_ESM.pdf]

Supplemental Table 11. Predicted E3s of multidrug resistance-associated protein 1 (MRP1).

E3	E3_GENE	SUB	SUB_GENE	HOMO	PFAM	GO	NET	MOTIF	SCORE
Q96PU5	NEDD4L	P33527	ABCC1	1	1	5.73	1	6.61	0.829
Q86TM6	SYVN1	P33527	ABCC1	1	1	1.25	1	6.61	0.714
P62879	GNB2	P33527	ABCC1	1	1	5.73	1	1	0.681
P46934	NEDD4	P33527	ABCC1	1	1	5.73	1	1	0.681
Q9UKV5	AMFR	P33527	ABCC1	1	1	1.78	1	2.8	0.668
Q9HCE7	SMURF1	P33527	ABCC1	1	1	4.05	1	1.06	0.653
Q86YT6	MIB1	P33527	ABCC1	1	1	1.51	1	2.8	0.652
P22681	CBL	P33527	ABCC1	1	1	3.98	1	1.06	0.651
P43034	PAFAH1B1	P33527	ABCC1	1	1	3.98	1	1	0.646
Q9UIG0	BAZ1B	P33527	ABCC1	1	1	3.77	1	1	0.64
O00257	CBX4	P33527	ABCC1	1	1	3.77	1	1	0.64
Q9HC52	CBX8	P33527	ABCC1	1	1	3.77	1	1	0.64
Q06587	RING1	P33527	ABCC1	1	1	3.77	1	1	0.64
Q9UNE7	STUB1	P33527	ABCC1	1	1	3.77	1	1	0.64
P35227	PCGF2	P33527	ABCC1	1	1	3.77	1	1	0.64
Q8TCQ1	MARCH1	P33527	ABCC1	1	1	1.25	1	2.8	0.633
Q5T0T0	MARCH8	P33527	ABCC1	1	1	1.25	1	2.8	0.633
Q86YJ5	MARCH9	P33527	ABCC1	1	1	1.25	1	2.8	0.633
P53804	TTC3	P33527	ABCC1	1	1	1.25	1	2.8	0.633
Q14258	TRIM25	P33527	ABCC1	1	1	1.51	1	2.12	0.624
Q9UH77	KLHL3	P33527	ABCC1	1	1	1.13	1	2.8	0.623
Q9UK99	FBXO3	P33527	ABCC1	1	1	1.13	1	2.8	0.623
P35226	BMI1	P33527	ABCC1	1	1	1.13	1	2.8	0.623

Q9UPN9	TRIM33	P33527	ABCC1	1	1	2.88	1	1	0.613
O14512	SOCS7	P33527	ABCC1	1	1	2.88	1	1	0.613
Q9ULV8	CBLC	P33527	ABCC1	1	1	2.88	1	1	0.613
O60315	ZEB2	P33527	ABCC1	1	1	2.88	1	1	0.613
P29590	PML	P33527	ABCC1	1	1	2.88	1	1	0.613
Q9P1Y6	PHRF1	P33527	ABCC1	1	1	2.88	1	1	0.613
P62873	GNB1	P33527	ABCC1	1	1	2.88	1	1	0.613
Q13216	ERCC8	P33527	ABCC1	1	1	2.88	1	1	0.613
Q99732	LITAF	P33527	ABCC1	1	1	2.88	1	1	0.613
Q86Y01	DTX1	P33527	ABCC1	1	1	2.88	1	1	0.613
Q8WWQ0	PHIP	P33527	ABCC1	1	1	2.88	1	1	0.613
Q8N448	LNX2	P33527	ABCC1	1	1	2.88	1	1	0.613
Q8TBB1	LNX1	P33527	ABCC1	1	1	2.88	1	1	0.613
Q96Q27	ASB2	P33527	ABCC1	1	1	1	1	2.8	0.61
Q7Z6E9	RBBP6	P33527	ABCC1	1	1	1.25	1	2.12	0.604
Q9UM11	FZR1	P33527	ABCC1	1	1	1.25	1	2.12	0.604
Q68DV7	RNF43	P33527	ABCC1	1	1	1.25	1	2.12	0.604
Q9H6Y7	RNF167	P33527	ABCC1	1	1	1.25	1	2.12	0.604
A6NNE9	MARCH11	P33527	ABCC1	1	1	1.25	1	2.12	0.604
Q9P2E8	MARCH4	P33527	ABCC1	1	1	1.25	1	2.12	0.604
Q15386	UBE3C	P33527	ABCC1	1	1	1.25	1	2.12	0.604
Q86T96	RNF180	P33527	ABCC1	1	1	1.25	1	2.12	0.604
